# Population Structure of the Invasive Asian Tiger Mosquito, *Aedes albopictus*, in Europe

**DOI:** 10.1002/ece3.71009

**Published:** 2025-03-07

**Authors:** Margaret K. Corley, Luciano Veiga Cosme, Peter A. Armbruster, Nigel Beebe, Anna Bega, Sebastien Boyer, Beniamino Caputo, Chun‐Hong Chen, Jacob E. Crawford, Alessandra della Torre, Roger Eritja, Michael C. Fontaine, Richard J. Gill, Trang Huynh, Perparim Kadriaj, Kevin Maringer, Ademir Jesus Martins, Andrew Maynard, Shomen Mukherjee, Leonard E. Munstermann, Verena Pichler, Maria Sharakhova, Sinnathamby Noble Surendran, Sandra Urbanelli, Enkelejda Velo, Isra Wahid, Muhammet Mustafa Akiner, Georgios Balatsos, Gilles Besnard, Maria Louise Borg, Daniel Bravo‐Barriga, Rubén Bueno Marí, Francisco Collantes, Cintia Horvath, Mihaela Kavran, Raquel Medialdea‐Carrera, Tanya Melillo, Antonios Michaelakis, Ognyan Mikov, Arianna Puggioli, Elton Rogozi, Francis Schaffner, Kayleigh Hackett, Thomas Johnson, Tina Wu, João Pinto, Vera Valadas, Adalgisa Caccone

**Affiliations:** ^1^ Department of Ecology and Evolutionary Biology Yale University New Haven Connecticut USA; ^2^ Department of Entomology University of California Riverside California USA; ^3^ Department of Biology Georgetown University Washington DC USA; ^4^ School of the Environment University of Queensland Australia Brisbane Queensland Australia; ^5^ Federal State University of Education Moscow Region, Mytishchi Russia; ^6^ Vavilov Institute of General Genetics in the Russian Academy of Sciences Moscow Russia; ^7^ Medical and Veterinary Entomology Unit, Institut Pasteur du Cambodge Phnom Penh Cambodia; ^8^ Department of Public Health & Infectious Diseases Sapienza University of Rome Rome Italy; ^9^ National Health Research Institutes, National Mosquito‐Borne Disease Control Research Center & National Institute of Infectious Diseases and Vaccinology Tainan Taiwan; ^10^ Verily Life Sciences South San Francisco California USA; ^11^ Centre d'Estudis Avançats de Blanes, Spanish Research Council (CEAB‐CSIC) Blanes Spain; ^12^ Infectious Diseases and Vectors: Ecology, Genetics, Evolution and Control (MIVEGEC) Université de Montpellier Montpellier France; ^13^ Groningen Institute for Evolutionary Life Sciences University of Groningen Groningen the Netherlands; ^14^ Georgina Mace Centre for the Living Planet, Department of Life Sciences Imperial College London London UK; ^15^ Department of Medical Entomology and Zoonotics Pasteur Institute in Ho Chi Minh City Ho Chi Minh City Vietnam; ^16^ Department of Epidemiology and Infectious Diseases Control Institute of Public Health Tirana Albania; ^17^ The Pirbright Institute Pirbright UK; ^18^ Laboratóro de Biologia, Controle e Vigilância de Insetos Vetores, Instituto Oswaldo Cruz, FIOCRUZ Rio de Janeiro RJ Brazil; ^19^ Biological and Life Sciences Division, School of Arts and Sciences Ahmedabad University Ahmedabad Gujarat India; ^20^ Yale University School of Public Health, and Yale Peabody Museum New Haven Connecticut USA; ^21^ Department of Entomology and the Fralin Life Science Institute Virginia Polytechnic and State University Blacksburg Virginia USA; ^22^ Department of Zoology University of Jaffna Jaffna Sri Lanka; ^23^ Department of Environmental Biology Sapienza University of Rome Rome Italy; ^24^ Center for Zoonotic and Emerging Diseases HUMRC Faculty of Medicine Hasanuddin University Makassar Indonesia; ^25^ Department of Biology Recep Tayyip Erdogan University Fener Rize Türkiye; ^26^ Laboratory of Insects & Parasites of Medical Importance, Scientific Directorate of Entomology and Agricultural Zoology Benaki Phytopathological Institute Kifissia Greece; ^27^ Entente Interdépartementale Rhône‐Alpes pour la Démoustication (EIRAD) Chindrieux France; ^28^ Infectious Disease Prevention and Control Unit (IDCU) – Health Promotion and Disease Prevention Directorate Pietà Malta; ^29^ Parasitology and Parasitic Diseases Unit, Animal Health Department, Veterinary Faculty University of Córdoba (UCO) Córdoba Spain; ^30^ R&D Department, Laboratorios Lokímica Valencia Spain; ^31^ European Center of Excellence for Vector Control, Rentokil Initial Valencia Spain; ^32^ Department of Pharmacy, Pharmaceutical Technology and Parasitology, Parasite & Health Research Group University of Valencia Valencia Spain; ^33^ Department of Zoology and Physical Anthropology Universidad de Murcia Murcia Spain; ^34^ Department of Parasitology and Parasitic Diseases University of Agricultural Sciences and Veterinary Medicine of Cluj‐Napoca Cluj‐Napoca Romania; ^35^ Faculty of Agriculture University of Novi Sad Novi Sad Serbia; ^36^ National Center of Infectious and Parasitic Diseases Sofia Bulgaria; ^37^ Medical and Veterinary Entomology Department Centro Agricoltura Ambiente CAA “G. Nicoli” Crevalcore Italy; ^38^ Vectors' Control Unit, Department of Epidemiology and Control of Infectious Diseases Institute of Public Health of Albania Tirana Albania; ^39^ Francis Schaffner Consultancy Riehen Switzerland; ^40^ Global Health and Tropical Medicine, LA‐REAL, Instituto de Higiene e Medicina Tropical, Universidade Nova De Lisboa Lisbon Portugal; ^41^ Global Health and Tropical Medicine, Instituto De Higiene E Medicina Tropical, Universidade Nova De Lisboa Lisbon Portugal

**Keywords:** *Aedes albopictus*, disease vector, invasive species, microsatellites, population genomics, population structure, SNP chip, tiger mosquito

## Abstract

The Asian tiger mosquito, *Aedes albopictus* , is currently the most widespread invasive mosquito species in the world. It poses a significant threat to human health, as it is a vector for several arboviruses. We used a SNP chip to genotype 748 *Ae. albopictus* mosquitoes from 41 localities across Europe, 28 localities in the native range in Asia, and 4 in the Americas. Using multiple algorithms, we examined population genetic structure and differentiation within Europe and across our global dataset to gain insight into the origin of the invasive European populations. We also compared results from our SNP data to those obtained using genotypes from 11 microsatellite loci (*N* = 637 mosquitoes from 25 European localities) to explore how sampling effort and the type of genetic marker used may influence conclusions about *Ae. albopictus* population structure. While some analyses detected more than 20 clusters worldwide, we found mosquitoes could be grouped into 7 distinct genetic clusters, with most European populations originating in East Asia (Japan or China). Interestingly, some populations in Eastern Europe did not share genetic ancestry with any populations from the native range or Americas, indicating that these populations originated from areas not sampled in this study. The SNP and microsatellite datasets found similar patterns of genetic differentiation in Europe, but the microsatellite dataset could not detect the more subtle genetic structure revealed using SNPs. Overall, data from the SNP chip offered a higher resolution for detecting the genetic structure and the potential origins of invasions.

## Graphical abstract

The Asian tiger mosquito, *Aedes albopictus* , is currently the most widespread invasive mosquito species in the world and poses a significant threat to human health. We genotyped 748 *Ae. albopictus* mosquitoes from locations across Europe and Asia (the native range) to examine population genetic structure and found evidence for multiple independent invasions into Europe from both the native region and other areas of the invasive range. SNP and microsatellite datasets found similar patterns of genetic differentiation in Europe, but the microsatellite dataset could not detect the more subtle genetic structure revealed using SNPs.

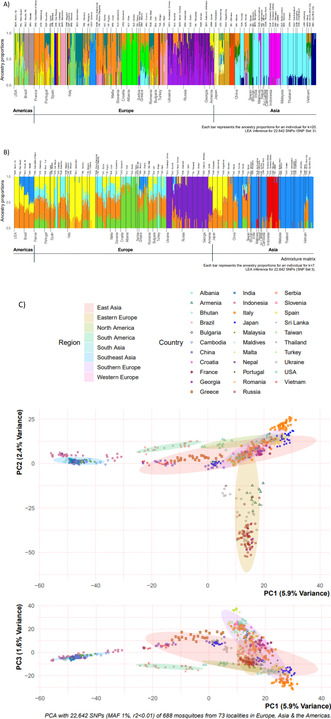

## 1 Introduction

Over the last 50 years, the Asian tiger mosquito, *Aedes albopictus* (Skuse 1984), has spread rapidly to become an invasive species in tropical and temperate areas worldwide, invading every continent except Antarctica (Kraemer et al. 2019, 2015). Its range expansion has been aided by human activities and its ability to breed in artificial containers and to feed on diverse hosts (Sprenger 1987; Swan et al. 2022). It was first observed in Europe in the late 1970s (Adhami and Murati 1987), and is now widespread there, with populations extending as far north as Germany (European Centre for Disease Prevention and Control 2023).

The spread of *Ae. albopictus* across Europe and other areas is concerning because this aggressive human‐biting mosquito is a competent vector of many arboviruses and a proven vector of dengue, chikungunya, and Zika viruses (Paupy et al. 2009; Gratz 2004). The vast extent of this species' range expansion means it holds the potential to instigate major epidemics of vector‐borne diseases, including in areas previously free of such tropical diseases. Locally initiated cases of arbovirus disease in Europe have been linked to invasive *Aedes* mosquitoes since 2007 (Rezza et al. 2007), with additional locally acquired cases of chikungunya, dengue (Tomasello and Schlagenhauf 2013), and Zika (Giron et al. 2019) occurring thereafter. With increasing range expansion and climatic changes, the occurrence of pathogen transmission by *Ae. albopictus* in Europe is only expected to grow (Schaffner et al. 2013).

There is also concern that as *Ae. albopictus* spreads, it may acquire new adaptations that will allow it to increase its population sizes and infect more people. Introduced species may acquire adaptations as their range increases, particularly if they colonize novel geographic areas or ecologies (Uller and Leimu 2011; Estoup et al. 2016). Indeed, there are indications that *Ae. albopictus* has already shifted its niche and adapted to local environments (Medley 2010; Goubert et al. 2017; Sherpa, Guéguen, et al. 2019). There is mounting evidence that it can become homodynamic (i.e., breeds year‐round) and thus over‐winter at the adult stage in areas of its invasive range, which could allow pathogens carried by it to become endemic in temperate regions (Schaffner et al. 2013; Bueno‐Marí and Jiménez‐Peydró 2015; Del Lesto et al. 2022; Collantes et al. 2014). Additionally, vector competence for arboviruses, such as chikungunya (Vega‐Rúa et al. 2020) and dengue (Brady et al. 2014), can rapidly evolve (de Lamballerie et al. 2008), and *Ae. albopictus* may also be under strong selective pressure to evolve insecticide resistance as management strategies to control arbovirus outbreaks in new areas intensify (Pichler, Malandruccolo, et al. 2019). Understanding the current population structure and past demographic history of *Ae. albopictus* has critical applications for assessing the potential risk this vector mosquito poses for human health and can offer insights into how to control these invasive populations (Unlu et al. 2013; Baldacchino et al. 2015; Caputo et al. 2016; Farajollahi et al. 2012) by enhancing efforts to determine the genetic bases of vector competence and insecticide resistance and predict invasion success in more temperate climates.

Past research has utilized a variety of genetic markers to understand the genetic diversity of *Ae. albopictus* in both its native and invasive ranges. Allozymes (Urbanelli et al. 2000), mitochondrial DNA sequences (mtDNA) (Battaglia et al. 2016; Minard et al. 2015; Usmani‐Brown et al. 2009; Mousson et al. 2005; Zé‐Zé et al. 2020; Shaikevich et al. 2023, 2018; Shaikevich and Talbalaghi 2013; Walther et al. 2017), Random amplified polymorphic DNA (RAPD) (Apostol et al. 1996), microsatellites (Minard et al. 2015; Walther et al. 2017; Manni et al. 2017; Lühken et al. 2020; Tancredi et al. 2020), ribosomal DNA (rDNA) (Shaikevich and Talbalaghi 2013; Shaikevich et al. 2018; Manni et al. 2015; Artigas et al. 2021; Gojković et al. 2019; Lucati et al. 2022), and more recently, double‐digest RADseq (ddRADseq) (Sherpa et al. 2018; Sherpa, Blum, Capblancq, et al. 2019; Kotsakiozi et al. 2017; Vavassori et al. 2022) have provided insights into population structure and phylogeography in Europe, to a degree. However, each method has various drawbacks and limitations, and the lack of a consistent set of markers across studies complicates comparisons, resulting in sometimes conflicting findings.

For example, some studies indicate that genetic diversity is much higher in the native (i.e., Southeast Asia) than in the invasive range, suggesting a loss of genetic diversity has occurred during the invasion process (Ruiling et al. 2018). However, other studies found evidence that multiple, independent introductions have resulted in relatively high levels of intra‐population variability and genetic diversity in the invasive range (Manni et al. 2017). While some markers suggest a general lack of structure in Europe, others indicate genetically distinct populations: previously, Greece and Albania were found to be genetically distinct from other parts of Europe (Manni et al. 2017; Sherpa, Blum, Capblancq, et al. 2019; Vavassori et al. 2022; Mercier et al. 2022), and two spatially isolated genetic lineages were identified in southern Russia (Shaikevich et al. 2023; Pichler et al. 2022). Some of the apparent discrepancies in the observed degree of population structure, compared to gene flow and admixture, may be due to genuine differences among regions in the history of introductions and patterns of gene flow. For instance, older established invasive populations may trace their ancestry directly to populations from the native range or other early‐established invasive populations. At the same time, areas that were more recently invaded may be characterized by admixture of multiple populations that already existed in Europe. Some data support this hypothesis: populations from Italy, which is believed to have received relatively early introduction(s) from the United States and/or Japan (Urbanelli et al. 2000; Manni et al. 2017), were found to be genetically distinct (Manni et al. 2017; Kotsakiozi et al. 2017; Pichler, Kotsakiozi, et al. 2019), while those from the Iberian Peninsula were found to have low levels of genetic structure, likely stemming from human‐mediated gene flow and multiple, on‐going introductions (Zé‐Zé et al. 2020; Lucati et al. 2022). However, the lack of consistent markers and methods across the studies and sampling locations makes it challenging to interpret the apparent differences among studies: it is often unclear whether the differences observed are genuine or merely a consequence of using different methods/markers with varying sensitivity levels. For example, mtDNA may not correctly identify the source of invasive populations due to the slower rate at which it accumulates variation (Browett et al. 2020). Using higher resolution genome‐wide markers would eliminate this source of uncertainty among studies.

To address outstanding questions and resolve some of the knowledge gaps from previous studies regarding the origin and spread of this invasive mosquito, we characterized the genetic structure of *Ae. albopictus* across Europe and the native range. We had three main objectives. Our first objective was to clarify the ancestry of *Ae. albopictus* populations across the invasive range in Europe and determine which population(s) in the native range are the most likely sources of invasion(s). Our second objective was to characterize the fine‐scale genetic structure within Europe. Our third objective was to compare the patterns of genetic structure across Europe determined using single nucleotide polymorphisms (SNPs) to patterns determined using microsatellite markers.

To achieve these objectives, we determined the distribution of genome‐wide polymorphisms in this species using the Aealbo SNP chip (Cosme et al. 2024), a species‐specific genotyping tool that targets 175,296 SNPs and was previously demonstrated to successfully determine structure in populations across the native range (Cosme et al. 2024). In contrast to previous studies, the SNP chip provides us with a more extensive set of informative genetic markers across the entire genome, allowing us to detect fine‐scale structure that may not be evident when using less sensitive genetic markers. For the first time, we applied this SNP chip to characterize the genetic structure of *Ae. albopictus* in its invasive range. Specifically, we characterized the genetic structure for 688 wild‐caught mosquitoes from 74 locations (41 in Europe, 28 in the native range, and 4 in the Americas). We used this data set to assess patterns and levels of genomic differentiation and diversity in European populations and between Europe and the native range. We included four American populations to capture secondary invasions into Europe that may have come from these continents (rather than directly from areas in the native range). We first evaluated global patterns of differentiation and invasion in Europe. Then we focused on selected European regions (Italy, Greece and Albania, the area around southern Russia, and the Iberian Peninsula) for which previous studies indicate somewhat conflicting conclusions regarding the history of invasion and spread and for which we have a good geographic representation. To gain further insight into the spread of *Ae. albopictus* into Europe over time, we also examined the extent of variation among temporally spaced populations from Italy, comparing mosquitoes collected in 1995 and 18–25 years later, in 2013–2020. In addition to using SNP data, we screened 637 mosquitoes from 25 locations in 15 European countries for genetic variation at 11 microsatellite loci and compared these results to the exact same 25 locations in our European SNP dataset. This provided the opportunity to explore how the type of genetic marker may influence conclusions about the population structure of *Ae. albopictus* across Europe, especially considering that all samples from the microsatellite dataset were from locations represented in the SNP dataset.

## 2 Materials and Methods

### 2.1 SNP Dataset

#### 2.1.1 Sample Collection and Acquisition

**FIGURE 1:**
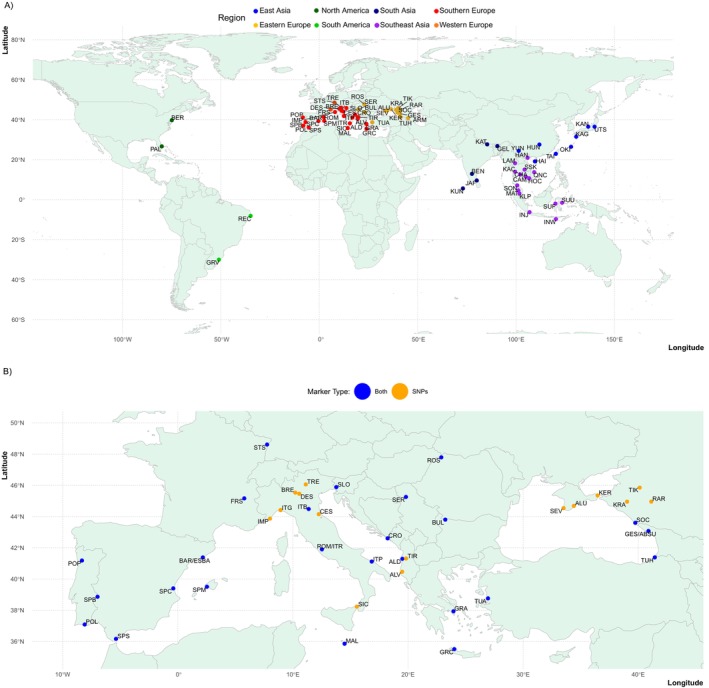
Maps of sampling locations for all genotyped *Aedes albopictus* mosquitoes. Panel A shows the 74 locations, by region, for all samples genotyped in the Global SNP Chip dataset. Panel B shows a zoomed in view of the box outlined in gray in Panel A, to highlight the locations for the 41 European sampling sites in the SNP dataset (orange) and 25 in the microsatellite dataset. Locations with samples in both the SNP and microsatellite data sets are indicated in blue. Each locality is identified by a unique three‐letter abbreviation identifying the location. Details on the location, year of collection, and the number of samples per locality for both sets of samples are reported in Tables A2 and A3 in Appendix S1.

Mosquitoes used for SNP genotyping were collected between 1995 and 2021 and shipped to Yale University for processing. When available, we attempted to genotype 12 individuals from each sampling locality. In total, we genotyped 748 mosquitoes from 32 different countries (Figure 1): 440 individuals collected from the invasive range in Europe (41 localities in 17 countries), 260 individuals collected from the native range in Asia (28 localities in 13 countries), and 48 individuals from four locations in the Americas (two in the United States and two in Brazil) to assess whether introductions to Europe may have come from previously established invasions in North or South America rather than directly from the native range (Table A2 in Appendix S1).

#### 2.1.2 DNA Extraction and Species Identification

DNA was extracted from *Ae. albopictus* adults or larvae using the DNeasy Blood and Tissue kit (Qiagen, Valencia, CA), using modifications to the manufacturer's instructions that have been previously described for the purification of total DNA from insects (Cosme et al. 2024). For larvae and other samples that could not be identified to species using morphology (*N* = 75), we checked species assignments by sequencing a 1537 bp fragment of the mitochondrial cytochrome oxidase subunit 1 COI gene and comparing it to the species known sequences (File S3). The protocols for PCR amplification and sequencing have been detailed previously (Cosme et al. 2024). Only samples confirmed to be *Ae. albopictus* were sent for genotyping.

#### 2.1.3 SNP Chip Genotyping

All SNP samples were genotyped using the Aealbo microarray (Affymetrix, Santa Clara, CA) in the Functional Genomics Core at the University of North Carolina at Chapel Hill. We obtained genotype calls using Axiom Analysis Suite Software v.5.1.1, running the “Best Practices Workflow” and the off‐target variants (OTV) caller algorithm to identify miscalled clusters. Thresholds and other details used for quality control during data processing in Axiom Analysis Suite have been described previously (Cosme et al. 2024).

We performed two distinct genotype calls: first, using the full dataset of samples from European locations and global data (from the native range and the Americas), which we refer to as the “Global” dataset, and second, exclusively on the samples from European locations, which we refer to as the “Europe” dataset. These genotype data were exported in VCF file format and utilized for subsequent quality control filtering. The raw data used to obtain genotype calls are available at Zenodo (see Data Accessibility Statement for details).

### 2.2 Microsatellite Dataset

For microsatellite analyses, 637 mosquitoes were collected between 2011 and 2020 from 25 locations in 15 European countries (Figure 1B). The number of individuals genotyped from each population ranged from 5 to 60 (mean = 26.5 mosquitoes; Table A3 in Appendix S1). Additional details on sample collection are provided in Files S1 and S2.

All samples were genotyped at 11 microsatellite loci using previously published primers and protocols (Manni et al. 2017), with minor modifications. Specifically, PCR products were multiplexed in groups of two or three according to fluorescent dye (FAM, NED, HEX) and size interval and subjected to fragment analysis on an ABI 3130xl DNA analyzer (Applied Biosystems) at the Yale DNA Analysis Facility at Science Hill. At least three positive controls were used to account for inter‐run variations in fragment size. Alleles were scored from electropherograms using the software GENEMARKER (SoftGenetics) (Hulce et al. 2011). Additional details of the microsatellite loci and extraction and amplification methods are outlined in File S3.

### 2.3 Statistical Analysis

#### 2.3.1 SNP Data Quality Control

After genotyping, we used stringent quality control measures to filter the samples and SNPs used in downstream analyses using Plink v 1.9 or v 2.0 (Chang et al. 2015; Chen et al. 2019). Specifically, we removed all SNPs that did not uniquely map to autosomes or failed segregation tests based on laboratory crosses (as detailed in (Cosme et al. 2024)). We also removed (1) loci with more than 10% missingness, (2) loci with a minor allele frequency smaller than 10% (or 1% for use in population structure analyses), (3) loci that failed Hardy–Weinberg tests with a threshold of 0.00001 for each population, (4) individuals with more than 20% missing loci, (5) individuals whose expected heterozygosity values deviated more than ±4 standard deviations from the mean of all samples, which might indicate low DNA quality, contamination, or high inbreeding, and (6) related individuals using a KING kinship coefficient of > 0.354 to identify monozygotic twins and duplicate samples. Additional details of the quality control steps are described in Files S6 and S7.

#### 2.3.2 Linkage Disequilibrium

Linkage disequilibrium (LD), the correlation of frequencies of genes at different loci, can be increased in populations during bottleneck events and thus vary among populations depending on their history (Wang et al. 1998). We used PopLDDecay (Zhang et al. 2019) to calculate the half distance of maximum *r* ^2^ (LD half‐life) for the SNP dataset. As a correlation between sample size and LD estimates has previously been observed for *Ae. albopictus* populations in the native range (Cosme et al. 2024), we compared the half distance of the maximum *r* ^2^ value only for populations in Europe and the native range with at least 10 individuals. To be consistent with previously published analyses, for populations with more than 10 individuals we followed the same method used to standardize sample sizes for LD estimates of *Ae. albopictus* in the native range: we randomly selected ten individuals to retain and dropped the excess individual(s) from analyses. We then compared patterns of LD decay among the sampling locations and broader geographical regions (e.g., Europe vs. the native range) by plotting the LD decay for each area for each of the three chromosomes with the ggstatsplot package in R (Patil 2021). Additional details of linkage disequilibrium analyses are in File S8.

#### 2.3.3 SNP Sets

We were interested in retaining relatively rare alleles that may allow us to better distinguish between sampling locations. Therefore, we created a set of SNPs (with LD half‐life pruning at *r* ^2^ < 0.01) that only removed SNPs with a minor allele frequency (MAF) of less than 1%. This dataset contained 22,642 variants for the global dataset and 20,968 variants for the Europe dataset (Figure A2 in Appendix S1).

For all population structure and differentiation analyses (described below), we report results for this SNP Set, which we called SNP Set 3 (filtered for LD half‐life *r* ^2^ < 0.01 and MAF > 0.01). We also provide results from other sets of SNPs with different LD half‐lives and MAF filtering criteria for select analyses in the supplementary files and Appendix figures for comparative purposes (see Table A1 in Appendix S1).

#### 2.3.4 Population Structure and Differentiation

To assess the relationship between European mosquito samples and those in the native range, we included samples from 13 countries in eastern, southern, and southeastern Asia. These samples were previously analyzed by a study that examined the accuracy of the Aealbo SNP chip and the population structure of *Ae. albopictus* mosquitoes in the native range (Cosme et al. 2024). Since previous research found evidence that some introductions of *Ae. albopictus* to Europe may have originated in the Americas (Artigas et al. 2021; Vavassori et al. 2022), we included two locations from the United States and two from Brazil, which had not previously been analyzed. We performed structure analyses on the two SNP data sets: (1) the European, native range, and American samples combined (“Global” dataset, *N* = 688), and (2) the European samples alone (“Europe” dataset, *N* = 410).

We combined several methods to explore genetic structure and identify related clusters of samples in our data. First, to identify major patterns of variation and visualize genetic distances among samples from different locations, we used principal component analysis (PCA), which was implemented using the R package LEA (Frichot and François 2015). We then used the R package adegenet to perform discriminant analysis of principal components (DAPC) (Jombart 2008; Jombart et al. 2010). For DAPC analyses, we used the cross‐validation function to choose the number of principal components (PCs) to retain. For both PCA and DAPC, we carried out analyses on both the Global and Europe datasets, as well as different subsets of European populations. We also repeated these analyses for the subset of 242 mosquitoes from the 24 locations overlapping with the microsatellite dataset. Step‐by‐step procedures for these analyses are presented in Files S12–S14.

To further evaluate population structure, we used three different clustering algorithms: Admixture (Alexander and Lange 2011), fastStructure (Raj et al. 2014), and LEA (Frichot and François 2015), on the Global and Europe datasets to cross‐check consistency across methodologies and ensure our findings were robust. To facilitate comparison with the microsatellite dataset, we repeated the LEA structure analysis for a subset of 242 mosquitoes from the locations that were shared (i.e., overlapping) with the microsatellite dataset. Parameters used to run each algorithm are listed in Table A6 in Appendix S1, and step‐by‐step procedures for these analyses are presented in Files S9 and S10.

To quantify genetic differentiation among sampling locations, we used the R package StAMPP (Pembleton et al. 2013) to estimate the pairwise genetic differentiation (*F*ST), estimating *F*ST values across each locus according to Weir & Cockerham (Weir and Cockerham 1984), taking into account population size and using 100 bootstraps to estimate *p* values and confidence intervals. For all *F*ST analyses, we used only locations with at least four individuals (Files S19 and S20). We also examined potential patterns of isolation by distance (IBD) among European populations, using a Mantel test to assess the correlation between genetic and geographic distances using the mantel.randtest function in the R package ade4 (Dray and Dufour 2007). For IBD analyses, genetic distance was calculated on the basis of gene frequencies in the R package adegenet (Jombart 2008). We also repeated all *F*ST and isolation by distance analyses for the subset from the 24 locations with four or more individuals that overlapped with the microsatellite dataset to facilitate comparisons between marker types.

#### 2.3.5 Microsatellites

We used several approaches to characterize population structure and determine the number of genetically distinct populations represented by our microsatellite data set. First, we analyzed individuals using the Bayesian clustering method of the software STRUCTURE v 2.3.4 (Pritchard et al. 2000; Falush et al. 2003, 2007; Hubisz et al. 2009). We carried out two versions of the analyses: the first used only genetic information to cluster our data, and the second used sampling locations as prior information to assist clustering (LOCPRIOR method). This second method is appropriate for data sets with few markers, few individuals, or where the signal of structure is relatively weak. We employed this second model to ensure that we could detect subtle population structures that may be too weak for standard structure models to detect using our 11 loci. We used an admixture model for both analyses and set parameters to 1,000,000 MCMC runs, with a burn‐in of 100,000 and 10 replicates of each possible number of clusters (K) from 2 to 25. To assess the STRUCTURE results, we used the Evanno method (Evanno et al. 2005) implemented in CLUMPAK (Kopelman et al. 2015). Then we plotted the proportions of ancestry for each individual, grouped by sampling location, in R. Additional details of STRUCTURE analyses can be found in File S3.

We complemented clustering analyses with multivariate approaches, including PCA and discriminant analysis of principal components (DAPC). PCA was implemented in the package LEA (Frichot and François 2015), and DAPC in the package adegenet (Jombart 2008; Jombart et al. 2010) in R, using the cross‐validation function to choose the number of principal components (PCs) to retain. We also repeated analyses using various subsets of European populations to better visualize the clusters among samples in specific regions of Europe (File S11).

We evaluated the level of genetic differentiation among European sampling locations by estimating *F*ST values using the Weir and Cockerham method (Weir and Cockerham 1984), as implemented in the R package hierfstat (Goudet 2005). Using the mantel function in the R package vegan (Oksanen et al. 2022), we also used the Mantel test to assess the correlation between genetic and geographic distances (Legendre and Legendre 2012). As with the SNP datasets, we only utilized populations with at least four individuals.

## 3 Results

### 3.1 Genotyping and Quality Control

Of the 768 samples in the global dataset submitted for genotyping, 712 mosquitoes passed all quality control criteria in the Axiom Analysis Suite Software's “Best Practices Workflow” and 113,823 variants were recommended after running the Off Target Variant (OTV) caller. Of the 454 samples in the Europe dataset submitted for genotyping, 423 passed all quality control criteria, and 117,981 SNPs were recommended after the OTV caller. Additional details of the quality control methods and workflow used in Axiom Analysis Suite are outlined in Files S4 and S5.

The SNP sets exported were subjected to additional quality control measures (see Files S6 and S7). Filtering with Plink to remove the 2047 SNPs that failed the segregation test, variants missing in more than 10% of samples, and variants with a minor allele frequency < 10% resulted in a SNP set of 87,183 variants for the global dataset and 85,306 variants for the Europe dataset (Figure A1 in Appendix S1). For both datasets, all variants passed the Hardy–Weinberg Equilibrium test. Additional details of the SNP sets are shown in Tables A4, A5 in Appendix S1.

No individuals were missing > 20% of variants. Four samples in the 712 genotyped individuals in the global dataset were duplicates and were thus removed (two from KAG and two from BEN, Table A2 in Appendix S1). Of the 708 remaining samples, we removed nine with heterozygosity that deviated more than 4SD from the mean and eleven with high relatedness (> 0.354), leaving 688 individuals in the final global dataset for subsequent analyses. Of the 423 European individuals genotyped, we removed five samples for having heterozygosity > 4 SD from the mean and nine for having high relatedness (> 0.354), leaving 410 individuals in the Europe dataset. A summary of the number of individuals retained in each dataset is presented in Table A2 in Appendix S1.

### 3.2 Linkage Disequilibrium

We compared the half distance of the maximum *r* ^2^ value for populations with at least ten individuals (*N* = 41 populations in 24 countries; File S8). The LD half‐life estimates varied among locations within the European and native ranges, and the degree of LD also differed across the three chromosomes (Figures A3 and A4 in Appendix S1). The LD half‐distance tended to be the smallest and showed the least variability among populations for chromosome 1, while chromosome 2 had the most significant variability and the highest LD half‐distance estimates (Figure A3 in Appendix S1). Longer LD half‐distance estimates on chromosome 2 indicated that linkage persists over greater distances, possibly due to a lower recombination rate or selection patterns on this chromosome.

There was not a consistent correlation between patterns of LD decay and geographic region. The mean half distance of the maximum *r* ^2^ value was somewhat higher for European compared to Asian populations for chromosomes 1 and 3. In contrast, Asian populations' mean values were slightly higher than European ones for chromosome 2. However, none of these differences was statistically significant (Figure A4 in Appendix S1).

### 3.3 Genetic Ancestry

#### 3.3.1 Global Dataset

**FIGURE 2:**
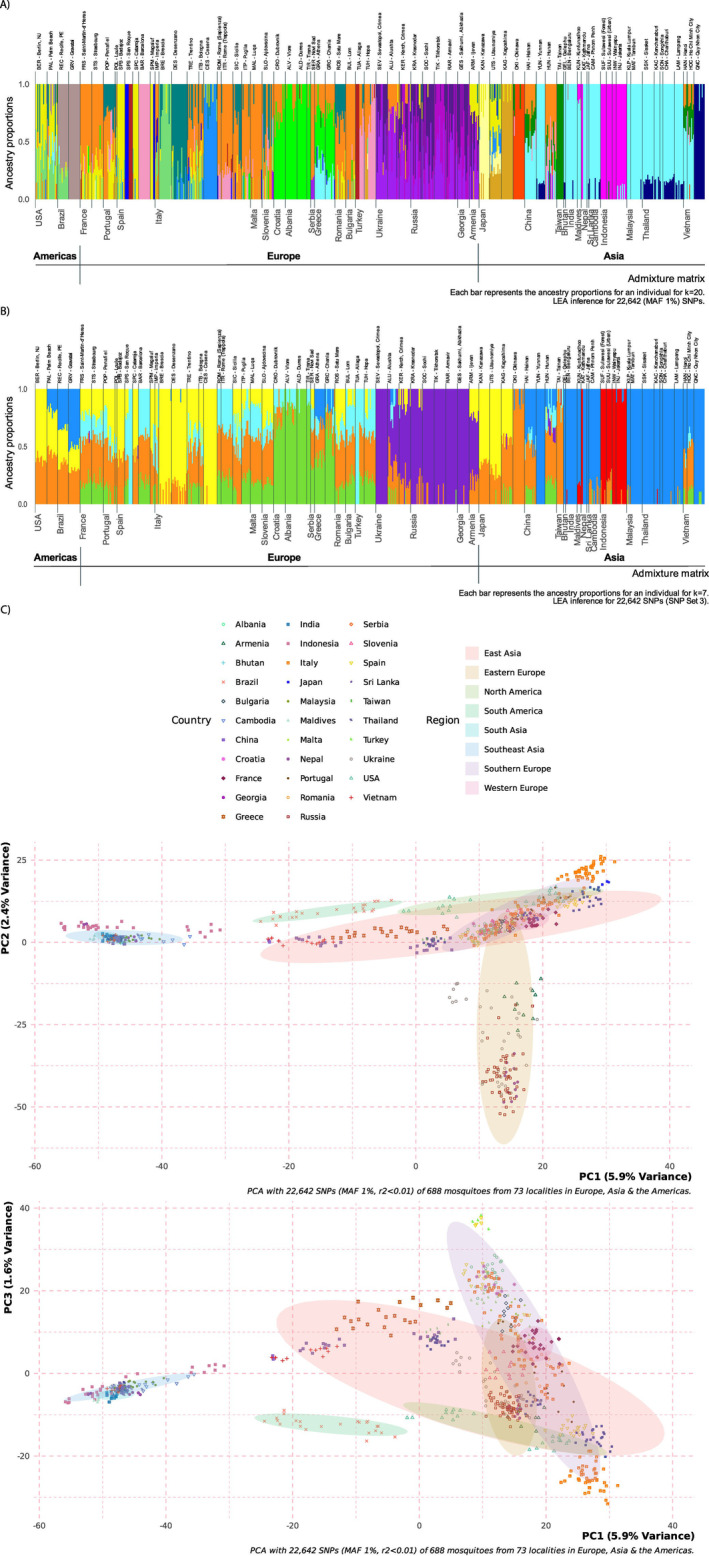
The population structure shows the best‐supported number of clusters (K) identified for 688 mosquitos sampled from American, European, and native locations (K = 20) (Panel A). Panel B contains the same data shown in A, but plotted for K = 7. In A and B each vertical bar on the *x*‐axis represents one mosquito, and the *y*‐axis shows the proportion of admixture for each individual ancestral genetic group. Panel C shows scatterplots of principal component analysis (PCA) showing the first PC on the *x*‐axis and PCs 2 and 3 on the *y*‐axes. Each symbol represents a mosquito, and the color and shape of the symbol indicates the country where they were sampled. Ellipses mark each region covering 80% of the samples. All plots were created in the R package LEA using data from SNP Set 3. Plots for the best K obtained from other structure algorithms (fastStructure and admixture) and using alternate SNP sets are shown in Figures A6–A9, Table A7 in Appendix S1, and Files S10 and S17.

The three clustering algorithms indicated a K value between 20 and 23 as the best number of ancestral clusters for the global dataset (Table A7 in Appendix S1). Figure 2A shows a representative diagram of the clustering result for K = 20. There was evidence of at least some admixture in most European sampling locations and many native range and American populations. Nonetheless, there was still clear evidence of population structure (Figure 2 and Figure A5 in Appendix S1).

Three clusters observed in the K = 20 plot were present in both Europe and the native range. Specifically, the orange cluster, which was the most prominent in many southern and western European locations, was also observed in China (Hunan and Hainan) and, to a lesser extent, in Vietnam. The bright yellow cluster in the Iberian Peninsula, France, Armenia (and mixed in small amounts in other European locations) was also observed in the United States and in Japan (Utsonomyia). Finally, Greece showed evidence of admixture with the cyan cluster found in the south and southeast Asian locations and parts of China, particularly Yunnan.

Several European clusters were either absent or poorly represented in the native range. For example, the three Italian locations with historic samples collected in the 1990s each formed a cluster, with the teal cluster present in Desenzano (from 1995) also observed as admixture in other European regions, but not in the native range. The light green cluster that constituted most of the ancestry in another Italian location (Brescia, collected in 1995) was found admixed among individuals in both the United States and Recife, Brazil, but was completely absent from Asia. A bright green cluster observed primarily in Croatia, Albania, and Greece was also absent from the native range. Notably, the purple clusters were confined to the most eastern European locations, suggesting mosquitoes in this region were entirely distinct not only from populations sampled in the native range but also from other parts of Europe. The same was true for the blue and red clusters observed only in Spain and Aliaga, Turkey (Figure 2A). Ancestry matrices derived from other algorithms showed similar patterns (Figure A5 in Appendix S1).

**FIGURE 3:**
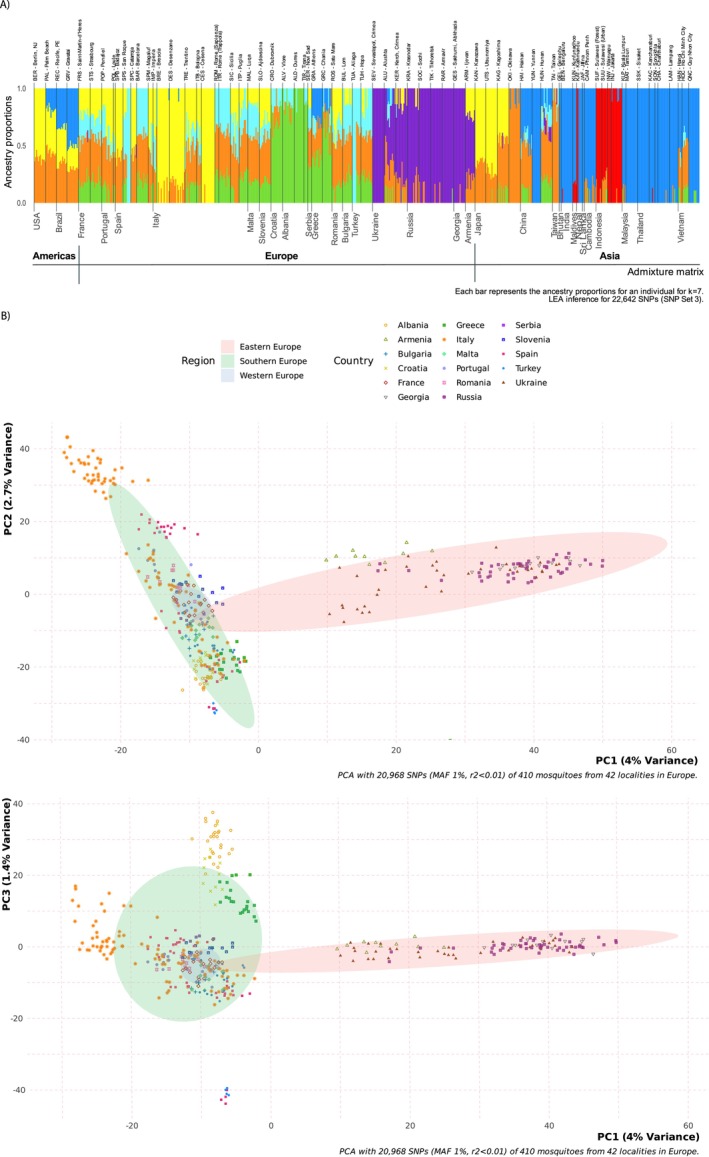
Panel A shows population structure for the best‐supported number of clusters (K) identified for 410 mosquitos sampled from 41 European locations (K = 14). Each vertical bar on the *x*‐axis represents one mosquito, and the *y*‐axis shows the proportion of admixture for each individual ancestral genetic group. Panel B shows scatterplots of principal component analysis (PCA) showing the first PC on the *x*‐axis and PCs 2 and 3 on the *y*‐axes. Each symbol represents a mosquito, and the color and shape of the symbol indicates the country where they were sampled. Ellipses mark each region covering 80% of the samples. All plots were created using LEA with data from SNP Set 3. Plots for the best K obtained from other structure algorithms (fastStructure and admixture) and using alternate SNP sets are shown in Figures A10 and A11, Table A7 in Appendix S1, and Files S9 and S16.

The PCA of the global dataset indicated that mosquitoes from South and most of Southeast Asia clustered together, separately from other samples, except for some individuals from China. Samples from South America also formed a cluster, although this cluster was less distant from the remaining samples than those formed by South and Southeast Asia. In contrast, East Asian samples spread along PC 1 substantially overlapped with Southern and Western European samples. Samples from North America also overlapped with this cluster, particularly with mosquitoes from Southern Europe and Japan. Mosquitoes in Eastern Europe formed two distinct clusters, with some populations overlapping with East Asia and Southern Europe, and the four farthest east locations (Russia, Georgia, Armenia, and Ukraine) forming their cluster at the bottom of PC2. However, when PC3 was plotted against PC1, these four eastern locations overlapped with parts of Southern Europe and East Asia (Figure 3B).

DAPC, which uses discriminant functions to accentuate the genetic differences between groups, identified six clusters in the SNP global dataset. One central cluster consisted of most samples from southern and western Europe, locations in eastern Europe west of the Black Sea, and most of East Asia. As with the PCA, South and Southeast Asia formed their own cluster, which overlapped with some East Asian samples. The four samples from Serbia also clustered somewhat apart from other populations. The three groups of mosquitoes most distinguished from the central cluster were samples from 1‐the most eastern locations in Europe, 2‐Albania, Croatia, Greece, and 3‐Brazil (Figure A7 in Appendix S1). Plotting additional PCs (i.e., PC3 and 4) derived from the DAPC analyses showed similar patterns of clustering but also indicated that Indonesian samples were separate from the rest of the southeast Asian cluster (Figure A7.B in Appendix S1; File S14).

PCA and DAPC analyses from all three SNP Sets for the global dataset were very similar. Scatterplots of additional principal components and other SNP Sets are presented in Files S13, S14 and Figures A6 and A7 in Appendix S1. Additional plots highlighting PCA results for specific regions are also presented in Figure A8 in Appendix S1.

As we were interested in investigating links between mosquitoes sampled in Europe and populations in the native range, we also plotted the ancestry matrices for K = 6 and K = 7 to visualize how locations in the European invasive range may group with Asian locations. We chose to examine structures with six and seven clusters because our PCA and DAPC analyses (see Figures A6–A8 in Appendix S1) indicated that samples from the most eastern locations in Europe, and possibly also samples from Croatia, Albania, and Greece, may each represent a distinct cluster not defined in the five clusters found in the native range populations in our dataset (Cosme et al. 2024).

When the structure analyses for the global dataset were plotted for K = 6 and K = 7, we observed that most European clusters were also present in the native range. However, some were represented only by small amounts of admixture (Figure 2C, Figures A9 and A11.B in Appendix S1). The three historic Italian locations shared a cluster with northern Japanese locations (yellow). This yellow cluster was also observed admixed in western and southern Europe and the Americas, particularly the United States. Greece, Brazil, and Palm Beach, United States showed admixture with the primary cluster observed in most south and southeast Asian locations (blue). Taiwan, Okinawa, and parts of China (Hunan and Hainan) primarily comprised the orange cluster, which was also observed broadly across modern samples in eastern and southern Europe and a few locations in eastern Europe (e.g., Alushta, Ukraine). However, Hunan and Hainan, China also contained admixture with the green cluster found in samples from Croatia, Albania, and Greece. The red cluster, represented in Indonesia and some samples in Nepal, was present only in Asia and did not appear substantially in Europe or the Americas. The purple cluster was mainly represented in the most eastern European samples and was observed elsewhere only in small proportions of admixture in some eastern Asia locations (Figures 2C and 3, Figure A9 in Appendix S1). When a seventh cluster was identified (cyan), it was represented primarily in San Roque, Spain, and Aliaga, Turkey (Figure 2C and Figure A9.B in Appendix S1), which corresponds to the dark red cluster identified in the K = 20 structure plot (Figure 2A). Table A7 and Figure A9 in Appendix S1 present additional details of the structure analyses for different SNP Sets for comparison (also see Figures A16–A18 in Appendix S1).

#### 3.3.2 European Datasets

When European locations were analyzed on their own, the composition of clusters was very similar to the clusters found in the global dataset. For the European dataset, our three algorithms indicated a K value between 13 and 15 as the number of ancestral clusters for all but one set of analyses (data from SNP Set 3 run with fastStructure indicated K = 18; Table A7, Figure A10 in Appendix S1). Figure 3A shows a typical clustering result for K‐14. While most sampling locations showed some admixture, there was again clear evidence of structure among European populations.

One cluster (orange) had a relatively broad distribution, found throughout many locations in western and southern Europe and some parts of eastern Europe west of the Black Sea (e.g., Bulgaria, Romania). The three Italian populations sampled in the 1990s each formed unique clusters. Clusters from these historic Italian samples, especially the one in Desenzano (teal), were also found admixed in several modern populations in Italy (e.g., Sicily) and elsewhere in Europe (e.g., Portugal, Romania, Slovenia). Mosquitoes from Croatia and Albania (bright green) and Greece (dark green) formed two distinct clusters. However, in some algorithms' analyses, Greek samples were grouped with the Croatia–Albania cluster (Figure A10 in Appendix S1). Serbia formed a cluster (brown), which was not found in any other locations (Figure 3A).

The four countries east of the Black Sea, Ukraine, Russia, Georgia, and Armenia (hereafter referred to as the farthest east), were characterized by a mixture of two to three clusters (purple shades) not found in any other part of Europe (Figure 3A and Figures A10, A11 in Appendix S1). Turkish samples, including those from eastern Turkey (Hopa), had no admixture with these purple clusters, despite being very geographically close to the four farthest east European countries. Instead, the Turkish samples were primarily grouped with mosquitoes sampled in Spain. The Iberian Peninsula comprised five main clusters, though the Portuguese samples showed admixture with several other clusters (primarily those found in Desenzano). Spanish samples showed comparatively little admixture; individuals in all five Spanish sampling locations were comprised primarily of a single cluster. The cluster in Barcelona (pink) was shared with Turkish samples and showed admixture with some modern Italian populations. The most southern Spanish location, San Roque, consisted of two distinct sets of individuals, each forming a unique cluster: blue and red. Some individuals shared the blue cluster in Magaluf, Spain, and the red cluster was shared by about half the individuals sampled in Aliaga, Turkey. The final cluster found in Spain (yellow) was detected in three locations: Catarroja, Badajoz, and Magaluf. This yellow cluster was also observed in admixture in the two French locations, some Italian locations, Slovenia, Romania, and the farthest east, particularly Armenia (Figure 3A). For comparison, additional details of the structure analyses for different SNP Sets are presented in Table A7 and Figures A10, A11 in Appendix S1 (also see Figures A16–A18 in Appendix S1).

The PCA of the European dataset alone allows for visualization of finer scale structure among European populations. Specifically, it showed that a subset of Italian samples (i.e., those from the three locations sampled in 1995) forms a cluster separate from other Italian or European populations (at the top of PC2 in Figure 3 and Figure A12 in Appendix S1). Plotting PC3 against PC1 indicated that Albania, Croatia, and Greece also clustered together, apart from other countries. Locations in Eastern Europe were spread along PC1, apart from Western and Southern Europe. As in the global dataset, mosquitoes from the four countries farthest to the east (Armenia, Georgia, Russia, and Ukraine) showed no overlap with other regions of Europe, even when additional PCs (i.e., PC2, PC3) were examined (Figure 3 and Figure A12 in Appendix S1). The DAPC showed similar clustering patterns, but also indicated that Serbian samples were somewhat distinct from the rest of Europe (Figure A13 in Appendix S1).

Overall, PCA and DAPC analyses from all three European SNP Sets were very similar. Scatterplots and pie charts of other SNP Sets are presented in Files S12–S14 and Figures A10–A12 in Appendix S1. Additional plots highlighting PCA results for specific European regions are also presented in Figure A8 in Appendix S1.

#### 3.3.3 Genetic Differentiation

When we estimated genetic differentiation (*F*ST) for all localities with at least four individuals (*N* = 69 for European and native range; *N* = 40 for Europe), the overall patterns of *F*ST were similar for all three SNP sets. *F*ST analyses for the other SNP sets are detailed in Files S19 and S20, and results are shown in Figures A14–A16 in Appendix S1.

When European and native range countries were examined together, Serbia was the European country with the highest *F*ST (mean = 0.23, range 0.15–0.44), and the Maldives was the native range country with the highest *F*ST (mean = 0.25, range 0.13–0.44; Figures A14.C and A15 in Appendix S1). When European regions were examined on their own, the mean *F*ST was similar for Southern (0.10) and Eastern Europe (0.11), and slightly lower for Western Europe (0.08). Pair‐wise *F*ST among European countries ranged from 0.01 to 0.23, with Serbia again having the highest mean *F*ST (0.20, range 0.15–0.23), and four countries in eastern Europe (Russia, Ukraine, Georgia, and Romania) having the second highest (mean *F*ST for each of the four countries = 0.11; Table A8; Figure A14.B in Appendix S1).

#### 3.3.4 Isolation by Distance

We created a matrix with the *F*ST values and geographical distance in kilometers for all European locations and fit a linear regression to our *F*ST estimates. The *F*ST values were generally higher as the distance between the European sampling sites increased. Still, the correlation was only about 4% (*R* ^2^ for *F*ST vs. distance = 0.04), indicating that only a small proportion of variation in *F*ST could be attributed to geographic distance (Figure A17.A in Appendix S1).

**FIGURE 4:**
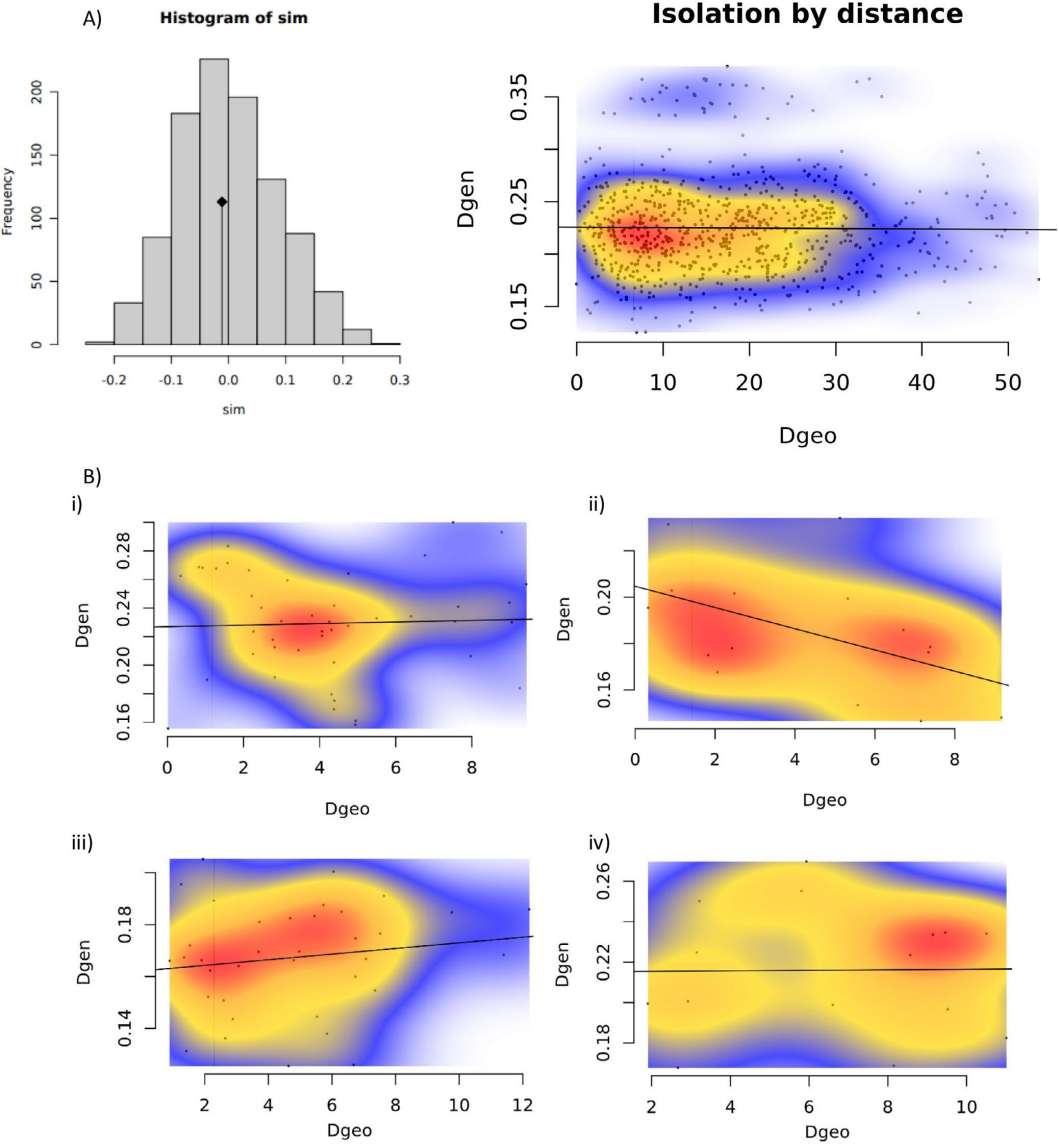
Panel A Plots of the Mantel test for isolation‐by‐distance (IBD) using the 40 European populations with at least four mosquitoes (Table A3 in Appendix S1) for SNP Set 3 (20,968 SNPs). The histogram shows the distribution of correlation coefficients between genetic (Dgen) and geographic (Dgeo) distances obtained from 999 random permutations. The arrow indicates the observed correlation coefficient (−0.011) derived from the data, which suggests no significant deviation from random expectations (*p* < 0.519). The scatterplot shows geographic distance (Dgeo) against genetic distance (Dgen). The line from the linear regression model fit to the data had an *R* ^2^ = 0.01. The density of overlapping points is represented by color, with warmer shades indicating more overlap. Panel B shows scatterplots of geographic distance (Dgeo) against genetic distance (Dgen) for subsets of European samples by region: (i) Italy (*R* ^2^ = 0.0), (ii) Greece, Albania and Croatia (*R* ^2^ = −0.25), (iii) Eastern Europe (*R* ^2^ = 0.02), and (iv) Iberian Peninsula (*R* ^2^ = 0.0). Additional plots for subsets are shown in Figures A18 and A19 in Appendix S1.

There was also no strong evidence for isolation by distance (IBD) in Europe when allele frequencies were used to estimate genetic distance. When we fitted a linear regression model for all 40 European locations, the correlation coefficient (*R* ^2^) was 0, indicating that variation in genetic distance cannot be explained by geographic distance among European populations (Figure A17.B in Appendix S1). Mantel test observations for the European SNP data were slightly negative (−0.01, *p* value = 0.52), indicating that we cannot reject the null hypothesis that geographic and genetic distance are uncorrelated (Figure 4A). Patterns of IBD were similar for all three SNP sets, and analyses for SNP Sets 1 and 2 are detailed in Files S19 and S20. There was also no strong evidence for IBD when looking at regional subsets of European populations (Figure 4B).

### 3.4 Microsatellites and Overlapping SNP Dataset in Europe

We carried out population structure and differentiation analyses for the microsatellite dataset for 637 mosquitoes in 24 European locations. Then, to facilitate comparisons between the two sets of markers (microsatellites and SNPs), we repeated all clustering and genetic differentiation analyses for SNPs using data from the “overlapping” subset of 24 locations sampled with both types of markers (Figure 1, Tables A2 and A3 in Appendix S1).

### 3.5 Microsatellites: Population Structure and Differentiation

**FIGURE 5:**
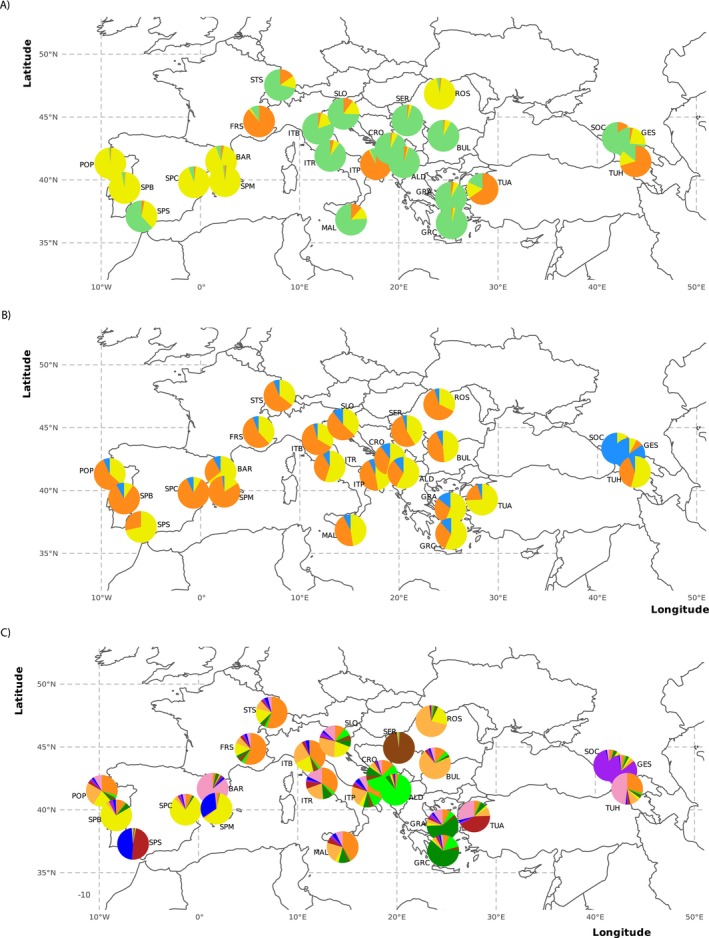
Maps of the ancestry matrices obtained using the 24 locations shared between the microsatellite and SNP datasets. The top panel shows results when the clustering analysis was run for 637 mosquitos in the microsatellite dataset using only genetic data for inferencing in STRUCTURE (best supported number of clusters: K = 3). The middle panel show the clusters obtained using LEA to analyze structure for the same 24 locations with SNP Set 3 dataset. For the SNP data, K = 3 is shown to facilitate comparison to microsatellites. K = 10, which was the best supported number of clusters for SNPs is shown in the bottom panel. For more detailed maps showing sampling locations in the microsatellite dataset see File S18.

The results of the STRUCTURE analyses for the microsatellite dataset (*N* = 24 locations) indicated that the best‐supported number of clusters was K = 3, and there was relatively little admixture in most locations (Figure 5 and Figure A20.A in Appendix S1). The best‐supported number of clusters was also K = 3 when both genetic and population information were used to analyze this same subset for the microsatellite dataset (Figure A20.B in Appendix S1). An orange cluster mainly constituted four locations (Saint‐Martin‐d'Hères, France, Puglia, Italy, and the two Turkish locations), a yellow cluster accounted for most of the ancestry in Portugal, Romania, and all but one of the five Spanish locations (San Roque), and the remaining locations were mainly composed of a third (green) cluster (Figure 5 and Figure A20.A,B in Appendix S1).

The PCA of microsatellite data using the subset of 24 overlapping locations showed most samples clustered tightly together, except for Western Europe (France) and some individuals from Italian and Turkish locations, which were spread out along the first PC axis. Some samples from Portugal and one location from Spain also plotted somewhat separately from others (Figure A22.B in Appendix S1). Scatterplots of the first two principal components derived from DAPC for the microsatellite dataset showed a similar pattern (Figure A23.B in Appendix S1).

Regional estimates of average *F*ST were slightly higher for Eastern (0.10) than for Western or Southern Europe (0.10). Russia had the highest mean pairwise *F*ST (0.12), while Slovenia and Malta had the lowest (0.07) (Figures A24.B, A25.B; Table A9 in Appendix S1). The correlation between *F*ST values and geographic distance was minimal (*R* ^2^ = 0.02; Figure A26.B in Appendix S1). When we fit a linear regression model to the genetic and geographic distance, the correlation coefficient (*R* ^2^) was 0.01 (Figure A26.B in Appendix S1; File S20). There was no evidence for isolation by distance (IBD): the Mantel test observation was slightly negative (−0.009; *p* value = 0.52), indicating that we cannot reject the null hypothesis that geographic and genetic distance are uncorrelated for this dataset (Figure A27.B in Appendix S1).

#### 3.5.1 Overlapping SNP Dataset: Population Structure and Differentiation

When the subset of 24 overlapping locations with SNP data was used in clustering analyses using the R package LEA (*N* = 242 mosquitoes), the best‐supported number of clusters was K = 10 (Figures A20.C and A21 in Appendix S1). To facilitate comparisons between datasets, K = 3 was also plotted. At K = 3, Russia and Georgia formed a distinct cluster (blue), and three Spanish locations (Badajoz, Catarroja, and Magaluf) were composed almost entirely of one cluster (orange). All other locations showed substantial admixture between the orange and yellow clusters (Figure 5 and Figure A20.D in Appendix S1).

Plots of the first two PCs showed that Eastern Europe (Russia and Georgia) and some individuals from Spain and Turkey formed discrete clusters that did not overlap with samples from other locations (Figure A22.C in Appendix S1). Plots of the first two PCs derived from the DAPC analysis indicated that both Eastern Europe (Russia and Georgia) and the region including Albania, Croatia, Greece, and Serbia constituted discrete clusters, completely separated from other locations (Figure A23.C in Appendix S1).

Regional estimates of average *F*ST were somewhat higher for Eastern (0.11) than for Southern (0.08) or Western Europe (0.07). Serbia had the highest mean pairwise *F*ST (0.19), followed by Russia and Georgia (0.12). Slovenia and Malta had the lowest mean pairwise *F*ST (0.06) (Figures A24.C, A25.C; Table A9 in Appendix S1). The correlation between genetic and geographic distance was minimal: when *F*ST and geographic distance were plotted, the *R* ^2^ was 0.02, and the correlation coefficient (*R* ^2^) was 0.01 when a linear regression model fitted the genetic and geographic distance (Figure A26.C in Appendix S1). There was no evidence for isolation by distance (IBD), as the Mantel test observation was slightly negative (−0.10; *p* value = 0.75; Figure A27.C in Appendix S1).

## 4 Discussion

While population genomic data are crucial to our understanding of biological invasions (North et al. 2021), genomic resources are lacking for many invasive species, and full‐genome sequencing may not always be feasible (Matheson and McGaughran, 2022). SNPs are widely recognized as cost‐effective tools for characterizing genetic diversity and population structure, and they have come to be recognized as valuable tools for assessing invasion dynamics in a variety of taxa, including comb jellies (Pujolar et al. 2022), fire ants (Lenancker et al. 2022), rodents (Oh et al. 2023), and other invasive mosquito species (Evans et al. 2015; Gómez‐Palacio et al. 2024). Specifically, they can be useful for linking invasions to their source population (Resh et al. 2021; Sjodin et al. 2021; Puckett et al. 2016), as well as for informing population management strategies (Sjodin et al. 2019) and revising eradication programs (Sjodin et al. 2020). We used a SNP chip to characterize the genetic structure of *Ae. albopictus* across Europe and the native range to better understand the origin and spread of this invasive mosquito. This study marks the first time a SNP chip has been used to characterize the genetic structure of *Ae. albopictus* outside of its native range. Our findings support the notion of a complex history of multiple invasions from a number of different origins in both the native range (Asia) and previously invaded areas of Europe and the United States (Kotsakiozi et al. 2017; Vavassori et al. 2022). In addition, the Aealbo SNP chip revealed fine‐scale genetic structure in *Ae. albopictus* populations across Europe, not reported in previous studies using less sensitive genetic markers (i.e., microsatellites, mitochondrial DNA) or in our own analyses that utilized only microsatellites.

### 4.1 Genetic Structure in the Native Range

To assess potential sources of invasions into Europe, we first needed to validate the patterns of population structure found in the native range. We identified the same major patterns in the native range that were found by previous analyses using data from the Aealbo SNP chip (Cosme et al. 2024): in both studies, PCA indicated that south and southeast Asian samples generally grouped together, while east Asian samples formed a separate group (Figure 2C). Previous structure analyses found support for five ancestral clusters in Asia (Cosme et al. 2024). Specifically, one cluster was observed primarily in Japan, with some admixture in China. A second cluster was found in Taiwan and southern Japan (Okinawa), with admixture in parts of China. A third cluster was exclusive to Indonesia and some individuals in Nepal. A fourth cluster was found primarily in Quy Nhon City, Vietnam. The fifth cluster was the most widely distributed and constituted the primary cluster in most of south and southeast Asia, as well as parts of China (particularly Yunnan) (Cosme et al. 2024).

Our analyses, which combined data from the native range with European and American populations, found more than five Asian clusters. Specifically, eight of the 20 clusters in the ancestry matrix best supported by our algorithms (shown in Figure 2A) are represented among native range populations. The primary difference from the previous analysis, which examined Asian populations independently, is that some populations previously represented primarily by a single cluster were split into two or more groups. For example, the northern Japanese cluster was divided into at least two clusters (light yellow and gold), and the single cluster that previously represented Okinawa and Taiwan was split into two clusters (orange‐red and dark green). The remaining clustering pattern is very similar to previous findings, in which most of south and southeast Asia cluster together (cyan), and distinct clusters are observed in Indonesia (along with some individuals in Nepal) and Quy Nhon City, Vietnam (Cosme et al. 2024).

When we plotted the ancestry matrices for K = 6 and K = 7 for the global dataset, we observed four of the five clusters previously identified in the native range by the Aeablo SNP chip (Cosme et al. 2024). The main differences were that, in our results, Quy Nhon City, Vietnam clusters with the majority of south and southeast Asia (blue) rather than forming a distinct cluster, and the cluster that primarily constituted Taiwan and Okinawa in the previous analysis showed more admixture (Figure 2C and Figure A9 in Appendix S1).

### 4.2 Origins of Invasive Populations and Genetic Structure Within Europe

In evaluating the origins and patterns of population genetic structure, we focus on European regions whose history of invasion and spread was somewhat unresolved based on previous studies and for which we have a substantial geographic representation in our sample set: Italy, Greece, and Albania; the region around southern Russia; and the Iberian Peninsula. While our analyses confirmed some of the patterns suggested by earlier studies, they also revealed novel findings.

*Aedes albopictus* was first observed in northern Italy in 1990 (Dalla Pozza and Majori 1992; Sabatini et al. 1990), and an invasive population in the United States (originating primarily from Japan) was believed to be the source of this introduction (Urbanelli et al. 2000; Manni et al. 2017). However, various studies have found high levels of genetic diversity in Italy, suggesting that multiple, unrelated introductions from different sources likely contributed to the establishment of Italian populations (Shaikevich and Talbalaghi 2013; Manni et al. 2017; Kotsakiozi et al. 2017; Pichler, Kotsakiozi, et al. 2019). Our analyses support the latter scenario. The three locations from northern Italy sampled in 1995 (Brescia, Cesena, and Desenzano) shared a cluster with samples from northern Japan and the United States (yellow cluster in Figure 2B). While this cluster was also observed admixed in samples from western and southern Europe, the three Italian locations with historical samples were differentiated from one another (Figure 3A) and from modern Italian samples (Figures A8.A, A13 in Appendix S1). In contrast to samples collected in the 1990s, modern Italian locations exhibited high levels of admixture. Still, they shared a cluster (orange), which was observed broadly across western and southern Europe, a few locations in eastern Europe (e.g., Alushta, Ukraine) (Figure 2A), China (Hunan and Hainan), and also as admixture in Taiwan, Okinawa, more northern areas of Japan, and the Americas (Figure 2B and Figure A8.A in Appendix S1).

In summary, modern Italian samples showed intermixing of ancestry derived from historic Italian locations, other parts of Europe, and East Asia (Figure 2), suggesting that *Ae. albopictus* became established throughout Italy via a combination of spreading from the initial introductions in the north in the early 1990s and multiple new introductions from both native and other invasive regions. Furthermore, it appears that populations from northern Italy have played an important role in establishing invasive populations in other European countries: mosquitoes throughout Europe (e.g., Portugal, Romania, Slovenia) shared ancestry with individuals sampled in Desenzano in 1995. This is consistent with indications that Italy has been a source, and even an essential stepping‐stone, for *Ae. albopictus* range expansion into more northern locations, such as Switzerland (Vavassori et al. 2022; Wymann et al. 2008).

Mosquitoes in Greece and Albania (and some other populations in the South Balkans, including south Serbia) have repeatedly been observed to be genetically distinct from other parts of Europe (Manni et al. 2017; Sherpa, Blum, Capblancq, et al. 2019; Vavassori et al. 2022; Mercier et al. 2022). *Aedes albopictus* was first identified in Albania in 1979 (Adhami and Murati 1987), but was not observed in Greece until 2003 (Samanidou‐Voyadjoglou et al. 2005). Previous analyses using microsatellites suggested different sources for invasions in these countries, specifically that populations in Albania originated in China and populations in Greece originated in Thailand (Manni et al. 2017). However, our SNP analyses suggested that samples from Greece and Albania are genetically similar (Sherpa, Blum, Capblancq, et al. 2019; Vavassori et al. 2022), and these populations may have been introduced from the United States (Vavassori et al. 2022).

Our data confirm that Albania and Greece are genetically distinct from most other European populations (Figures A7 and A13 in Appendix S1). While *F*ST for these countries was similar to levels estimated for other southern European countries, when we analyzed population structure in our global dataset, Greece and Albania were grouped into their own cluster that also included Croatia (and Serbia, at K = 7). Greece also showed admixture with the largest cluster in the native range, which included most of South and Southeast Asia and parts of China (cyan cluster, Figure 2A), particularly Hunan and Hainan (Figure 2B and Figure A8.B in Appendix S1). When we examined population structure using only European locations, Serbia constituted its own unique cluster (brown), and Greece was also differentiated from Albania and Croatia, though admixture with the Albanian–Croatian cluster was observed (Figure 3A). This suggests that Albania may have contributed, at least in part, to establishing the invasive population in Greece, but additional introductions directly from Asia or via an invasive population in the Americas have also occurred (Figure 2B and Figure A8.B in Appendix S1).

Our analyses of populations from Eastern Europe suggest an invasion history somewhat different from that identified by previous studies. Studies based on diversity in mitochondrial genomes identified two spatially isolated genetic lineages of *Ae. albopictus* in southern Russia: one linked to Mediterranean populations and another similar to those from the United States and Japan (Shaikevich et al. 2023). While some locations in the farthest east of Europe, namely Armenia and Ukraine, showed admixture with clusters also found in the Mediterranean area, most locations in our analyses were comprised of an admixture of two to three genetic clusters not observed anywhere else in Europe or Asia (purple clusters, Figure 2). Regional estimates of average *F*ST were somewhat higher for eastern Europe compared to southern or western Europe (Tables A8 and A9 in Appendix S1), and the farthest eastern European clusters were distinct in multivariate analyses as well, showing no overlap with other samples in PCA or DAPC plots (Figure A8.C in Appendix S1), and remained differentiated from all other regions even when the number of clusters in our structure analyses was reduced to six (Figure A9 in Appendix S1). This indicates that mosquitoes in this region were introduced from somewhere in the native range not covered by our sample collection and have not had substantial introductions from other areas of Europe. Though the four farthest east countries are separated from the rest of Europe by the Black Sea, it is unclear why most sampling sites there show so little admixture with any other localities. Samples from Turkey (Hopa) likewise showed no admixture with these purple clusters, despite being very geographically close to Georgia and southern Russia (Figures 1 and 3). Another study that utilized mitochondrial genomes found that southern Russia was characterized by strong differentiation and highly restricted gene flow between local populations, and suggested either a single introduction or multiple transfers from the same source population (Konorov et al. 2021). Our SNP data did not confirm this, as locations in southern Russia were genetically similar to one another and showed evidence of extensive admixture among the same three purple clusters throughout the region (Figure A11 in Appendix S1).

Across the Iberian Peninsula, previous work using mtDNA and rDNA generally identified little genetic structure, likely stemming from human‐mediated gene flow and multiple ongoing introductions (Zé‐Zé et al. 2020; Lucati et al. 2022). However, other analyses found that northern Spain and the Balearic Islands were somewhat differentiated from southern Spain and the rest of Europe (Artigas et al. 2021; Sherpa, Blum, Capblancq, et al. 2019). The initial introduction of *Ae. albopictus* to Spain was first detected in 2004 near Barcelona (Aranda et al. 2006), and the species is believed to have spread along the Spanish Mediterranean coast and inland from there (Collantes et al. 2015). While previous findings suggest that Barcelona was the largest source of inter‐province transfers of mosquitoes in Spain (Eritja et al. 2017), our analyses do not support this scenario. We found that mosquitoes in Barcelona formed a distinct cluster (pink) that was not shared with other locations in Spain. However, this cluster was also observed in Turkey and admixed throughout other southern European countries (e.g., Italy and Slovenia; Figure 3A). On the other hand, some mosquitoes from western Spain (Badajoz), eastern Spain (Catarroja), and the Balearic islands (Magaluf) all shared ancestry (yellow in Figures 2A, 3A), indicating a common source from either Japan or the United States for mosquitoes in these geographically separated areas. While Portuguese samples showed some admixture with this cluster, they were more genetically similar to samples from France and other southern European countries. Interestingly, there was less admixture within Spanish samples compared to other European locations, though two locations contained individuals from two separate clusters: one cluster unique to Spain (blue) and the other shared with some individuals from Aliaga, Turkey (red, Figure 3A).

Overall, our findings are consistent with the hypothesis that older established invasive populations (Albania, Italy) seem to be derived from populations in the native range (e.g., Japan) or other early‐established invasive populations (United States), while more recently established populations share ancestry with multiple sources, including populations already existing in Europe.

### 4.3 Factors Influencing Population Differentiation and Local Adaptation

We did not find a significant correlation between genetic and geographical distances among European samples. This lack of correlation is consistent with the results of most previous studies (Manni et al. 2017; Vavassori et al. 2022), including those that examined IBD in the native range (Cosme et al. 2024), and suggests that factors other than distance, such as human‐facilitated transportation and ecological factors, are more important in influencing the genetic structure of *Ae. albopictus* in Europe. Population bottlenecks during introduction can also substantially influence genetic variability (Sherpa et al. 2020). We did not calculate diversity estimates in this study due to limitations stemming from ascertainment bias of our SNP chip. However, previous studies found no statistically significant differences in genetic diversity levels between native and invasive populations, including populations in Europe, and these findings were consistent across various methods (Kotsakiozi et al. 2017; Vavassori et al. 2022), which indicates that bottlenecks are unlikely to contribute to the major patterns in genetic structure that we observed. Studies have shown that introduced *Ae. albopictus* mosquitoes spread through a combination of natural and human‐aided dispersal, and the degree of dispersal may be affected by the landscape (Sherpa et al. 2020). These factors could help to explain why we see different levels of genetic structuring among some European regions more than others. For example, we found that most central European populations showed high degrees of admixture, but the most eastern European populations (i.e., Armenia, Georgia, Russia, and Ukraine) were largely distinct from other areas of Europe. This suggests that after *Ae. albopictus* was introduced into the area in 2011 (Shaikevich et al. 2023) from a unique source not represented elsewhere in Europe, the Black Sea acted as a geographic barrier to limit gene flow with other areas. However, at least one population east of the Black Sea, Hopa (Turkey), did not fit this pattern. Instead, it shares ancestry with more Western European populations (e.g., Spain), despite being geographically close to the Georgian and Armenian populations. This underscores the somewhat “chaotic” nature of dispersion identified in previous studies (Manni et al. 2017) and highlights the importance of human‐mediated introductions and transport, in addition to geographic barriers, in shaping patterns of genetic structure. Our sample size per population was relatively small, with typically 12 or fewer individuals from each location genotyped. Future work should include larger numbers of individuals from each population to carry out migration analysis that looks for first or second‐generation migrants. This would improve our understanding of current gene flow and how human‐mediated transportation of mosquitoes may be impacting the spread and population structure of *Ae. albopictus* throughout their invasive ranges.

While we could not explicitly test for local adaptations using our dataset, there is evidence for thermal adaptation across the wide temperature range occupied by *Ae. albopictus* in its native range (Sherpa et al. 2022), which may have facilitated its spread to temperate regions worldwide (Sherpa, Blum and Després 2019). Our findings are consistent with this idea: the most northern, temperate locations in the native range, Japan and China, were the dominant contributors to populations throughout most of Europe (Figure 2B and Figure A11 in Appendix S1). Previous analyses of *Ae. albopictus* in southern Russia found patterns of selective sweep near genomic regions associated with neural protection, lipid conservation, and cuticle formation during diapause, which could indicate recent selection for cold adaptation within the invasive range (Konorov et al. 2021). Additional systematic sampling, focusing on a range of temperatures and other climatic variables across the European continent, will be essential for determining the extent and specifics of local adaptation to cold and other ecological factors that mosquitoes have undergone as they spread to new, more temperate areas. The availability of a reliable microarray, the Aealbo SNP chip (Cosme et al. 2024), can facilitate the expansion of sampling locations and allow researchers to merge existing data sets with new ones to develop a more thorough and nuanced understanding of *Ae. albopictus* range expansion and local adaptation.

### 4.4 Comparisons of Microsatellite and SNP Datasets

Our SNP markers detected more fine‐scale population structure than the microsatellites, even when the exact overlapping locations were examined with both sets of markers. Specifically, microsatellite data suggested that only three distinct genetic clusters were present in Europe, while SNP data indicated that the best‐supported number of clusters was ten (Figure 5 and Figure A20 in Appendix S1). This suggests that the microsatellite markers were unable to detect genetic structure at the same resolution as the SNPs. Additionally, the three clusters that the microsatellites identified differed from those found when we plotted K = 3 for the SNP data. For example, microsatellite data failed to identify the farthest eastern European locations (Russia and Georgia) as a unique cluster, which was a consistent finding from all the SNP datasets (Figures 2, 3, 5; Figures A9–A11 and A20, A21 in Appendix S1).

Multivariate analyses (PCA and DAPC) also showed less discrimination among populations when using microsatellitesssssss compared to SNP data. For the microsatellite dataset, most samples from all three European regions clustered tightly together, with only France and some individuals from Italy and Türkiye (PCA), and Portugal (DAPC) being differentiated from the rest of Europe. In contrast, in the SNP dataset, the farthest eastern Europe (Russia and Georgia) and some individuals from Spain and Turkey (PCA) as well as Albania, Croatia, Greece, and Serbia (DAPC) formed discrete clusters that did not overlap with samples from other locations (Figures A22 and A23 in Appendix S1).

In both the microsatellites and SNPs datasets, the correlation between genetic and geographic distance was very small (Figure A26 in Appendix S1). Similarly, there was no substantial evidence for isolation by distance (IBD) among the overlapping locations for either the microsatellite or SNP datasets (Mantel test observations were slightly negative for both; Figure A27 in Appendix S1). Overall, these results indicate that using genome‐wide markers, such as SNPs, provides not only finer resolution but also identifies genetic structuring undescribed by microsatellite loci.

## 5 Conclusion

Our results are consistent with previous studies that provide evidence for multiple independent invasions to Europe from both the native region and other areas of the invasive range. Our analyses also confirmed admixture among mosquitoes from different genetic ancestries in many European locations. This highlights the complexity of their spread and the need for comprehensive genetic tools to understand it fully, particularly as genetic mixture can alter spatial expansion and accelerate the spread of invasive species (Wagner et al. 2017). A more extensive set of informative genetic markers across the entire genome was found to be essential to resolve outstanding questions about population structure for invasive populations of *Ae. albopictus* in Europe that have only recently been established and may still be experiencing on‐going introductions (Goubert et al. 2016). Our study underscores the importance of using appropriately sensitive genetic markers when working with invasive populations, and highlights the potential for using SNP chip data to understand and manage other invasive species. Unlike previous studies of *Ae. albopictus*, which used SNPs obtained from ddRAD sequencing (Kotsakiozi et al. 2017), the SNP chips provide a standardized panel of SNPs that can be utilized in future studies to integrate new populations with results from existing datasets. The findings from this study can serve as a baseline for association studies that examine the genetic basis of key traits such as diapause, cold tolerance, arbovirus transmission, insecticide resistance, and other complex traits. Our work can also assist in predicting this species' establishment and spread into other invasive locations, with implications for understanding local adaptation, the dynamics of disease transmission, and strategies for controlling this invasive disease vector throughout Europe and the rest of its expanding range. By providing a deeper understanding of *Ae. albopictus* genetic diversity and movement patterns, our research contributes to efforts to mitigate the public health impacts of this globally significant mosquito species.

## Author Contributions

**Margaret K. Corley:** conceptualization (supporting), data curation (lead), formal analysis (lead), investigation (lead), methodology (equal), supervision (supporting), writing – original draft (lead), writing – review and editing (lead). **Luciano Veiga Cosme:** conceptualization (supporting), methodology (equal), supervision (supporting), writing – review and editing (supporting). **Peter A. Armbruster:** funding acquisition (equal), resources (equal), writing – review and editing (supporting). **Nigel Beebe:** resources (equal), writing – review and editing (supporting). **Anna Bega:** resources (equal), writing – review and editing (supporting). **Sebastien Boyer:** resources (equal), writing – review and editing (supporting). **Beniamino Caputo:** resources (equal), writing – review and editing (supporting). **Chun‐Hong Chen:** resources (equal), writing – review and editing (supporting). **Jacob E. Crawford:** resources (equal), writing – review and editing (supporting). **Alessandra della Torre:** resources (equal), writing – review and editing (supporting). **Roger Eritja:** resources (equal), writing – review and editing (supporting). **Michael C. Fontaine:** resources (equal), writing – review and editing (supporting). **Richard J. Gill:** resources (equal), writing – review and editing (supporting). **Trang Huynh:** resources (equal), writing – review and editing (supporting). **Perparim Kadriaj:** resources (equal), writing – review and editing (supporting). **Kevin Maringer:** resources (equal), writing – review and editing (supporting). **Ademir Jesus Martins:** resources (equal), writing – review and editing (supporting). **Andrew Maynard:** resources (equal), writing – review and editing (supporting). **Shomen Mukherjee:** resources (equal), writing – review and editing (supporting). **Leonard E. Munstermann:** resources (equal), writing – review and editing (supporting). **Verena Pichler:** resources (equal), writing – review and editing (supporting). **Maria Sharakhova:** resources (equal), writing – review and editing (supporting). **Sinnathamby Noble Surendran:** resources (equal), writing – review and editing (supporting). **Sandra Urbanelli:** resources (equal), writing – review and editing (supporting). **Enkelejda Velo:** resources (equal), writing – review and editing (supporting). **Isra Wahid:** resources (equal), writing – review and editing (supporting). **Muhammet Mustafa Akiner:** resources (equal), writing – review and editing (supporting). **Georgios Balatsos:** resources (equal), writing – review and editing (supporting). **Gilles Besnard:** resources (equal), writing – review and editing (supporting). **Maria Louise Borg:** resources (equal), writing – review and editing (supporting). **Daniel Bravo‐Barriga:** resources (equal), writing – review and editing (supporting). **Rubén Bueno Marí:** resources (equal), writing – review and editing (supporting). **Francisco Collantes:** resources (equal), writing – review and editing (supporting). **Cintia Horvath:** resources (equal), writing – review and editing (supporting). **Mihaela Kavran:** resources (equal), writing – review and editing (supporting). **Raquel Medialdea‐Carrera:** resources (equal), writing – review and editing (supporting). **Tanya Melillo:** resources (equal), writing – review and editing (supporting). **Antonios Michaelakis:** resources (equal), writing – review and editing (supporting). **Ognyan Mikov:** resources (equal), writing – review and editing (supporting). **Arianna Puggioli:** resources (equal), writing – review and editing (supporting). **Elton Rogozi:** resources (equal), writing – review and editing (supporting). **Francis Schaffner:** resources (equal), writing – review and editing (supporting). **Kayleigh Hackett:** investigation (supporting), writing – review and editing (supporting). **Thomas Johnson:** methodology (supporting), resources (equal), validation (supporting), writing – review and editing (supporting). **Tina Wu:** investigation (supporting), writing – review and editing (supporting). **João Pinto:** conceptualization (supporting), data curation (supporting), resources (equal), writing – original draft (supporting), writing – review and editing (equal). **Vera Valadas:** formal analysis (supporting), investigation (supporting), resources (equal), writing – review and editing (supporting). **Adalgisa Caccone:** conceptualization (lead), funding acquisition (equal), project administration (lead), resources (equal), supervision (lead), writing – original draft (supporting), writing – review and editing (equal).

## Ethics Statement

This research complies with all applicable laws on sampling from natural populations. All sample handling and shipping were in accordance with Yale Environmental and Health Safety protocols.

## Conflicts of Interest

The authors declare no conflicts of interest.

## Supporting information


Appendix S1.

Data S1.

Data S2.

File S15.

File S3.–S5.

## Data Availability

This research involved international partnerships with scientists across many countries. All collaborators providing genetic samples were invited to be co‐authors, and the data and results have been made available in public databases. The codes describing the step‐by‐step of all analyses are available at https://rpubs.com/margaret_corley, and an overview of the analyses contained in each RMarkdown file is provided in Table A1 in Appendix S1. The raw data files required to reproduce the analyses are available in Zenodo (native range: https://zenodo.org/records/10048029, Europe & Americas: https://doi.org/10.5281/zenodo.13760661).

## References

[ece371009-bib-0001] Adhami, J. , and N. Murati . 1987. “The Presence of the Mosquito *Aedes albopictus* in Albania.” Revista Mjekësore 1: 13–16.

[ece371009-bib-0002] Alexander, D. H. , and K. Lange . 2011. “Enhancements to the ADMIXTURE Algorithm for Individual Ancestry Estimation.” BMC Bioinformatics 12: 246. 21682921 10.1186/1471-2105-12-246 PMC3146885

[ece371009-bib-0003] Apostol, B. L. , W. C. Black , P. Reiter , and B. R. Miller . 1996. “Population Genetics With RAPD‐PCR Markers: The Breeding Structure of *Aedes aegypti* in Puerto Rico.” Heredity 76: 325–334. 8626220 10.1038/hdy.1996.50

[ece371009-bib-0004] Aranda, C. , R. Eritja , and D. Roiz . 2006. “First Record and Establishment of the Mosquito *Aedes albopictus* in Spain.” Medical and Veterinary Entomology 20: 150–152. 16608499 10.1111/j.1365-2915.2006.00605.x

[ece371009-bib-0005] Artigas, P. , M. Reguera‐Gomez , M. A. Valero , et al. 2021. “ *Aedes albopictus* Diversity and Relationships in South‐Western Europe and Brazil by rDNA/mtDNA and Phenotypic Analyses: ITS‐2, a Useful Marker for Spread Studies.” Parasites & Vectors 14, no. 1: 333. 10.1186/s13071-021-04829-9. 34174940 PMC8235640

[ece371009-bib-0006] Baldacchino, F. , B. Caputo , F. Chandre , et al. 2015. “Control Methods Against Invasive *Aedes* Mosquitoes in Europe: A Review.” Pest Management Science 71, no. 11: 1471–1485. 10.1002/ps.4044. 26037532

[ece371009-bib-0007] Battaglia, V. , P. Gabrieli , S. Brandini , et al. 2016. “The Worldwide Spread of the Tiger Mosquito as Revealed by Mitogenome Haplogroup Diversity.” Frontiers in Genetics 7: 208. 10.3389/fgene.2016.00208. 27933090 PMC5120106

[ece371009-bib-0008] Brady, O. J. , N. Golding , D. M. Pigott , et al. 2014. “Global Temperature Constraints on *Aedes aegypti* and *Ae. albopictus* Persistence and Competence for Dengue Virus Transmission.” Parasites & Vectors 7, no. 1: 1–17. 10.1186/1756-3305-7-338. 25052008 PMC4148136

[ece371009-bib-0009] Browett, S. S. , D. B. O'Meara , and A. D. McDevitt . 2020. “Genetic Tools in the Management of Invasive Mammals: Recent Trends and Future Perspectives.” Mammal Review 50: 200–210.

[ece371009-bib-0010] Bueno‐Marí, R. , and R. Jiménez‐Peydró . 2015. “First Observations of Homodynamic Populations of *Aedes albopictus* (Skuse) in Southwest Europe.” Journal of Vector Borne Diseases 52: 175. 26119552

[ece371009-bib-0011] Caputo, B. , M. Manica , A. D'Alessandro , et al. 2016. “Assessment of the Effectiveness of a Seasonal‐Long Insecticide‐Based Control Strategy Against *Aedes albopictus* Nuisance in an Urban Area.” PLoS Neglected Tropical Diseases 10: e0004463. 26937958 10.1371/journal.pntd.0004463 PMC4777573

[ece371009-bib-0012] Chang, C. C. , C. C. Chow , L. C. A. M. Tellier , S. Vattikuti , S. M. Purcell , and J. J. Lee . 2015. “Second‐Generation PLINK: Rising to the Challenge of Larger and Richer Datasets.” GigaScience 4, no. 1: 7. 10.1186/s13742-015-0047-8. 25722852 PMC4342193

[ece371009-bib-0013] Chen, Z. L. , J. M. Meng , Y. Cao , et al. 2019. “A High‐Speed Search Engine pLink 2 With Systematic Evaluation for Proteome‐Scale Identification of Cross‐Linked Peptides.” Nature Communications 10: 3404. 10.1038/s41467-019-11337-z PMC6667459 31363125

[ece371009-bib-0014] Collantes, F. , S. Delacour , P. M. Alarcón‐Elbal , et al. 2015. “Review of Ten‐Years Presence of *Aedes albopictus* in Spain 2004–2014: Known Distribution and Public Health Concerns.” Parasites & Vectors 8: 655. 26694818 10.1186/s13071-015-1262-y PMC4688962

[ece371009-bib-0015] Collantes, F. , J. A. Delgado , P. M. Alarcón‐Elbal , S. Delacour , and J. Lucientes . 2014. “First Confirmed Outdoor Winter Reproductive Activity of Asian Tiger Mosquito (*Aedes albopictus*) in Europe.” Anales de Biologia 36: 71–76.

[ece371009-bib-0016] Cosme, L. V. , M. Corley , T. Johnson , et al. 2024. “A Genotyping Array for the Globally Invasive Vector Mosquito, *Aedes albopictus* .” Parasites & Vectors 17: 106. 10.1186/s13071-024-06158-z. 38439081 PMC10910840

[ece371009-bib-0017] Dalla Pozza, G. , and G. Majori . 1992. “First Record of *Aedes albopictus* Establishment in Italy.” Journal of the American Mosquito Control Association 8: 318–320. 1402871

[ece371009-bib-0018] de Lamballerie, X. , E. Leroy , R. N. Charrel , K. Ttsetsarkin , S. Higgs , and E. A. Gould . 2008. “Chikungunya Virus Adapts to Tiger Mosquito via Evolutionary Convergence: A Sign of Things to Come?” Virology Journal 5: 1–4. 18304328 10.1186/1743-422X-5-33 PMC2266737

[ece371009-bib-0019] Del Lesto, I. , C. De Liberato , R. Casini , A. Magliano , A. Ermenegildi , and F. Romiti . 2022. “Is Asian Tiger Mosquito ( *Aedes albopictus* ) Going to Become Homodynamic in Southern Europe in the Next Decades due to Climate Change?” Royal Society Open Science 9: 220967. 36533199 10.1098/rsos.220967 PMC9748500

[ece371009-bib-0020] Dray, S. , and A.‐B. Dufour . 2007. “The ade4 Package: Implementing the Duality Diagram for Ecologists.” Journal of Statistical Software 22: 1–20.

[ece371009-bib-0021] Eritja, R. , J. R. B. Palmer , D. Roiz , I. Sanpera‐Calbet , and F. Bartumeus . 2017. “Direct Evidence of Adult *Aedes albopictus* Dispersal by Car.” Scientific Reports 7: 14399. 29070818 10.1038/s41598-017-12652-5 PMC5656642

[ece371009-bib-0022] Estoup, A. , V. Ravigné , R. Hufbauer , R. Vitalis , M. Gautier , and B. Facon . 2016. “Is There a Genetic Paradox of Biological Invasion?” Annual Review of Ecology, Evolution, and Systematics 47: 51–72.

[ece371009-bib-0023] European Centre for Disease Prevention and Control . 2023. “*Aedes albopictus* – Current Known Distribution: February 2023.” Accessed January 17, 2024. https://www.ecdc.europa.eu/en/publications‐data/aedes‐albopictus‐current‐known‐distribution‐february‐2023.

[ece371009-bib-0024] Evanno, G. , S. Regnaut , and J. Goudet . 2005. “Detecting the Number of Clusters of Individuals Using the Software Structure: A Simulation Study.” Molecular Ecology 14: 2611–2620. 15969739 10.1111/j.1365-294X.2005.02553.x

[ece371009-bib-0025] Evans, B. R. , A. Gloria‐Soria , L. Hou , et al. 2015. “A Multipurpose, High‐Throughput Single‐Nucleotide Polymorphism Chip for the Dengue and Yellow Fever Mosquito, *Aedes aegypti* .” G3: Genes, Genomes, Genetics 5: 711–718. 25721127 10.1534/g3.114.016196 PMC4426360

[ece371009-bib-0026] Falush, D. , M. Stephens , and J. K. Pritchard . 2003. “Inference of Population Structure Using Multilocus Genotype Data: Linked Loci and Correlated Allele Frequencies.” Genetics 164: 1567–1587. 12930761 10.1093/genetics/164.4.1567 PMC1462648

[ece371009-bib-0027] Falush, D. , M. Stephens , and J. K. Pritchard . 2007. “Inference of Population Structure Using Multilocus Genotype Data: Dominant Markers and Null Alleles.” Molecular Ecology Notes 7: 574–578. 18784791 10.1111/j.1471-8286.2007.01758.x PMC1974779

[ece371009-bib-0028] Farajollahi, A. , S. P. Healy , I. Unlu , R. Gaugler , and D. M. Fonseca . 2012. “Effectiveness of Ultra‐Low Volume Nighttime Applications of an Adulticide Against Diurnal *Aedes albopictus*, a Critical Vector of Dengue and Chikungunya Viruses.” PLoS One 7: e49181. 23145115 10.1371/journal.pone.0049181 PMC3493500

[ece371009-bib-0029] Frichot, E. , and O. François . 2015. “LEA: An R Package for Landscape and Ecological Association Studies.” Methods in Ecology and Evolution 6: 925–929.

[ece371009-bib-0030] Giron, S. , F. Franke , A. Decoppet , et al. 2019. “Vector‐Borne Transmission of Zika Virus in Europe, Southern France, August 2019.” Eurosurveillance 24, no. 45: 1900655. 10.2807/1560-7917.ES.2019.24.45.1900655. 31718742 PMC6852313

[ece371009-bib-0031] Gojković, N. , J. Ludoški , B. Krtinić , and V. Milankov . 2019. “The First Molecular and Phenotypic Characterization of the Invasive Population of *Aedes albopictus* (Diptera: Culicidae) From the Central Balkans.” Journal of Medical Entomology 56: 1433–1440. 31100120 10.1093/jme/tjz064

[ece371009-bib-0032] Gómez‐Palacio, A. , G. Morinaga , P. E. Turner , et al. 2024. “Robustness in Population‐Structure and Demographic‐Inference Results Derived From the *Aedes aegypti* Genotyping Chip and Whole‐Genome Sequencing Data.” G3: Genes, Genomes, Genetics 14: jkae082. 38626295 10.1093/g3journal/jkae082 PMC11152066

[ece371009-bib-0033] Goubert, C. , H. Henri , G. Minard , et al. 2017. “High‐Throughput Sequencing of Transposable Element Insertions Suggests Adaptive Evolution of the Invasive Asian Tiger Mosquito Towards Temperate Environments.” Molecular Ecology 26: 3968–3981. 28517033 10.1111/mec.14184

[ece371009-bib-0034] Goubert, C. , G. Minard , C. Vieira , and M. Boulesteix . 2016. “Population Genetics of the Asian Tiger Mosquito *Aedes albopictus*, an Invasive Vector of Human Diseases.” Heredity 117: 125–134. 27273325 10.1038/hdy.2016.35 PMC4981682

[ece371009-bib-0035] Goudet, J. 2005. “Hierfstat, a Package for R to Compute and Test Hierarchical F‐Statistics.” Molecular Ecology Notes 5, no. 184: 184–186. 10.1111/j.1471-8286.2004.00828.x.

[ece371009-bib-0036] Gratz, N. G. 2004. “Critical Review of the Vector Status of *Aedes albopictus* .” Medical and Veterinary Entomology 18: 215–227. 15347388 10.1111/j.0269-283X.2004.00513.x

[ece371009-bib-0037] Hubisz, M. J. , D. Falush , M. Stephens , and J. K. Pritchard . 2009. “Inferring Weak Population Structure With the Assistance of Sample Group Information.” Molecular Ecology Resources 9: 1322–1332. 21564903 10.1111/j.1755-0998.2009.02591.x PMC3518025

[ece371009-bib-0038] Hulce, D. , X. Li , T. Snyder‐Leiby , and C. J. Liu . 2011. “GeneMarker® Genotyping Software: Tools to Increase the Statistical Power of DNA Fragment Analysis.” Journal of Biomolecular Techniques 22: S35–S36.

[ece371009-bib-0039] Jombart, T. 2008. “Adegenet: A R Package for the Multivariate Analysis of Genetic Markers.” Bioinformatics 24: 1403–1405. 18397895 10.1093/bioinformatics/btn129

[ece371009-bib-0040] Jombart, T. , S. Devillard , and F. Balloux . 2010. “Discriminant Analysis of Principal Components: A New Method for the Analysis of Genetically Structured Populations.” BMC Genetics 11, no. 94: 94. 10.1186/1471-2156-11-94. 20950446 PMC2973851

[ece371009-bib-0041] Konorov, E. A. , V. Yurchenko , I. Patraman , A. Lukashev , and N. Oyun . 2021. “The Effects of Genetic Drift and Genomic Selection on Differentiation and Local Adaptation of the Introduced Populations of *Aedes albopictus* in Southern Russia.” PeerJ 9: e11776. 34327056 10.7717/peerj.11776 PMC8308624

[ece371009-bib-0042] Kopelman, N. M. , J. Mayzel , M. Jakobsson , N. A. Rosenberg , and I. Mayrose . 2015. “Clumpak: A Program for Identifying Clustering Modes and Packaging Population Structure Inferences Across K.” Molecular Ecology Resources 15: 1179–1191. 25684545 10.1111/1755-0998.12387 PMC4534335

[ece371009-bib-0043] Kotsakiozi, P. , J. B. Richardson , V. Pichler , et al. 2017. “Population Genomics of the Asian Tiger Mosquito, *Aedes albopictus* : Insights Into the Recent Worldwide Invasion.” Ecology and Evolution 7: 10143–10157. 29238544 10.1002/ece3.3514 PMC5723592

[ece371009-bib-0044] Kraemer, M. U. , M. E. Sinka , K. A. Duda , et al. 2015. “The Global Distribution of the Arbovirus Vectors *Aedes Aegypti* and *Ae. albopictus* .” eLife 4: e08347. 10.7554/eLife.08347. 26126267 PMC4493616

[ece371009-bib-0045] Kraemer, M. U. G. , R. C. Reiner Jr. , O. J. Brady , et al. 2019. “Past and Future Spread of the Arbovirus Vectors *Aedes aegypti* and *Aedes albopictus* .” Nature Microbiology 4, no. 5: 854–863. 10.1038/s41564-019-0376-y. PMC6522366 30833735

[ece371009-bib-0046] Legendre, P. , and L. Legendre . 2012. Numerical Ecology. Elsevier.

[ece371009-bib-0047] Lenancker, P. , T. Walsh , S. Metcalfe , et al. 2022. “Genome‐Wide SNPs Reveal the Social Structure and Invasion Pathways of the Invasive Tropical Fire Ant (*Solenopsis geminata*).” Preprint, Accessed July 24, 2024. https://www.biorxiv.org/content/10.1101/2022.07.20.500883v1.

[ece371009-bib-0048] Lucati, F. , S. Delacour , J. R. B. Palmer , et al. 2022. “Multiple Invasions, Wolbachia and Human‐Aided Transport Drive the Genetic Variability of *Aedes albopictus* in the Iberian Peninsula.” Scientific Reports 12: 20682. 36450768 10.1038/s41598-022-24963-3 PMC9712423

[ece371009-bib-0049] Lühken, R. , A. Heitmann , S. Jansen , et al. 2020. “Microsatellite Typing of *Aedes albopictus* (Diptera: Culicidae) Populations From Germany Suggests Regular Introductions.” Infection, Genetics and Evolution 81: 104237. 10.1016/j.meegid.2020.104237 32045712

[ece371009-bib-0050] Manni, M. , L. M. Gomulski , N. Aketarawong , et al. 2015. “Molecular Markers for Analyses of Intraspecific Genetic Diversity in the Asian Tiger Mosquito, *Aedes albopictus* .” Parasites & Vectors 8: 188. 10.1186/s13071-015-0794-5. 25890257 PMC4404008

[ece371009-bib-0051] Manni, M. , C. R. Guglielmino , F. Scolari , et al. 2017. “Genetic Evidence for a Worldwide Chaotic Dispersion Pattern of the Arbovirus Vector, *Aedes albopictus* .” PLoS Neglected Tropical Diseases 11, no. 1: e0005332. 10.1371/journal.pntd.0005332. 28135274 PMC5300280

[ece371009-bib-0052] Matheson, P. , and A. McGaughran . 2022. “Genomic Data Is Missing for Many Highly Invasive Species, Restricting Our Preparedness for Escalating Incursion Rates.” Scientific Reports 12: 13987. 35977991 10.1038/s41598-022-17937-y PMC9385848

[ece371009-bib-0053] Medley, K. A. 2010. “Niche Shifts During the Global Invasion of the Asian Tiger Mosquito, *Aedes albopictus*s kuse (Culicidae), Revealed by Reciprocal Distribution Models.” Global Ecology and Biogeography 19: 122–133.

[ece371009-bib-0054] Mercier, A. , T. Obadia , D. Carraretto , et al. 2022. “Impact of Temperature on Dengue and Chikungunya Transmission by the Mosquito *Aedes albopictus* .” Scientific Reports 12: 6973. 35484193 10.1038/s41598-022-10977-4 PMC9051100

[ece371009-bib-0055] Minard, G. , F. H. Tran , V. T. Van , et al. 2015. “French Invasive Asian Tiger Mosquito Populations Harbor Reduced Bacterial Microbiota and Genetic Diversity Compared to Vietnamese Autochthonous Relatives.” Frontiers in Microbiology 6: 970. 10.3389/fmicb.2015.00970. 26441903 PMC4585046

[ece371009-bib-0056] Mousson, L. , C. Dauga , T. Garrigues , F. Schaffner , M. Vazeille , and A. B. Failloux . 2005. “Phylogeography of *Aedes (Stegomyia) aegypti* (L.) and *Aedes (Stegomyia) albopictus* (Skuse) (Diptera: Culicidae) Based on Mitochondrial DNA Variations.” Genetics Research 86: 1–11. 10.1017/S0016672305007627 16181519

[ece371009-bib-0057] North, H. L. , A. McGaughran , and C. D. Jiggins . 2021. “Insights Into Invasive Species From Whole‐Genome Resequencing.” Molecular Ecology 30: 6289–6308. 34041794 10.1111/mec.15999

[ece371009-bib-0058] Oh, K. P. , N. V. de Weyer , W. A. Ruscoe , S. Henry , and P. R. Brown . 2023. “From Chip to SNP: Rapid Development and Evaluation of a Targeted Capture Genotyping‐By‐Sequencing Approach to Support Research and Management of a Plaguing Rodent.” PLoS One 18, no. 8: e0288701. 10.1371/journal.pone.0288701. 37590245 PMC10434965

[ece371009-bib-0059] Oksanen, J. , F. G. Blanchet , R. Kindt , et al. 2022. “vegan: Community Ecology Package.” Deposited 2022.

[ece371009-bib-0060] Patil, I. 2021. “Visualizations With Statistical Details: The “Ggstatsplot” Approach.” Journal of Open Source Software 6: 3167.

[ece371009-bib-0061] Paupy, C. , H. Delatte , L. Bagny , V. Corbel , and D. Fontenille . 2009. “ *Aedes albopictus*, an Arbovirus Vector: From the Darkness to the Light.” Microbes and Infection 11: 1177–1185. 19450706 10.1016/j.micinf.2009.05.005

[ece371009-bib-0062] Pembleton, L. W. , N. O. Cogan , and J. W. Forster . 2013. “StAMPP: An R Package for Calculation of Genetic Differentiation and Structure of Mixed‐Ploidy Level Populations.” Molecular Ecology Resources 13: 946–952. 23738873 10.1111/1755-0998.12129

[ece371009-bib-0063] Pichler, V. , B. Caputo , V. Valadas , et al. 2022. “Geographic Distribution of the V1016G Knockdown Resistance Mutation in *Aedes albopictus* : A Warning Bell for Europe.” Parasites & Vectors 15: 280. 35932088 10.1186/s13071-022-05407-3 PMC9356396

[ece371009-bib-0064] Pichler, V. , P. Kotsakiozi , B. Caputo , P. Serini , A. Caccone , and A. della Torre . 2019. “Complex Interplay of Evolutionary Forces Shaping Population Genomic Structure of Invasive *Aedes albopictus* in Southern Europe.” PLoS Neglected Tropical Diseases 13: e0007554. 31437154 10.1371/journal.pntd.0007554 PMC6705758

[ece371009-bib-0065] Pichler, V. , C. Malandruccolo , P. Serini , et al. 2019. “Phenotypic and Genotypic Pyrethroid Resistance of *Aedes albopictus* , With Focus on the 2017 Chikungunya Outbreak in Italy.” Pest Management Science 75: 2642–2651. 30729706 10.1002/ps.5369

[ece371009-bib-0066] Pritchard, J. K. , M. Stephens , and P. Donnelly . 2000. “Inference of Population Structure Using Multilocus Genotype Data.” Genetics 155: 945–959. 10835412 10.1093/genetics/155.2.945 PMC1461096

[ece371009-bib-0067] Puckett, E. E. , J. Park , M. Combs , et al. 2016. “Global Population Divergence and Admixture of the Brown Rat ( *Rattus norvegicus* ).” Proceedings of the Royal Society B: Biological Sciences 283: 20161762. 10.1098/rspb.2016.1762 PMC5095384 27798305

[ece371009-bib-0068] Pujolar, J. M. , M. T. Limborg , M. Ehrlich , and C. Jaspers . 2022. “High Throughput SNP Chip as Cost Effective New Monitoring Tool for Assessing Invasion Dynamics in the Comb Jelly *Mnemiopsis leidyi* .” Frontiers in Marine Science 9: 1019001.

[ece371009-bib-0069] Raj, A. , M. Stephens , and J. K. Pritchard . 2014. “fastSTRUCTURE: Variational Inference of Population Structure in Large SNP Data Sets.” Genetics 197: 573–589. 24700103 10.1534/genetics.114.164350 PMC4063916

[ece371009-bib-0070] Resh, C. A. , M. P. Galaska , K. C. Benesh , et al. 2021. “Using Genomics to Link Populations of an Invasive Species to Its Potential Sources.” Frontiers in Ecology and Evolution 9: 575599. 10.3389/fevo.2021.575599.

[ece371009-bib-0071] Rezza, G. , L. Nicoletti , R. Angelini , et al. 2007. “Infection With Chikungunya Virus in Italy: An Outbreak in a Temperate Region.” Lancet 370: 1840–1846. 18061059 10.1016/S0140-6736(07)61779-6

[ece371009-bib-0072] Ruiling, Z. , L. Tongkai , M. Dezhen , and Z. Zhong . 2018. “Genetic Characters of the Globally Spread Tiger Mosquito, *Aedes albopictus* (Diptera, Culicidae): Implications From Mitochondrial Gene COI.” Journal of Vector Ecology 43: 89–97. 29757513 10.1111/jvec.12287

[ece371009-bib-0073] Sabatini, A. , V. Raineri , G. Trovato , and M. Coluzzi . 1990. “ *Aedes albopictus* in Italy and Possible Diffusion of the Species Into the Mediterranean Area.” Parassitologia 32: 301–304. 2132441

[ece371009-bib-0074] Samanidou‐Voyadjoglou, A. , E. Patsoula , G. Spanakos , and N. C. Vakalis . 2005. “Confirmation of *Aedes albopictus* (Skuse) (Diptera: Culicidae) in Greece.” European Mosquito Bulletin 9: 10–11.

[ece371009-bib-0075] Schaffner, F. , J. M. Medlock , and W. V. Bortel . 2013. “Public Health Significance of Invasive Mosquitoes in Europe.” Clinical Microbiology and Infection 19: 685–692. 23574618 10.1111/1469-0691.12189

[ece371009-bib-0076] Shaikevich, E. , L. Karan , and M. Fedorova . 2023. “Genetic Structure of *Aedes (Stegomyia) albopictus* Populations in Russia.” Journal of Arthropod‐Borne Diseases 17: 51–62. 37609565 10.18502/jad.v17i1.13201 PMC10440497

[ece371009-bib-0077] Shaikevich, E. , and A. Talbalaghi . 2013. “Molecular Characterization of the Asian Tiger Mosquito *Aedes albopictus* (Skuse) (Diptera: Culicidae) in Northern Italy.” International Scholarly Research Notices 2013: 157426.

[ece371009-bib-0078] Shaikevich, Е. V. , I. V. Patraman , A. S. Bogacheva , V. М. Rakova , O. Р. Zelya , and L. A. Ganushkina . 2018. “Invasive Mosquito Species *Aedes albopictus* and *Aedes aegypti* on the Black Sea Coast of the Caucasus: Genetics (COI, ITS2), Wolbachia and Dirofilaria Infections.” Vavilov Journal of Genetics and Breeding 22: 574–585.

[ece371009-bib-0079] Sherpa, S. , M. G. B. Blum , T. Capblancq , T. Cumer , D. Rioux , and L. Després . 2019. “Unravelling the Invasion History of the Asian Tiger Mosquito in Europe.” Molecular Ecology 28: 2360–2377. 30849200 10.1111/mec.15071

[ece371009-bib-0080] Sherpa, S. , M. G. B. Blum , and L. Després . 2019. “Cold Adaptation in the Asian Tiger Mosquito's Native Range Precedes Its Invasion Success in Temperate Regions.” Evolution 73: 1793–1808. 31313825 10.1111/evo.13801

[ece371009-bib-0081] Sherpa, S. , M. Guéguen , J. Renaud , et al. 2019. “Predicting the Success of an Invader: Niche Shift Versus Niche Conservatism.” Ecology and Evolution 9: 12658–12675. 31788205 10.1002/ece3.5734 PMC6875661

[ece371009-bib-0082] Sherpa, S. , J. Renaud , M. Guéguen , et al. 2020. “Landscape Does Matter: Disentangling Founder Effects From Natural and Human‐Aided Post‐Introduction Dispersal During an Ongoing Biological Invasion.” Journal of Animal Ecology 89: 2027–2042. 32597498 10.1111/1365-2656.13284

[ece371009-bib-0083] Sherpa, S. , D. Rioux , C. Pougnet‐Lagarde , and L. Després . 2018. “Genetic Diversity and Distribution Differ Between Long‐Established and Recently Introduced Populations in the Invasive Mosquito *Aedes albopictus* .” Infection, Genetics and Evolution 58: 145–156. 10.1016/j.meegid.2017.12.018 29275191

[ece371009-bib-0084] Sherpa, S. , J. Tutagata , T. Gaude , et al. 2022. “Genomic Shifts, Phenotypic Clines, and Fitness Costs Associated With Cold Tolerance in the Asian Tiger Mosquito.” Molecular Biology and Evolution 39, no. 5: msac104. 10.1093/molbev/msac104. 35574643 PMC9156037

[ece371009-bib-0085] Sjodin, B. M. F. , R. L. Irvine , A. T. Ford , G. R. Howald , and M. A. Russello . 2019. “ *Rattus* Population Genomics Across the Haida Gwaii Archipelago Provides a Framework for Guiding Invasive Species Management.” Evolutionary Applications 13: 889–904. 10.1111/eva.12907 PMC7232760 32431741

[ece371009-bib-0086] Sjodin, B. M. F. , R. L. Irvine , and M. A. Russello . 2020. “RapidRat: Development, Validation and Application of a Genotyping‐By‐Sequencing Panel for Rapid Biosecurity and Invasive Species Management.” PLoS One 15: e0234694. 32555734 10.1371/journal.pone.0234694 PMC7302687

[ece371009-bib-0087] Sjodin, B. M. F. , E. E. Puckett , R. L. Irvine , J. Munshi‐South , and M. A. Russello . 2021. “Global Origins of Invasive Brown Rats (*Rattus norvegicus*) in the Haida Gwaii Archipelago.” Biological Invasions 23: 611–623.

[ece371009-bib-0088] Sprenger, P. R. D. 1987. “The Used Tire Trade: A Mechanism for the Worldwide Dispersal of Container Breeding Mosquitoes.” Journal of the American Mosquito Control Association 3: 494. 2904963

[ece371009-bib-0089] Swan, T. , T. L. Russell , K. M. Staunton , M. A. Field , S. A. Ritchie , and T. R. Burkot . 2022. “A Literature Review of Dispersal Pathways of *Aedes albopictus* Across Different Spatial Scales: Implications for Vector Surveillance.” Parasites & Vectors 15: 303. 36030291 10.1186/s13071-022-05413-5 PMC9420301

[ece371009-bib-0090] Tancredi, A. , D. Papandrea , M. Marconcini , et al. 2020. “Tracing Temporal and Geographic Distribution of Resistance to Pyrethroids in the Arboviral Vector *Aedes albopictus* .” PLoS Neglected Tropical Diseases 14: e0008350. 32569337 10.1371/journal.pntd.0008350 PMC7332087

[ece371009-bib-0091] Tomasello, D. , and P. Schlagenhauf . 2013. “Chikungunya and Dengue Autochthonous Cases in Europe, 2007–2012.” Travel Medicine and Infectious Disease 11: 274–284. 23962447 10.1016/j.tmaid.2013.07.006

[ece371009-bib-0092] Uller, T. , and R. Leimu . 2011. “Founder Events Predict Changes in Genetic Diversity During Human‐Mediated Range Expansions.” Global Change Biology 17: 3478–3485.

[ece371009-bib-0093] Unlu, I. , A. Farajollahi , D. Strickman , and D. M. Fonseca . 2013. “Crouching Tiger, Hidden Trouble: Urban Sources of *Aedes albopictus* (Diptera: Culicidae) Refractory to Source‐Reduction.” PLoS One 8: e77999. 24167593 10.1371/journal.pone.0077999 PMC3805523

[ece371009-bib-0094] Urbanelli, S. , R. Bellini , M. Carrieri , P. Sallicandro , and G. Celli . 2000. “Population Structure of *Aedes albopictus* (Skuse): The Mosquito Which is Colonizing Mediterranean Countries.” Heredity 84: 331–337. 10762403 10.1046/j.1365-2540.2000.00676.x

[ece371009-bib-0095] Usmani‐Brown, S. , L. Cohnstaedt , and L. E. Munstermann . 2009. “Population Genetics of *Aedes albopictus* (Diptera: Culicidae) Invading Populations, Ssing Mitochondrial Nicotinamide Adenine Dinucleotide Dehydrogenase Subunit 5 Sequences.” Annals of the Entomological Society of America 102: 144–150. 22544973 10.1603/008.102.0116 PMC3337552

[ece371009-bib-0096] Vavassori, L. , A.‐C. Honnen , N. Saarman , A. Caccone , and P. Müller . 2022. “Multiple Introductions and Overwintering Shape the Progressive Invasion of *Aedes albopictus* Beyond the Alps.” Ecology and Evolution 12: e9138. 35903757 10.1002/ece3.9138 PMC9313497

[ece371009-bib-0097] Vega‐Rúa, A. , M. Marconcini , Y. Madec , et al. 2020. “Vector Competence of *Aedes albopictus* Populations for Chikungunya Virus Is Shaped by Their Demographic History.” Communications Biology 3, no. 1: 1–13. 10.1038/s42003-020-1046-6. 32581265 PMC7314749

[ece371009-bib-0098] Wagner, N. K. , B. M. Ochocki , K. M. Crawford , A. Compagnoni , and T. E. X. Miller . 2017. “Genetic Mixture of Multiple Source Populations Accelerates Invasive Range Expansion.” Journal of Animal Ecology 86: 21–34. 27363388 10.1111/1365-2656.12567

[ece371009-bib-0099] Walther, D. , D. E. Scheuch , and H. Kampen . 2017. “The Invasive Asian Tiger Mosquito *Aedes albopictus* (Diptera: Culicidae) in Germany: Local Reproduction and Overwintering.” Acta Tropica 166: 186–192. 27876647 10.1016/j.actatropica.2016.11.024

[ece371009-bib-0100] Wang, J. , A. Caballero , and W. G. Hill . 1998. “The Effect of Linkage Disequilibrium and Deviation From Hardy–Weinberg Proportions on the Changes in Genetic Variance With Bottlenecking.” Heredity 81: 174–186.

[ece371009-bib-0101] Weir, B. S. , and C. C. Cockerham . 1984. “Estimating F‐Statistics for the Analysis of Population Structure.” Evolution 38: 1358–1370. 28563791 10.1111/j.1558-5646.1984.tb05657.x

[ece371009-bib-0102] Wymann, M. N. , E. Flacio , S. Radczuweit , N. Patocchi , and P. Lüthy . 2008. “Asian Tiger Mosquito (*Aedes albopictus*) – A Threat for Switzerland?” Eurosurveillance 13: 3–4. 10.2807/ese.13.10.08058-en 18445441

[ece371009-bib-0103] Zé‐Zé, L. , V. Borges , H. C. Osório , J. Machado , J. P. Gomes , and M. J. Alves . 2020. “Mitogenome Diversity of *Aedes (Stegomyia) albopictus*: Detection of Multiple Introduction Events in Portugal.” PLoS Neglected Tropical Diseases 14: e0008657. 32997656 10.1371/journal.pntd.0008657 PMC7549828

[ece371009-bib-0104] Zhang, C. , S. S. Dong , J. Y. Xu , W. M. He , and T. L. Yang . 2019. “PopLDdecay: A Fast and Effective Tool for Linkage Disequilibrium Decay Analysis Based on Variant Call Format Files.” Bioinformatics 35: 1786–1788. 30321304 10.1093/bioinformatics/bty875

