# Supplementary material for: Population Structure of the Invasive Asian Tiger Mosquito, Aedes albopictus, in Europe
Source: Ecol Evol. 2025 Mar 7;15(3):e71009. doi: 10.1002/ece3.71009 (PMC11886418; doi:10.1002/ece3.71009)
Supplement: Supplementary file 1 — Appendix S1. [file ECE3-15-e71009-s002.docx]

**Appendix 1.**

**Appendix Figures**


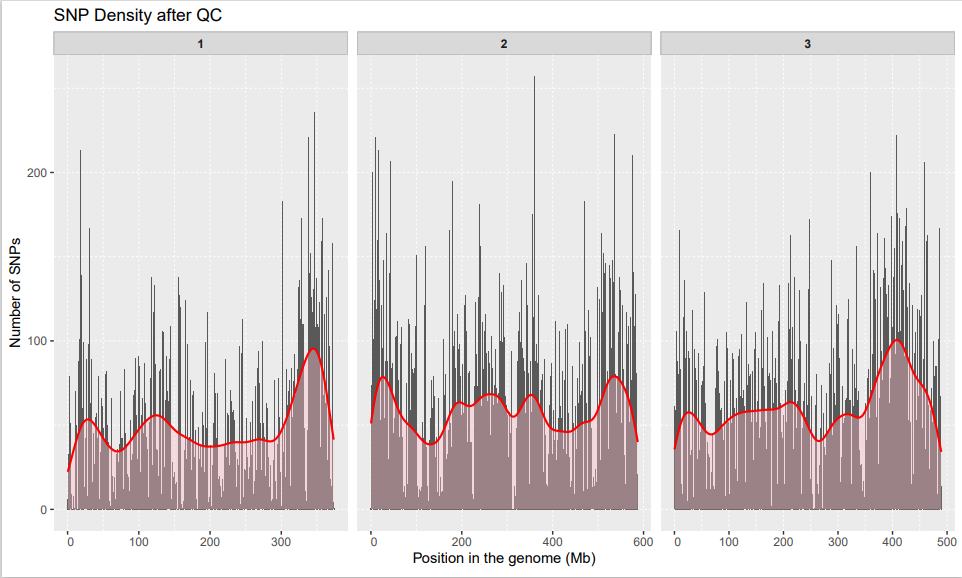


**Figure A1**. Density of 85,306 SNPs along the three chromosomes of the *Ae. albopictus* genome. The x-axis shows the genomic positions in Mb across each chromosome. The black vertical bars indicate the number of SNPs at each genome site, and the red line represents the mean SNPs density at each location. Prior to density analyses, 574 individual scaffolds were merged to create a chromosomal scale, as described in Cosme et al. 2024. The SNP data are from the 409 wild European samples retained in our analyses after quality control (Supplementary File 6).

A)

**
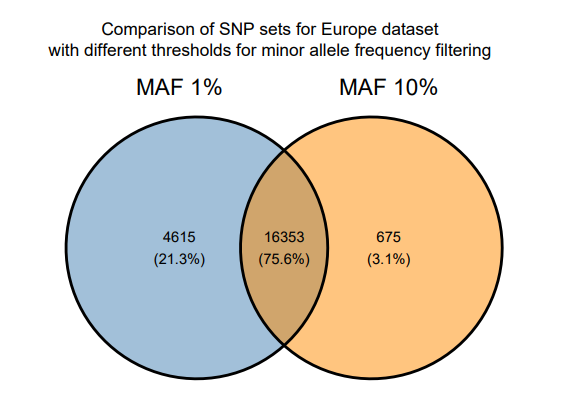
**

B)

**
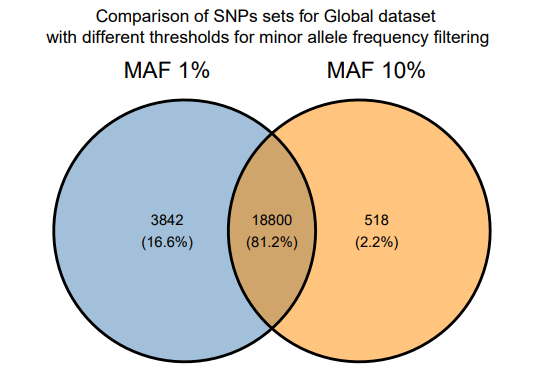
**

**Figure A2**. Venn diagrams comparing the variants retained in SNP Set 1 and SNP Set 3. The numbers inside the circles represent the number of SNPs with percentages in parenthesis. The two colors used for the Venn diagrams refer to two thresholds used to filter minor allele frequency (MAF) during quality control, with MAF <1% removed on the left, and MAF <10% removed on the right. Both SNP sets shown were pruned to remove linked SNPs (r^2^<0.01) after MAF pruning was completed (see Supplementary Files 6-7). SNP sets in A are for 409 samples in the European dataset and SNP sets in B are for the 643 samples in the Global dataset.

**
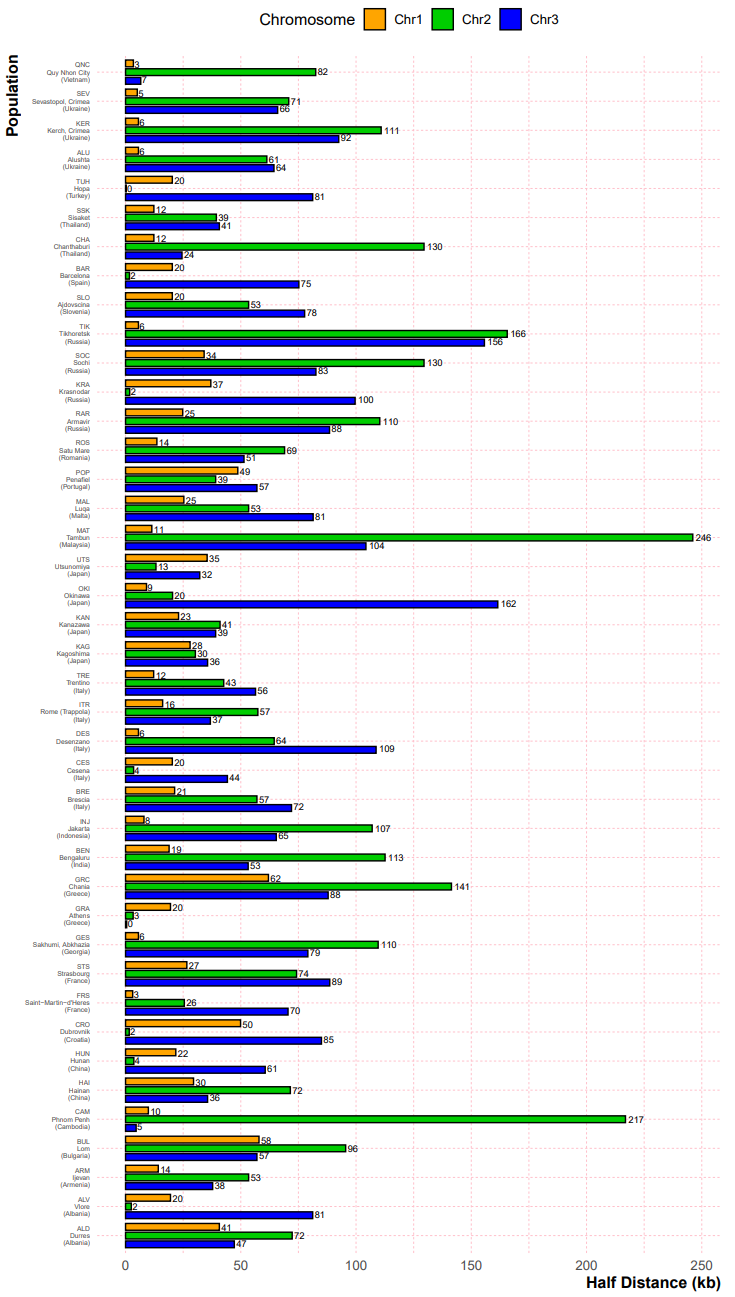
**

**Figure A3**. Linkage disequilibrium (LD) half distance, r^2^, estimated using PopLDdecay for *Ae. albopictus* populations in the Global dataset. The LD half distance (when LD falls to half its initial value) is shown on the x-axis in kilobases (kb). For each sampling location, horizontal bars represent the r^2^ in kb on each chromosome (Chr1 in orange, Chr2 in green, and Chr3 in blue). Sample sizes (N) are shown on the right.


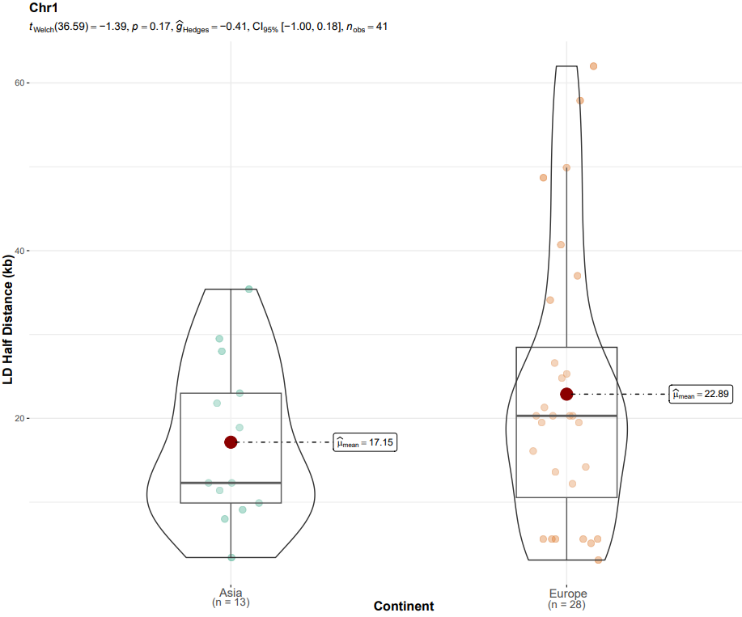


**
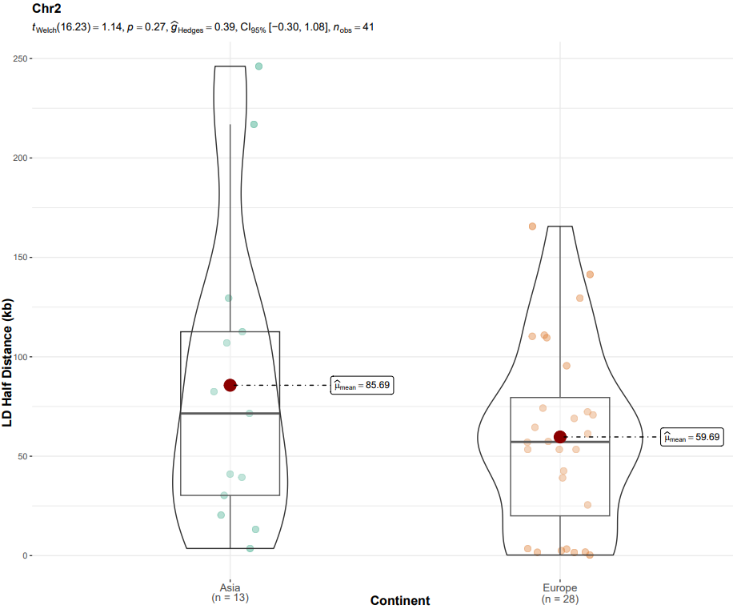
**

**
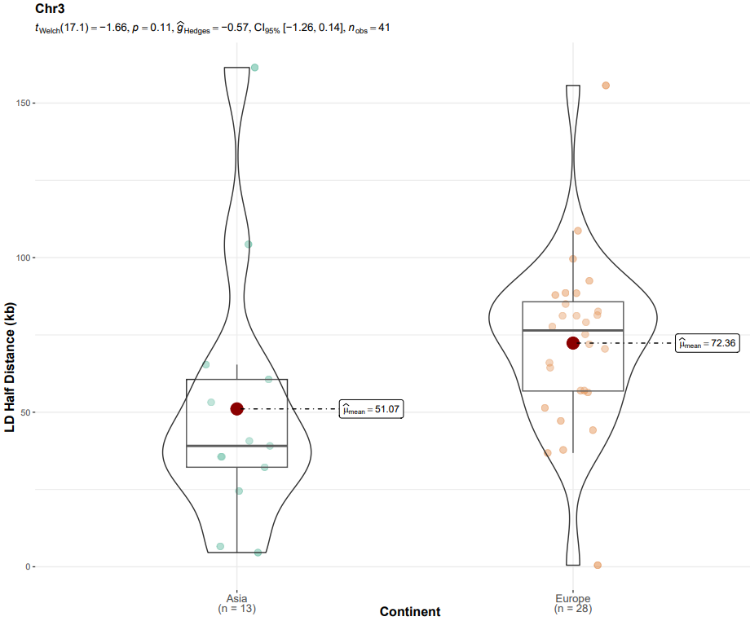
**

**Figure A4.** Comparison of the linkage disequilibrium (LD) half distance, r^2^, for populations in the Asia (native range) and Europe for each *Ae. albopictus* chromosome. LD half distances were computing in PopLDdecay and plots were created using the R package ggstatsplot.

**
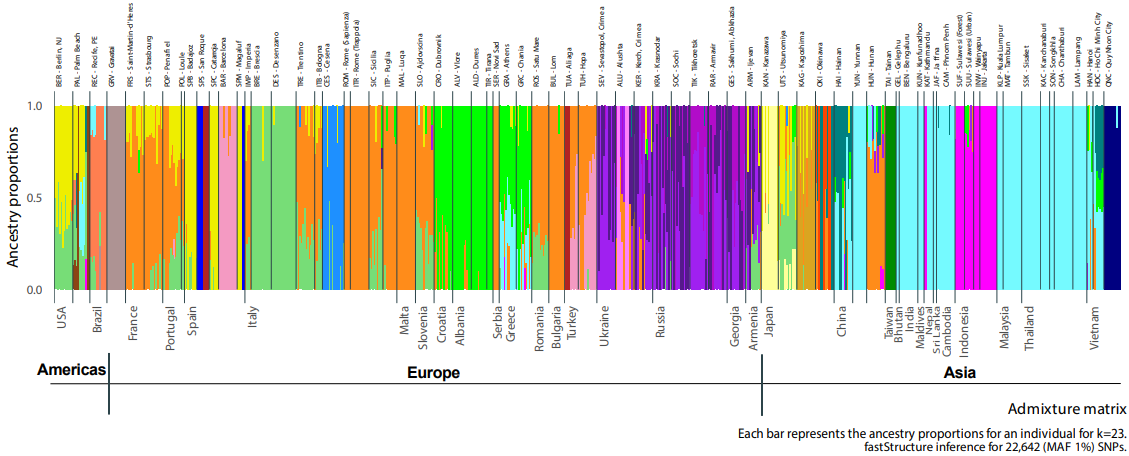

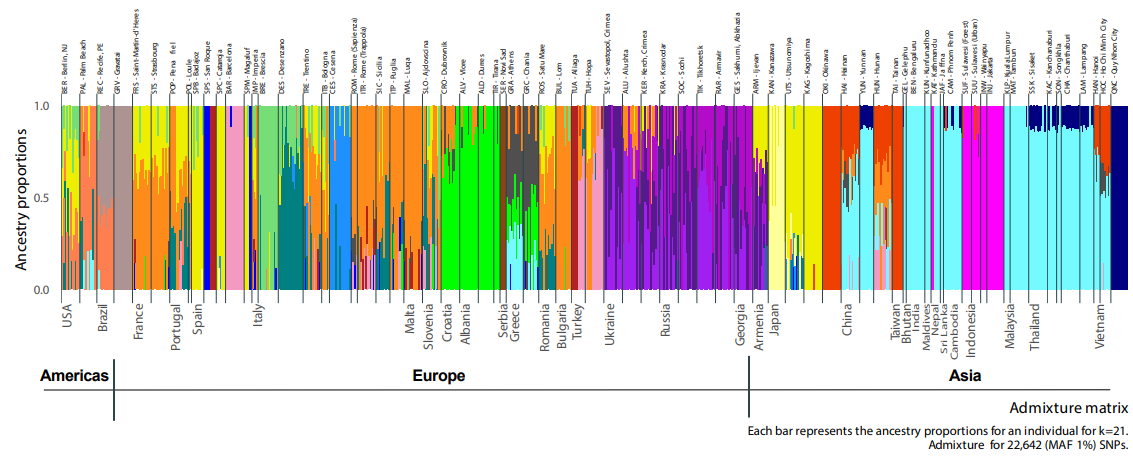
 Figure A5.** Populations structure of *Ae. albopictus* in Europe using the Global dataset. Ancestry matrices representing the best supported number of clusters (K) obtained from each clustering algorithm using SNP Set 3 are shown. Panels, from top to bottom, were obtained from fastStructure (K=23) run with simple prior, and Admixture (K=21). Results from LEA (K=20) are shown in Figure 2. In the plots, each vertical bar on the x-axis represents one mosquito and the y-axis shows the proportion of admixture for the ancestral genetic groups for each individual. See Figure 1 and Table A2 for full details on each sampling location.

A6.A)

**
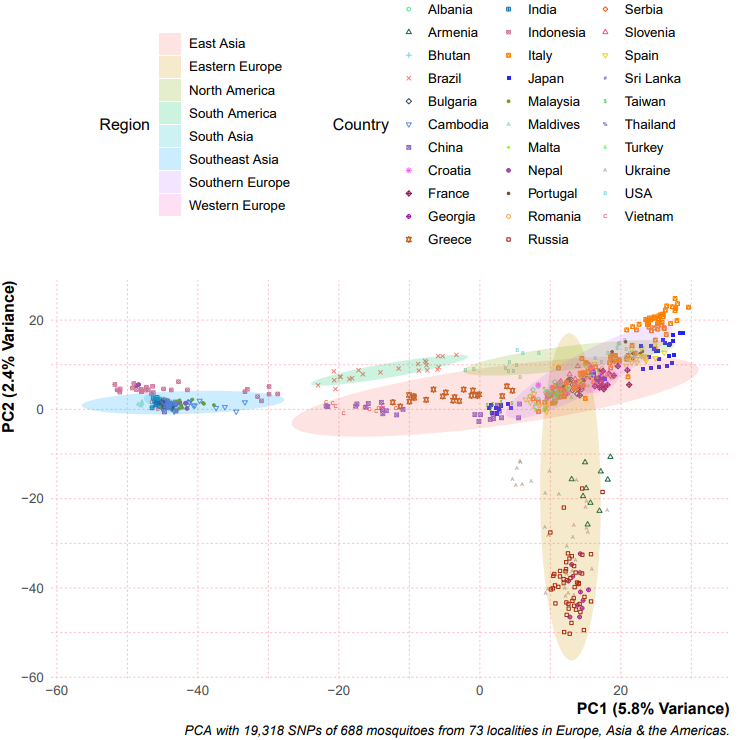
**


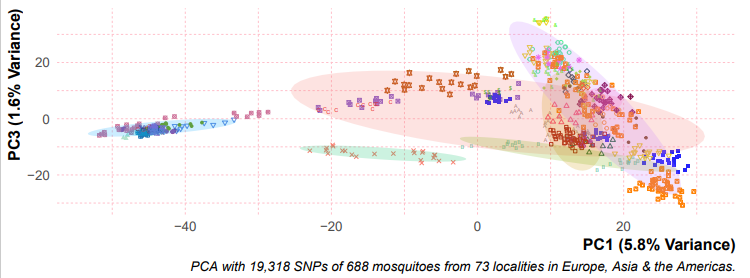


A6.B)

**
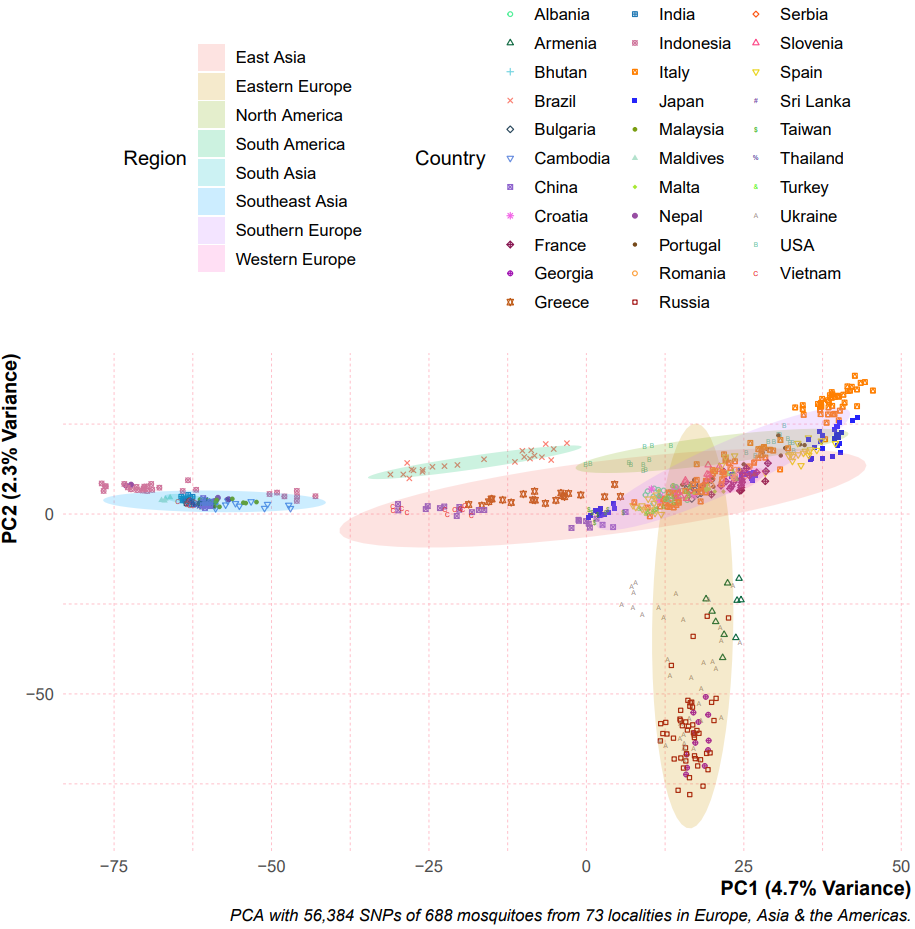
**

**
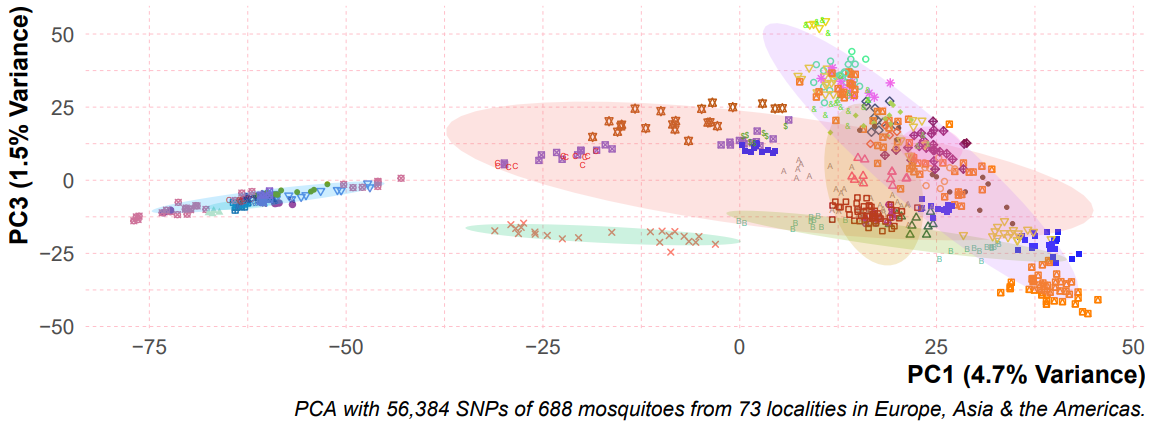
**

**Figure A6.** Scatterplots of principal component analysis (PCA) performed in the R package LEA showing the first three PCs for 688 *Ae. albopictus* mosquitoes in 73 localities in the Global dataset using A) SNP Set 1, B) SNP Set 2 (SNP Set 3 is shown in Figure 2 of the main text). In each plot the x-axis is principal component 1 and the y-axis is principal component (PC) 2 in the top panel and PC 3 in the bottom panel. The variance explained by each principal component is in parentheses on the y- and x-axes. Each symbol represents a mosquito, and the color and shape of the symbol indicates the country where they were sampled. Ellipses mark each region in Europe covering 80% of the samples. Additional plots and details of the PCA analyses are shown in Supplementary files 12 and 13.

A7.A)


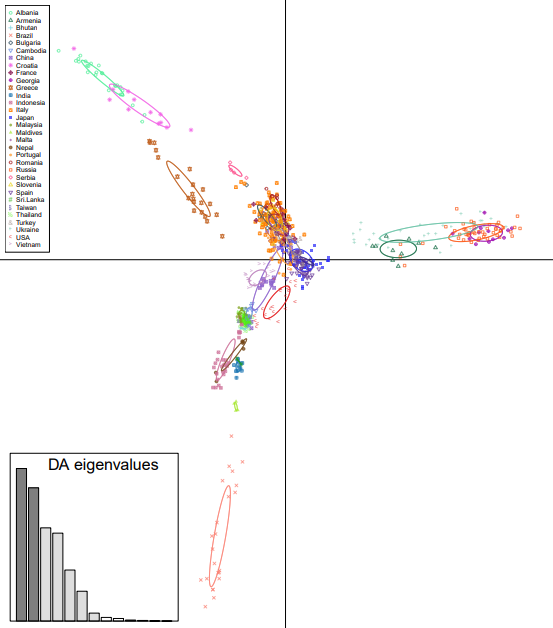


A7.B)

**
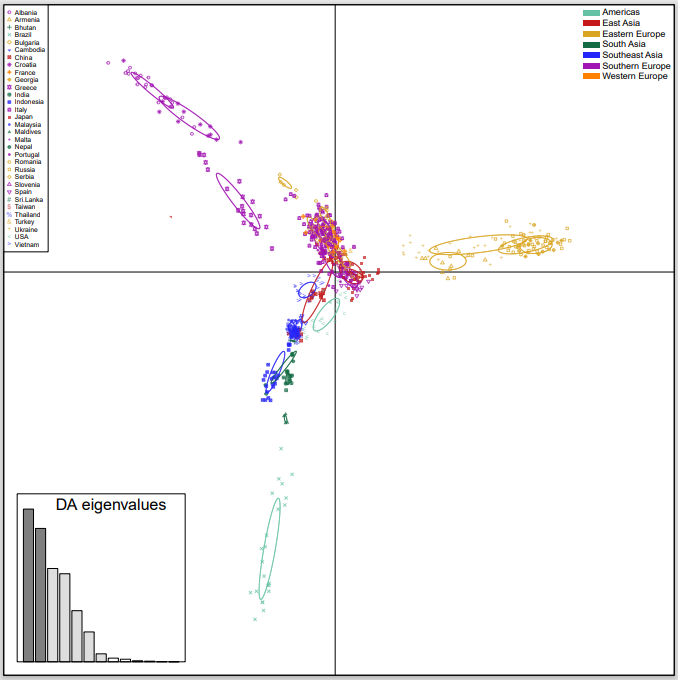
**


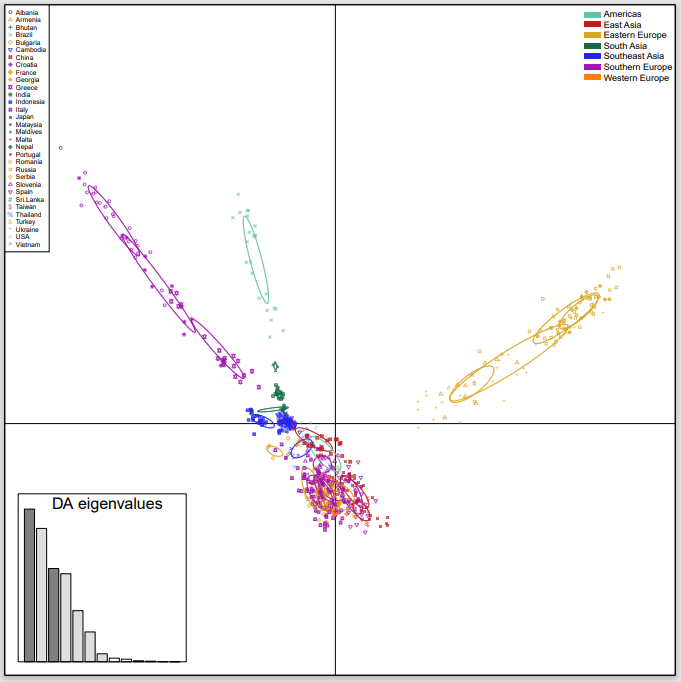


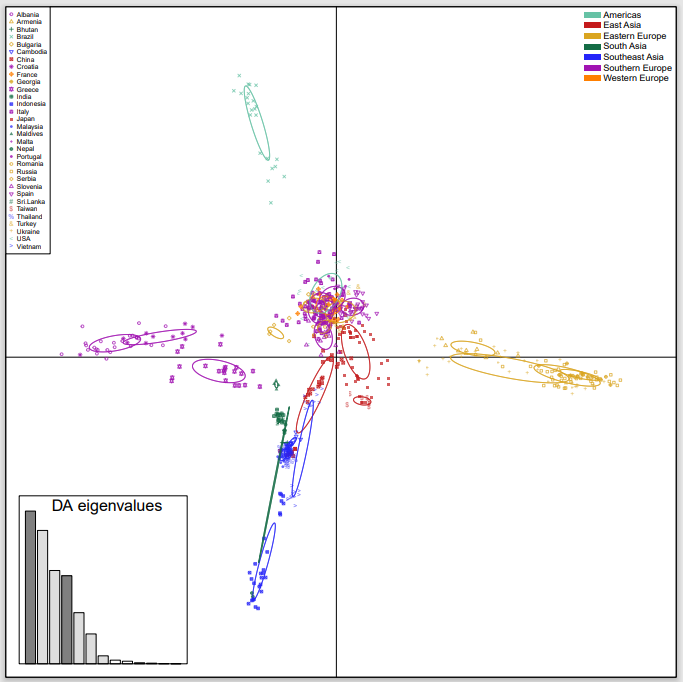


**Figure A7**. Scatterplots of the principal components derived from DAPC using SNP Sets 3 for the A) Global dataset showing each country in a different color, and B) Global dataset with locations grouped by region showing PCs 1 and 2 in top panel, PCs 1 and 3 in center panel, and PCs 1 and 4 in bottom panel. Each symbol represents a mosquito, and the color and shape of the symbol indicates the location where they were sampled. Points are plotted according to its scores on the discriminant functions, and axes are scaled to the eigenvalues, which represent the relative contribution of each function (shown in the inset bar graph) to the total genetic variance observed.

A8.A)


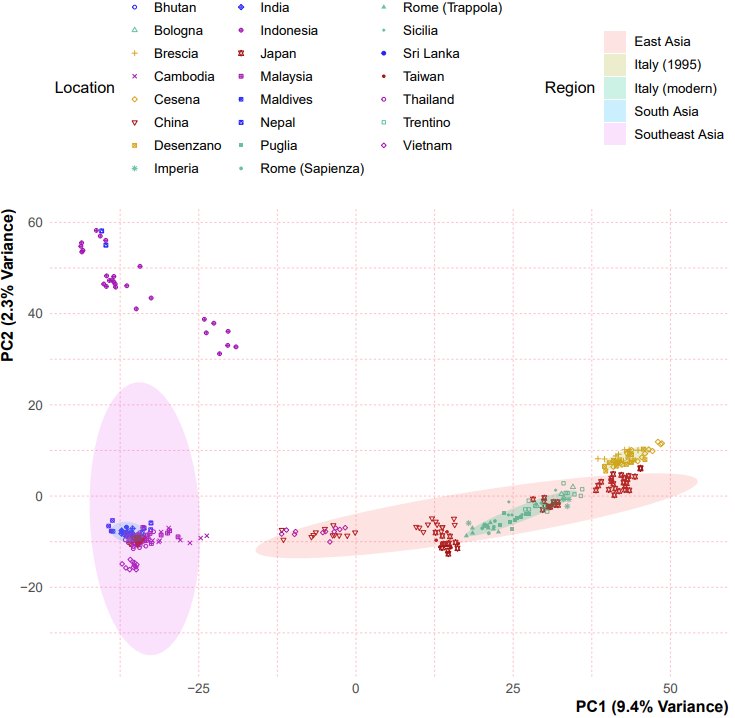


A8.B)


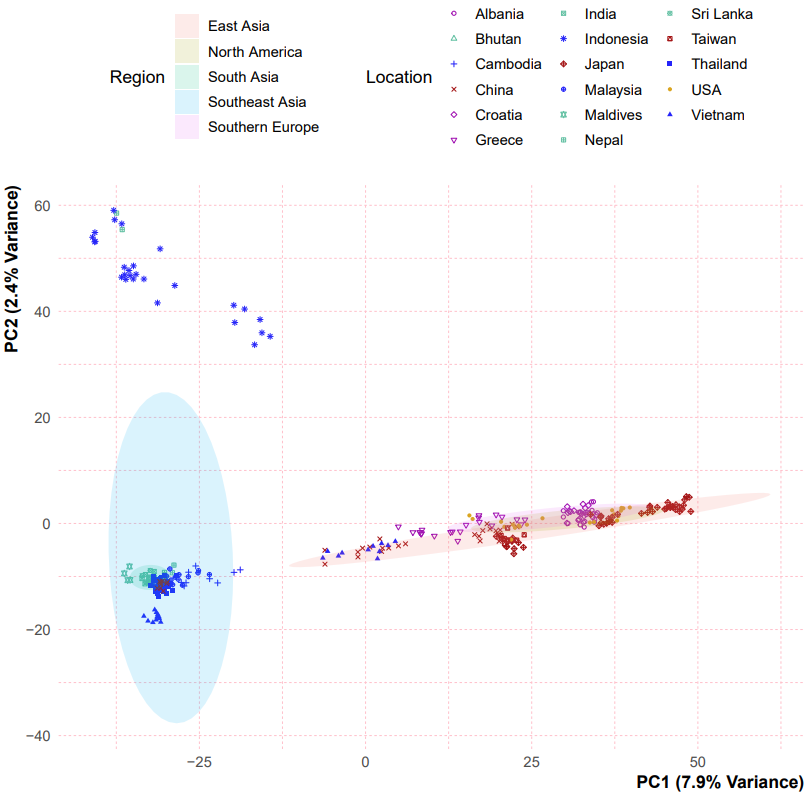


A8.C)


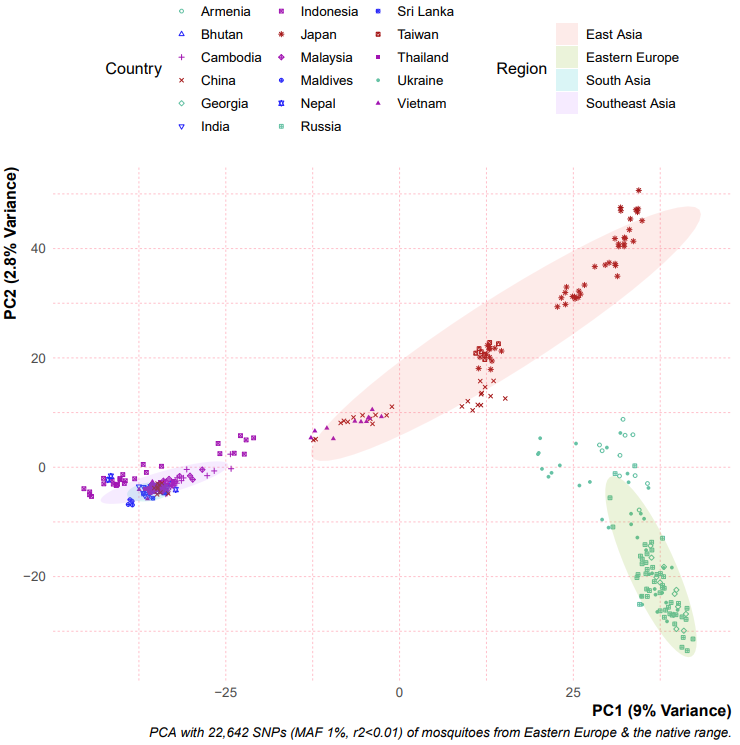


A8.D)


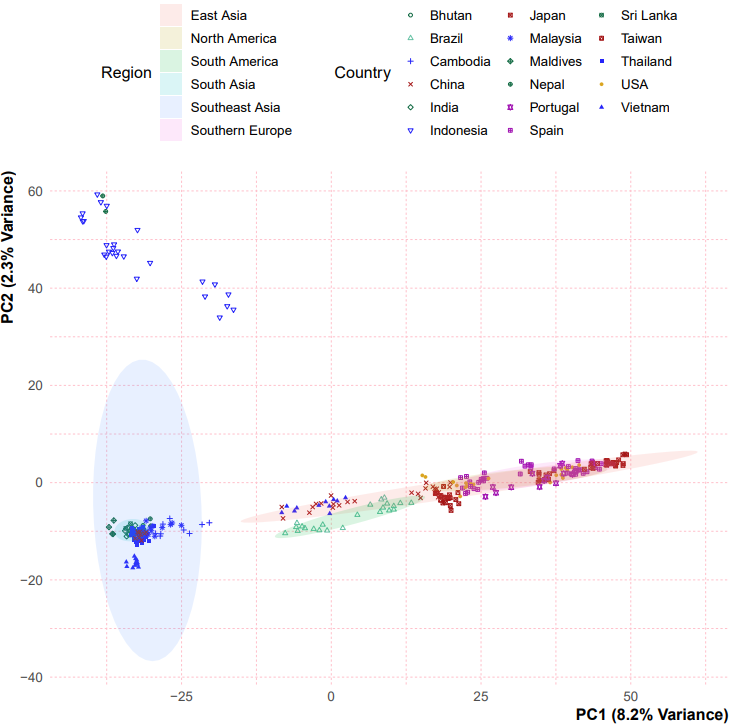


**Figure A8.** Scatterplot of PCA carried out on subsets of the Global dataset to highlight the relationships between European regions and the native range. A) Italy, B) Greece, Albania and Croatia, C) Eastern Europe, and D) Iberian peninsula. Each country is represented by a unique symbol and the color and ellipses indicate the region to which the population belongs.

A9.A)


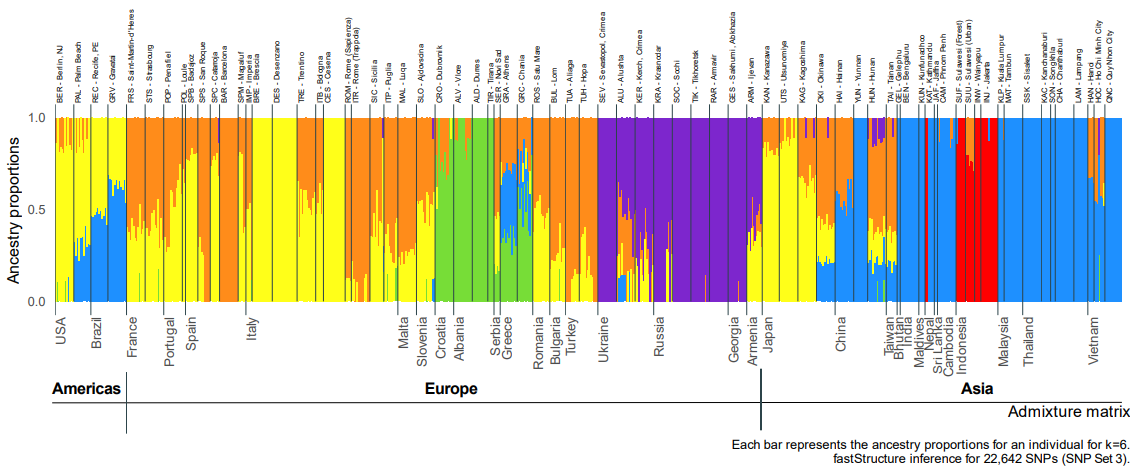


**
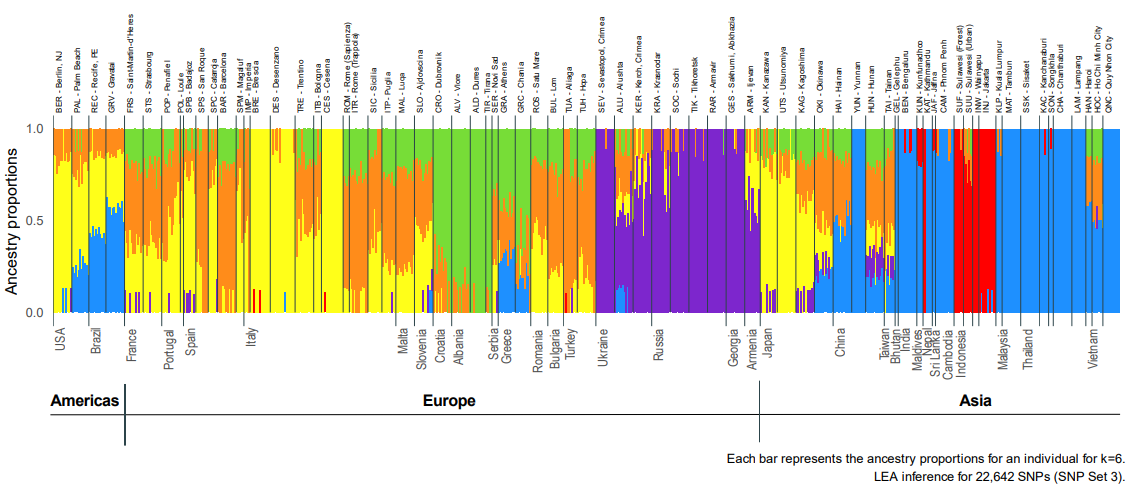
**


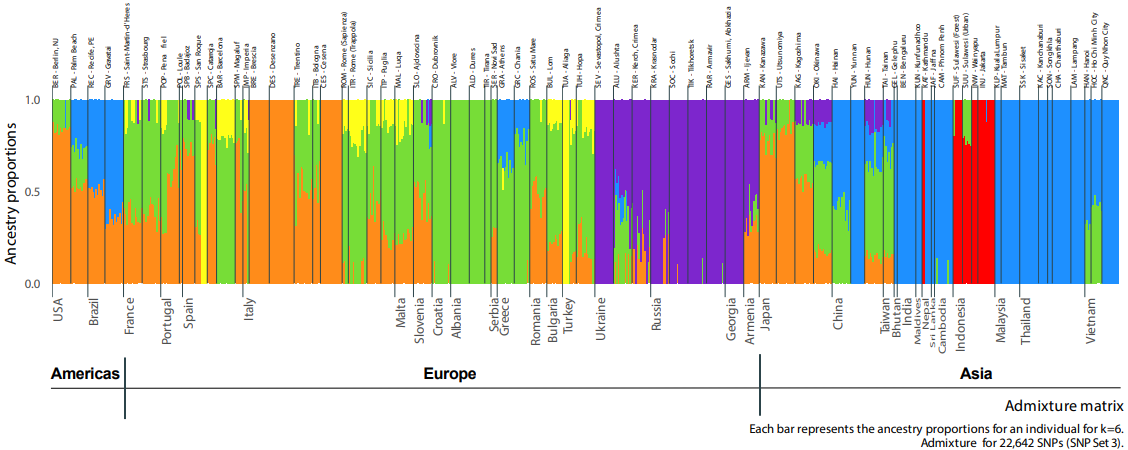


A9.B)


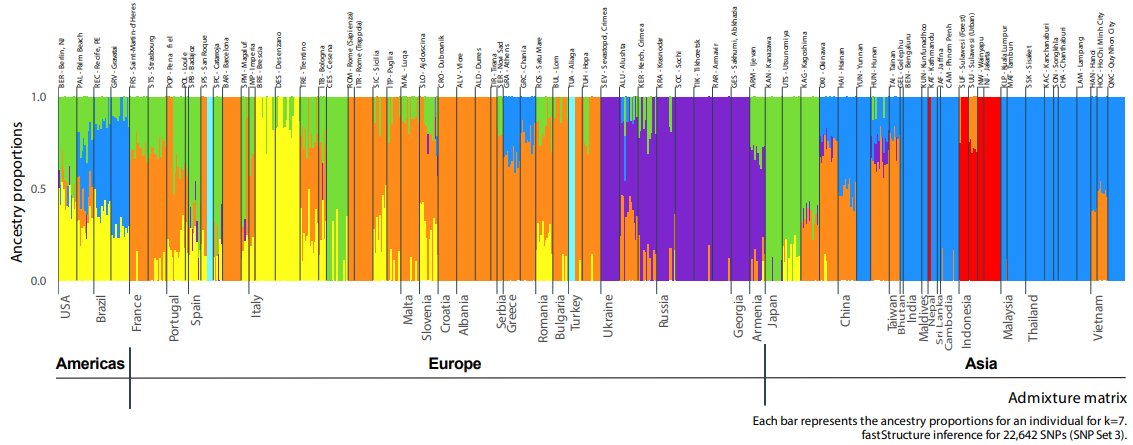

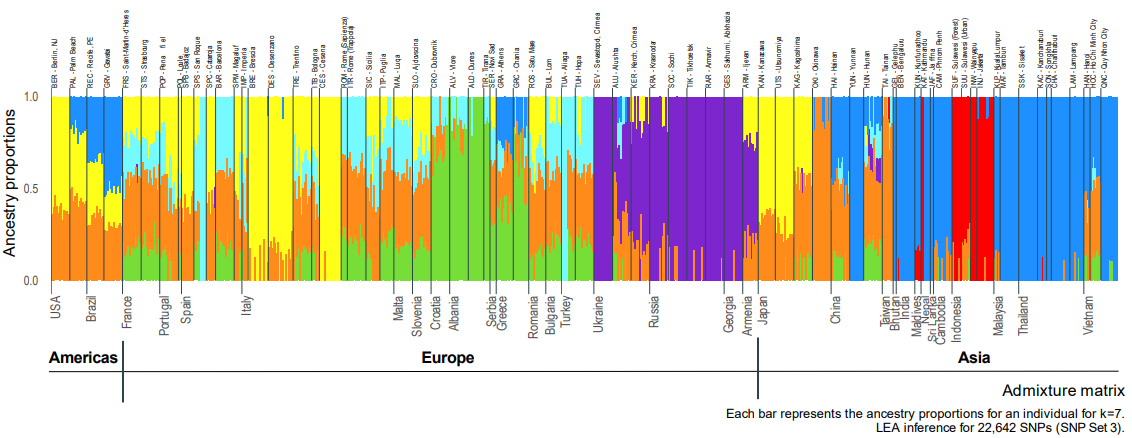

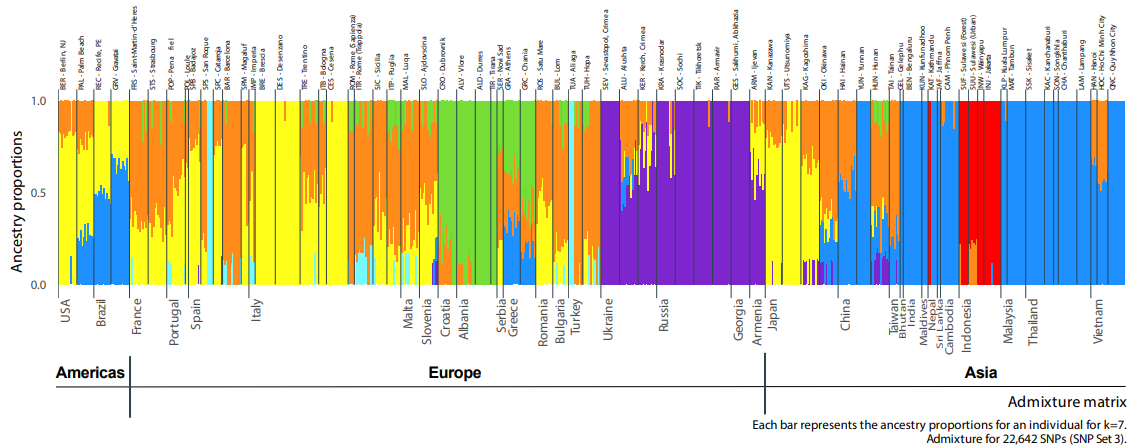


**Figure A9.** Ancestry matrices of Global dataset for 688 mosquitos sampled from American, European, and native locations using SNP Set 3. Panel A shows plots for K=6 for, from top to bottom, fastStructure, LEA, admixture. Panel B shows plots for K=7 for, from top to bottom, fastStructure, LEA, admixture. In all plots, each vertical bar on the x-axis represents one mosquito, and the y-axis shows the proportion of admixture for each individual ancestral genetic group.

A10.A)
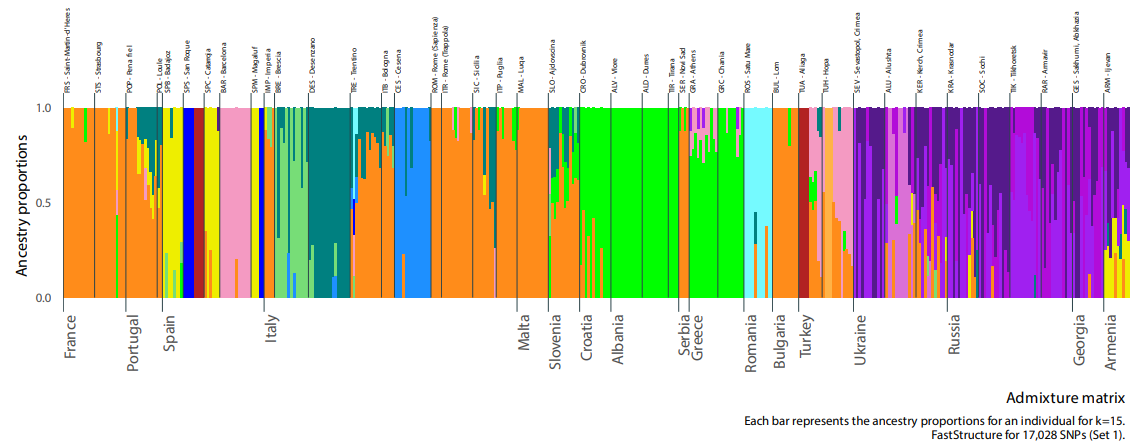
**
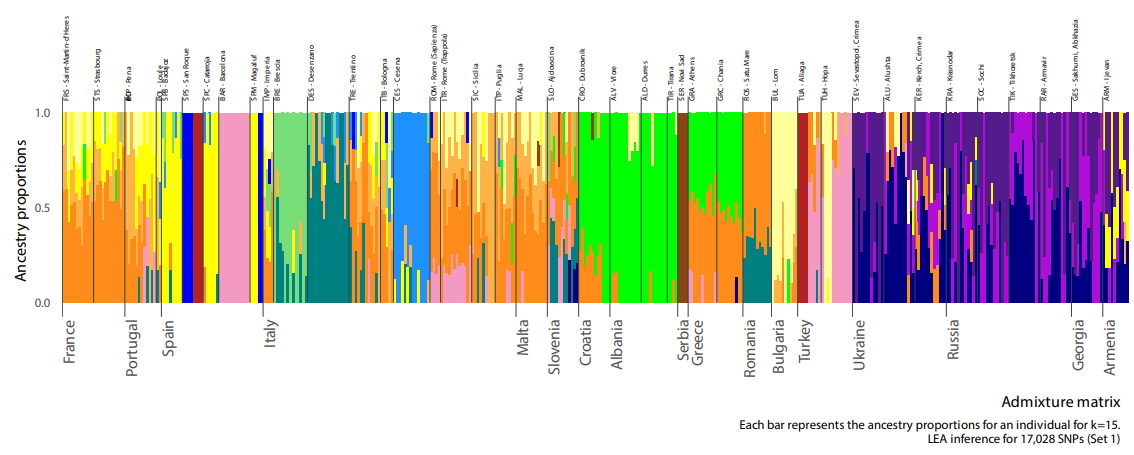
**
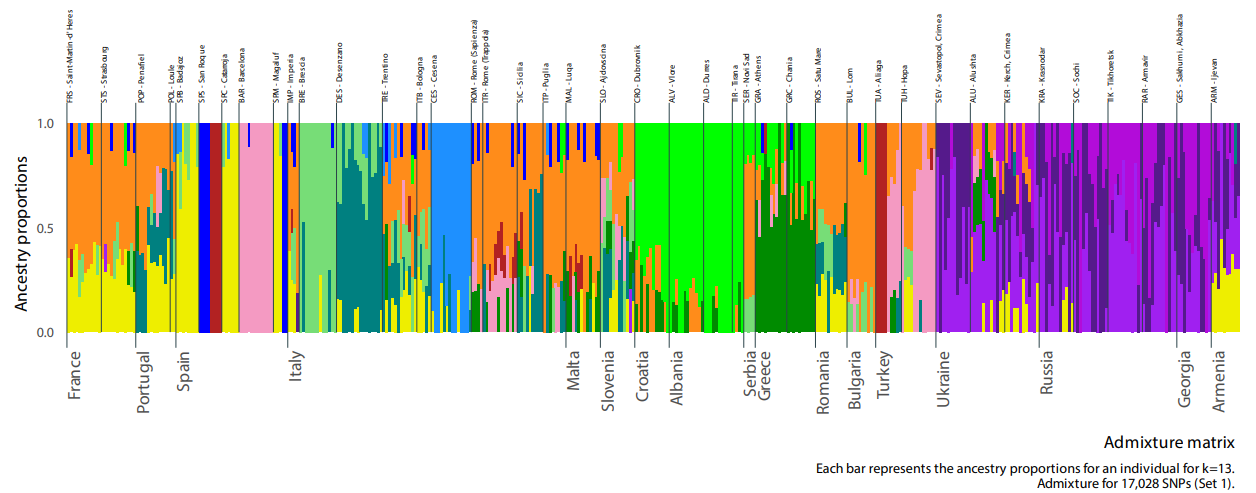


A10.B)
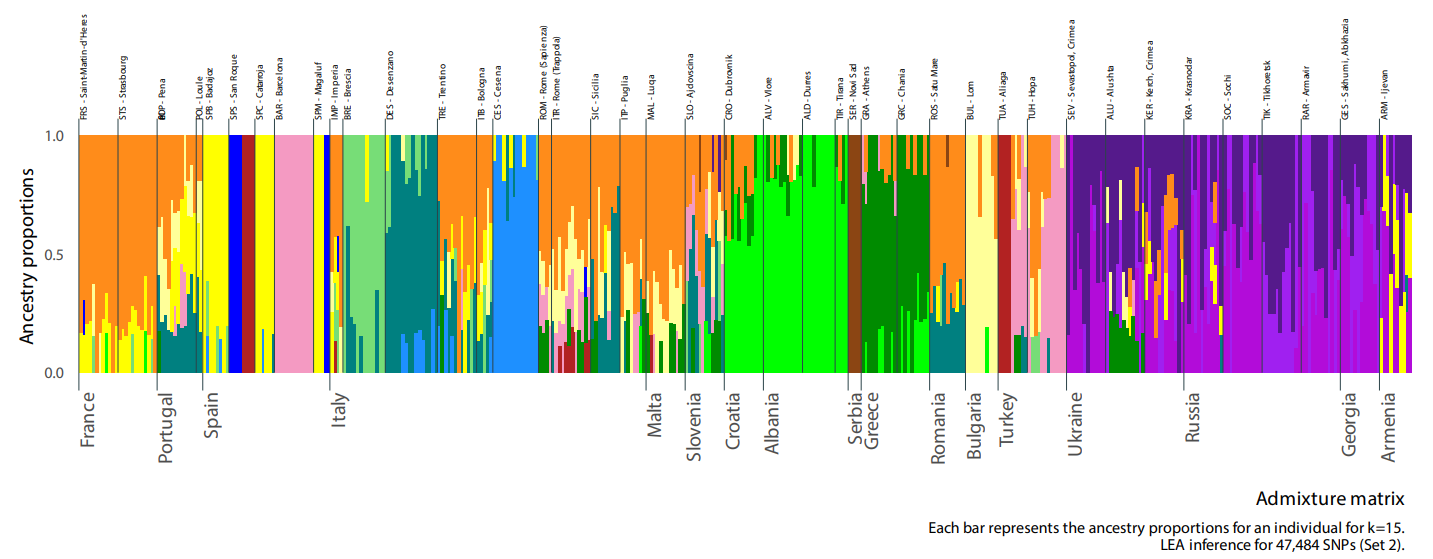

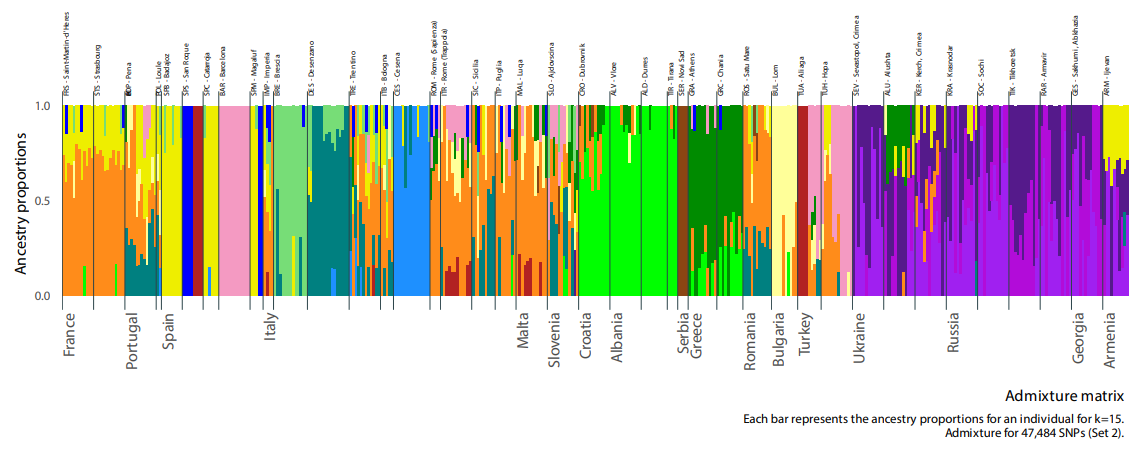

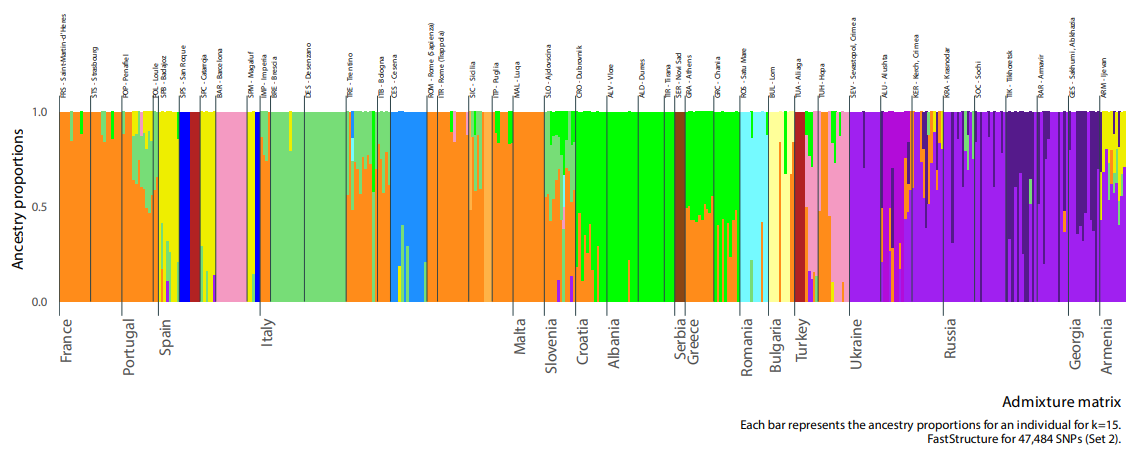


A10.C)
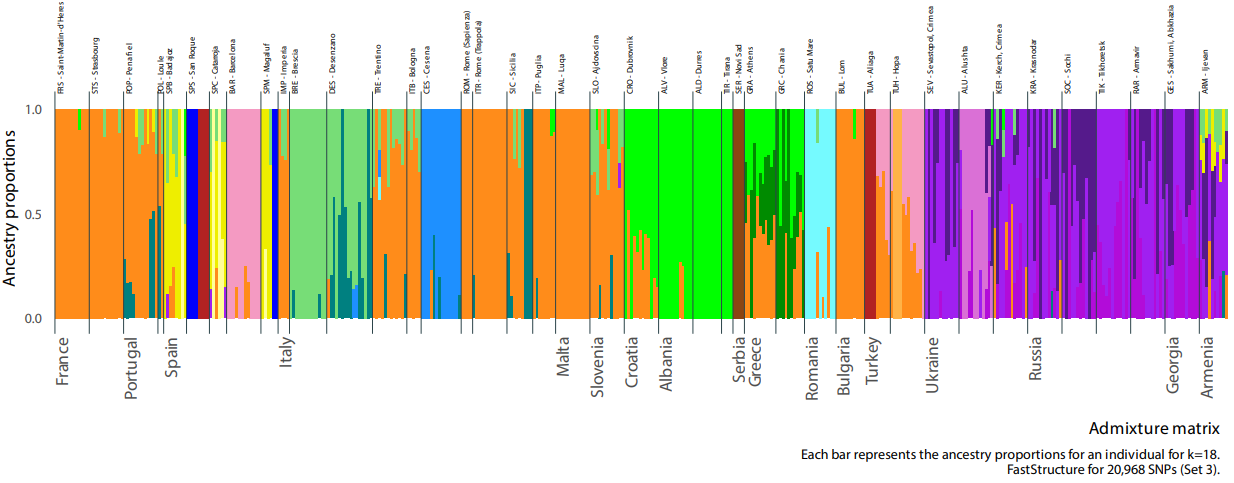

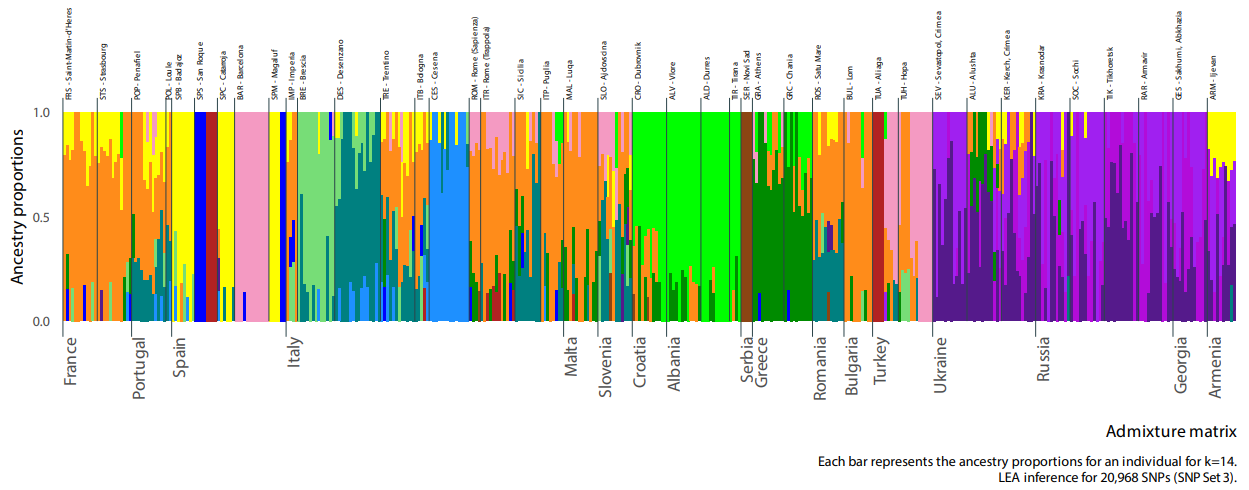

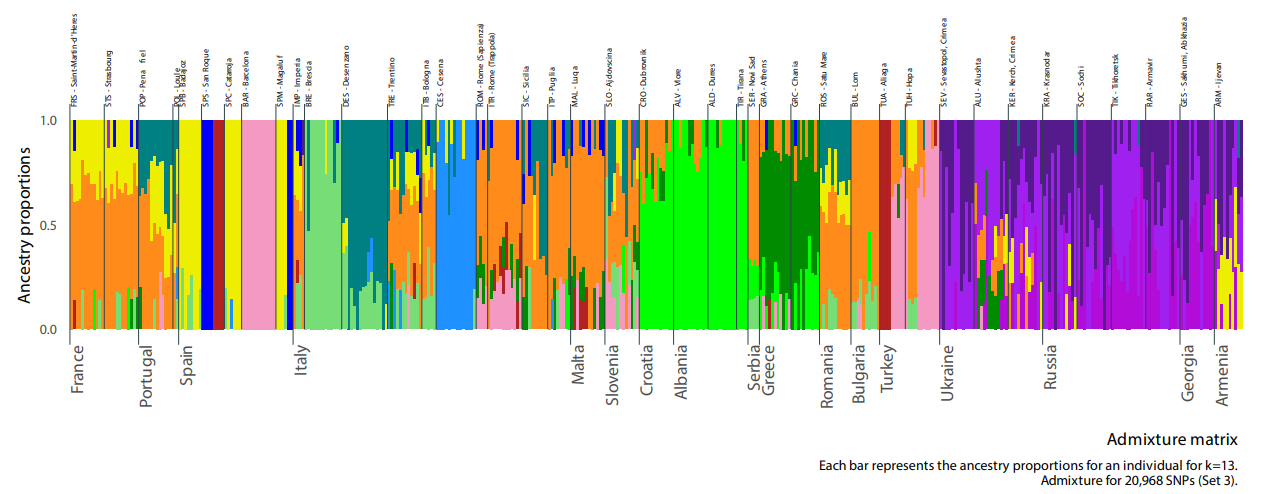
**Figure A10.** Populations structure of *Ae. albopictus* in Europe using 409 mosquitos from 41 sampling sites in 17 countries. Plots were created from Q matrices representing the best supported number of clusters (K) obtained from each clustering algorithm using A) SNP Set 1 (r^2^ < 0.01, ~17,028 SNPs), B) SNP Set 2 (r^2^ < 0.1, ~ 47,484 SNPs), and C) SNP Set 3 (r^2^ < 0.01 and MAF >1%, ~ 20,968 SNPs). In each section of the figure, plots were obtained from fastStructure run with simple prior (top), LEA (middle), and Admixture (bottom). In the plots, each vertical bar on the x-axis represents one mosquito and the y-axis shows the proportion of admixture for the ancestral genetic groups for each individual.

A11.A)


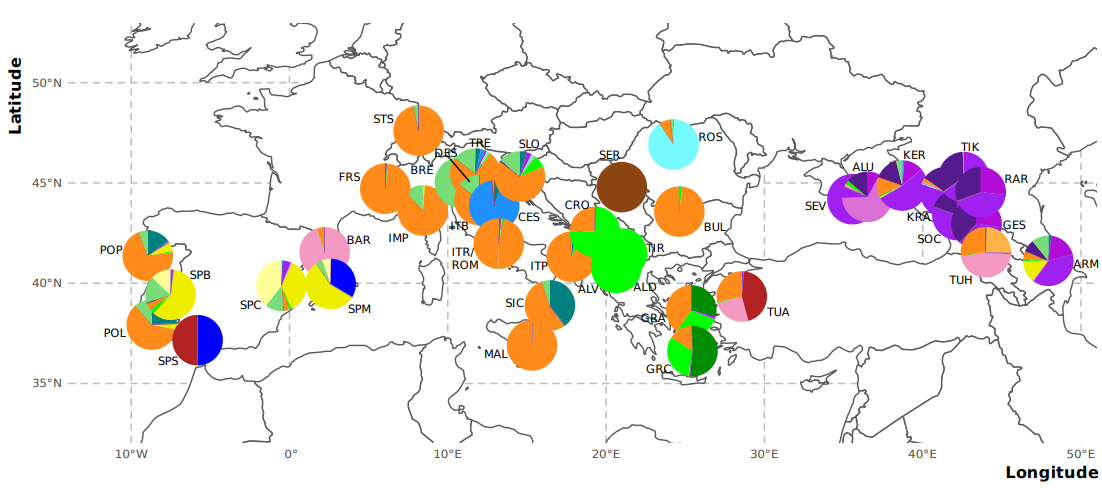


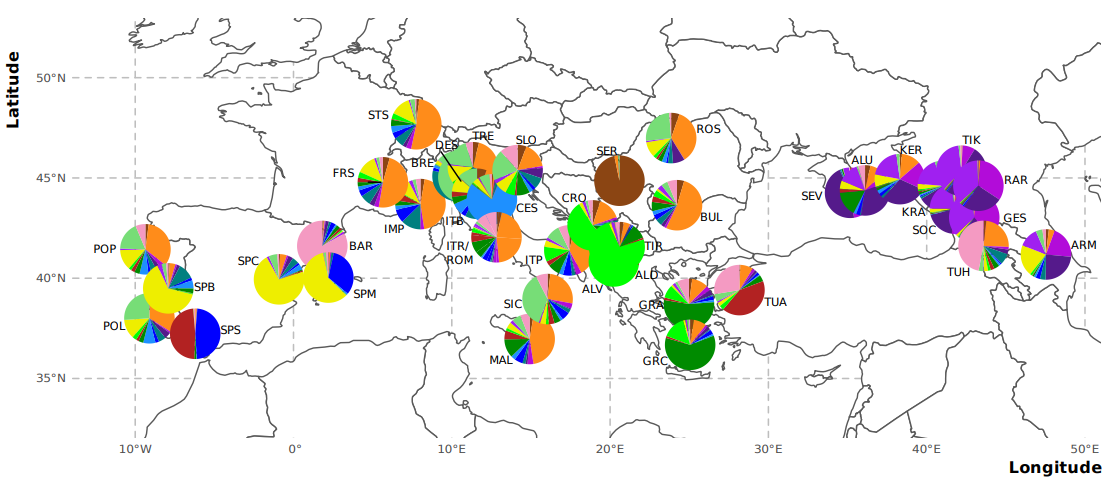


**
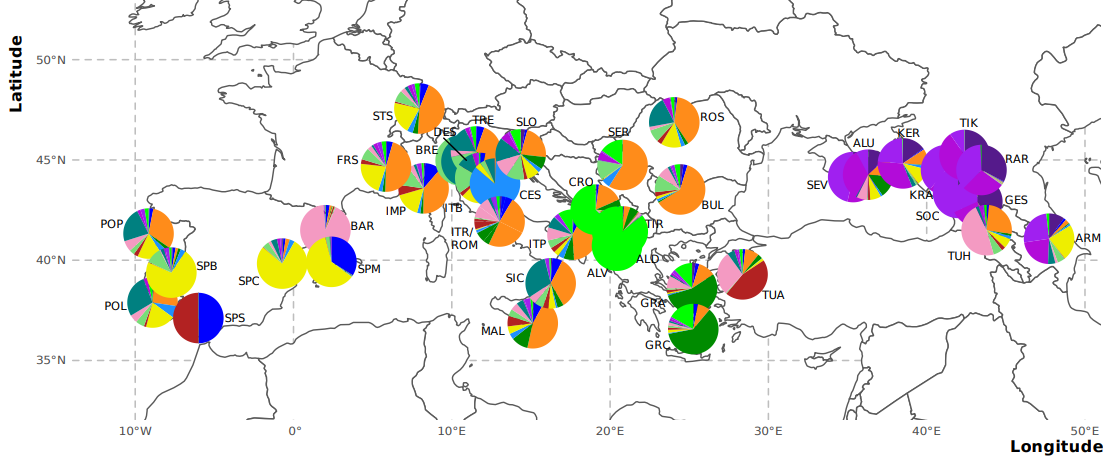
**

A11.B)


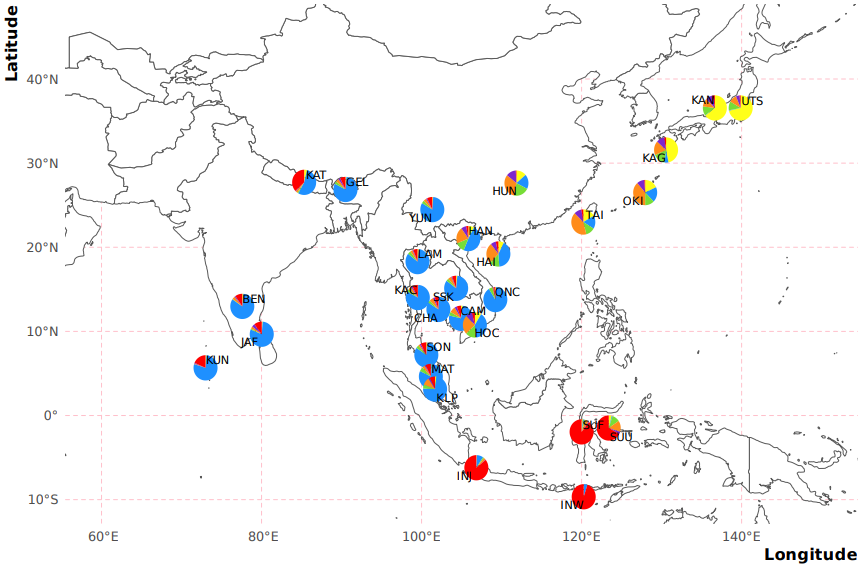
**
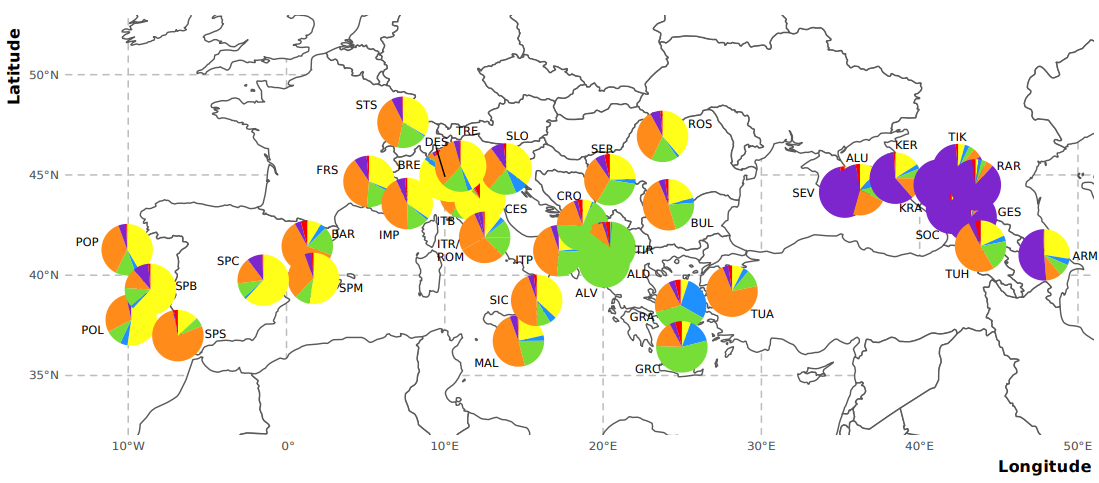
**

**Figure A11**. A) Map of the ancestry matrix for European samples showing the best K obtained from fastStructure (K=18) in the top panel, from LEA (K=14) in the middle panel, and from admixture (K=13) in the bottom panel. B) Maps of the ancestry matrix from LEA showing K=6 obtained when all 688 samples in the global dataset were analyzed together (top panel shows a close-up of the native range; lower panel shows European populations). The ancestry matrices used to create all maps were obtained using SNP Set 3. The colored pies at each sampling site reflect the proportion of the clusters found in each location. Abbreviated codes are used for each locality (see Figure 1 and Table A2 for full details on each sampling location). Maps obtained from the other structure algorithms and plots of data from SNP Sets 1 and 2 are available in Supplementary files 16-18.

A12


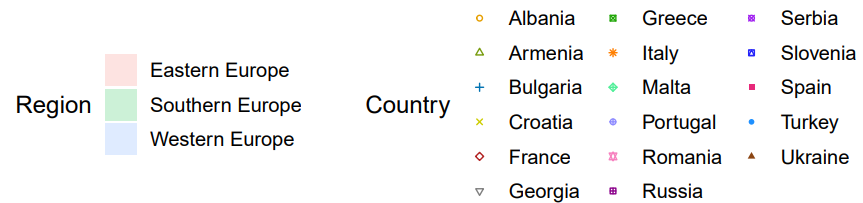


A12.A)


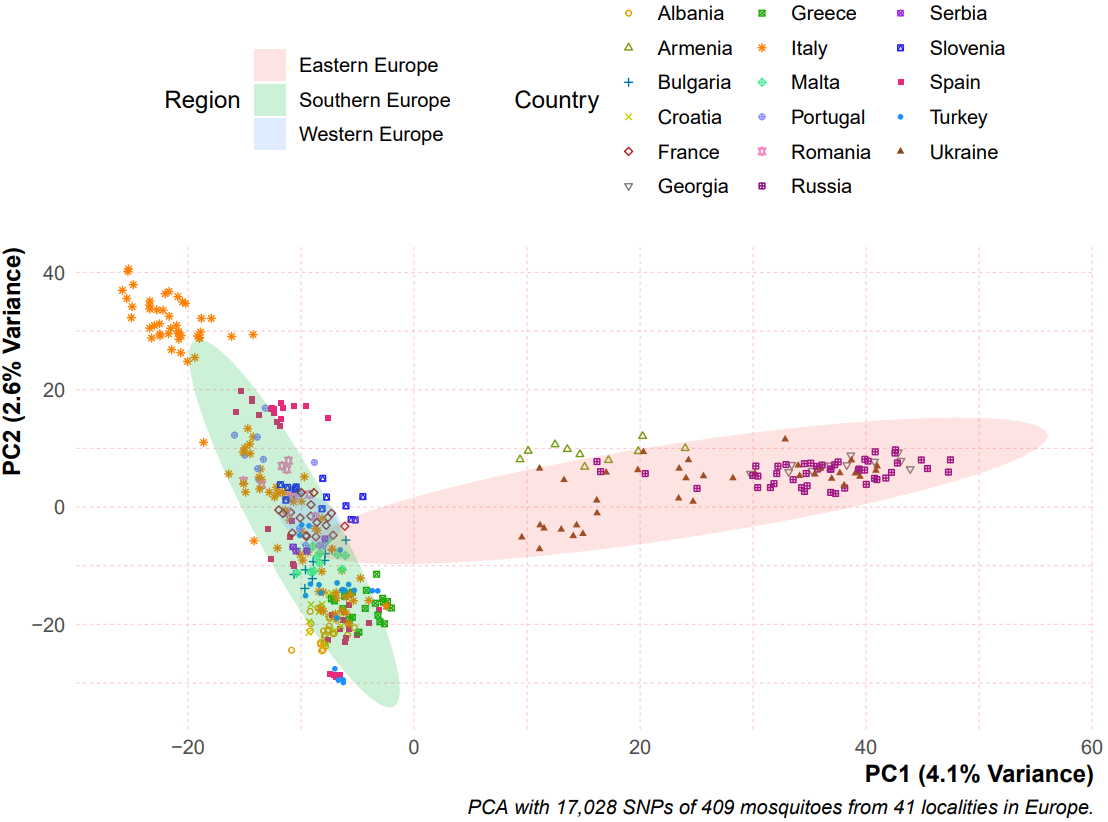


A12.B)


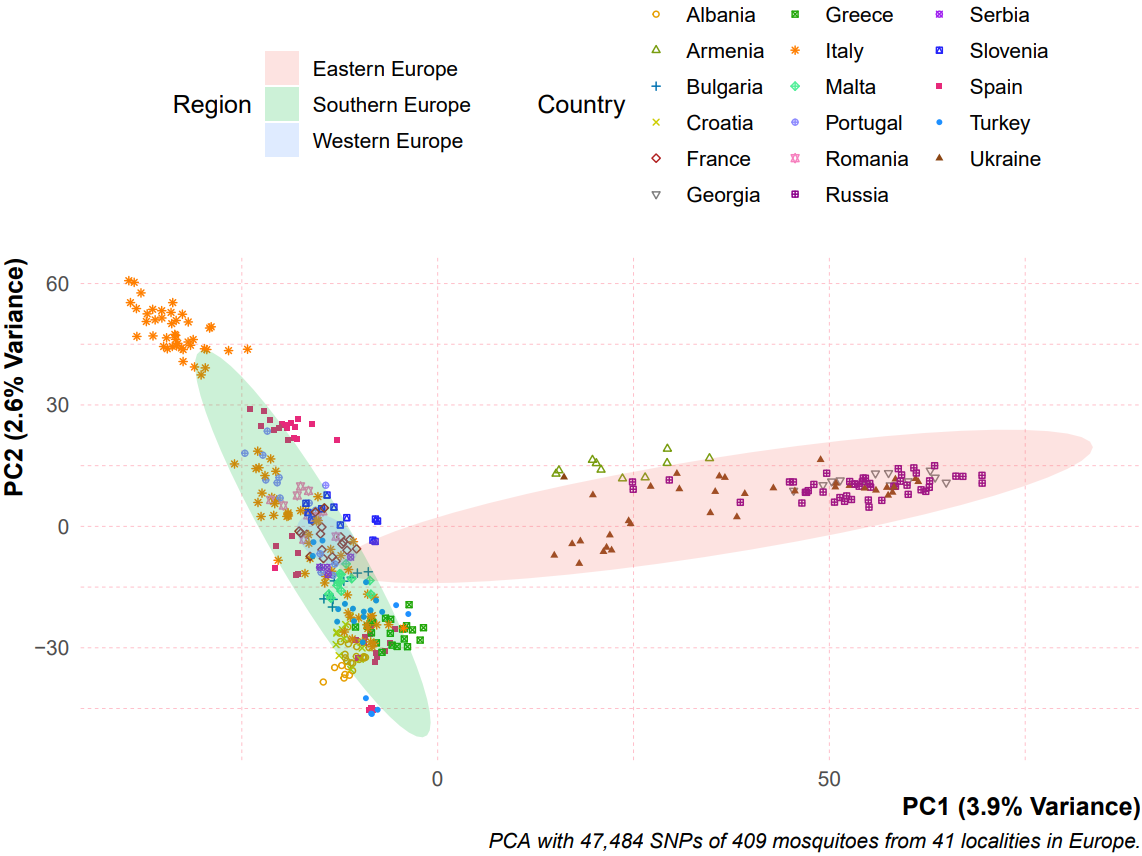


**Figure A12.** Principal component analysis performed in the R package LEA for 17 European countries using all three SNP Sets. Panels, from top to bottom, show plots for A) Set 1, B) Set 2, and C) Set 3 (see Table A4 for details about each SNP set). In each plot the x-axis is principal component 1 and the y-axis is principal component 2, 3 or 4. The variance explained by each principal component is in parentheses on the y- and x-axes. Each symbol represents a mosquito, and the color and shape of the symbol indicates the country where they were sampled. Ellipses mark each region in Europe covering 80% of the samples.

**
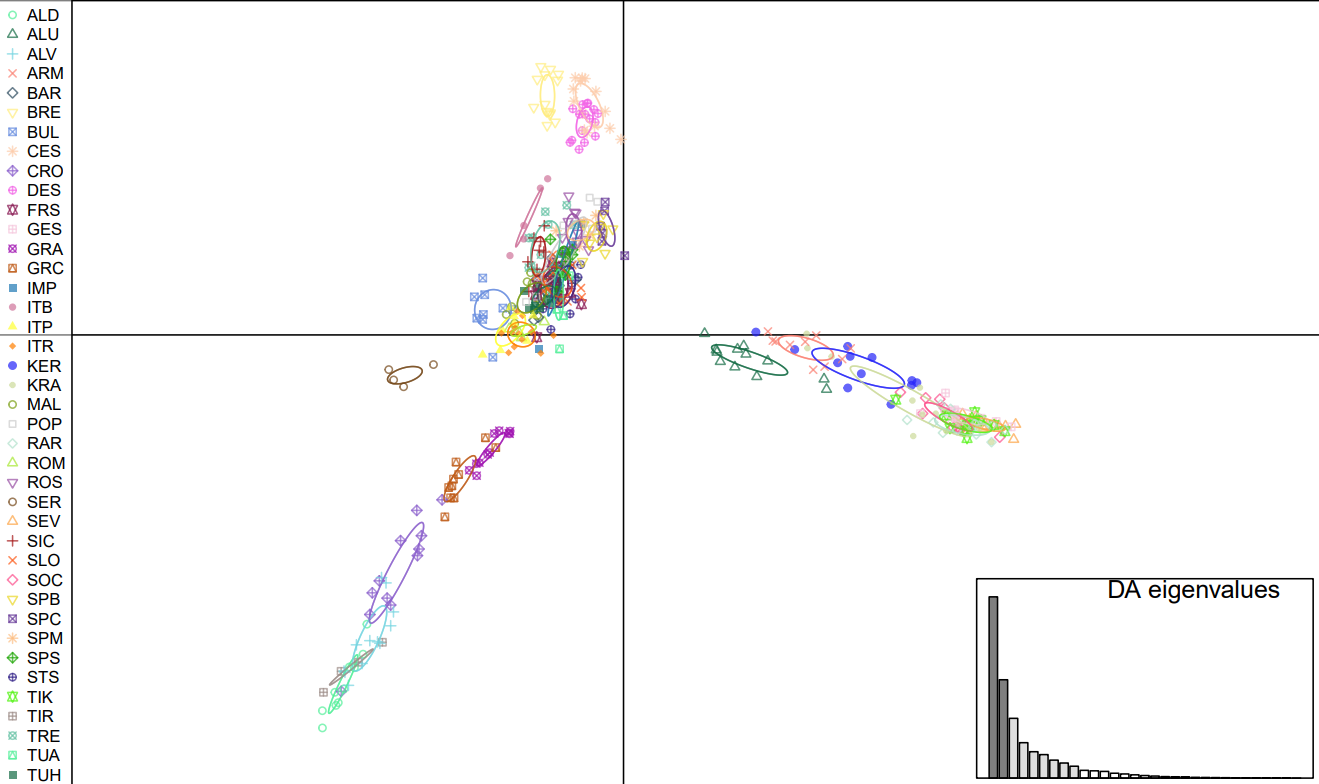
**

**Figure A13.** Scatterplot of the first two principal components derived from DAPC using SNP Sets 3 for each location in Europe showing each sampling location in a different color. Each symbol represents a mosquito, and the color and shape of the symbol indicates the location where they were sampled. Points are plotted according to its scores on the discriminant functions, and axes are scaled to the eigenvalues, which represent the relative contribution of each function (shown in the inset bar graph) to the total genetic variance observed. Abbreviated codes are used for each locality (see Figure 1 and Table A2 for full details on each sampling location).

A14.A)
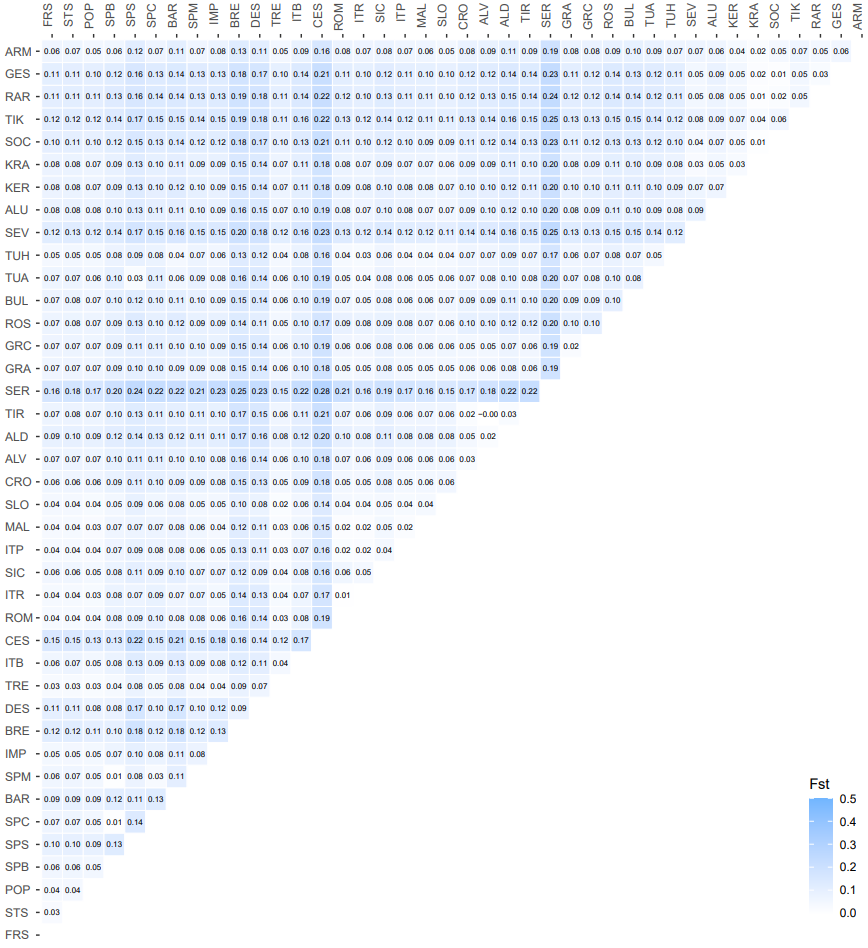


A14.B)


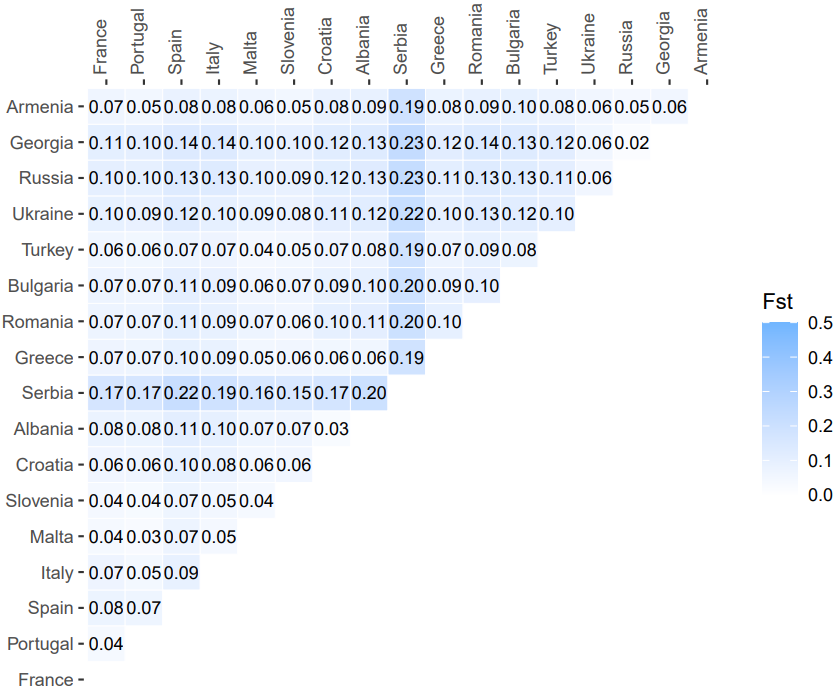


**Figure A14**. Matrix of pairwise FST values calculated using the StAMPP package in R with SNP Set 3 for A) all European localities (N=41), and B) mean FST by country for Europe (N=17). Pairwise FST values for countries were determined by computing the average FST across all populations within a country. See Supplementary File 19-20 for FST matrices for SNP Sets 1 and 2.


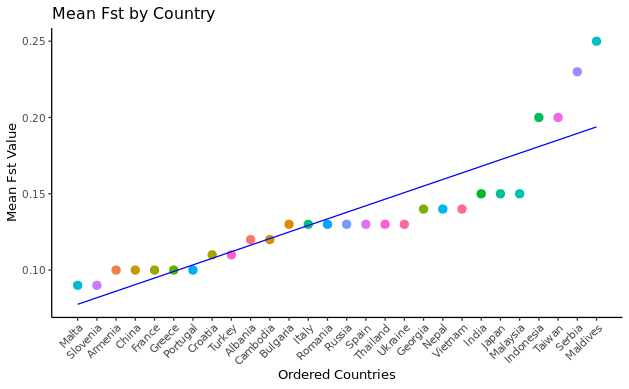


**Figure A15.** Mean Fixation Index (FST; y-axis) for countries in Europe and the native range computed using SNP Set 3 with the StAMPP package in R. Pairwise FST values were determined across all populations within a country, which were then averaged to estimate the mean FST per country. We fit a linear regression line to visualize the trend in mean FST across locations. Countries are sorted from left to right based on ascending mean FST values.

**
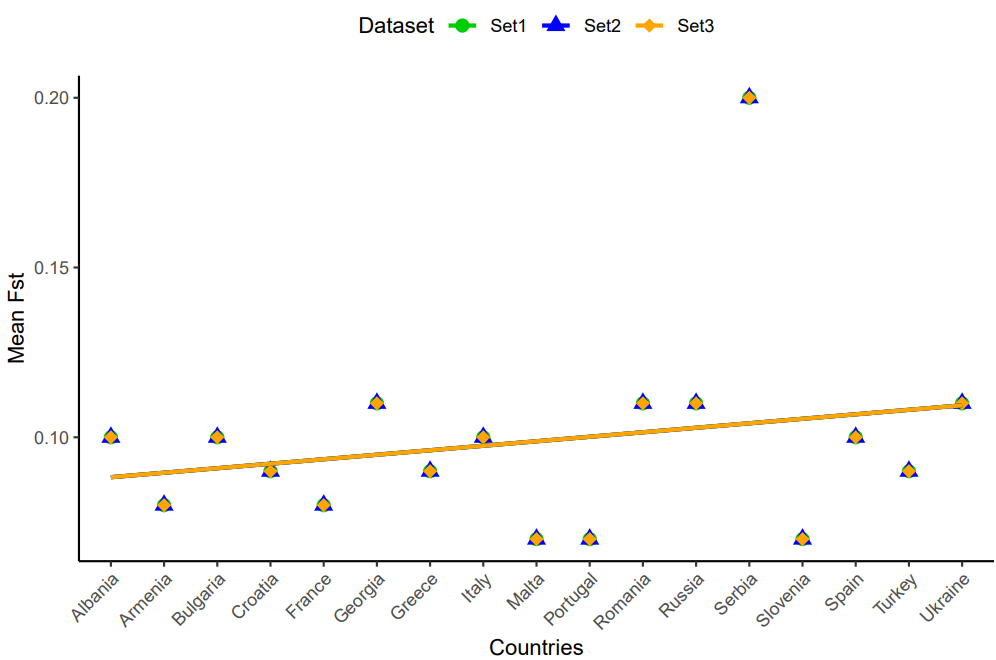
**

**Figure A16.** Mean Fixation Index (FST; y-axis) computed using the StAMPP package in R for European countries, using each of the three SNP sets: Set 1 (r2=0.01, MAF=10%, green), Set 2 (r2=0.1, MAF=10%, blue), and Set 3 (r2=0.01, MAF=1%, orange). For every SNP set, pairwise FST values were determined across all populations within a country, which were then averaged to estimate the mean FST per country. We fit a linear regression line for each SNP set to visualize the trend in mean FST across locations. Countries are sorted from left to right based on ascending mean FST values.

A17.A)

**
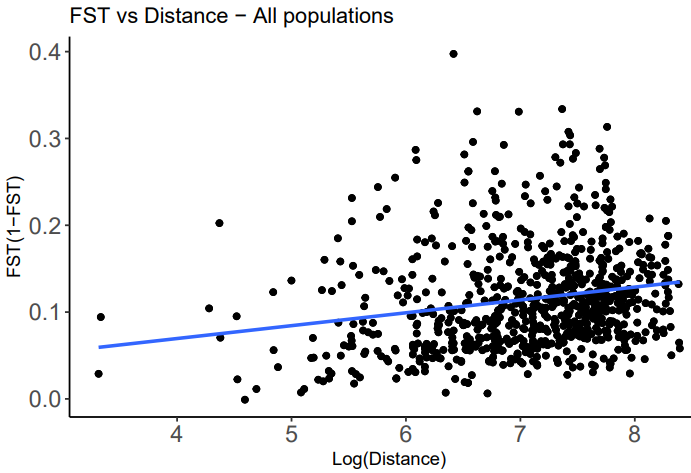
**

A17.B)


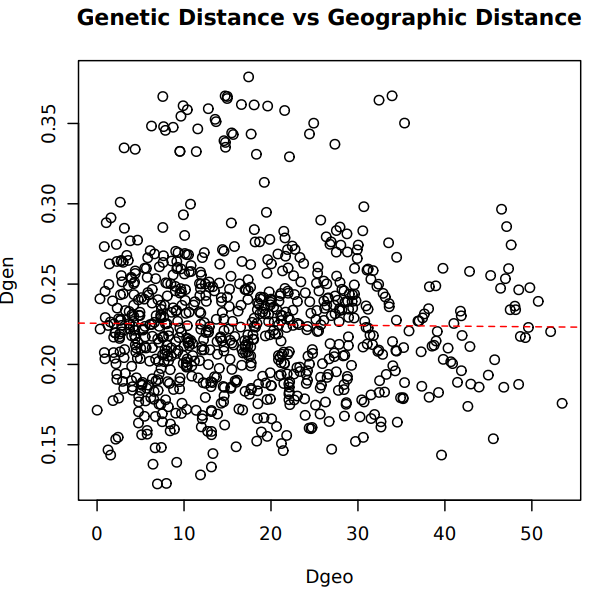


**Figure A17.** Relationships between genetic and geographic distance for European populations using SNP Set 3. Panel A shows FST plotted against log distance (Km). The FST estimates were calculated with the R package “StTAMP” and the linear regression indicated R2=0.04. Panel B shows genetic distance plotted against geographical distance (Km), which was calculated using the R package “geosphere”. The linear regression for this analysis indicated R2=0.0.


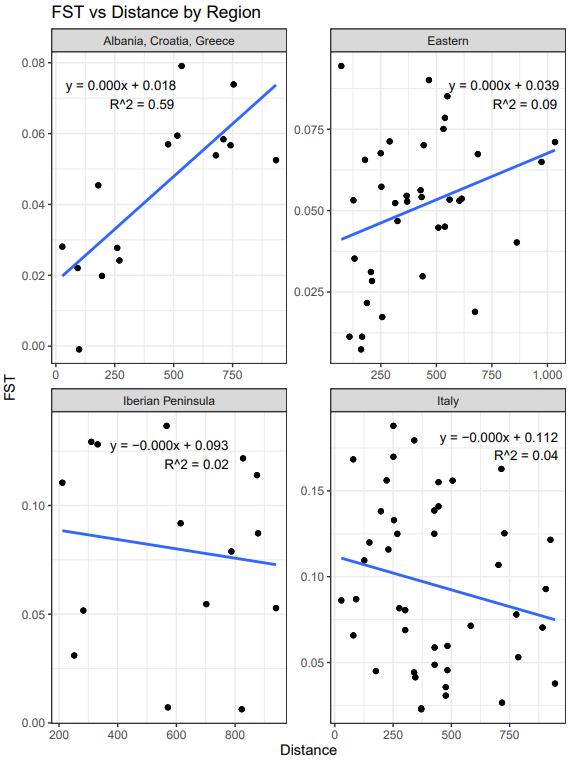


**Figure A18.**  Relationships between genetic and geographic distance for four subsets of European populations using SNP Set 3. Plots shows FST plotted against distance (Km). The FST estimates were calculated with the R package “StTAMP”.

i) ii)

**
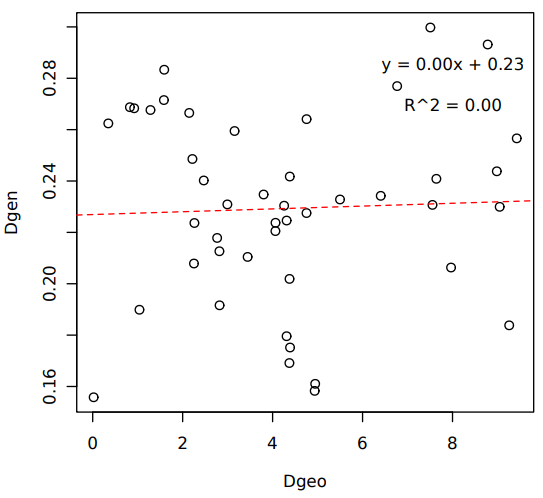

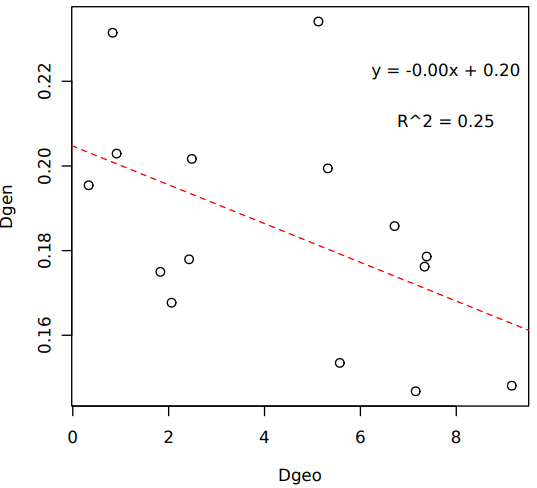
**

iii) iv)


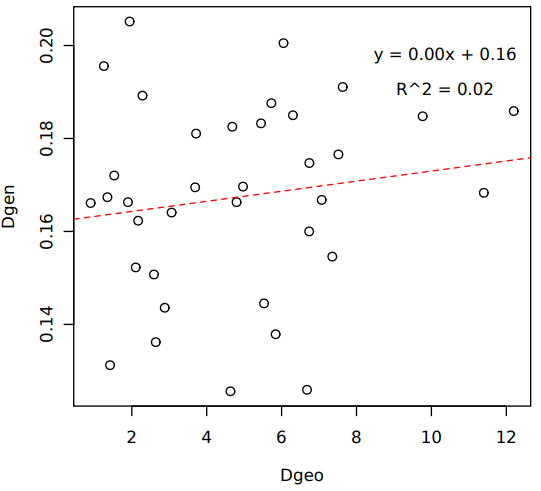

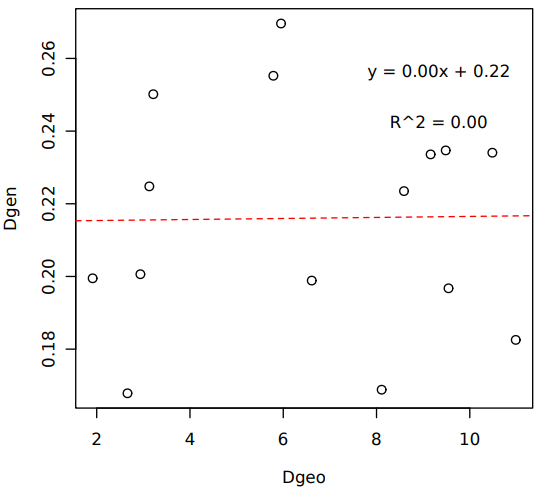


**Figure A19.** Relationships between genetic and geographic distance for four subsets of European populations using SNP Set 3: i) Italy (R^2^=0), ii) Greece, Albania and Croatia (R^2^=-0.25), iii) Eastern Europe (R^2^=0.02), and iv) Iberian Peninsula (R^2^=0). In each plot, the genetic distance is plotted against geographical distance (Km), which was calculated using the R package “geosphere”, and the red line shows the linear regression.

A20.A)


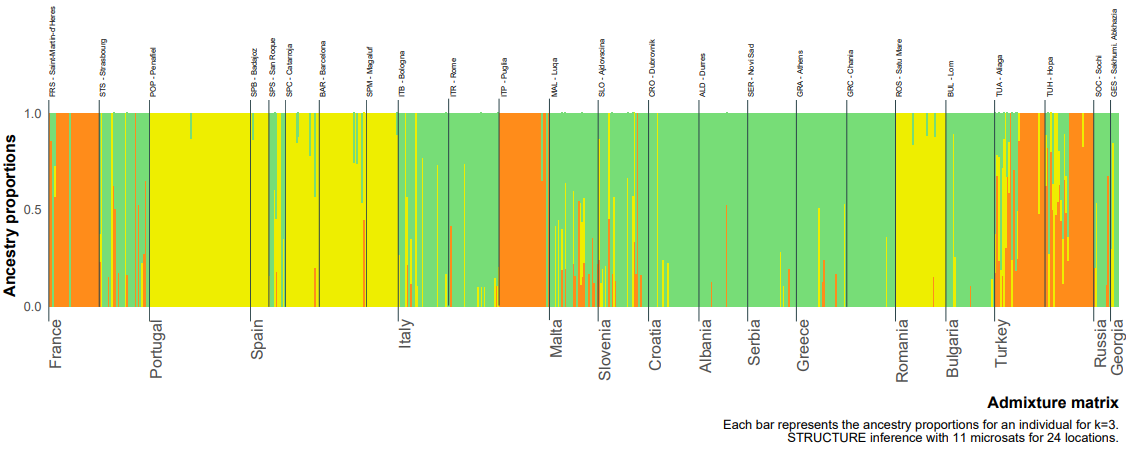


A20.B)


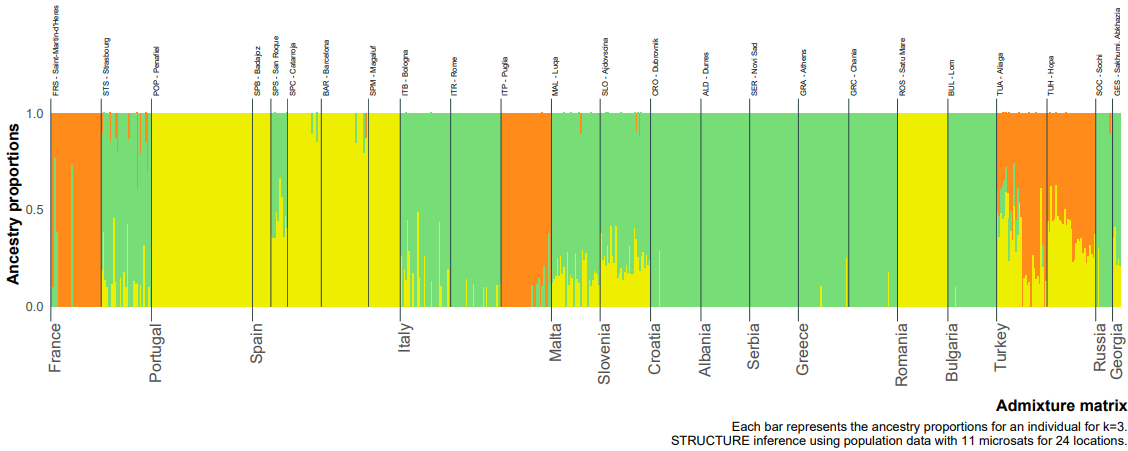


A20.C)


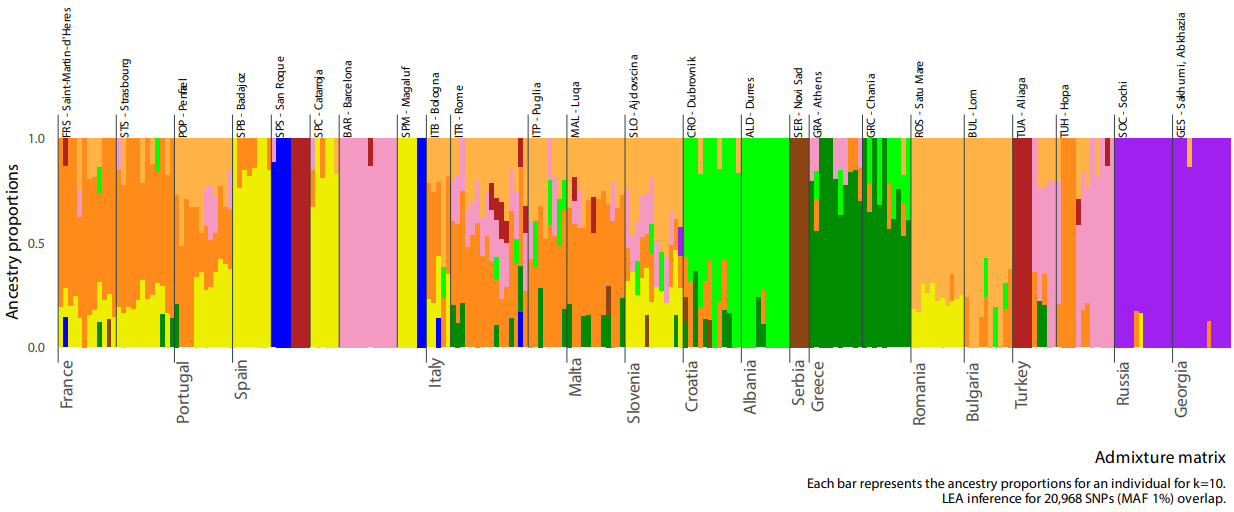


A20.D)


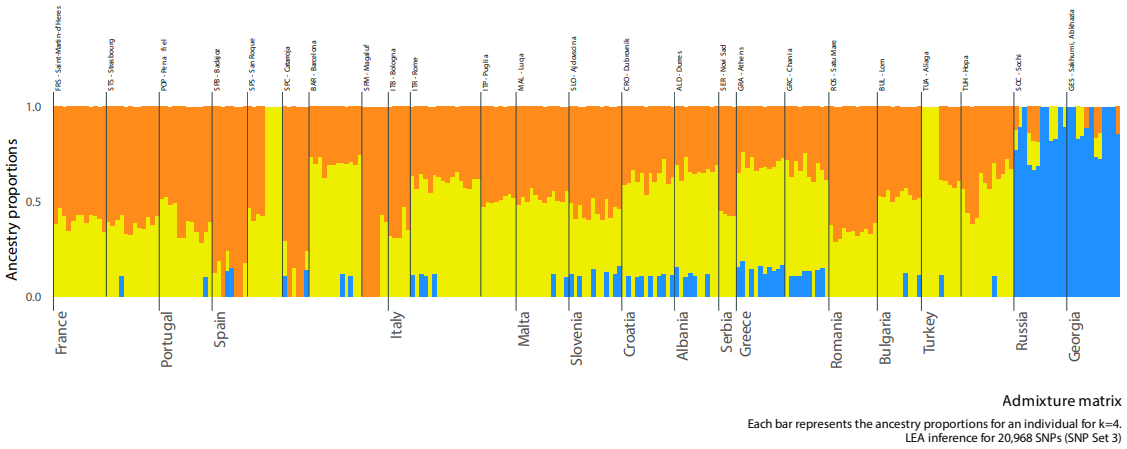


**Figure A20**. Panels A and B show plots of population structure for the microsatellite dataset for 637 mosquitos from the 24 locations that overlap with the SNP dataset. A shows the K=3 clusters found when only genetic information was used in the STRUCTURE analysis, and B shows the K=3 clusters found when both genetic and population information were used in the STRUCTURE analysis. Panels C-D show population structure for 242 mosquitos from the same 24 locations in the European SNP datasets plotted for the best K, K=10 (C) and K=3 (D), to facilitate comparison with the microsatellite results. Structure in Plots A and B were identified in the software STRUCTURE using data from 11 microsatellite loci, and Plots C and D were identified using the LEA package in R on SNP Set 3. In the plots, each vertical bar on the x-axis represents one mosquito and the y-axis shows the proportion of admixture for the ancestral genetic groups for each individual. Plots for the best K obtained from other structure algorithms (fastStructure and admixture) and using the other SNP sets are shown in Supplementary files 16-17 and Table A7.

A21.A)


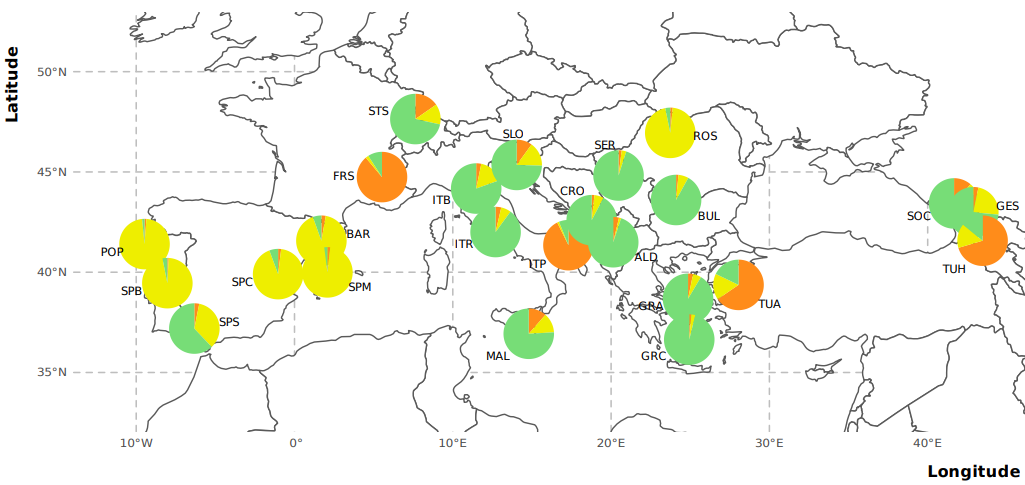


A21.B) **
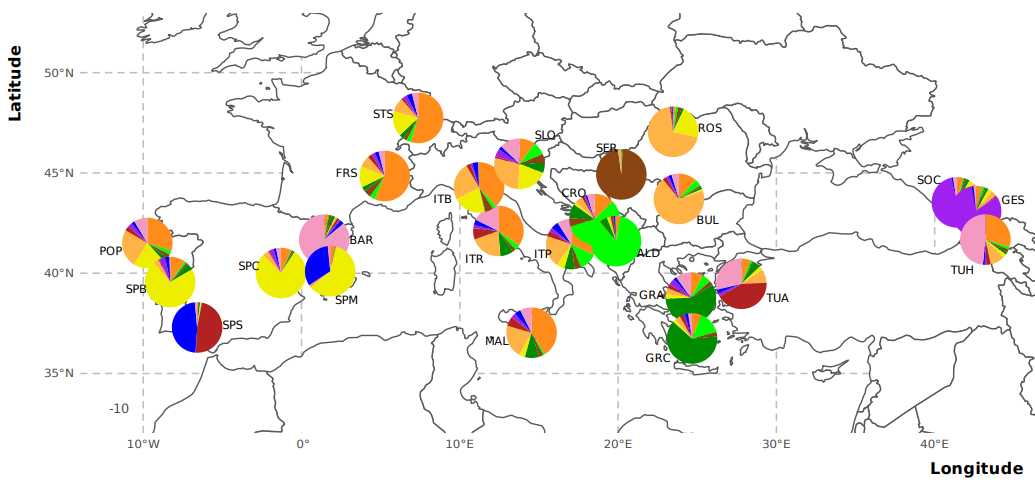
**

**Figure A21.** Maps of the ancestry matrices obtained using STRUCTURE for the microsatellite dataset. Panel A shows results when the STRUCTURE analysis was run using 637 mosquitos from the 24 locations shared with the SNP datasets, using both genetic and location data for inferencing (results from genetic data only model is shown in Figure 5). For comparison, Panel B show the clusters obtained using LEA to analyze structure for the same 24 locations with the SNP Set 3 dataset: B shows K=10, which was the best supported number of clusters. Results for K=3, to facilitate comparison to microsatellites, is shown in Figure 5. For more detailed maps showing all sampling locations in the microsatellite dataset see Supplementary files 11 and 18.

A22.A)


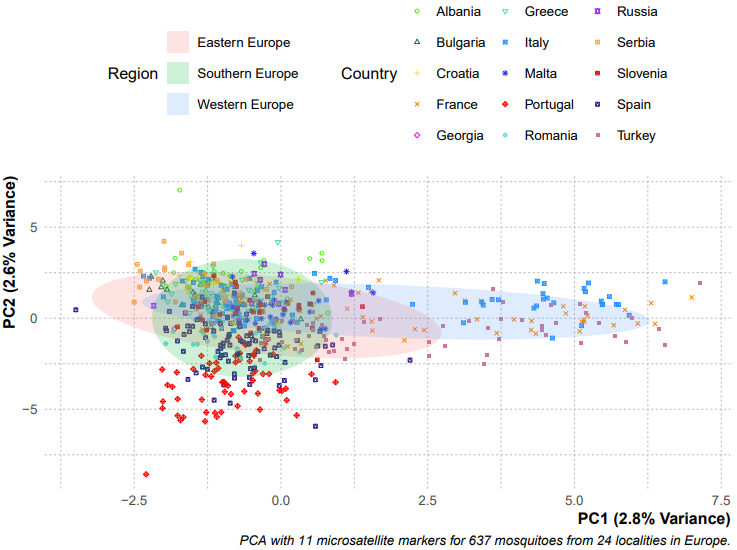


A22.B)


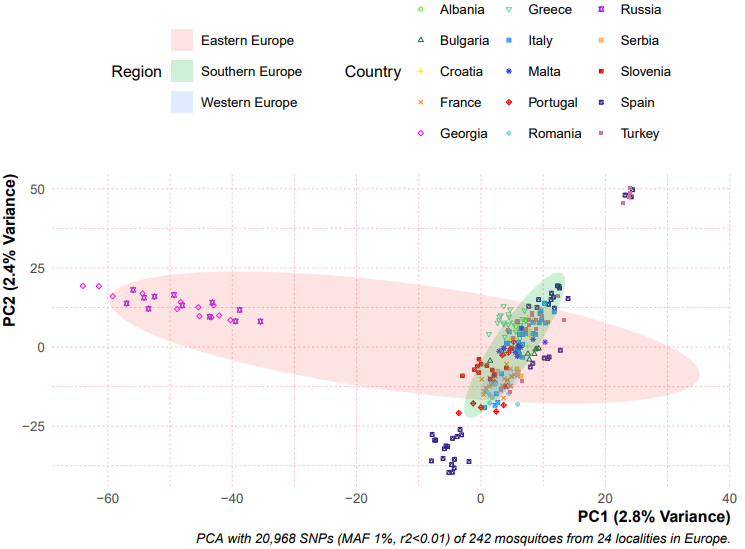


**Figure A22**. Principal component analysis performed on microsatellites datasets in the R package LEA for 637 mosquitos in 24 locations that overlap with the SNP dataset. For comparison, panel B shows the PCA for 242 mosquitos in SNP Set 3 for the same 24 locations shown in Panel B. In each plot the variance explained by each principal component is in parentheses on the y- and x-axes. Each symbol represents a mosquito, and the color and shape of the symbol indicates the country where they were sampled. Ellipses mark each region in Europe covering 80% of the samples. Additional details of the PCA analyses for microsatellites are presented in Supplementary file 11.

A23.A)


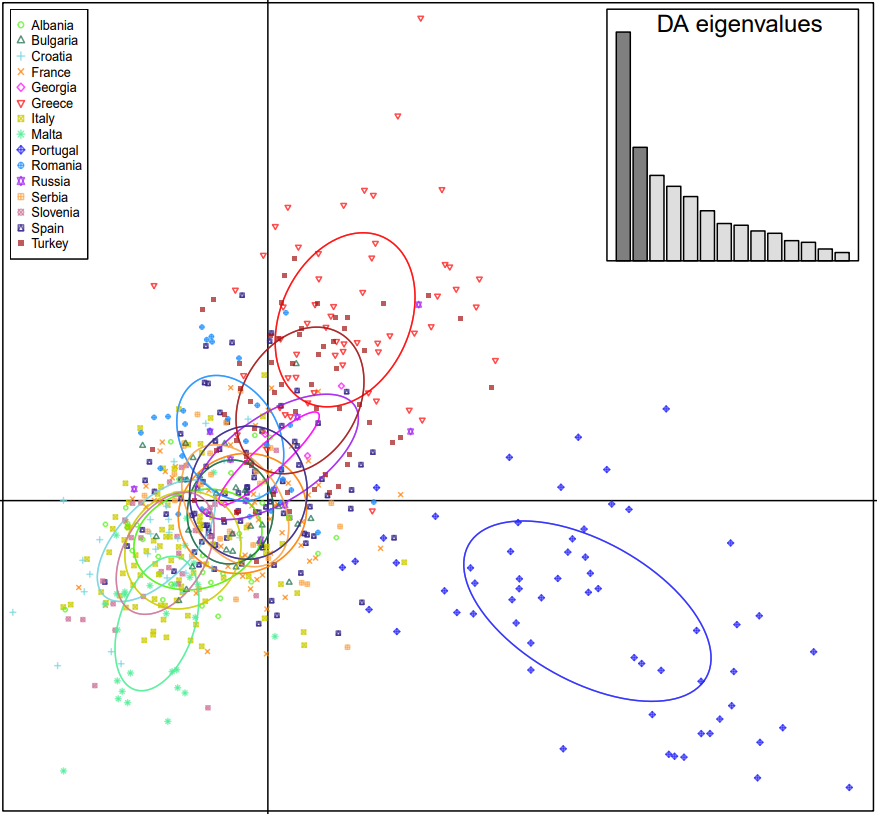


A23.B)


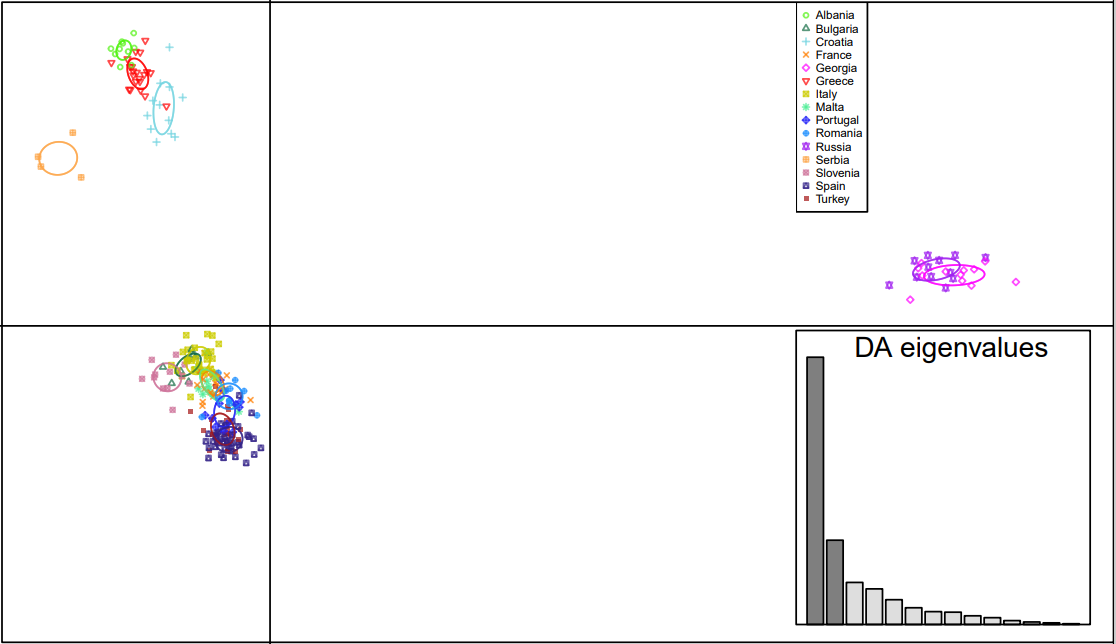


**Figure A23**. Scatterplots of the principal components derived from DAPC for 637 mosquitos in 24 locations in the microsatellite dataset that overlap with the SNP dataset (A). Panel B shows the DAPC for SNP Set 3 for the same 24 locations shown in Panel A, for comparison. Each symbol represents a mosquito, and the color and shape of the symbol indicates the location where they were sampled. Points are plotted according to scores on the discriminant functions, and axes are scaled to the eigenvalues, which represent the relative contribution of each function (shown in the inset bar graph) to the total genetic variance observed. Additional details and plots of the DAPC analyses are presented in Supplementary File S14.

A24.A)


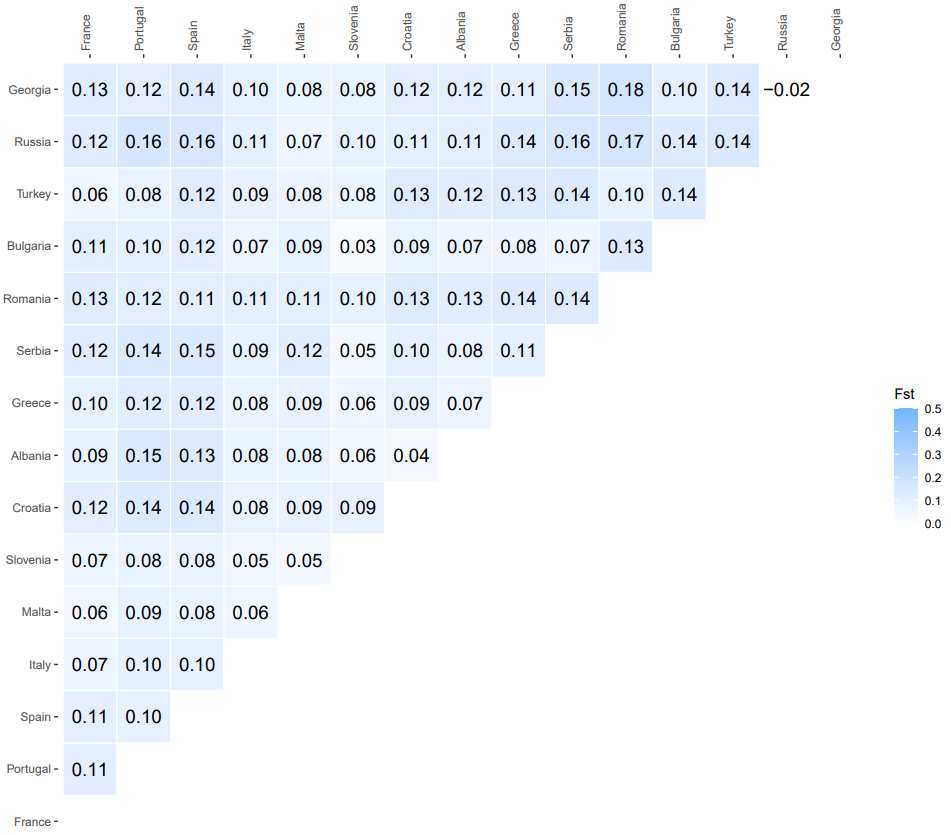


A24.B)


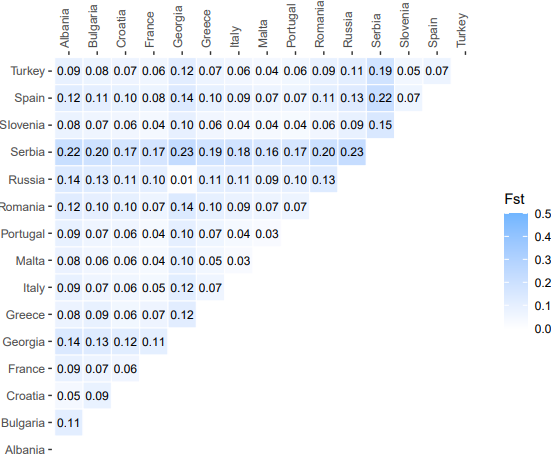


**Figure A24**. Matrix of pairwise FST values calculated with the hierfstat package in R using the dataset with 11 microsatellites. Pairwise FST values for countries were determined by computing the average FST across all populations within a country. Panel A shows the matrix for the 637 mosquitos in 24 locations that overlap with the SNP datasets. For comparison, panel B shows the matrix calculated using SNP Set 3 in the StAMPP package in R for 242 mosquitos the same 24 locations include in the microsatellite dataset. See Supplementary File S20 for additional FST matrices and analyses for microsatellite data.

A25.A)


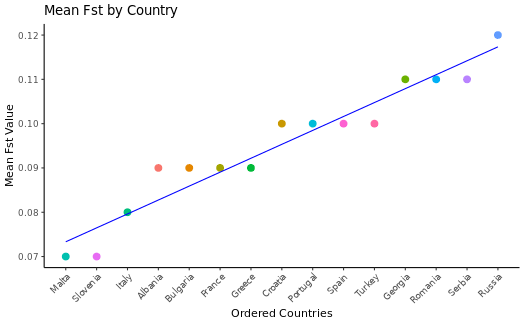


A25.B)


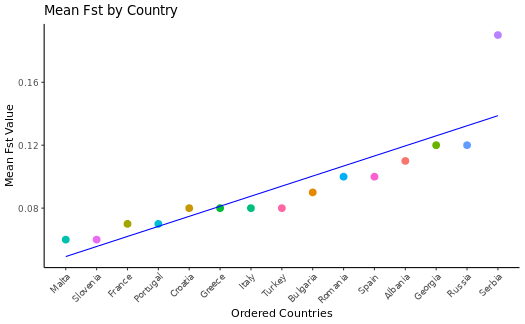


**Figure A25**. Mean Fixation Index (FST; y-axis) for countries in Europe. Data in plot A was computed using 11 microsatellite loci in hierfstat the package in R. B was computed using SNP Set 3 in the StAMPP package in R. Pairwise FST values were determined across all populations within a country, which were then averaged to estimate the mean FST per country. We fit a linear regression line to visualize the trend in mean FST across locations. Countries are sorted from left to right based on ascending mean FST values. Panel A shows FST values calculated with microsatellite data from the 24 locations that overlap with the SNP dataset. B shows FST values calculated with SNP data for the 24 locations that overlap with the microsatellite dataset.

A26.A)


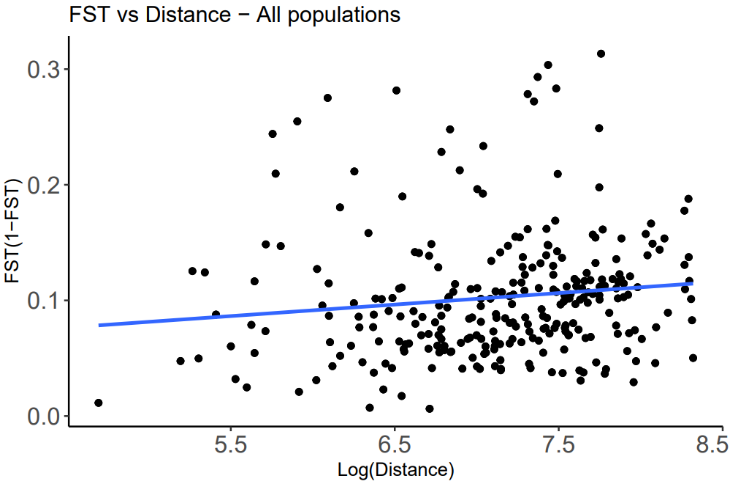

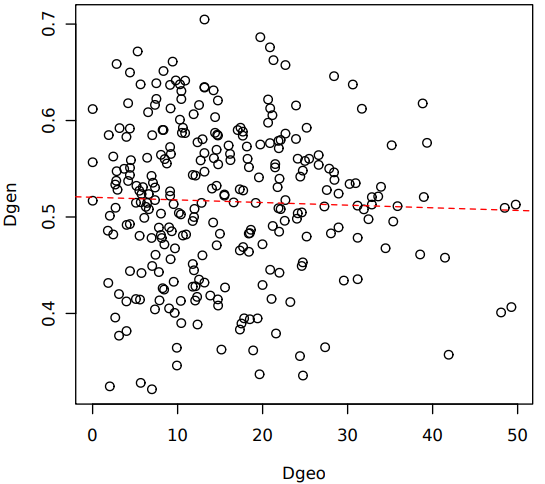


A26.B)


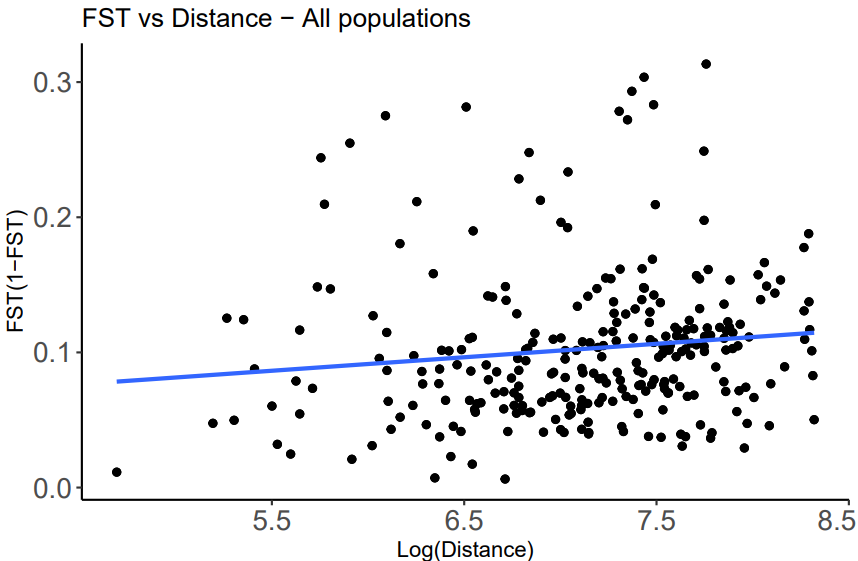

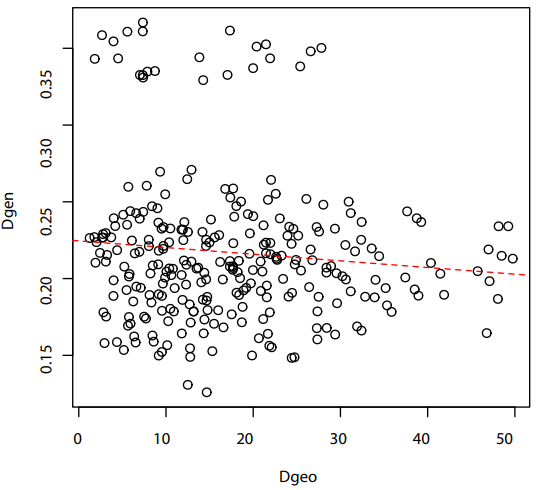


**Figure A26**. Relationship between genetic and geographic distance. In each panel, the plots on the left show the relationship between FST and log geographic distance. The blue line indicates the linear regression that was fit to the data. The plots on the right show the relationship between genetic and geographic distance used in the Mantel test calculations, and the dotted red line indicates the linear regression that was fit to the data. Panel A was calculated using data from 11 microsatellite loci and shows data for the 24 microsatellite locations that overlap with the SNP datasets (FST plot R^2^=0.02; Dgen plot R^2^=0.00). For comparison, panel B shows the data for the same 24 locations calculated from SNP Set 3 (FST plot R^2^=0.02; Dgen plot R^2^=0.01).

A27.A)


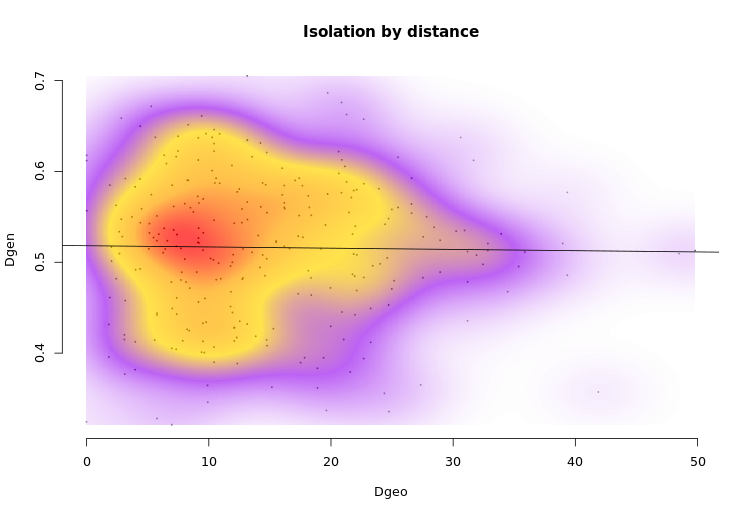


A27.B)


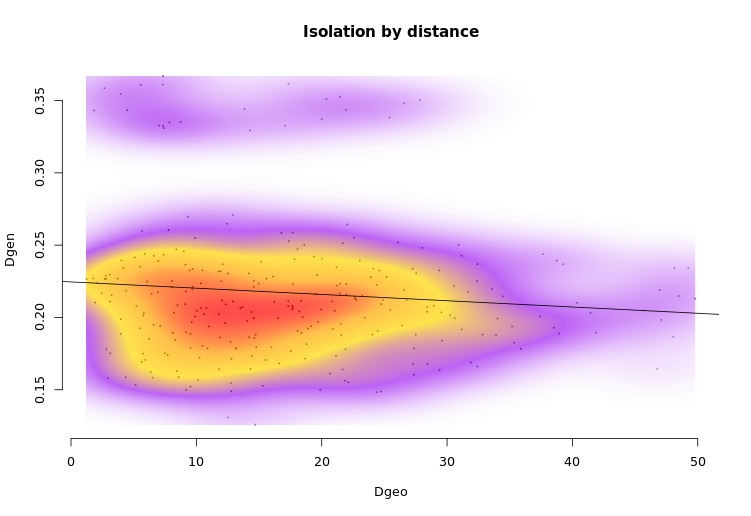


**Figure A27.** Plots and results from the Mantel test for isolation-by-distance (IBD) for European populations with at least four mosquitoes (Table A3). All panels show scatterplots of geographic distance (Dgeo) against genetic distance (Dgen), with a line from the linear regression model fit to each dataset. The density of overlapping points is represented by color, with warmer shades indicating more overlap. Panel A shows data from the 637 mosquitos from the 24 locations in the microsatellite dataset that overlap with the SNP dataset (R^2^=0.00). For comparison, panel B shows the 242 mosquitos from the same 24 locations in the SNP Set 3 dataset R^2^=0.01).

**Appendix Tables**

**Table A1.** List of the supplemental files that outline methods and present additional results. R Markdown files with the step-by-step details of analyses are available at the links provided.

| File | Description of file | Link |
| --- | --- | --- |
| File S1 | Sample providers and collection details for SNPs (Excel) |  |
| File S2 | Sample providers and collection details for microsatellites (Excel) |  |
| File S3 | Supplemental methods (pdf) |  |
| File S4 | Axiom Analysis Suites file for European dataset SNP genotype calls (pdf) |  |
| File S5 | Axiom Analysis Suites file for Global dataset SNP genotype calls (pdf) |  |
| File S6 | Quality control for European SNP dataset (R Markdown) | [Link](https://rpubs.com/margaret_corley/1221965) |
| File S7 | Quality control for Global SNP dataset (R Markdown) | [Link](https://rpubs.com/margaret_corley/1221949) |
| File S8 | Linkage Disequilibrium analyses (R Markdown) | [Link](https://rpubs.com/margaret_corley/1221966) |
| File S9 | European SNP dataset structure analyses (R Markdown) | [Link](https://rpubs.com/margaret_corley/1221971) |
| File S10 | Global SNP dataset structure analyses (R Markdown) | [Link](https://rpubs.com/margaret_corley/1222007) |
| File S11 | Microsatellite PCA and DAPC analyses and figures (R Markdown) | [Link](https://rpubs.com/margaret_corley/1221980) |
| File S12 | PCA and LEA Analyses for European SNP dataset (R Markdown) | [Link](https://rpubs.com/margaret_corley/1221981) |
| File S13 | PCA and LEA Analyses for Global SNP dataset (R Markdown) | [Link](https://rpubs.com/margaret_corley/1221950) |
| File S14 | DAPC for European and Global SNP datasets (R Markdown) | [Link](https://rpubs.com/margaret_corley/1221955) |
| File S15 | Microsatellite STRUCTURE analyses and CLUMPAK results (pdf) |  |
| File S16 | Structure Figures for Europe SNP datasets (R Markdown) | [Link](https://rpubs.com/margaret_corley/1221987) |
| File S17 | Structure Maps for Global SNP dataset (R Markdown) | [Link](https://rpubs.com/margaret_corley/1221964) |
| File S18 | Structure Figures for Microsatellites (R Markdown) | [Link](https://rpubs.com/margaret_corley/1222006) |
| File S19 | FST Analyses for European SNP dataset (R Markdown) | [Link](https://rpubs.com/margaret_corley/1221999) |
| File S20 | FST analyses for Microsatellites (R Markdown) | [Link](https://rpubs.com/margaret_corley/1221992) |

**Table A2.** List of all sampling locations, unique codes assigned to each location (‘Code’), including the year in which samples were collected, and the number of mosquitoes genotyped from each Sampling Site (‘Samples Genotyped’) and passing all quality control filtering (‘Samples passing QC’) for SNP dataset.

| **Region** | **Country** | **Locality** | **Code** | **Year** | **Samples Genotyped** | **Samples passing all QC** |
| --- | --- | --- | --- | --- | --- | --- |
| Western Europe | France | Saint-Martin-d'Hères | FRS | 2019 | 12 | 12 |
| Western Europe | France | Strasbourg | STS | 2019 | 12 | 12 |
| Eastern Europe | Armenia | Ijevan | ARM | 2020 | 10 | 10 |
| Eastern Europe | Bulgaria | Lom | BUL | 2019 | 10 | 10 |
| Eastern Europe | Georgia | Sakhumi, Abkhazia | GES | 2021 | 12 | 12 |
| Eastern Europe | Romania | Satu Mare | ROS | 2020 | 12 | 11 |
| Eastern Europe | Russia | Armavir | RAR | 2021 | 12 | 12 |
| Eastern Europe | Russia | Krasnodar | KRA | 2017 | 12 | 12 |
| Eastern Europe | Russia | Sochi | SOC | 2021 | 12 | 12 |
| Eastern Europe | Russia | Tikhoretsk | TIK | 2021 | 12 | 12 |
| Eastern Europe | Serbia | Novi Sad | SER | 2019 | 4 | 4 |
| Eastern Europe | Turkey | Aliaga | TUA | 2019 | 12 | 9 |
| Eastern Europe | Turkey | Hopa | TUH | 2019 | 12 | 12 |
| Eastern Europe | Ukraine | Alushta | ALU | 2021 | 12 | 12 |
| Eastern Europe | Ukraine | Kerch, Crimea | KER | 2021 | 12 | 12 |
| Eastern Europe | Ukraine | Sevastopol, Crimea | SEV | 2021 | 12 | 12 |
| Southern Europe | Albania | Durres | ALD | 2018 | 10 | 10 |
| Southern Europe | Albania | Tirana | TIR | 2020 | 4 | 4 |
| Southern Europe | Albania | Vlore | ALV | 2020 | 12 | 12 |
| Southern Europe | Croatia | Dubrovnik | CRO | 2017 | 12 | 12 |
| Southern Europe | Greece | Athens | GRA | 2019 | 12 | 11 |
| Southern Europe | Greece | Chania | GRC | 2019 | 14 | 10 |
| Southern Europe | Italy | Bologna | ITB | 2017 | 6 | 5 |
| Southern Europe | Italy | Brescia | BRE | 1995 | 13 | 13 |
| Southern Europe | Italy | Cesena | CES | 1995 | 20 | 14 |
| Southern Europe | Italy | Desenzano | DES | 1995 | 20 | 16 |
| Southern Europe | Italy | Imperia | IMP | 2017 | 4 | 4 |
| Southern Europe | Italy | Puglia | ITP | 2016 | 12 | 9 |
| Southern Europe | Italy | Rome | ITR | 2013 | 12 | 12 |
| Southern Europe | Italy | Rome | ROM | 2017 | 4 | 4 |
| Southern Europe | Italy | Sicilia | SIC | 2016 | 12 | 9 |
| Southern Europe | Italy | Trentino | TRE | 2020 | 12 | 12 |
| Southern Europe | Malta | Luqa | MAL | 2019 | 12 | 12 |
| Southern Europe | Portugal | Loulé* | POL | 2017 | 2 | 2 |
| Southern Europe | Portugal | Penafiel | POP | 2017 | 12 | 12 |
| Southern Europe | Slovenia | Ajdovščina | SLO | 2017 | 12 | 12 |
| Southern Europe | Spain | Badajoz | SPB | 2018 | 8 | 8 |
| Southern Europe | Spain | Barcelona | BAR | 2018 | 12 | 12 |
| Southern Europe | Spain | Catarroja | SPC | 2017 | 6 | 6 |
| Southern Europe | Spain | Magaluf | SPM | 2017 | 7 | 5 |
| Southern Europe | Spain | San Roque | SPS | 2017 | 10 | 8 |
| East Asia | China | Hainan | HAI | 2014 | 12 | 12 |
| East Asia | China | Hunan | HUN | 2001 | 12 | 12 |
| East Asia | China | Yunnan | YUN | 2014 | 9 | 9 |
| East Asia | Japan | Kagoshima | KAG | 2018 | 12 | 12 |
| East Asia | Japan | Kanazana | KAN | 2008 | 12 | 11 |
| East Asia | Japan | Okinawa | OKI | 2018 | 12 | 12 |
| East Asia | Japan | Utsonomyia | UTS | 2008 | 12 | 12 |
| East Asia | Taiwan | Tainan | TAI | 2018 | 8 | 7 |
| South Asia | Buthan | Gelephu | GEL | 2009 | 2 | 2 |
| South Asia | India | Bengaluru | BEN |  | 12 | 12 |
| South Asia | Nepal | Kathmandu | KAT | 2002 | 12 | 6 |
| South Asia | Sri Lanka | Jaffna | JAF |  | 2 | 2 |
| Southeast Asia | Cambodia | Cambodia | CAM |  | 12 | 12 |
| Southeast Asia | Indonesia | Jakarta, Indonesia | INJ |  | 12 | 11 |
| Southeast Asia | Indonesia | Sulawesi (Forest) | SUF |  | 6 | 6 |
| Southeast Asia | Indonesia | Sulawesi (Urban) | SUU |  | 6 | 6 |
| Southeast Asia | Indonesia | Wainyapu, Indonesia | INW |  | 8 | 4 |
| Southeast Asia | Malaysia | Kuala Lumpur | KLP |  | 4 | 4 |
| Southeast Asia | Malaysia | Tambun | MAT |  | 12 | 12 |
| Southeast Asia | Maldives | Kunfunadhoo | KUN |  | 4 | 4 |
| Southeast Asia | Thailand | Chanthaburi | CHA |  | 12 | 12 |
| Southeast Asia | Thailand | Kanchanaburi | KAC | 2015 | 12 | 6 |
| Southeast Asia | Thailand | Lampang | LAM | 2011 | 10 | 9 |
| Southeast Asia | Thailand | Si Sa Ket | SSK | 2018 | 12 | 12 |
| Southeast Asia | Thailand | Songkhla | SON | 2015 | 5 | 3 |
| Southeast Asia | Vietnam | Hanoi | HAN |  | 4 | 4 |
| Southeast Asia | Vietnam | Ho Chi Min | HOC | 2018 | 12 | 7 |
| Southeast Asia | Vietnam | Qhy Nhon City | QNC |  | 12 | 11 |
| North America | USA | Berlin, NJ | BER | 2018 | 12 | 12 |
| North America | USA | Palm Beach, FL | PAL | 2018 | 12 | 11 |
| South America | Brazil | Gravatai | GRV | 2018 | 12 | 12 |
| South America | Brazil | Recife | REC | 2017 | 12 | 11 |
| **Total:** |  |  |  |  | **748** | **688** |

*Since only two individuals were genotyped in this population, it was excluded from F_ST_ analyses and comparisons of the SNPs and microsatellite datasets.

**Table A3.** List of all sampling locations, unique codes assigned to each location (‘Code’), including the year in which samples were collected, and the number of mosquitoes genotyped from each Sampling Site (‘Samples Genotyped’) for microsatellite dataset.

| **Region** | **Country** | **Locality** | **Code** | **Year** | **N Genotyped** | **Collection method** |
| --- | --- | --- | --- | --- | --- | --- |
| Western Europe | France | Saint-Martin-d'Hères | FRS | 2019 | 30 | Human bait |
| Western Europe | France | Strasbourg | STS | 2019 | 30 | Human bait |
| Eastern Europe | Bulgaria | Lom | BUL | 2019 | 29 | Ovitrap |
| Eastern Europe | Georgia | Sakhumi, Abkhazia | ABSU | 2018 | 5 | Human bait |
| Eastern Europe | Romania | Satu Mare | ROS | 2020 | 30 | Human bait |
| Eastern Europe | Russia | Sochi | SOC | 2018 | 10 | Human bait |
| Eastern Europe | Serbia | Novi Sad | SER | 2019 | 29 | Ovitrap |
| Eastern Europe | Turkey | Aliaga | TUA | 2019 | 30 | Larval dipping |
| Eastern Europe | Turkey | Hopa | TUH | 2019 | 29 | Ovitrap |
| Southern Europe | Albania | Durres | ALD | 2018 | 29 | Ovitrap |
| Southern Europe | Croatia | Dubrovnik | CRO | 2017 | 30 | Larval dipping |
| Southern Europe | Greece | Athens | GRA | 2019 | 30 | Ovitrap |
| Southern Europe | Greece | Chania | GRC | 2019 | 29 | Ovitrap |
| Southern Europe | Italy | Bologna | ITB |  | 30 | Ovitrap |
| Southern Europe | Italy | Puglia | ITP | 2016 | 30 | Ovitrap |
| Southern Europe | Italy | Rome | ITRO | 2016 | 30 | Ovitrap |
| Southern Europe | Malta | Luqa | MAL | 2019 | 29 | Baited trap |
| Southern Europe | Portugal | Loulé* | POL | 2017 | 15 | Human bait |
| Southern Europe | Portugal | Penafiel | POP | 2017 | 60 | Human bait |
| Southern Europe | Slovenia | Ajdovščina | SLO | 2017 | 30 | Human bait |
| Southern Europe | Spain | Badajoz | SPB | 2018 | 11 | Ovitrap |
| Southern Europe | Spain | Catarroja | SPC | 2017 | 20 | Larval dipping |
| Southern Europe | Spain | Cornellà del Llobregat | ESBA | 2017 | 28 | Larval dipping |
| Southern Europe | Spain | Magaluf | SPM | 2017 | 19 | Ovitrap |
| Southern Europe | Spain | San Roque | SPS | 2017 | 10 | Ovitrap |
| **Total:** |  |  |  |  | **652** |  |

*This population was excluded from comparisons of the SNPs and microsatellite datasets, since only two individuals from this location were genotyped in the SNP dataset.

**Table A4**. Number of individuals (N) and number of variants in each SNP Set that passed quality control and were included in subsequent analyses for the Europe and Global datasets. The first two SNP sets were filtered for linkage disequilibrium (LD) thresholds, r^2^ < 0.01 (Set 1) and r^2^ < 0.1 (Set 2) respectively, to assess the potential influence that LD filtering had on our results. In both Sets 1 and 2 variants with a minor allele frequency (MAF) of < 10% were removed. Set 3 was filtered for linkage at r^2^ < 0.01 and variants with MAF < 1% were removed. Analyses were performed with all three sets, but only the results for SNP Set 3 are reported in the main text. For comparison, select results for Sets 1 and 2, which are not reported in the main text, can be found in the Supplementary files.

| **Dataset** | **Set 1**  **N** | **Set 1 SNPs**  **(LD r^2^ <0.01)** | **Set 2**  **N** | **Set 2 SNPs**  **(LD r^2^ <0.1)** | **Set 3**  **N** | **Set 3 SNPs**  **(MAF 1%)** |
| --- | --- | --- | --- | --- | --- | --- |
| Europe | 409 | 17,028 | 409 | 47,484 | 410 | 20,968 |
| Global | 688 | 19,318 | 688 | 56,384 | 688 | 22,642 |

**Table A5.** Calculated SNP density for each of the three chromosomes after filtering SNPs for missingness and minor allele frequency MAF<10% (A, C) and MAF <1% (B, D). The number of SNPs per chromosome (N) and per 1Mb window, as well as the number of windows for each chromosome are shown for European dataset samples (A & B) and the Global dataset samples (C & D) after quality control.

*Europe*

A)

| **Chromosome** | **SNPs (N)** | **SNPs per 1Mb window** | **Number of windows** |
| --- | --- | --- | --- |
| 1 | 18,957 | 51.80 | 366 |
| 2 | 35,754 | 61.75 | 579 |
| 3 | 30,595 | 62.69 | 488 |

B)

| **Chromosome** | **SNPs (N)** | **SNPs per 1Mb window** | **Number of windows** |
| --- | --- | --- | --- |
| 1 | 22,358 | 60.43 | 370 |
| 2 | 42,076 | 72.42 | 581 |
| 3 | 36,013 | 73.80 | 488 |

*Global*

C)

| **Chromosome** | **SNPs (N)** | **SNPs per 1Mb window** | **Number of windows** |
| --- | --- | --- | --- |
| 1 | 19,362 | 52.76 | 367 |
| 2 | 36,611 | 63.34 | 578 |
| 3 | 31,210 | 63.82 | 489 |

D)

| **Chromosome** | **SNPs (N)** | **SNPs per 1Mb window** | **Number of windows** |
| --- | --- | --- | --- |
| 1 | 22,313 | 60.47 | 369 |
| 2 | 42,102 | 72.46 | 581 |
| 3 | 35,952 | 73.52 | 489 |

**Table A6.** Summary of algorithms and parameters used for analyses of population structure for Europe and Global SNP datasets. Admixture: *“run”* specifies 5 computational runs, *“--cv”* enables cross-validation for model assessment, *“-B”* sets the number of bootstrap replicates for robust estimates, *“-j”* defines the number of threads for computation. LEA: *“repetitions = 5”* sets the number of times to repeat the analysis, *“CPU=10”* allocates ten processing threads to computation, *“entropy=TRUE”* enables the calculation of entropy, providing an additional measure of genetic diversity or information, *“percentage=0.25”*= specifies 25% of the genotypes are masked when computing the cross-entropy criterion, “*iterations = 500*” sets the maximum number of iterations performed to 500. fastStructure: *“100 runs”* repeats the analysis 100 times for better reliability, *“--prior=simple”* sets the simple prior, affecting how the model handles ancestry estimates; *“--full”* computes a full model, rather than a simplified version; *“--cv=10”* enables 10-fold cross-validation for assessing the model's generalizability; *“--tol=10e-6”* sets the tolerance level for convergence at 10^-6, ensuring that the algorithm stops when the estimates are sufficiently accurate.

| **Algorithm** | **K tested** | **Parameters** |
| --- | --- | --- |
| Admixture | 1:30 | 5 run, --cv=10, -B2000, -j20 |
| LEA | 1:30 | repetitions = 5, CPU=10, entropy=TRUE, percentage = 0.25, iterations = 500 |
| fastStructure | 1:40 | 100 runs, --prior=simple --full --cv=10 --tol=10e-6 |
|  |  |  |

**Table A7.** Summary of results from population structure analyses for each algorithm run on the *Ae. albopictus* SNP datasets using the parameters outlined in Table A6.

| **dataset** | **Structure algorithm** | **# of runs** | **Method for choosing K** | **SNP Set** | **Best K** |
| --- | --- | --- | --- | --- | --- |
| **Europe** | fastStructure | 100 | fastStructure -python -m chooseK function | 1 | 15 |
|  |  |  |  | 2 | 15 |
|  |  |  |  | 3 | 18 |
|  | admixture | 5 | lowest CV error | 1 | 15 |
|  |  |  |  | 2 | 13 |
|  |  |  |  | 3 | 13 |
|  | LEA | 5 | cross-entropy criterion; "elbow" in plot | 1 | 15 |
|  |  |  |  | 2 | 15 |
|  |  |  |  | 3 | 14 |
| **Global** | fastStructure | 100 | fastStructure -python -m chooseK function | 3 | 23 |
|  | admixture | 5 | lowest CV error | 3 | 21 |
|  | LEA | 5 | cross-entropy criterion; "elbow" in plot | 3 | 20-22 |

**Table A8.** Mean FST values for all European locations in the SNP dataset with at least four individuals (N=40). Values were computed using SNP Set 3 in the package StAMPP in R. Mean FST values for regions and countries were determined by computing the average FST across all populations within that region or country.

| **Region** | **Mean Fst by region** | **Country** | **Mean Fst by country** | **Location** | **Code** | **Mean Fst** |
| --- | --- | --- | --- | --- | --- | --- |
| Eastern Europe | 0.11 | Armenia | 0.08 | Ijevan | ARM | 0.08 |
|  |  | Bulgaria | 0.10 | Lom | BUL | 0.10 |
|  |  | Georgia | 0.11 | Sakhumi, Abkhazia | GES | 0.11 |
|  |  | Romania | 0.11 | Satu Mare | ROS | 0.11 |
|  |  | Russia | 0.11 | Armavir | RAR | 0.12 |
|  |  |  |  | Krasnodar | KRA | 0.09 |
|  |  |  |  | Sochi | SOC | 0.11 |
|  |  |  |  | Tikhoretsk | TIK | 0.13 |
|  |  | Serbia | 0.20 | Novi Sad | SER | 0.20 |
|  |  | Turkey | 0.09 | Aliaga | TUA | 0.09 |
|  |  |  |  | Hopa | TUH | 0.08 |
|  |  | Ukraine | 0.11 | Alushta | ALU | 0.10 |
|  |  |  |  | Kerch, Crimea | KER | 0.09 |
|  |  |  |  | Sevastopol, Crimea | SEV | 0.13 |
| Southern Europe | 0.10 | Albania | 0.10 | Durres | ALD | 0.11 |
|  |  |  |  | Tirana | TIR | 0.10 |
|  |  |  |  | Vlore | ALV | 0.09 |
|  |  | Croatia | 0.09 | Dubrovnik | CRO | 0.09 |
|  |  | Greece | 0.09 | Athens | GRA | 0.09 |
|  |  |  |  | Chania | GRC | 0.09 |
|  |  | Italy | 0.10 | Bologna | ITB | 0.10 |
|  |  |  |  | Brescia | BRE | 0.15 |
|  |  |  |  | Cesena | CES | 0.18 |
|  |  |  |  | Desenzano | DES | 0.13 |
|  |  |  |  | Imperia | IMP | 0.09 |
|  |  |  |  | Puglia | ITP | 0.07 |
|  |  |  |  | Rome (Sapienza) | ROM | 0.08 |
|  |  |  |  | Rome (Trappola) | ITR | 0.07 |
|  |  |  |  | Sicilia | SIC | 0.09 |
|  |  |  |  | Trentino | TRE | 0.06 |
|  |  | Malta | 0.07 | Luqa | MAL | 0.07 |
|  |  | Portugal | 0.07 | Penafiel | POP | 0.07 |
|  |  | Slovenia | 0.07 | Ajdovscina | SLO | 0.07 |
|  |  | Spain | 0.10 | Badajoz | SPB | 0.09 |
|  |  |  |  | Barcelona | BAR | 0.11 |
|  |  |  |  | Catarroja | SPC | 0.10 |
|  |  |  |  | Magaluf | SPM | 0.09 |
|  |  |  |  | San Roque | SPS | 0.12 |
| Western Europe | 0.08 | France | 0.08 | Saint-Martin-d'Heres | FRS | 0.08 |
|  |  |  |  | Strasbourg | STS | 0.08 |

**Table A9**. Mean FST values for European locations with at least four individuals that were sampled in both the SNP and microsatellite datasets (N=24). Values were computed in R using the package StAMPP for SNP Set 3 and the package hierfstat for microsatellite loci. Mean FST values for regions and countries were determined by computing the average FST across all populations within that region or country.

| **Region** | **Mean Fst by region**  **SNPs** | **Mean Fst by region**  **microsats** | **Country** | **Mean Fst by country**  **SNPs** | **Mean Fst by country microsats** |
| --- | --- | --- | --- | --- | --- |
| Eastern Europe | 0.11 | 0.10 | Bulgaria | 0.09 | 0.09 |
|  |  |  | Georgia | 0.12 | 0.11 |
|  |  |  | Romania | 0.10 | 0.11 |
|  |  |  | Russia | 0.12 | 0.12 |
|  |  |  | Serbia | 0.19 | 0.11 |
|  |  |  | Turkey | 0.08 | 0.10 |
| Southern Europe | 0.08 | 0.09 | Albania | 0.11 | 0.09 |
|  |  |  | Croatia | 0.08 | 0.10 |
|  |  |  | Greece | 0.08 | 0.09 |
|  |  |  | Italy | 0.08 | 0.08 |
|  |  |  | Malta | 0.06 | 0.07 |
|  |  |  | Portugal | 0.07 | 0.10 |
|  |  |  | Slovenia | 0.06 | 0.07 |
|  |  |  | Spain | 0.10 | 0.10 |
| Western Europe | 0.07 | 0.09 | France | 0.07 | 0.09 |
