# Supplementary material for: Population Structure of the Invasive Asian Tiger Mosquito, Aedes albopictus, in Europe
Source: Ecol Evol. 2025 Mar 7;15(3):e71009. doi: 10.1002/ece3.71009 (PMC11886418; doi:10.1002/ece3.71009)
Supplement: Supplementary file 4 — File S15. [file ECE3-15-e71009-s001.pdf]

Supplementary File 15

Results from STRUCTURE v 2.3.4 run using an admixture model utilizing only genetic data (NOT population information) to clusters data. Ten repetitions for each K (2-20) were run for the subset of 637 mosquitoes from 24 locations that overlapped with the SNP dataset in Europe using 11 microsatellites.

The admixture plots shown below were created with the CLUMPAK main pipeline. The major mode for each K is shown first, followed by any minor modes for each, and then a summary of the division of runs by mode is provided. Finally, the results of 'Estimating of the Best K' using the Evanno method from CLUMPAK are shown. Additional plots of the major mode for the best K were created in Supplementary File 21.

References:

Evanno, G., Regnaut, S., & Goudet, J. (2005). Detecting the number of clusters of individuals using the software STRUCTURE: a simulation study. *Molecular Ecology*, *14*(8): 2611-2620, doi: https://doi.org/10.1111/j.1365-294X.2005.02553.x

Kopelman, Naama M; Mayzel, Jonathan; Jakobsson, Mattias; Rosenberg, Noah A; Mayrose, Itay. (2015). **CLUMPAK:** a program for identifying clustering modes and packaging population structure inferences across K. *Molecular Ecology Resources* 15(5): 1179-1191, doi: 10.1111/1755-0998.12387

Prof. Itay Mayrose Lab - Plant Evolution, bioinformatics, & comparative genomics

CLUMPAK - CLUSTER MARKOV PACKAGER ACROSS *K*

Job type: *Distruct for many K's*

Output files:

K=2 albo_microsats_overlap_nopops_run_1_f

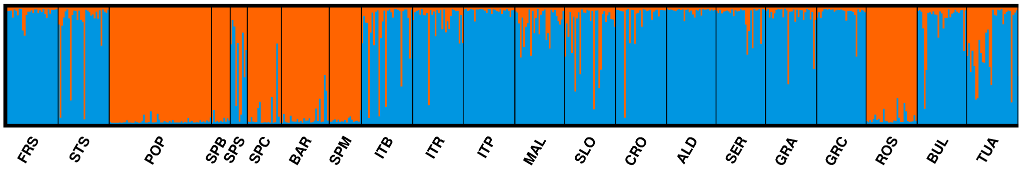

K=2 albo_microsats_overlap_nopops_run_10_f

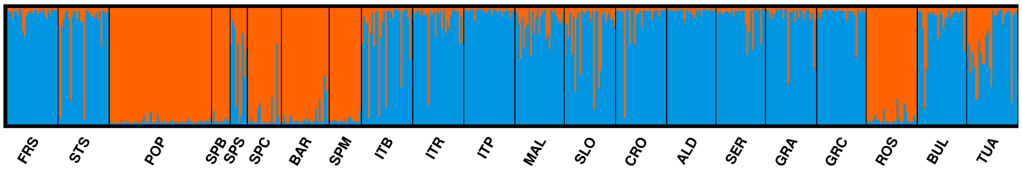

K=2 albo_microsats_overlap_nopops_run_2_f

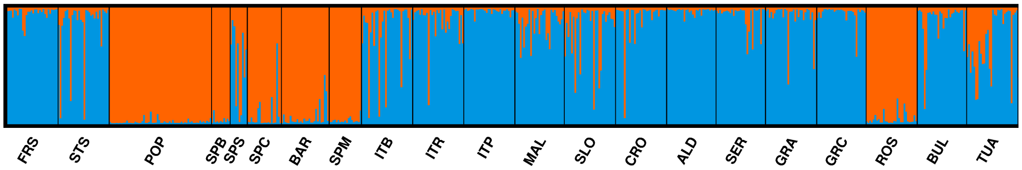

K=2 albo_microsats_overlap_nopops_run_3_f

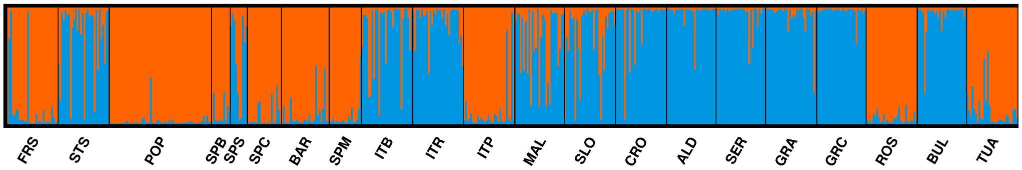

K=2 albo_microsats_overlap_nopops_run_4_f

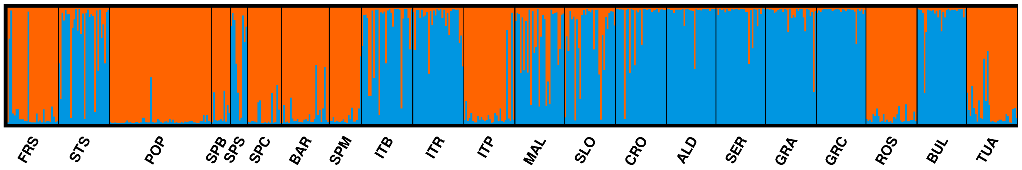

K=2 albo_microsats_overlap_nopops_run_5_f

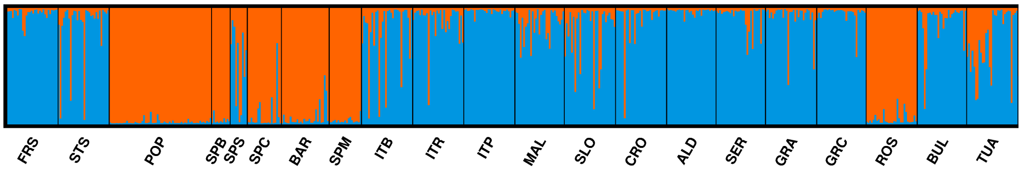

K=2 albo_microsats_overlap_nopops_run_6_f

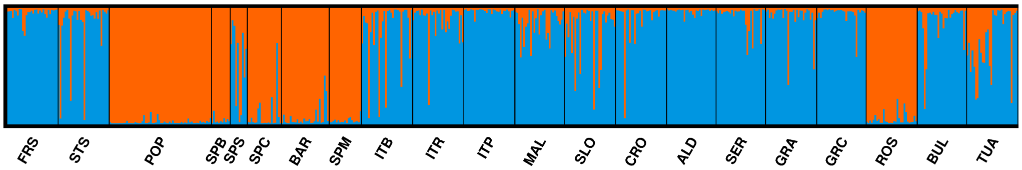

K=2 albo_microsats_overlap_nopops_run_7_f

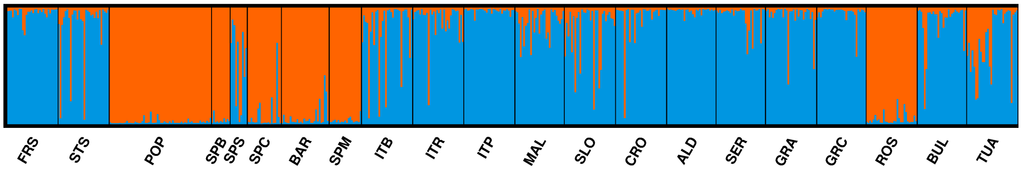

K=2 albo_microsats_overlap_nopops_run_8_f

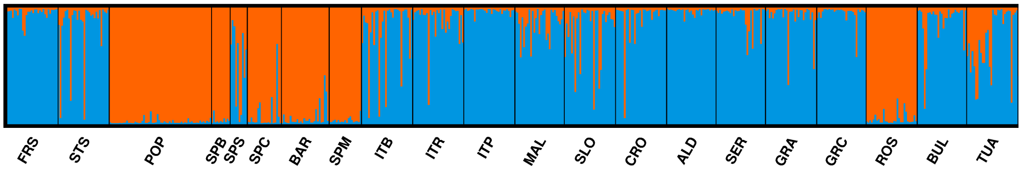

K=2 albo_microsats_overlap_nopops_run_9_f

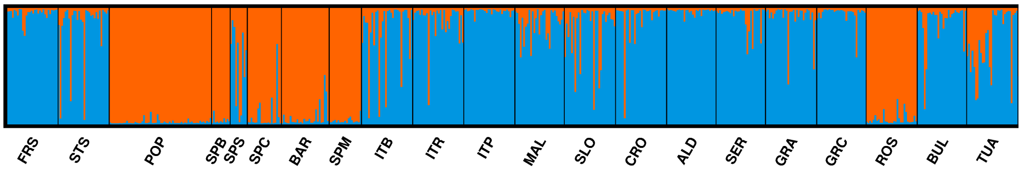

K=3 albo_microsats_overlap_nopops_run_11_f

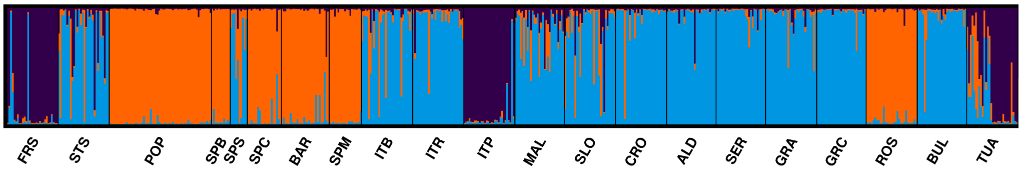

K=3 albo_microsats_overlap_nopops_run_12_f

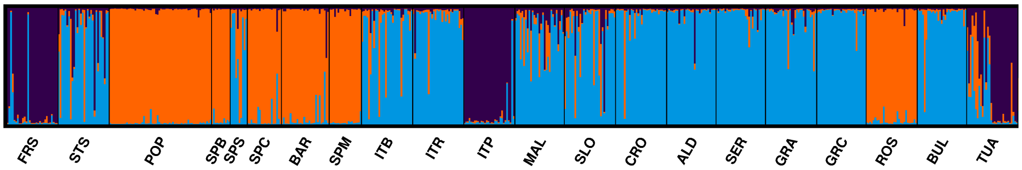

K=3 albo_microsats_overlap_nopops_run_13_f

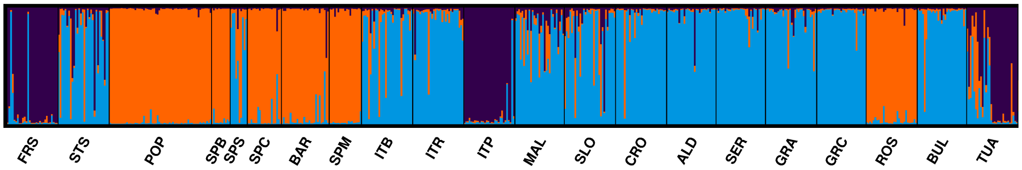

K=3 albo_microsats_overlap_nopops_run_14_f

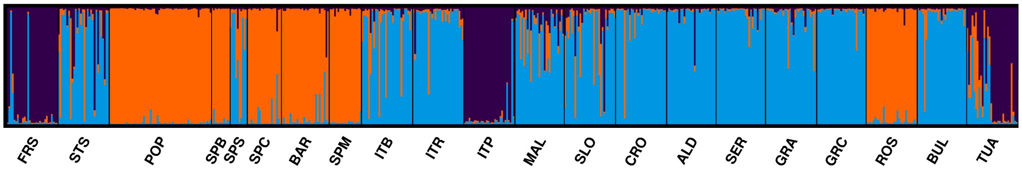

K=3 albo_microsats_overlap_nopops_run_15_f

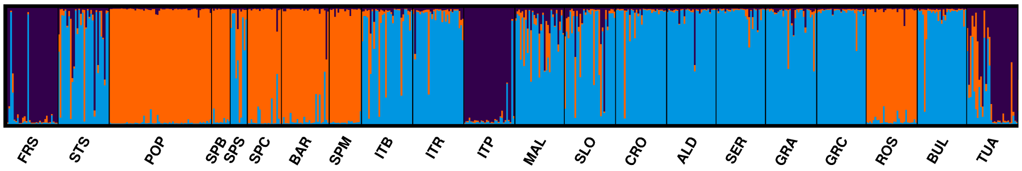

K=3 albo_microsats_overlap_nopops_run_16_f

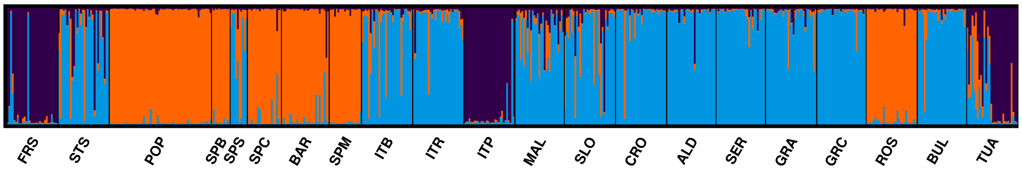

K=3 albo_microsats_overlap_nopops_run_17_f

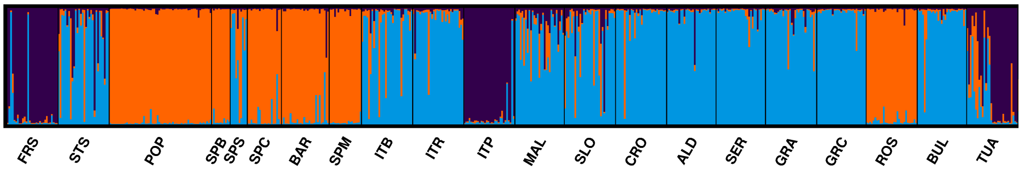

K=3 albo_microsats_overlap_nopops_run_18_f

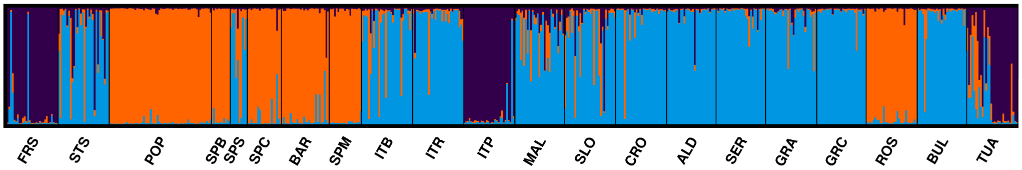

K=3 albo_microsats_overlap_nopops_run_19_f

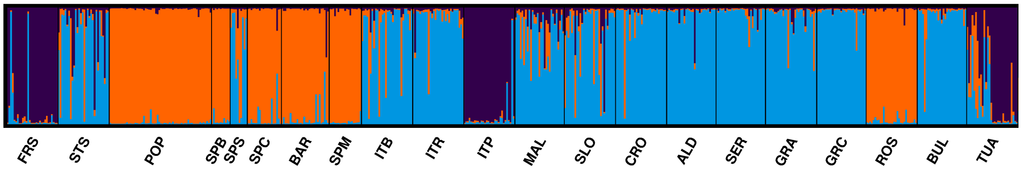

K=3 albo_microsats_overlap_nopops_run_20_f

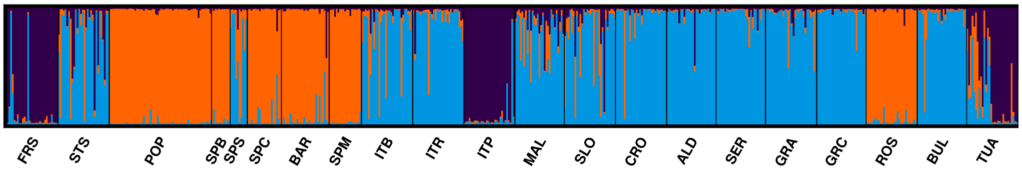

K=4 albo_microsats_overlap_nopops_run_21_f

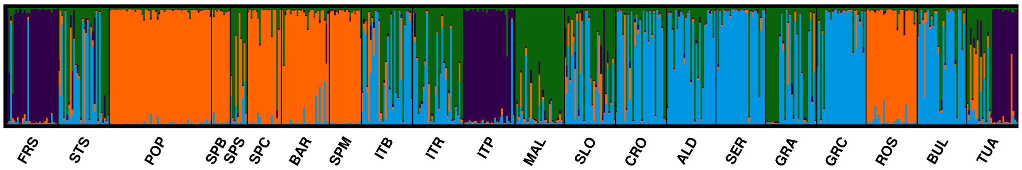

K=4 albo_microsats_overlap_nopops_run_22_f

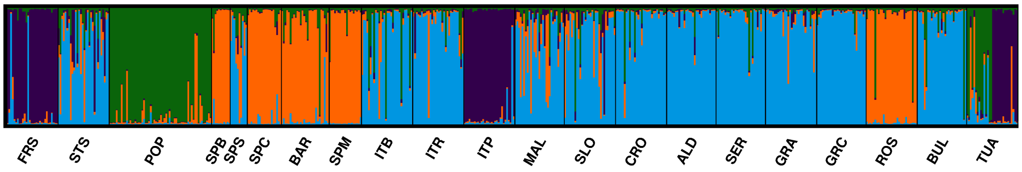

K=4 albo_microsats_overlap_nopops_run_23_f

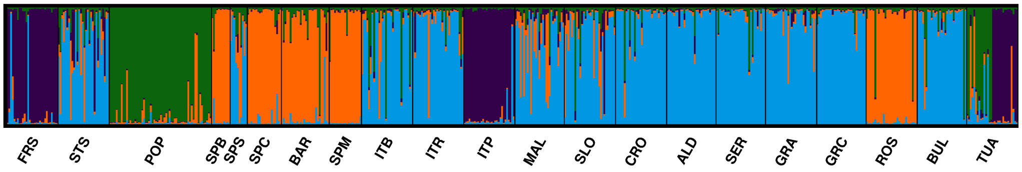

K=4 albo_microsats_overlap_nopops_run_24_f

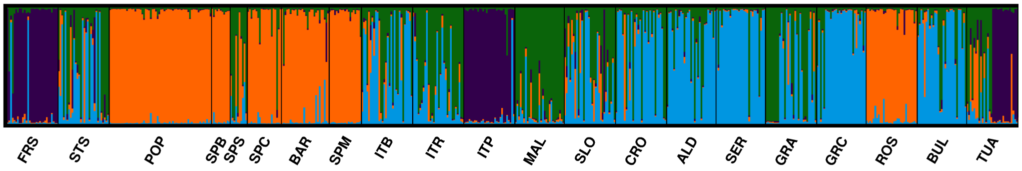

K=4 albo_microsats_overlap_nopops_run_25_f

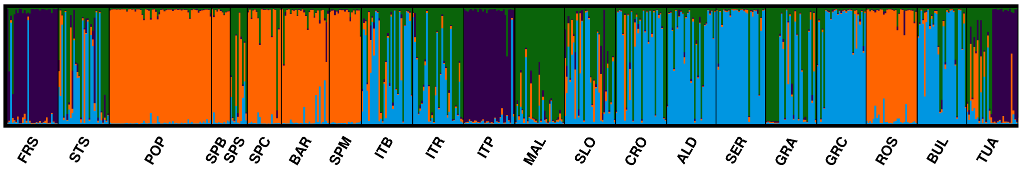

K=4 albo_microsats_overlap_nopops_run_26_f

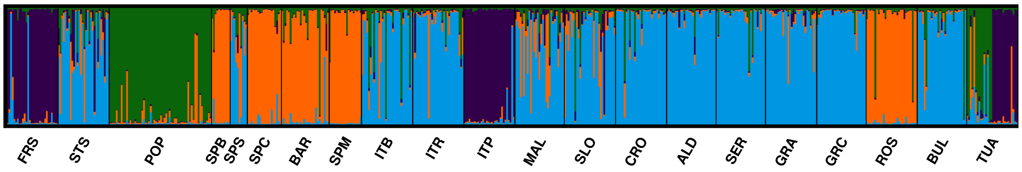

K=4 albo_microsats_overlap_nopops_run_27_f

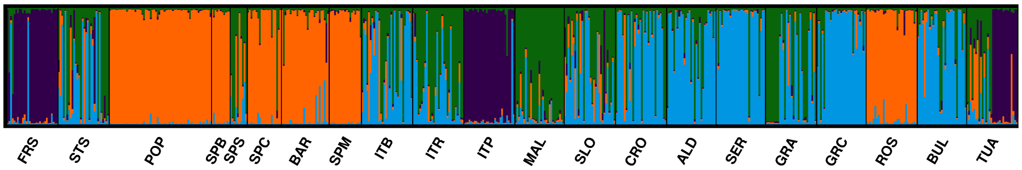

K=4 albo_microsats_overlap_nopops_run_28_f

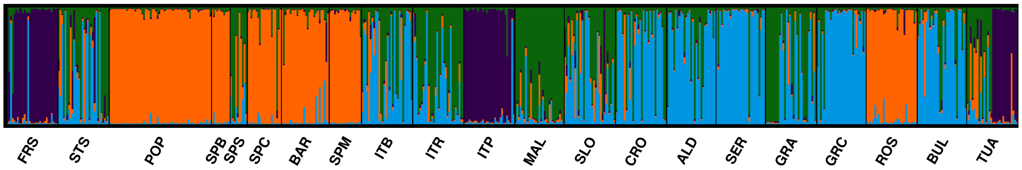

K=4 albo_microsats_overlap_nopops_run_29_f

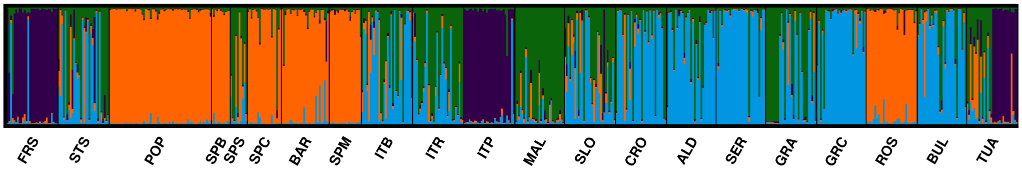

K=4 albo_microsats_overlap_nopops_run_30_f

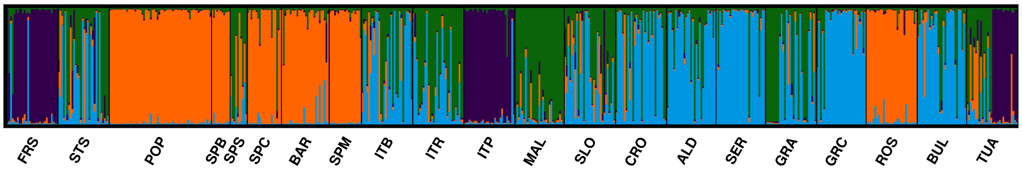

K=5 albo_microsats_overlap_nopops_run_31_f

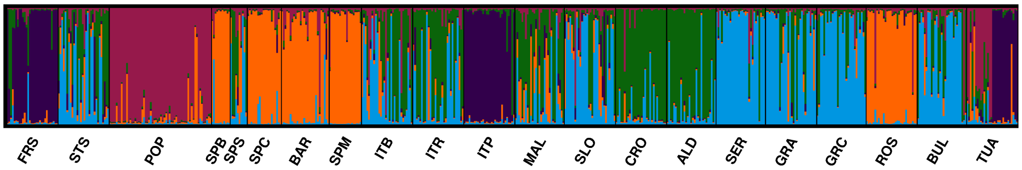

K=5 albo_microsats_overlap_nopops_run_32_f

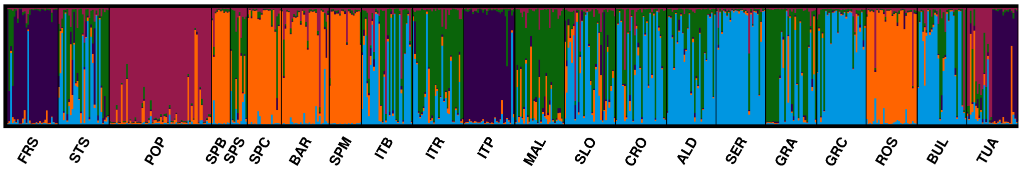

K=5 albo_microsats_overlap_nopops_run_33_f

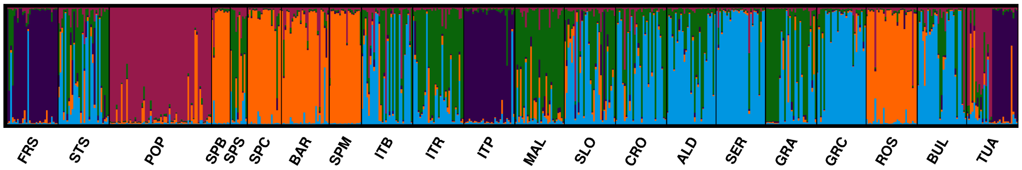

K=5 albo_microsats_overlap_nopops_run_34_f

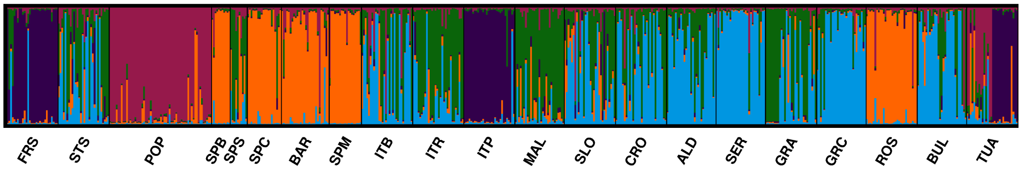

K=5 albo_microsats_overlap_nopops_run_35_f

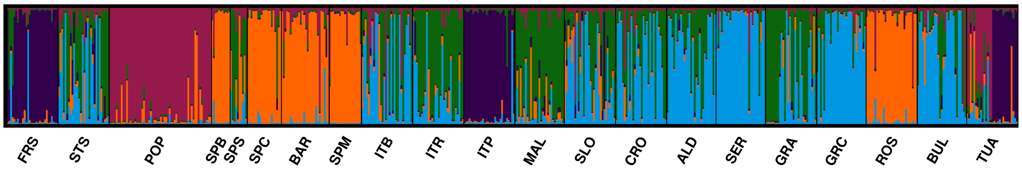

K=5 albo_microsats_overlap_nopops_run_36_f

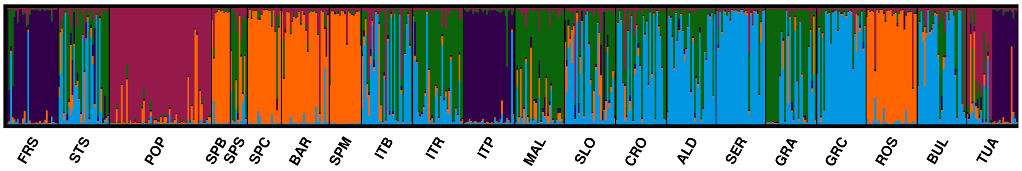

K=5 albo_microsats_overlap_nopops_run_37_f

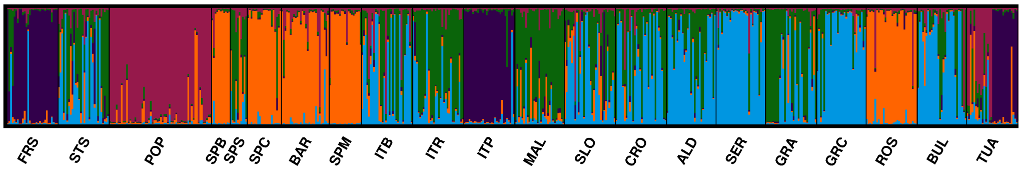

K=5 albo_microsats_overlap_nopops_run_38_f

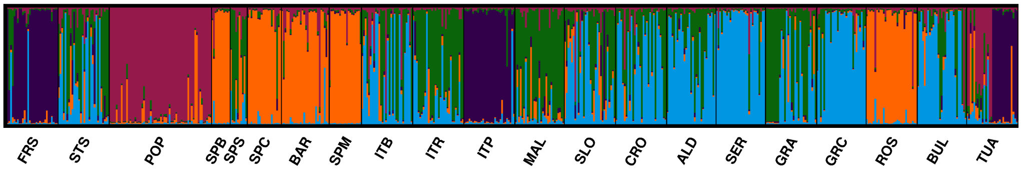

K=5 albo_microsats_overlap_nopops_run_39_f

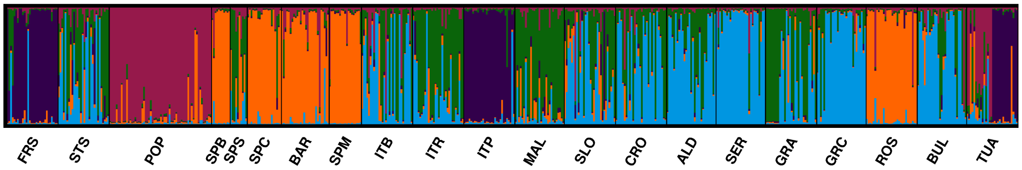

K=5 albo_microsats_overlap_nopops_run_40_f

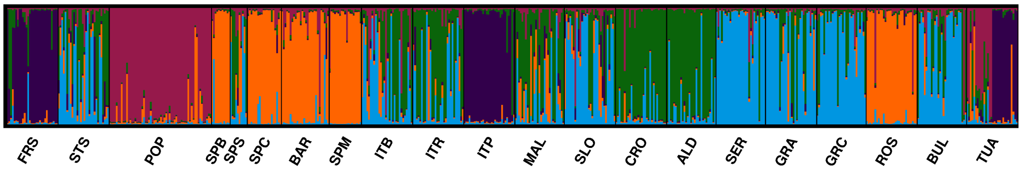

K=6 albo_microsats_overlap_nopops_run_41_f

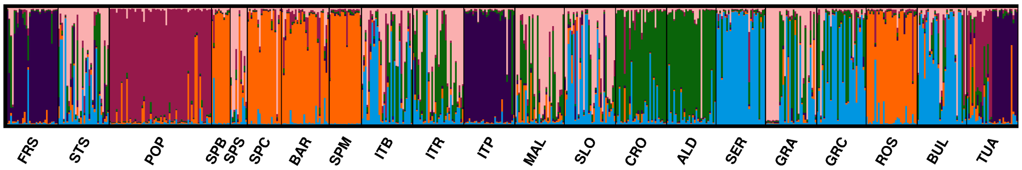

K=6 albo_microsats_overlap_nopops_run_42_f

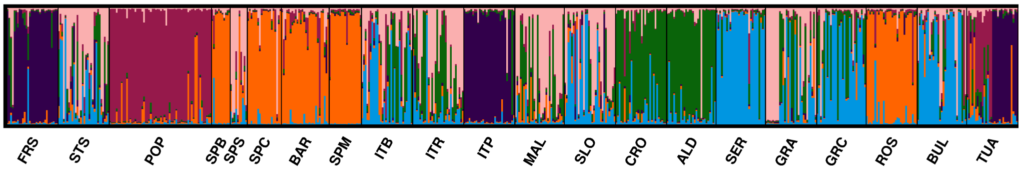

K=6 albo_microsats_overlap_nopops_run_43_f

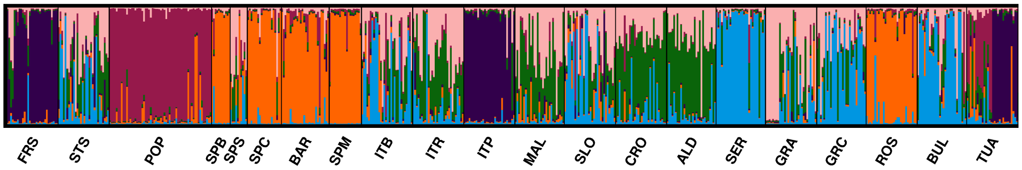

K=6 albo_microsats_overlap_nopops_run_44_f

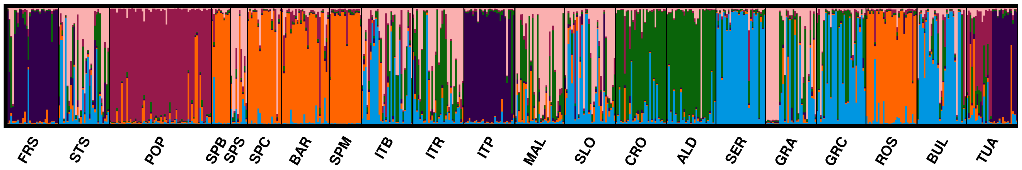

K=6 albo_microsats_overlap_nopops_run_45_f

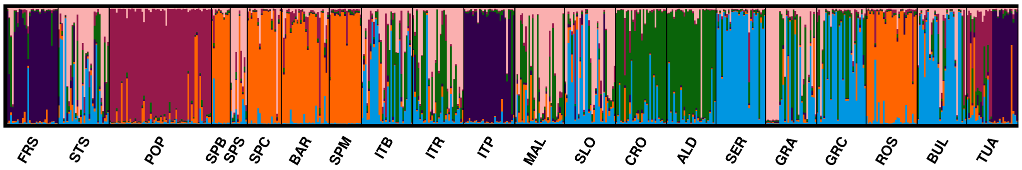

K=6 albo_microsats_overlap_nopops_run_46_f

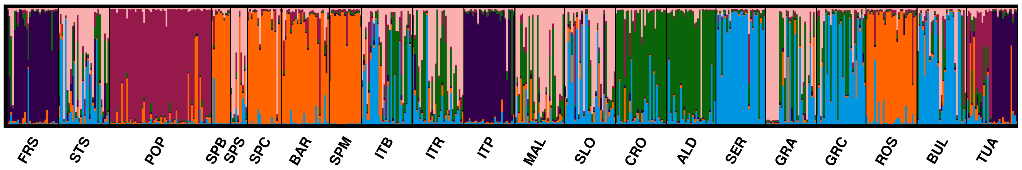

K=6 albo_microsats_overlap_nopops_run_47_f

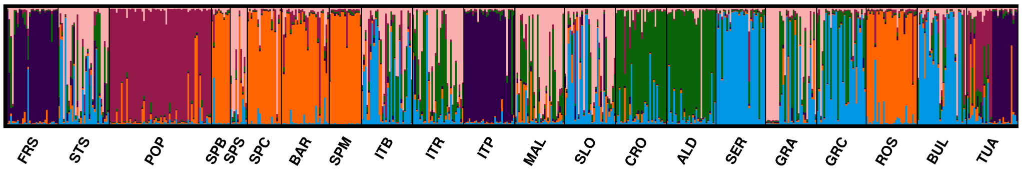

K=6 albo_microsats_overlap_nopops_run_48_f

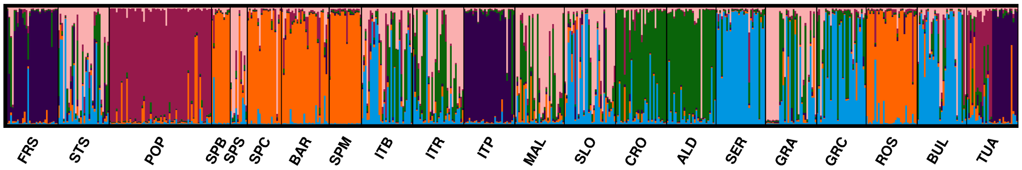

K=6 albo_microsats_overlap_nopops_run_49_f

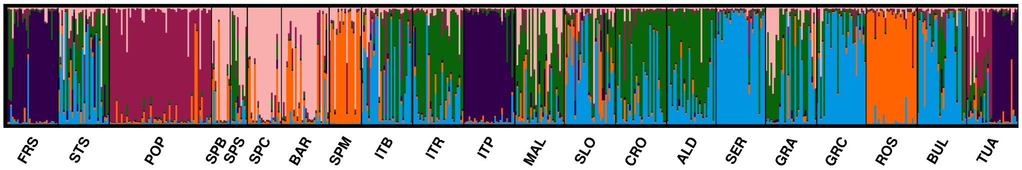

K=6 albo_microsats_overlap_nopops_run_50_f

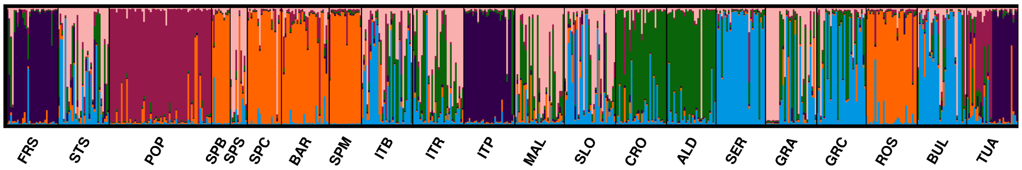

K=7 albo_microsats_overlap_nopops_run_51_f

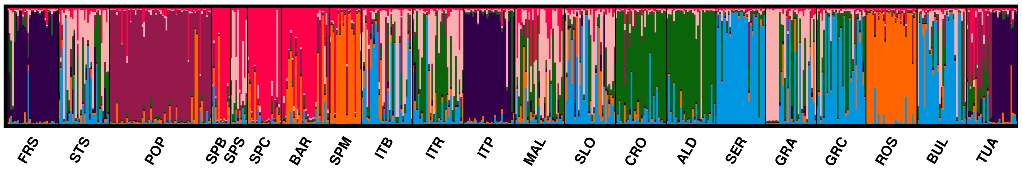

K=7 albo_microsats_overlap_nopops_run_52_f

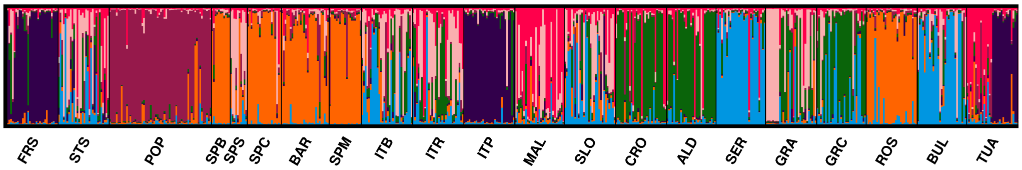

K=7 albo_microsats_overlap_nopops_run_53_f

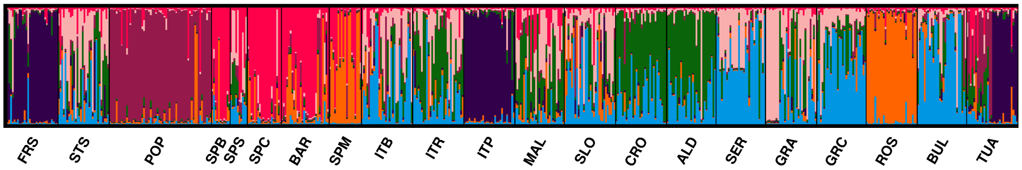

K=7 albo_microsats_overlap_nopops_run_54_f

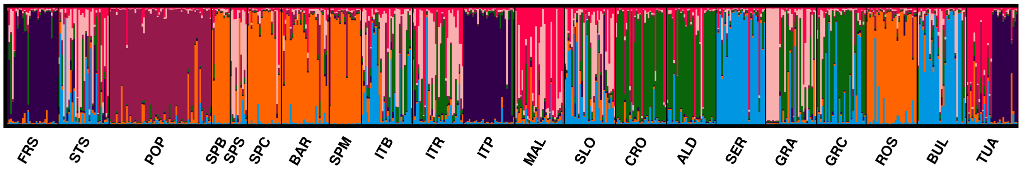

K=7 albo_microsats_overlap_nopops_run_55_f

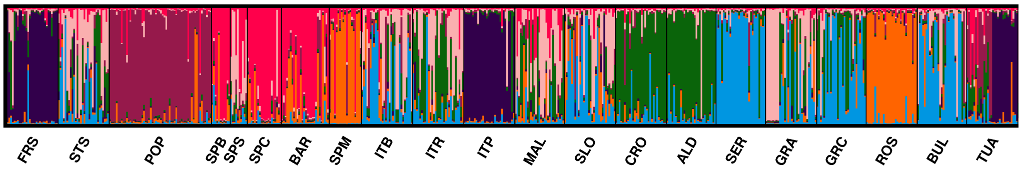

K=7 albo_microsats_overlap_nopops_run_56_f

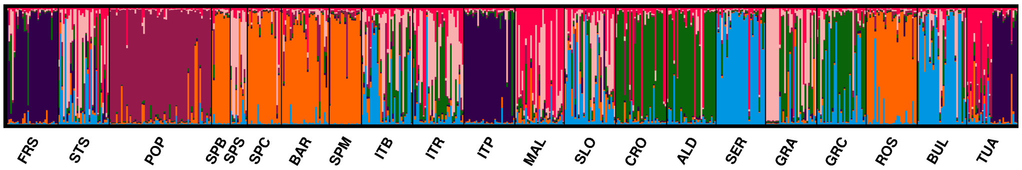

K=7 albo_microsats_overlap_nopops_run_57_f

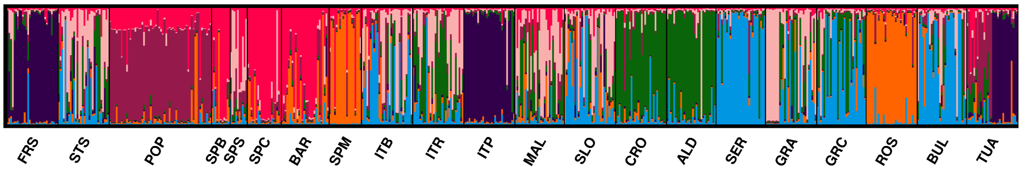

K=7 albo_microsats_overlap_nopops_run_58_f

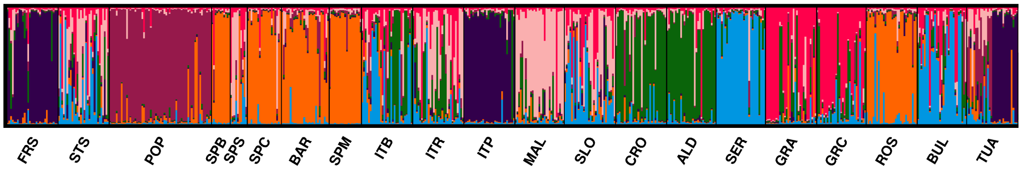

K=7 albo_microsats_overlap_nopops_run_59_f

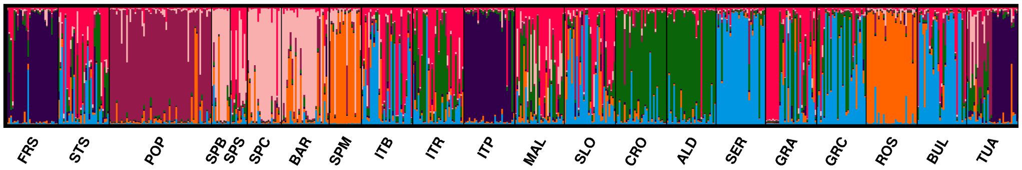

K=7 albo_microsats_overlap_nopops_run_60_f

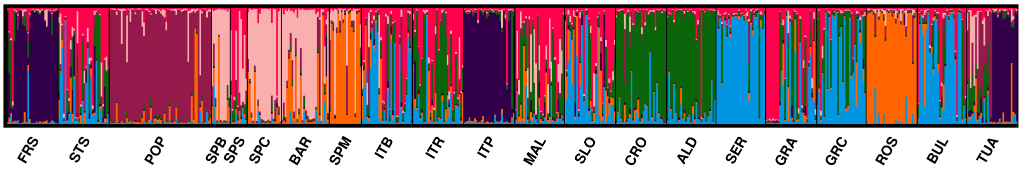

K=8 albo_microsats_overlap_nopops_run_61_f

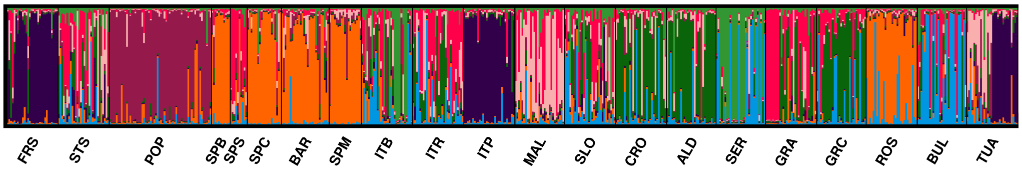

K=8 albo_microsats_overlap_nopops_run_62_f

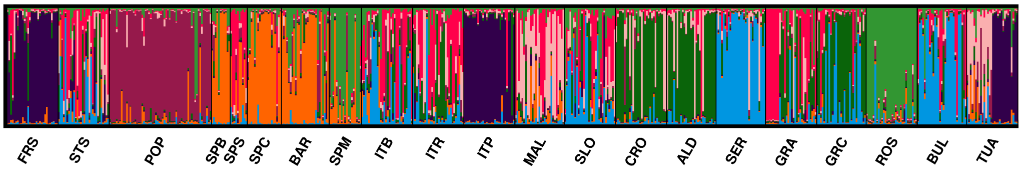

K=8 albo_microsats_overlap_nopops_run_63_f

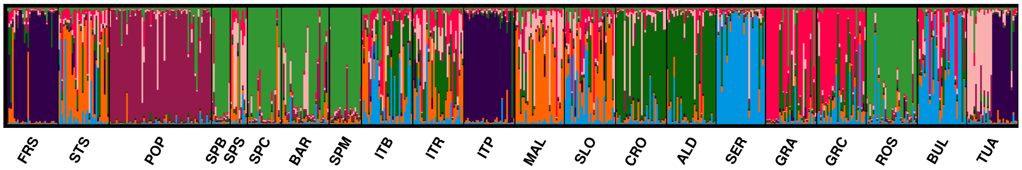

K=8 albo_microsats_overlap_nopops_run_64_f

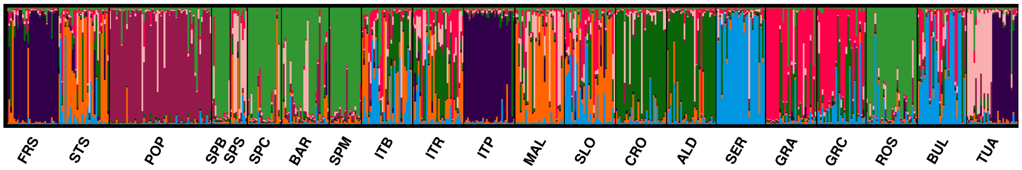

K=8 albo_microsats_overlap_nopops_run_65_f

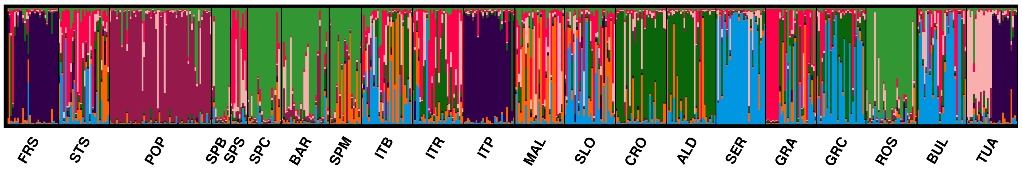

K=8 albo_microsats_overlap_nopops_run_66_f

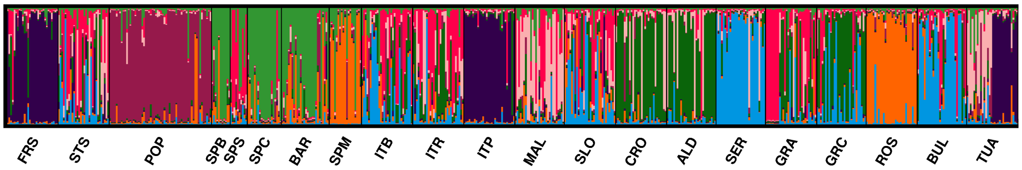

K=8 albo_microsats_overlap_nopops_run_67_f

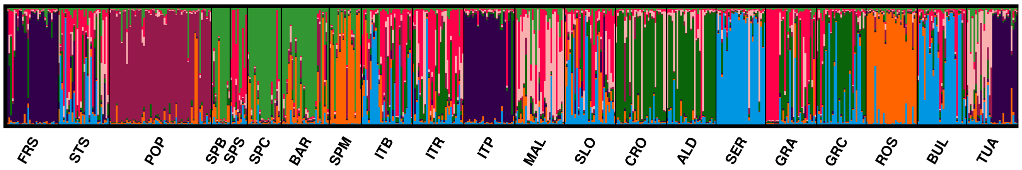

K=8 albo_microsats_overlap_nopops_run_68_f

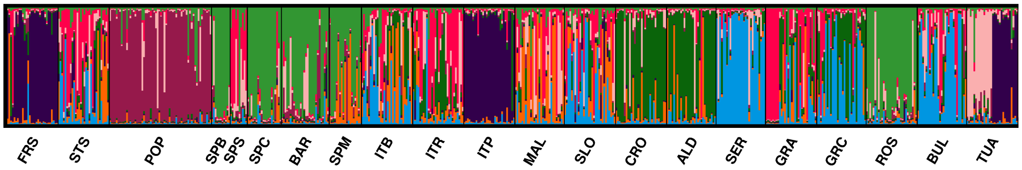

K=8 albo_microsats_overlap_nopops_run_69_f

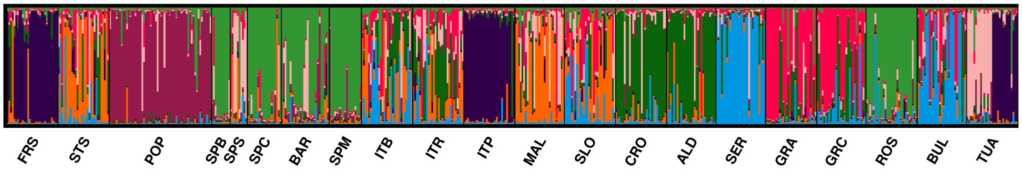

K=8 albo_microsats_overlap_nopops_run_70_f

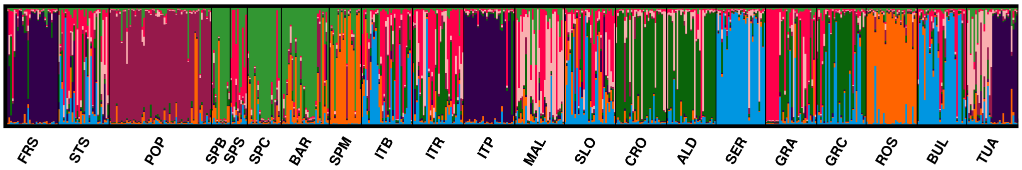

K=9 albo_microsats_overlap_nopops_run_71_f

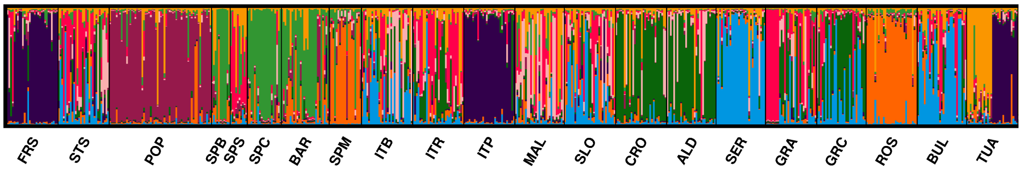

K=9 albo_microsats_overlap_nopops_run_72_f

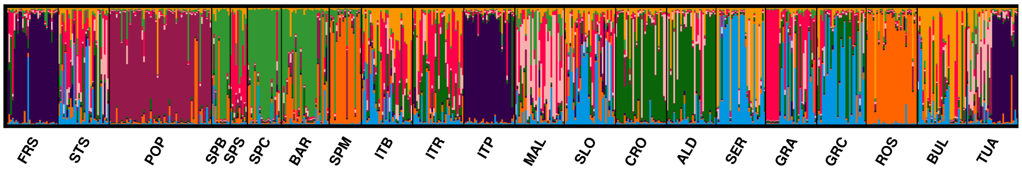

K=9 albo_microsats_overlap_nopops_run_73_f

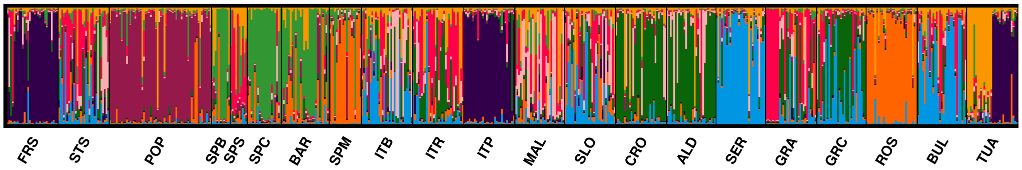

K=9 albo_microsats_overlap_nopops_run_74_f

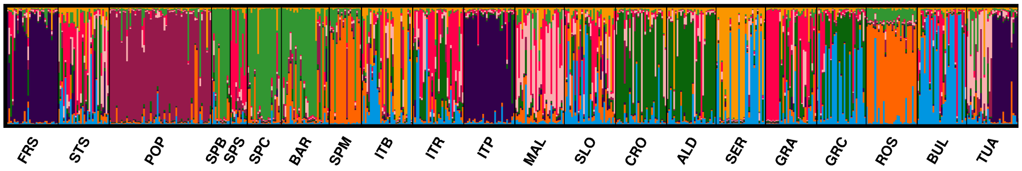

K=9 albo_microsats_overlap_nopops_run_75_f

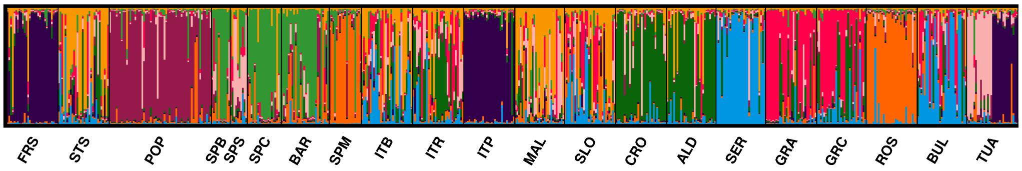

K=9 albo_microsats_overlap_nopops_run_76_f

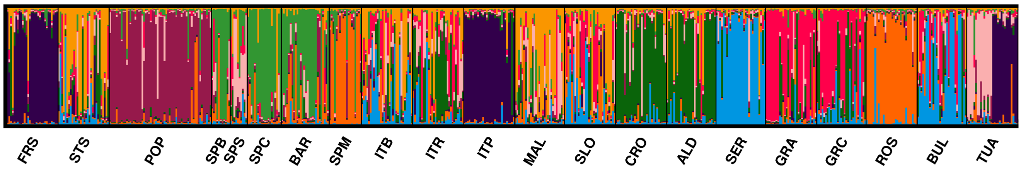

K=9 albo_microsats_overlap_nopops_run_77_f

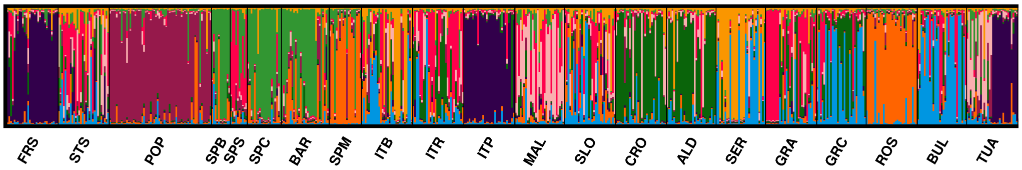

K=9 albo_microsats_overlap_nopops_run_78_f

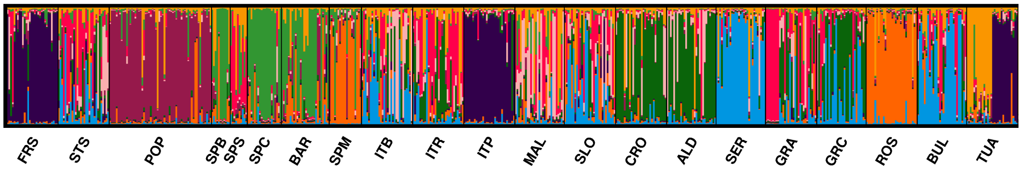

K=9 albo_microsats_overlap_nopops_run_79_f

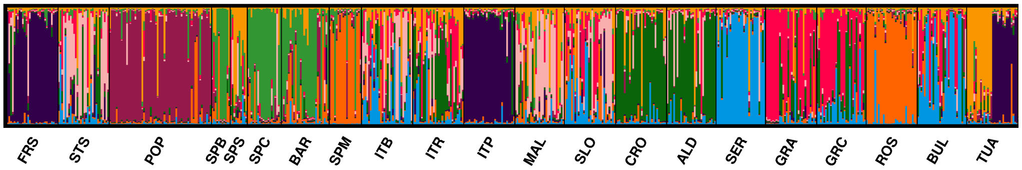

K=9 albo_microsats_overlap_nopops_run_80_f

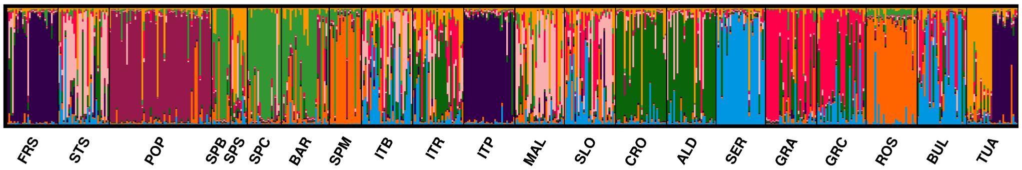

K=10 albo_microsats_overlap_nopops_run_81_f

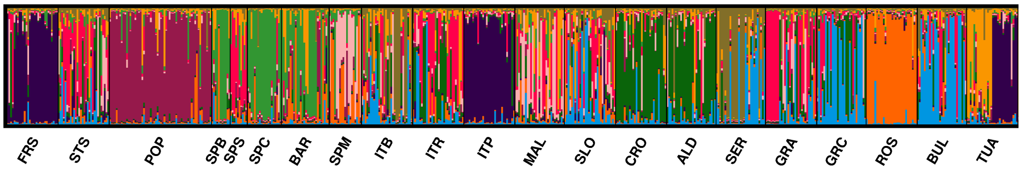

K=10 albo_microsats_overlap_nopops_run_82_f

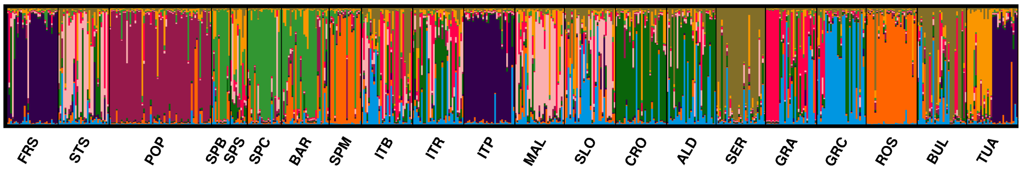

K=10 albo_microsats_overlap_nopops_run_83_f

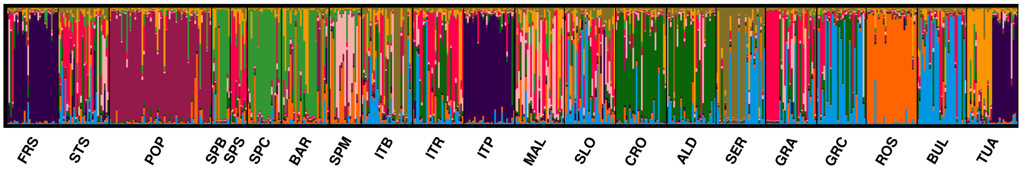

K=10 albo_microsats_overlap_nopops_run_84_f

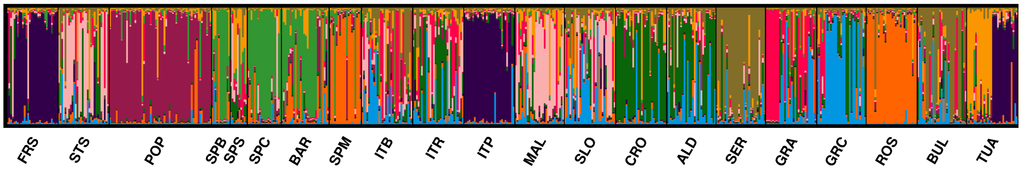

K=10 albo_microsats_overlap_nopops_run_85_f

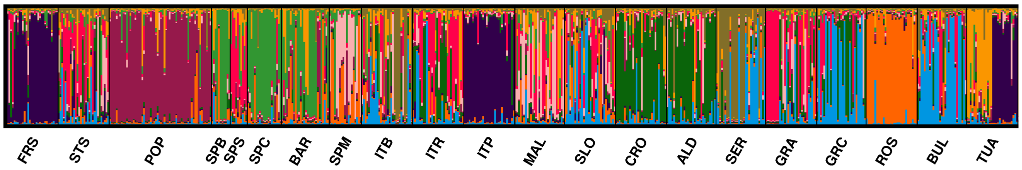

K=10 albo_microsats_overlap_nopops_run_86_f

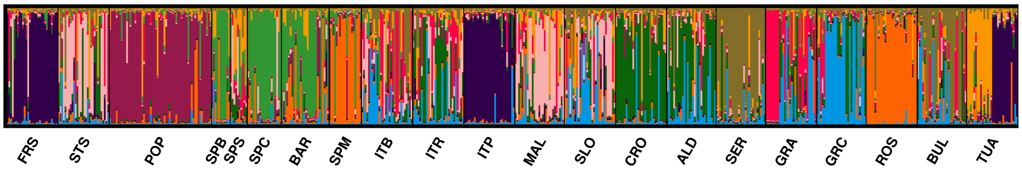

K=10 albo_microsats_overlap_nopops_run_87_f

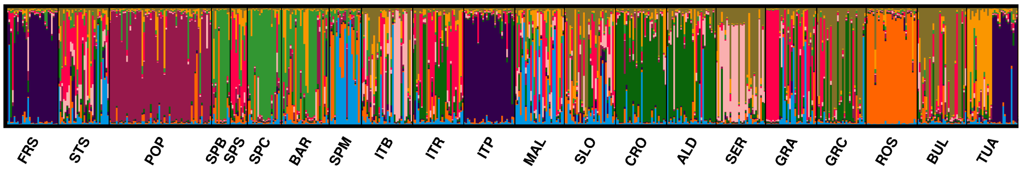

K=10 albo_microsats_overlap_nopops_run_88_f

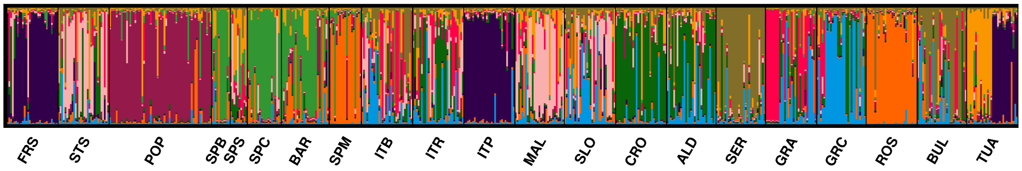

K=10 albo_microsats_overlap_nopops_run_89_f

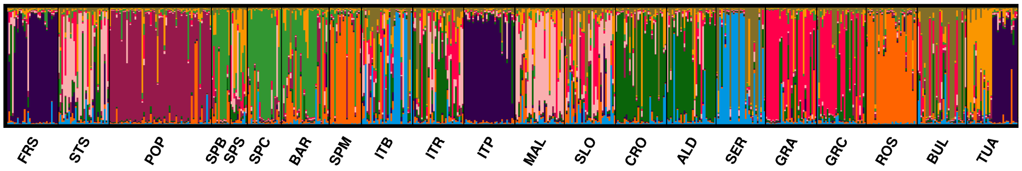

K=10 albo_microsats_overlap_nopops_run_90_f

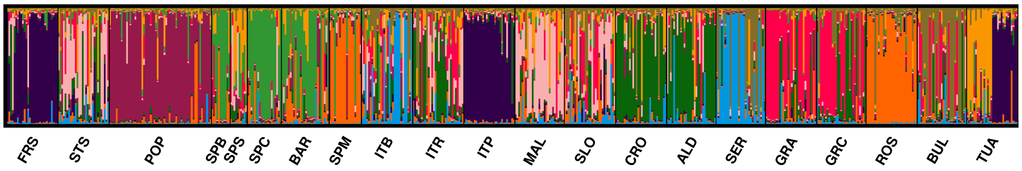

K=11 albo_microsats_overlap_nopops_run_100_f

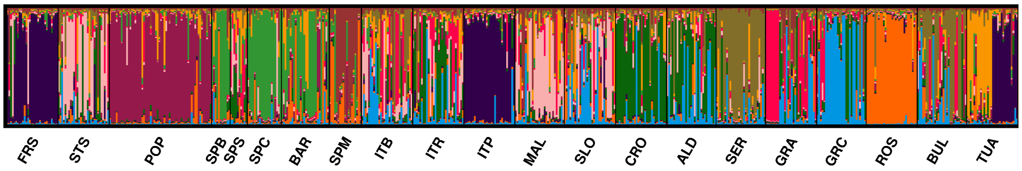

K=11 albo_microsats_overlap_nopops_run_91_f

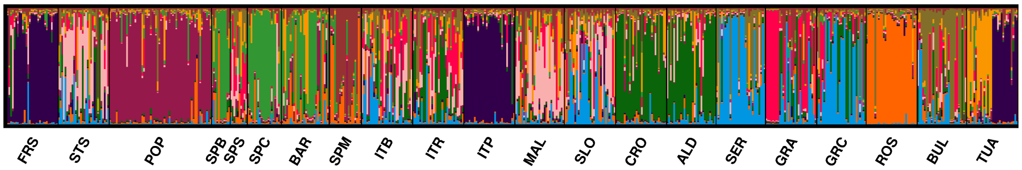

K=11 albo_microsats_overlap_nopops_run_92_f

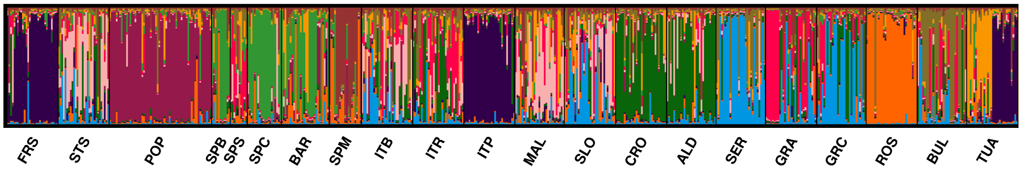

K=11 albo_microsats_overlap_nopops_run_93_f

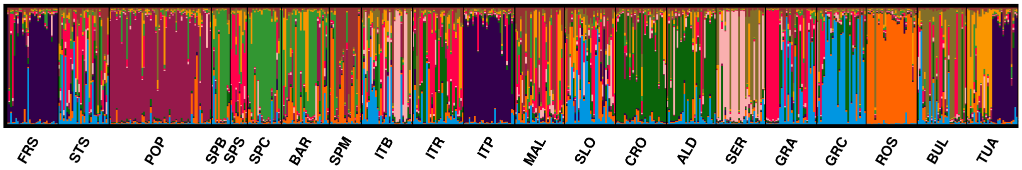

K=11 albo_microsats_overlap_nopops_run_94_f

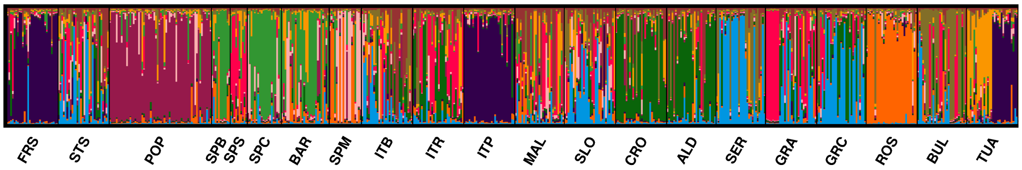

K=11 albo_microsats_overlap_nopops_run_95_f

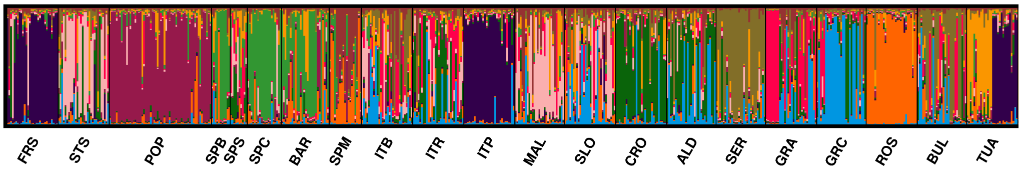

K=11 albo_microsats_overlap_nopops_run_96_f

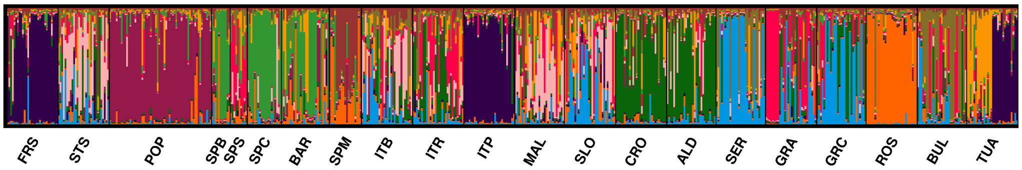

K=11 albo_microsats_overlap_nopops_run_97_f

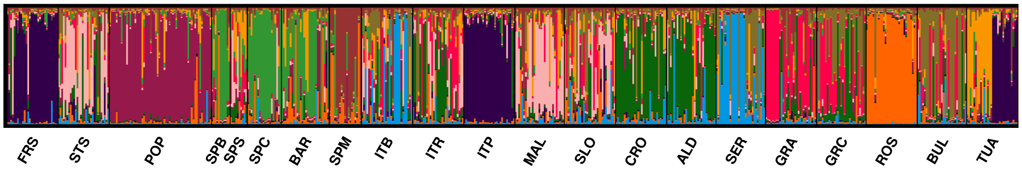

K=11 albo_microsats_overlap_nopops_run_98_f

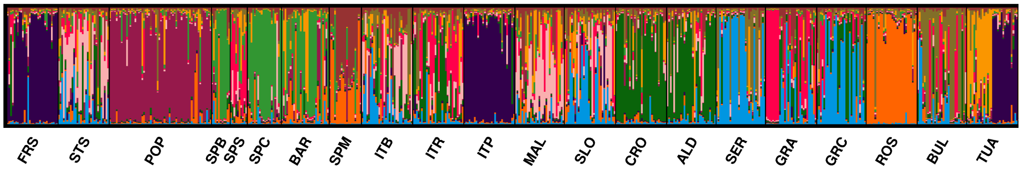

K=11 albo_microsats_overlap_nopops_run_99_f

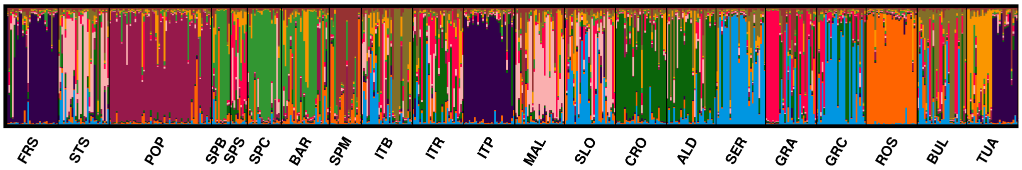

K=12 albo_microsats_overlap_nopops_run_101_f

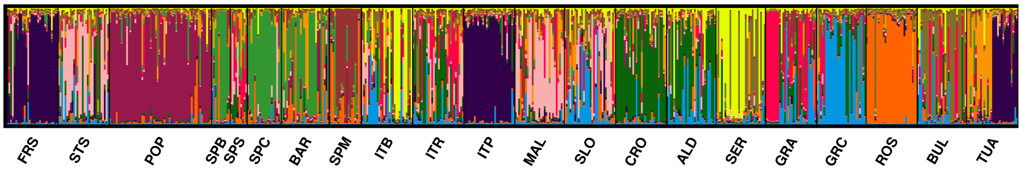

K=12 albo_microsats_overlap_nopops_run_102_f

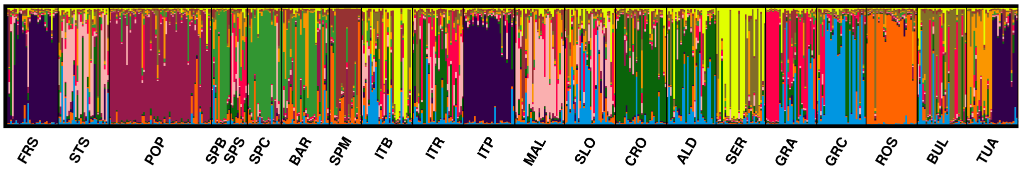

K=12 albo_microsats_overlap_nopops_run_103_f

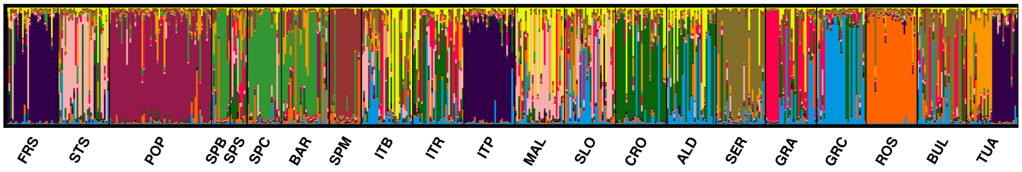

K=12 albo_microsats_overlap_nopops_run_104_f

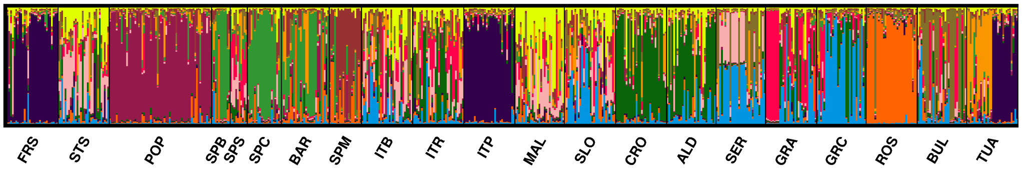

K=12 albo_microsats_overlap_nopops_run_105_f

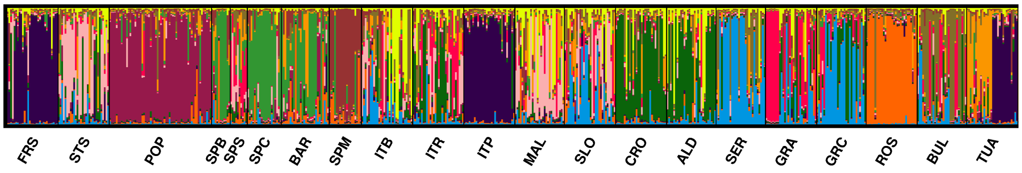

K=12 albo_microsats_overlap_nopops_run_106_f

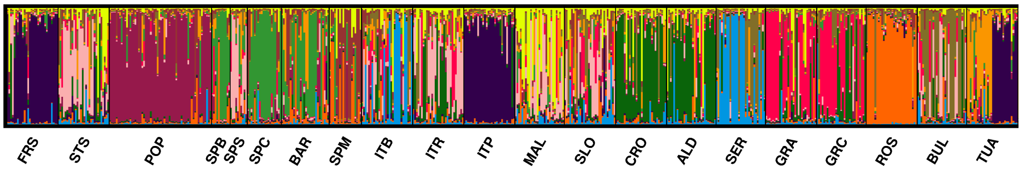

K=12 albo_microsats_overlap_nopops_run_107_f

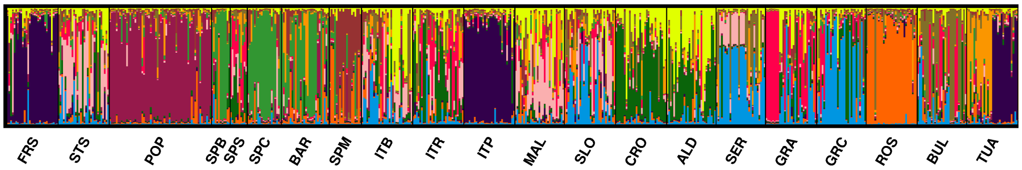

K=12 albo_microsats_overlap_nopops_run_108_f

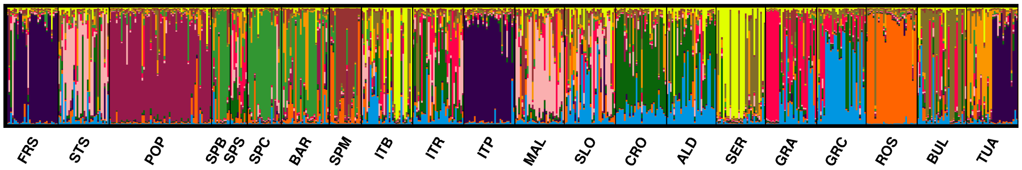

K=12 albo_microsats_overlap_nopops_run_109_f

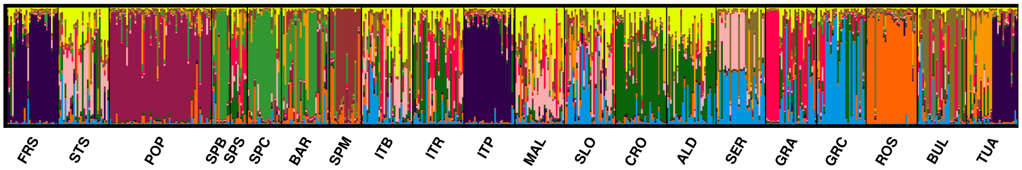

K=12 albo_microsats_overlap_nopops_run_110_f

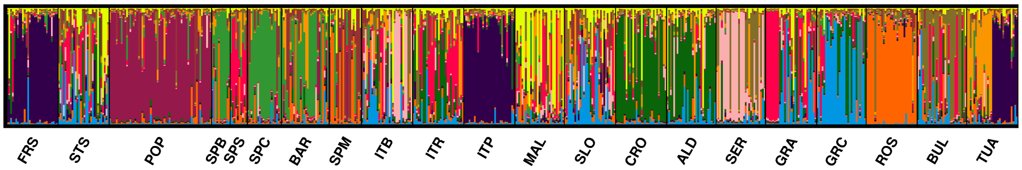

K=13 albo_microsats_overlap_nopops_run_111_f

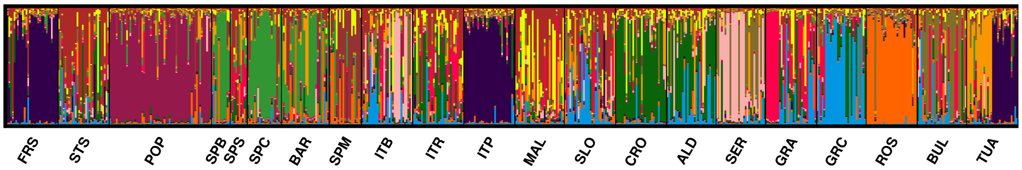

K=13 albo_microsats_overlap_nopops_run_112_f

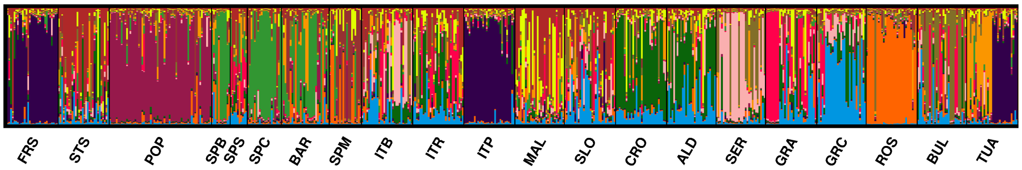

K=13 albo_microsats_overlap_nopops_run_113_f

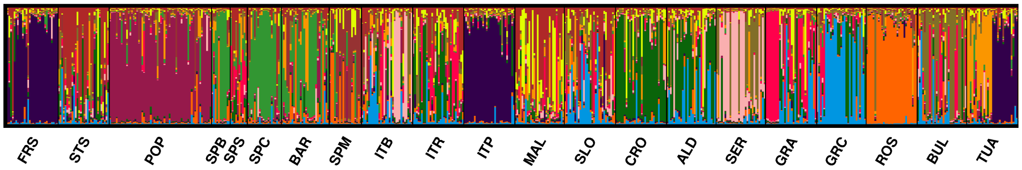

K=13 albo_microsats_overlap_nopops_run_114_f

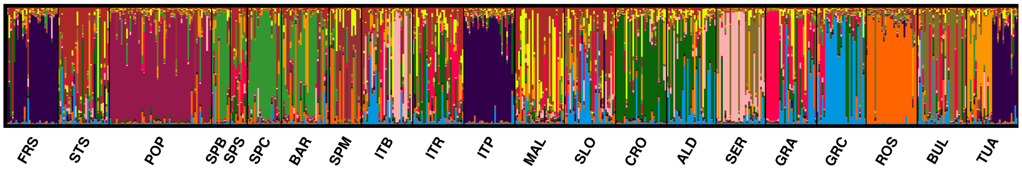

K=13 albo_microsats_overlap_nopops_run_115_f

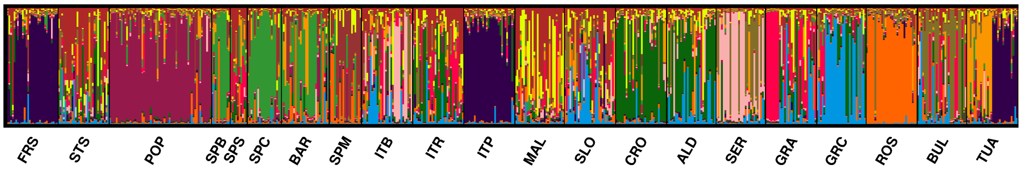

K=13 albo_microsats_overlap_nopops_run_116_f

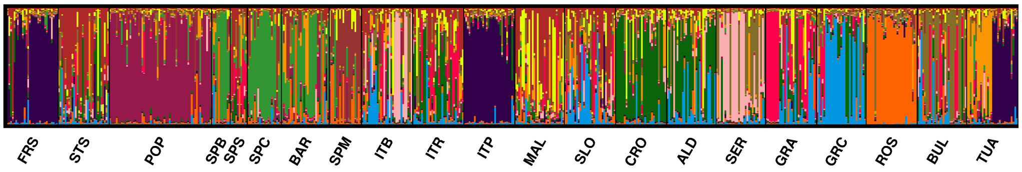

K=13 albo_microsats_overlap_nopops_run_117_f

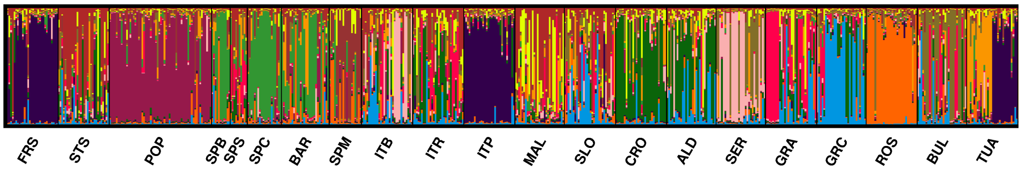

K=13 albo_microsats_overlap_nopops_run_118_f

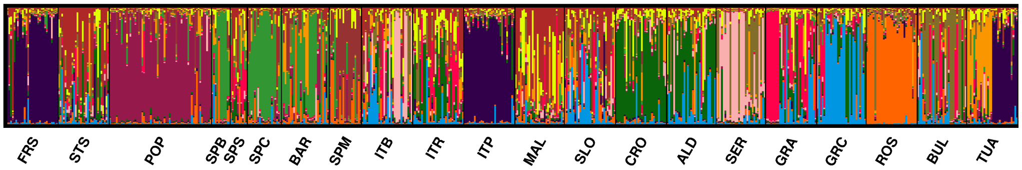

K=13 albo_microsats_overlap_nopops_run_119_f

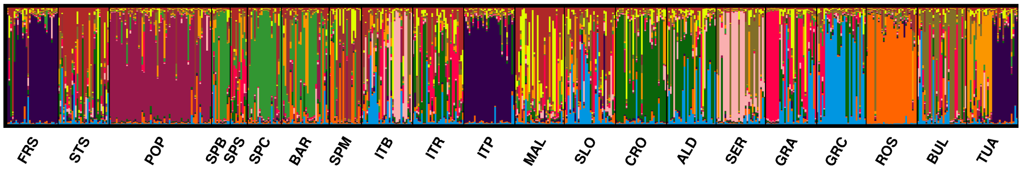

K=13 albo_microsats_overlap_nopops_run_120_f

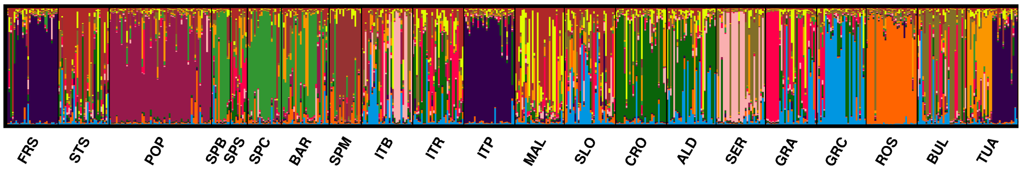

K=14 albo_microsats_overlap_nopops_run_121_f

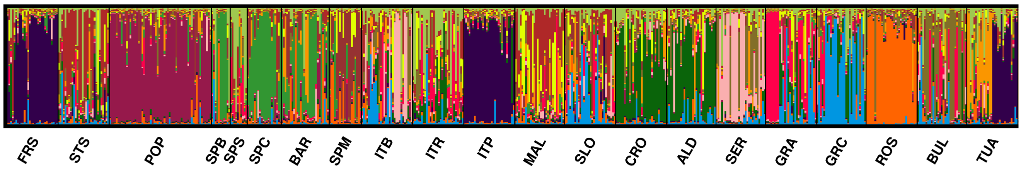

K=14 albo_microsats_overlap_nopops_run_122_f

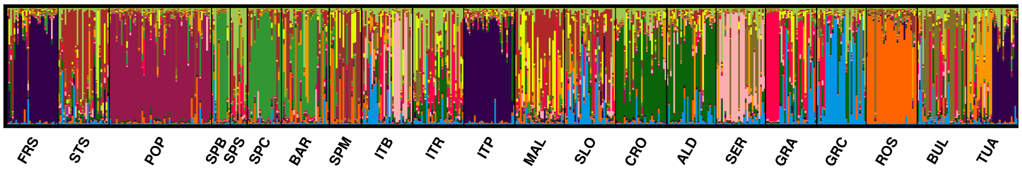

K=14 albo_microsats_overlap_nopops_run_123_f

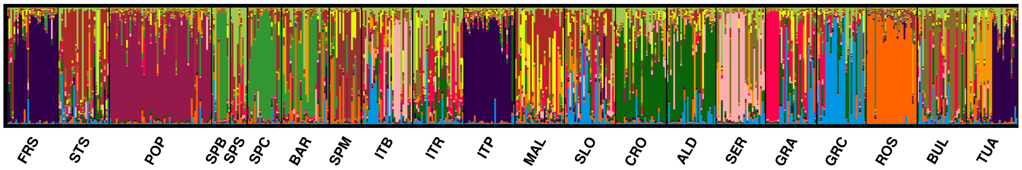

K=14 albo_microsats_overlap_nopops_run_124_f

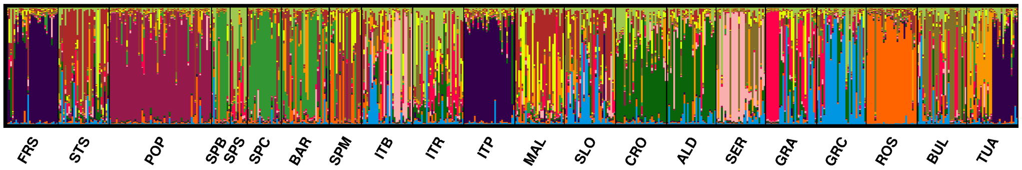

K=14 albo_microsats_overlap_nopops_run_125_f

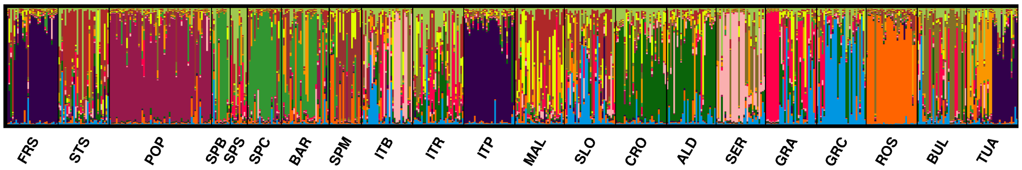

K=14 albo_microsats_overlap_nopops_run_126_f

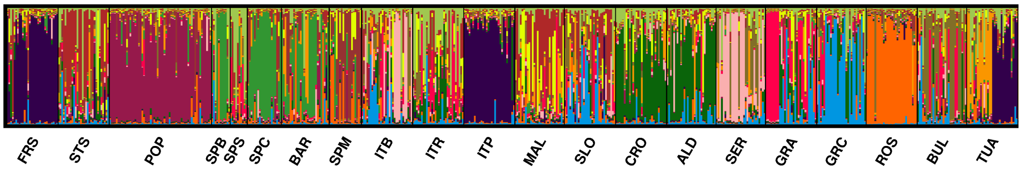

K=14 albo_microsats_overlap_nopops_run_127_f

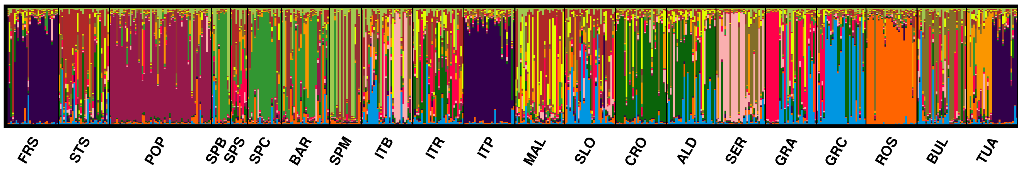

K=14 albo_microsats_overlap_nopops_run_128_f

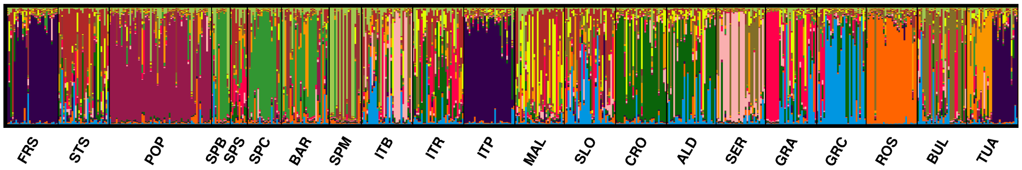

K=14 albo_microsats_overlap_nopops_run_129_f

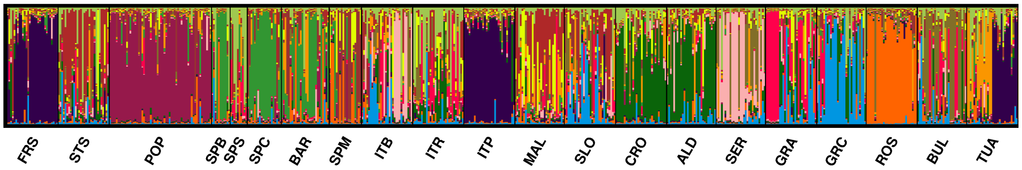

K=14 albo_microsats_overlap_nopops_run_130_f

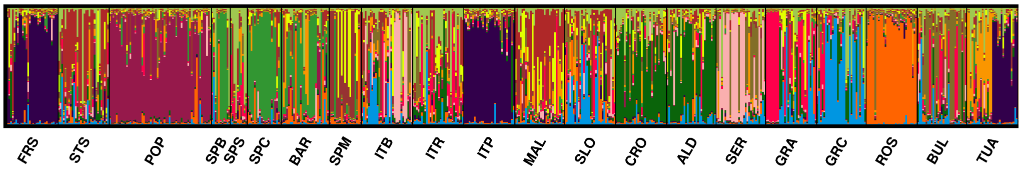

K=15 albo_microsats_overlap_nopops_run_131_f

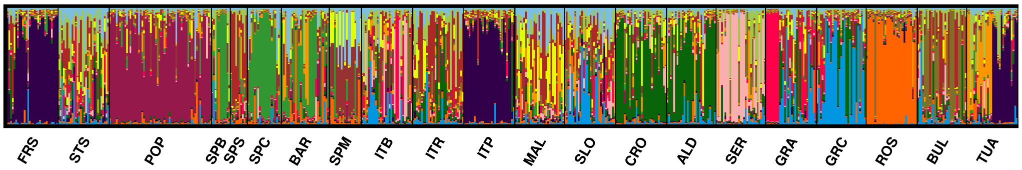

K=15 albo_microsats_overlap_nopops_run_132_f

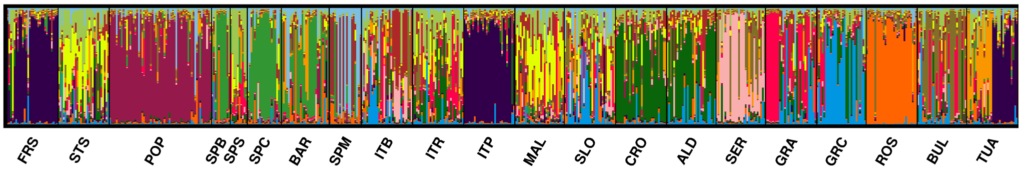

K=15 albo_microsats_overlap_nopops_run_133_f

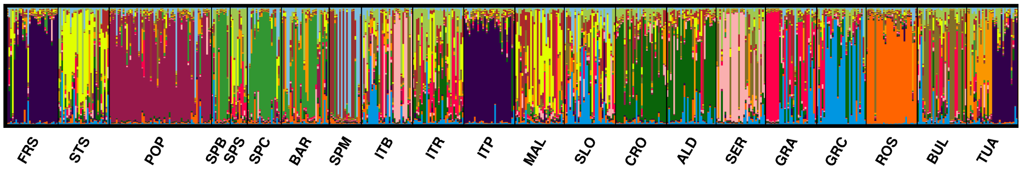

K=15 albo_microsats_overlap_nopops_run_134_f

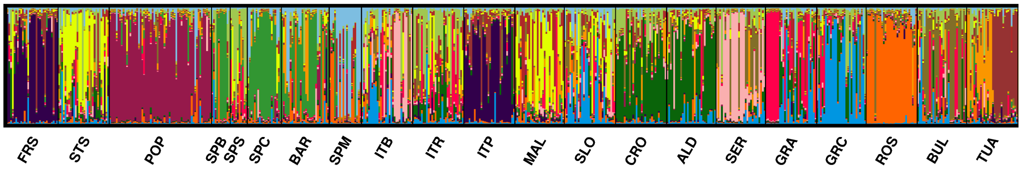

K=15 albo_microsats_overlap_nopops_run_135_f

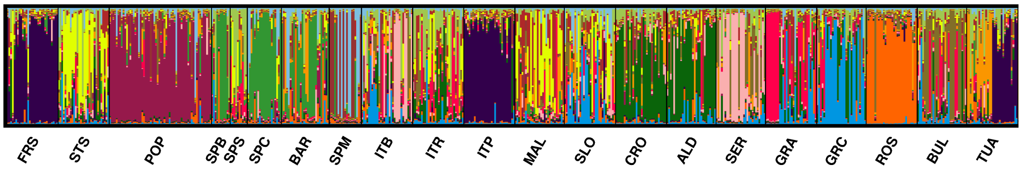

K=15 albo_microsats_overlap_nopops_run_136_f

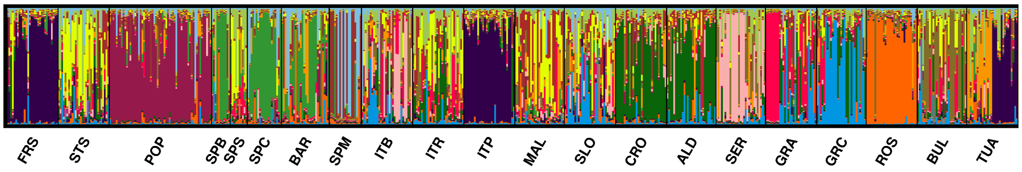

K=15 albo_microsats_overlap_nopops_run_137_f

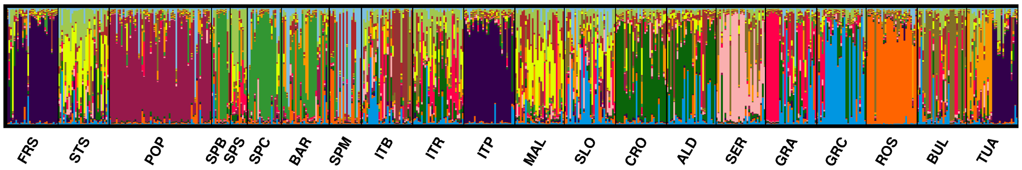

K=15 albo_microsats_overlap_nopops_run_138_f

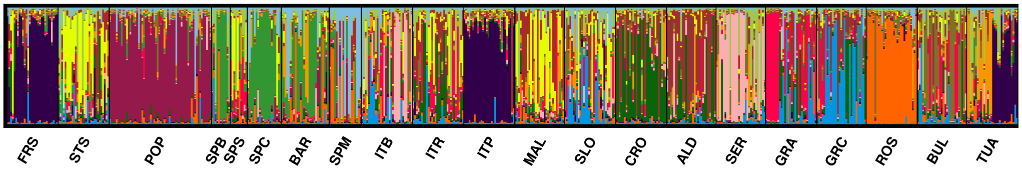

K=15 albo_microsats_overlap_nopops_run_139_f

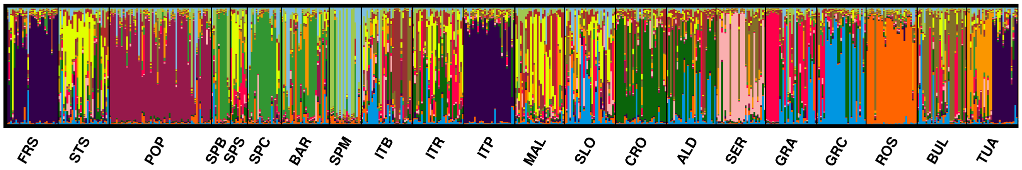

K=15 albo_microsats_overlap_nopops_run_140_f

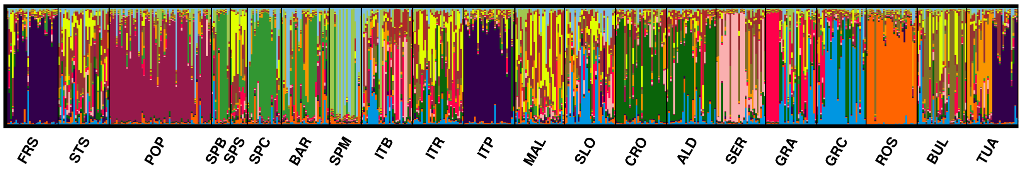

K=16 albo_microsats_overlap_nopops_run_141_f

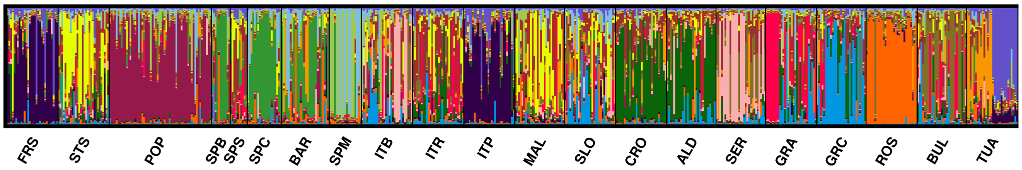

K=16 albo_microsats_overlap_nopops_run_142_f

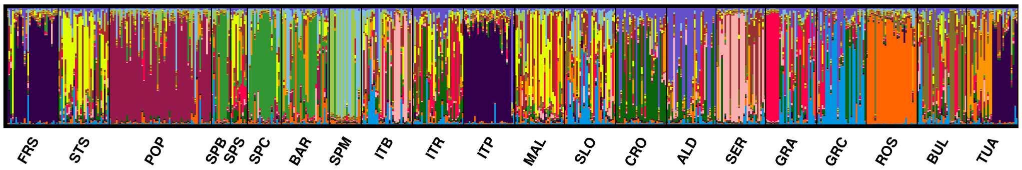

K=16 albo_microsats_overlap_nopops_run_143_f

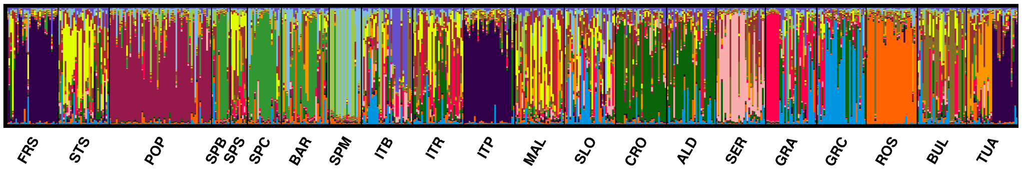

K=16 albo_microsats_overlap_nopops_run_144_f

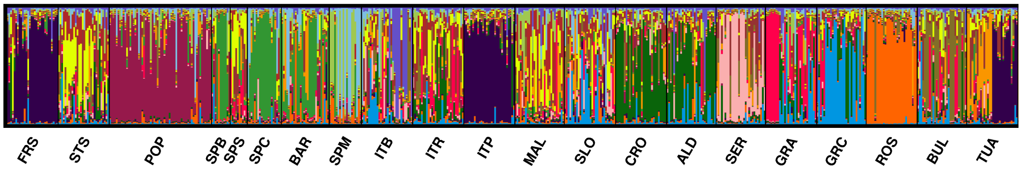

K=16 albo_microsats_overlap_nopops_run_145_f

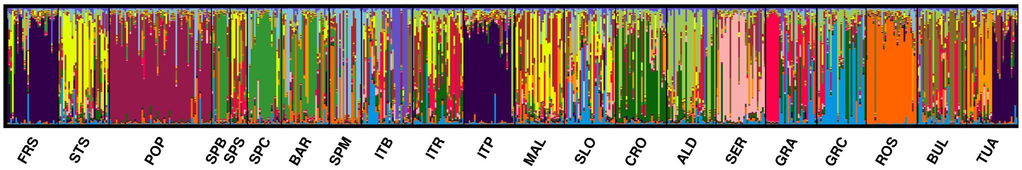

K=16 albo_microsats_overlap_nopops_run_146_f

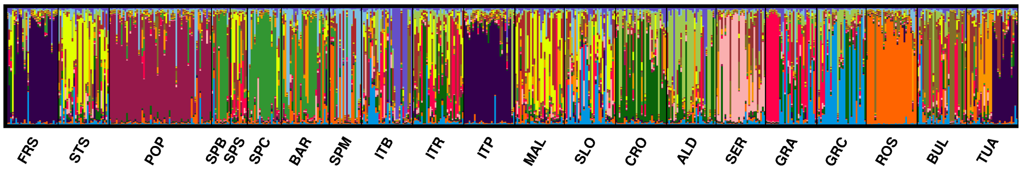

K=16 albo_microsats_overlap_nopops_run_147_f

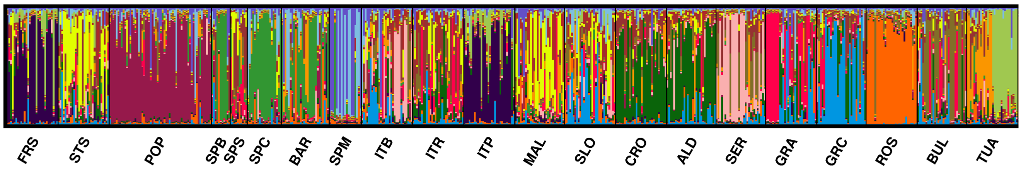

K=16 albo_microsats_overlap_nopops_run_148_f

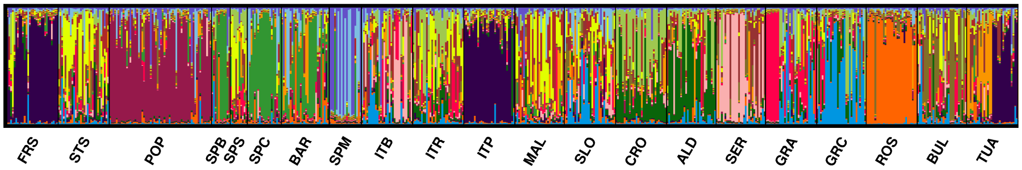

K=16 albo_microsats_overlap_nopops_run_149_f

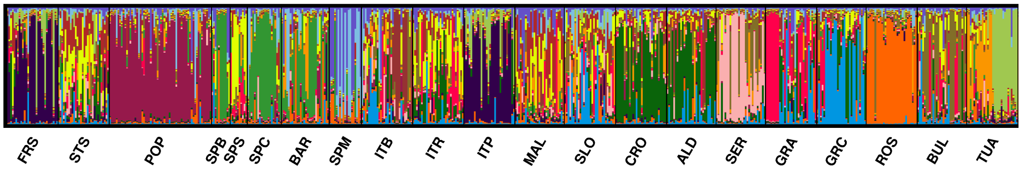

K=16 albo_microsats_overlap_nopops_run_150_f

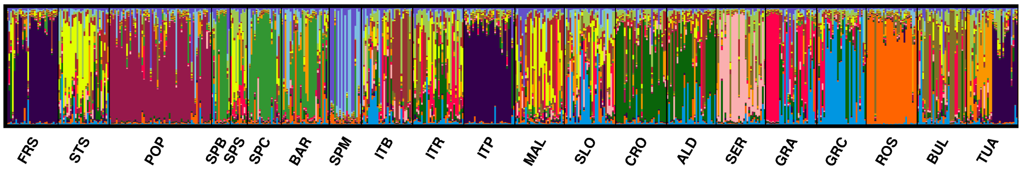

K=17 albo_microsats_overlap_nopops_run_151_f

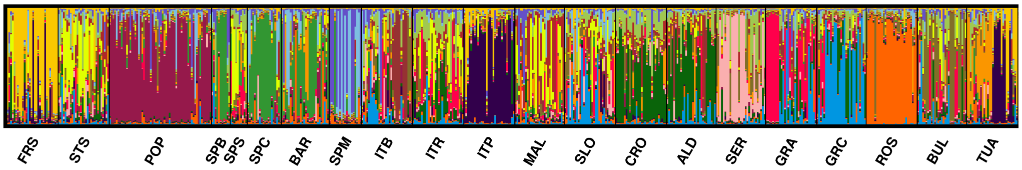

K=17 albo_microsats_overlap_nopops_run_152_f

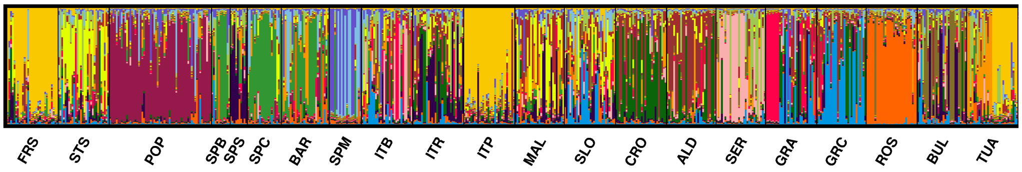

K=17 albo_microsats_overlap_nopops_run_153_f

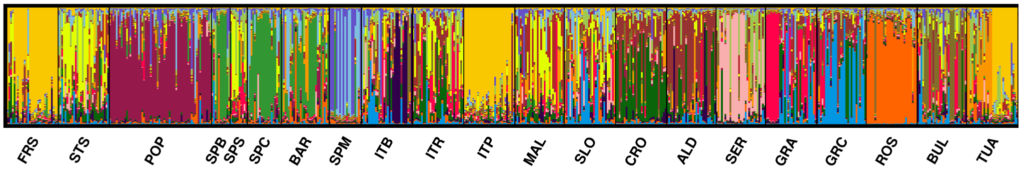

K=17 albo_microsats_overlap_nopops_run_154_f

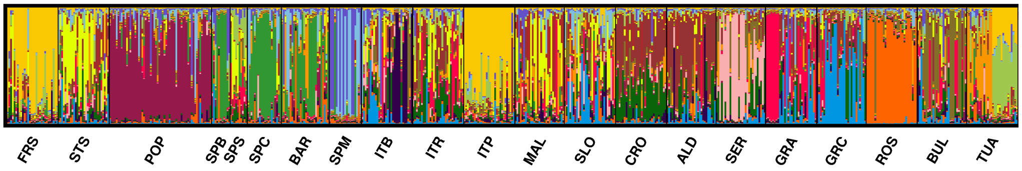

K=17 albo_microsats_overlap_nopops_run_155_f

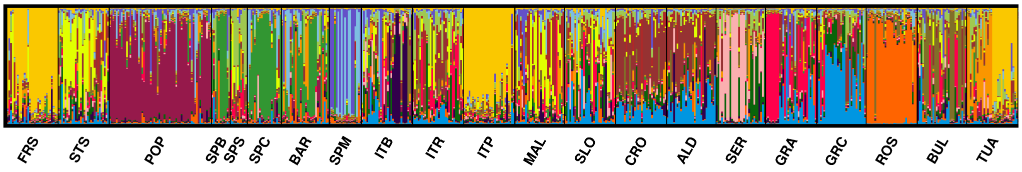

K=17 albo_microsats_overlap_nopops_run_156_f

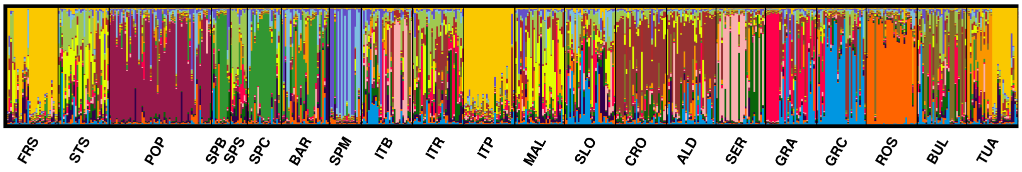

K=17 albo_microsats_overlap_nopops_run_157_f

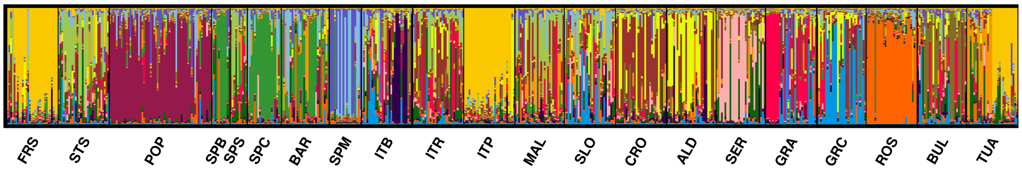

K=17 albo_microsats_overlap_nopops_run_158_f

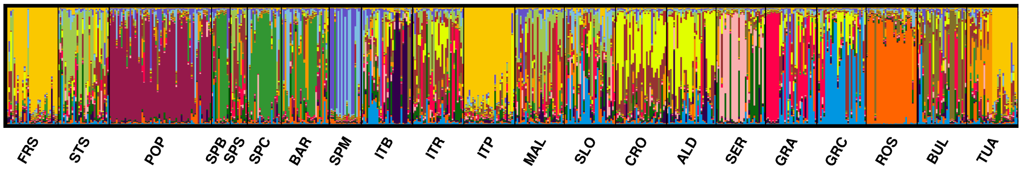

K=17 albo_microsats_overlap_nopops_run_159_f

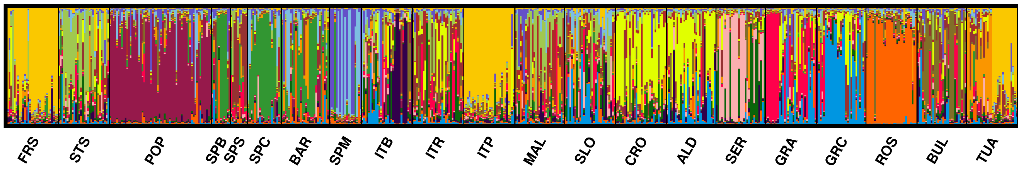

K=17 albo_microsats_overlap_nopops_run_160_f

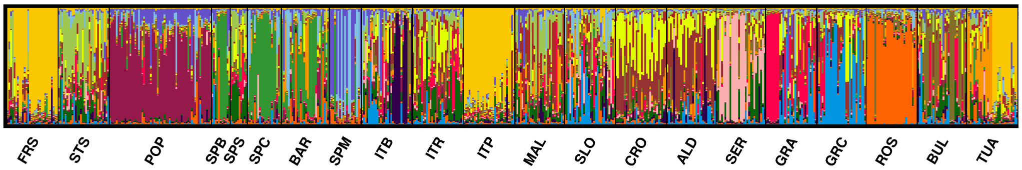

K=18 albo_microsats_overlap_nopops_run_161_f

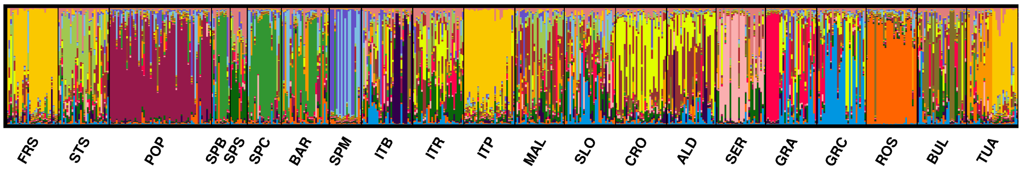

K=18 albo_microsats_overlap_nopops_run_162_f

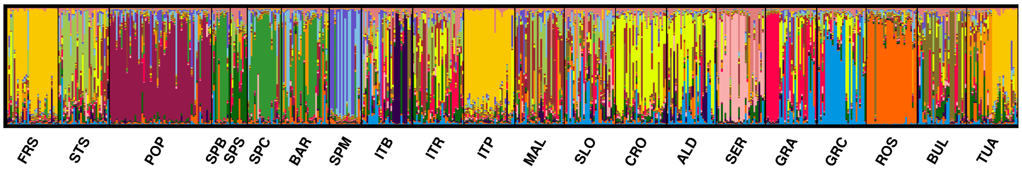

K=18 albo_microsats_overlap_nopops_run_163_f

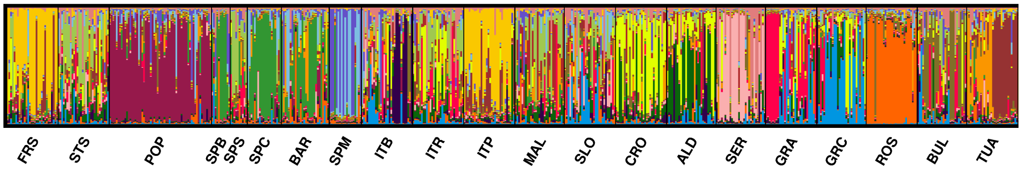

K=18 albo_microsats_overlap_nopops_run_164_f

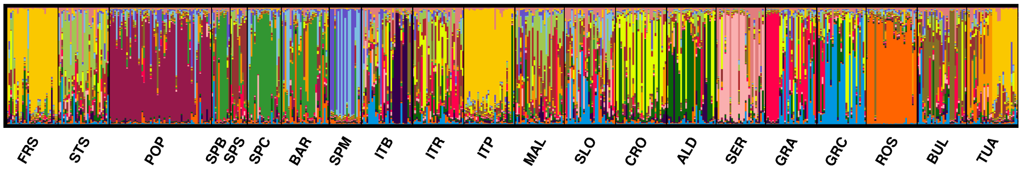

K=18 albo_microsats_overlap_nopops_run_165_f

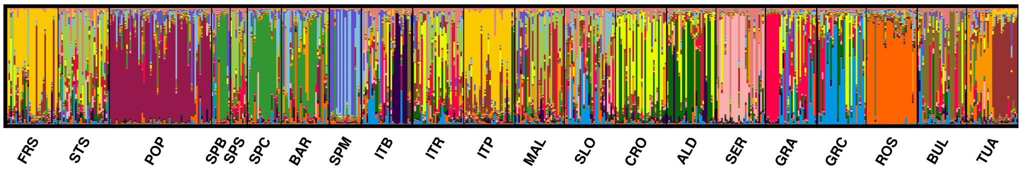

K=18 albo_microsats_overlap_nopops_run_166_f

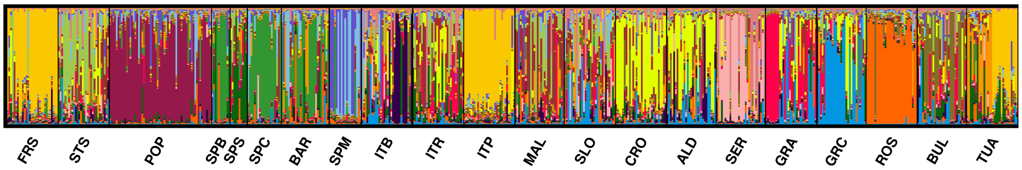

K=18 albo_microsats_overlap_nopops_run_167_f

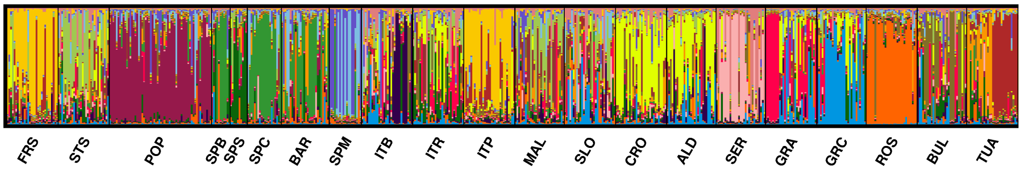

K=18 albo_microsats_overlap_nopops_run_168_f

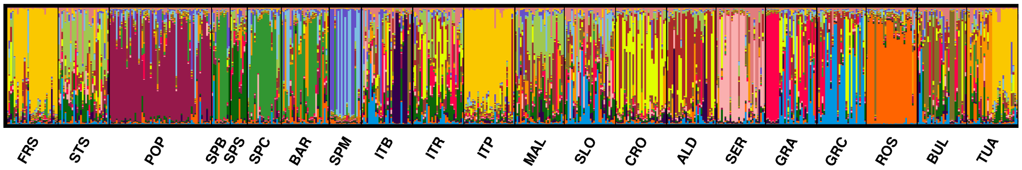

K=18 albo_microsats_overlap_nopops_run_169_f

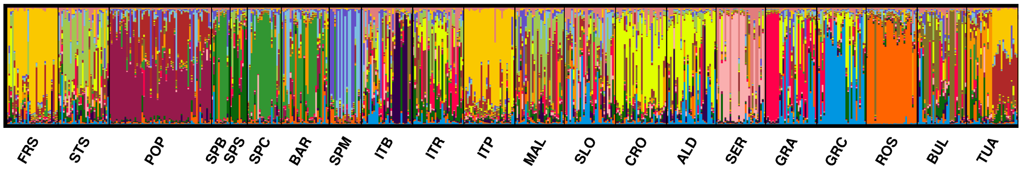

K=18 albo_microsats_overlap_nopops_run_170_f

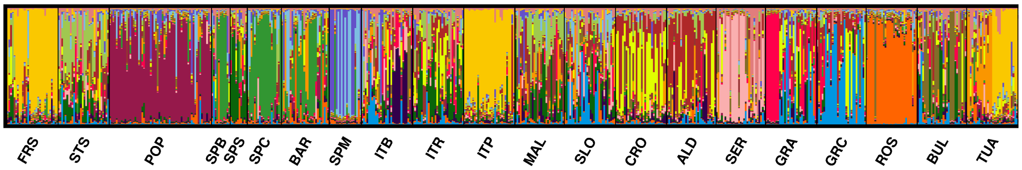

K=19 albo_microsats_overlap_nopops_run_171_f

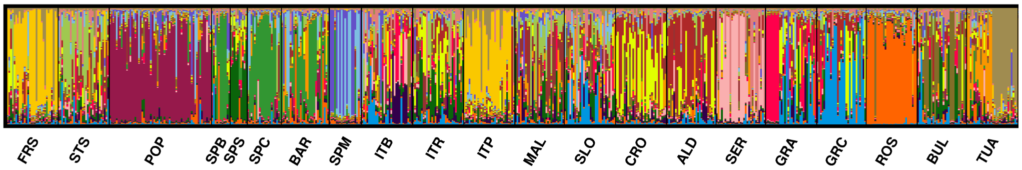

K=19 albo_microsats_overlap_nopops_run_172_f

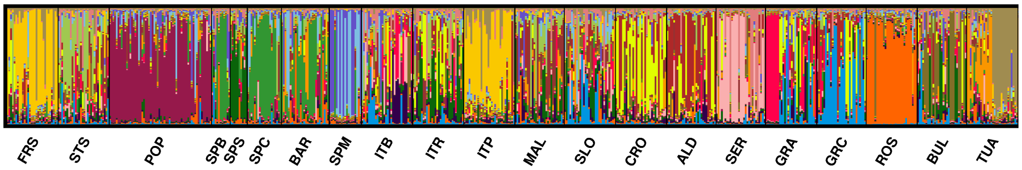

K=19 albo_microsats_overlap_nopops_run_173_f

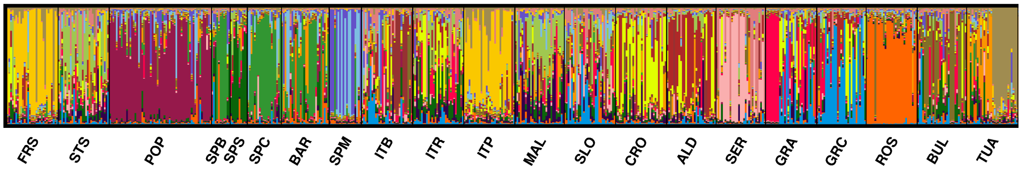

K=19 albo_microsats_overlap_nopops_run_174_f

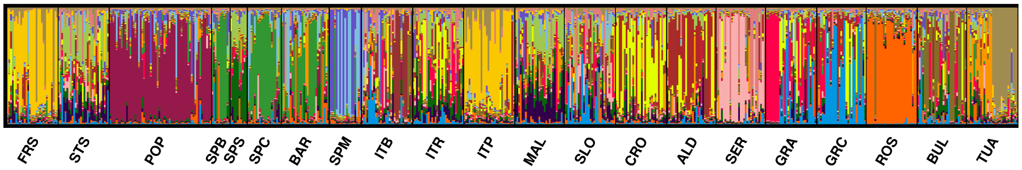

K=19 albo_microsats_overlap_nopops_run_175_f

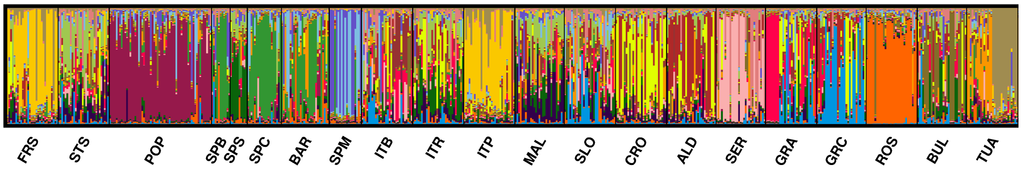

K=19 albo_microsats_overlap_nopops_run_176_f

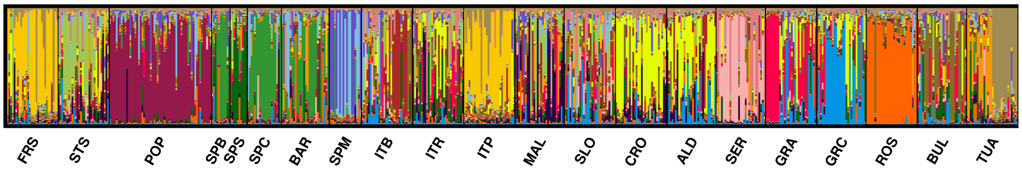

K=19 albo_microsats_overlap_nopops_run_177_f

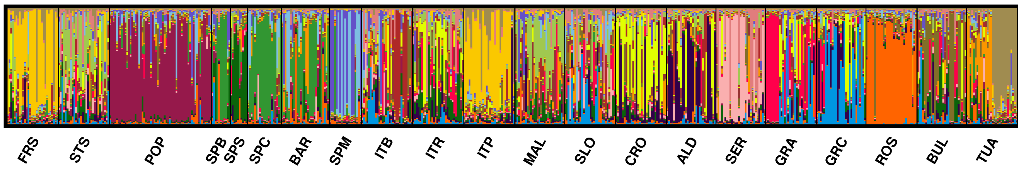

K=19 albo_microsats_overlap_nopops_run_178_f

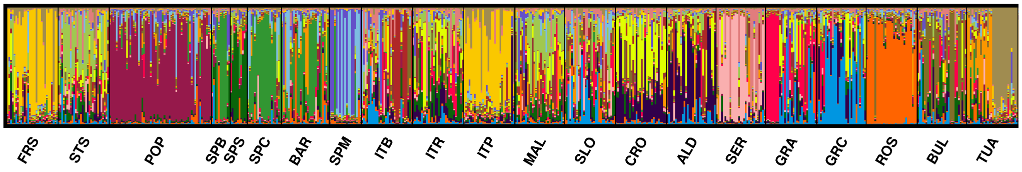

K=19 albo_microsats_overlap_nopops_run_179_f

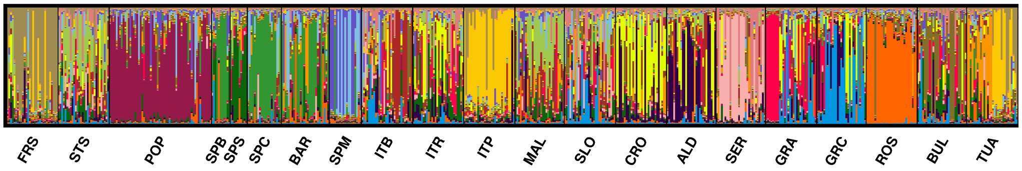

K=19 albo_microsats_overlap_nopops_run_180_f

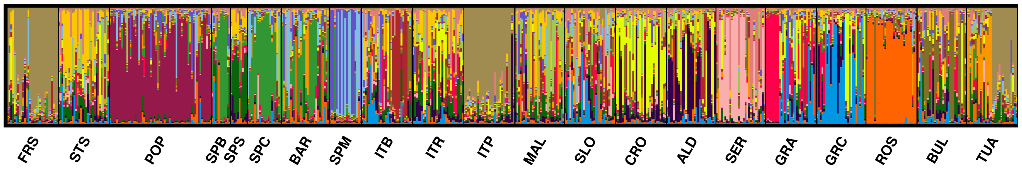

K=20 albo_microsats_overlap_nopops_run_181_f

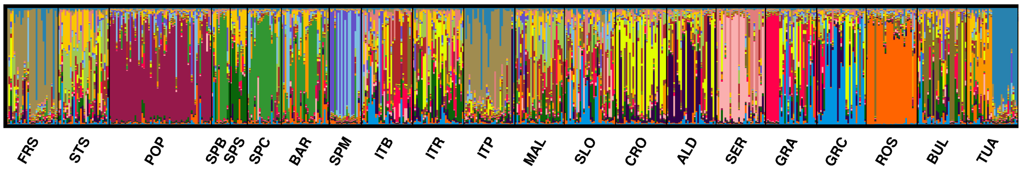

K=20 albo_microsats_overlap_nopops_run_182_f

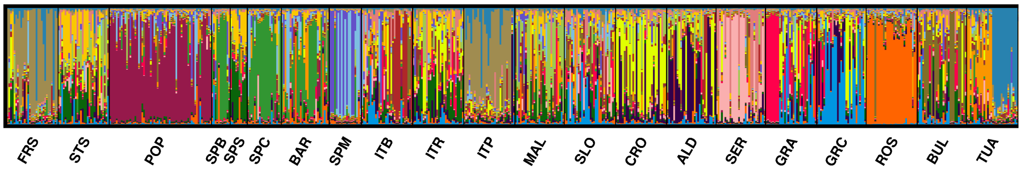

K=20 albo_microsats_overlap_nopops_run_183_f

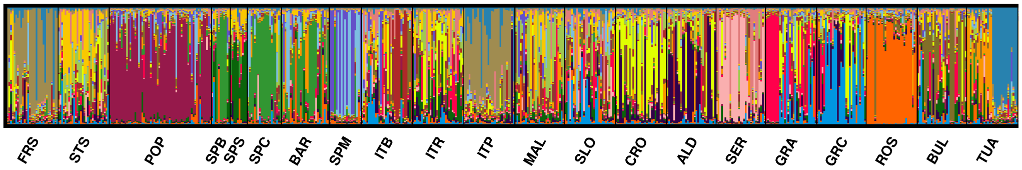

K=20 albo_microsats_overlap_nopops_run_184_f

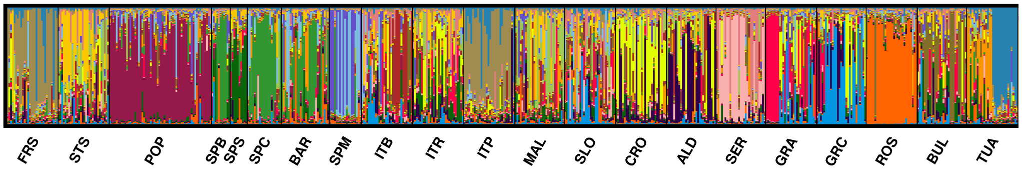

K=20 albo_microsats_overlap_nopops_run_185_f

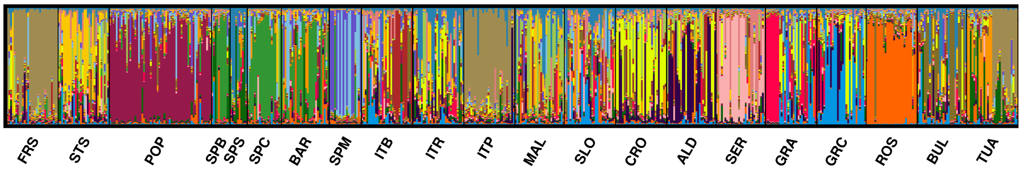

K=20 albo_microsats_overlap_nopops_run_186_f

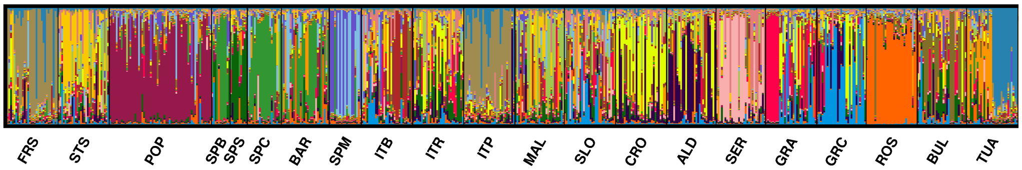

K=20 albo_microsats_overlap_nopops_run_187_f

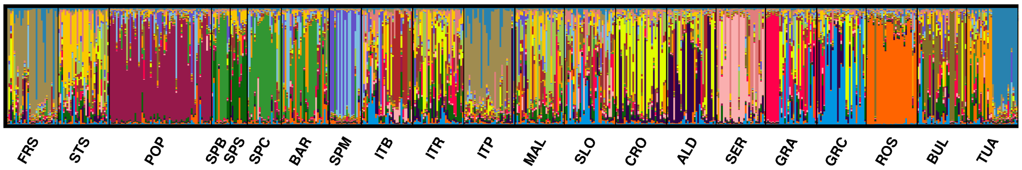

K=20 albo_microsats_overlap_nopops_run_188_f

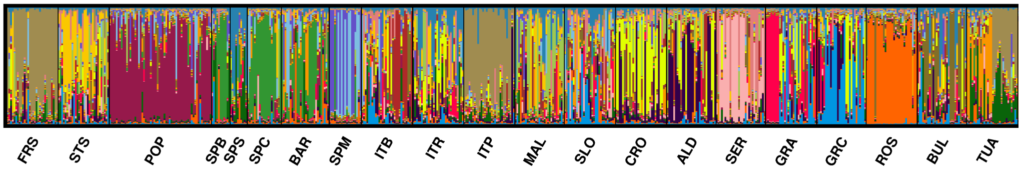

K=20 albo_microsats_overlap_nopops_run_189_f

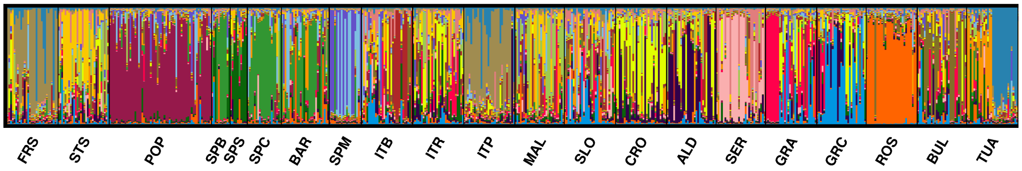

K=20 albo_microsats_overlap_nopops_run_190_f

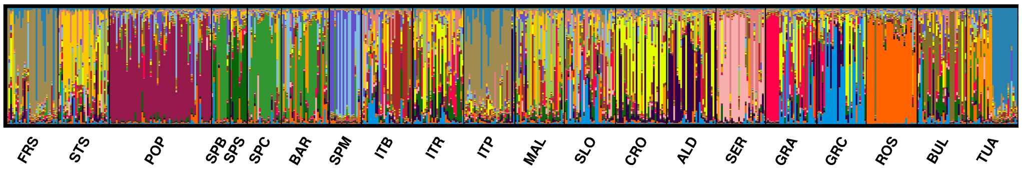

Results from STRUCTURE v 2.3.4 run using an admixture model utilizing only genetic data (NOT population information) to clusters data. Ten repetitions for each K (2-20) were run for the subset of 637 mosquitoes from 24 locations that overlapped with the SNP dataset in Europe using 11 microsatellites.

Job type: *Best K by Evanno*

Output files:

Output Images:

DeltaK graph Optimal K by Evanno is: 3

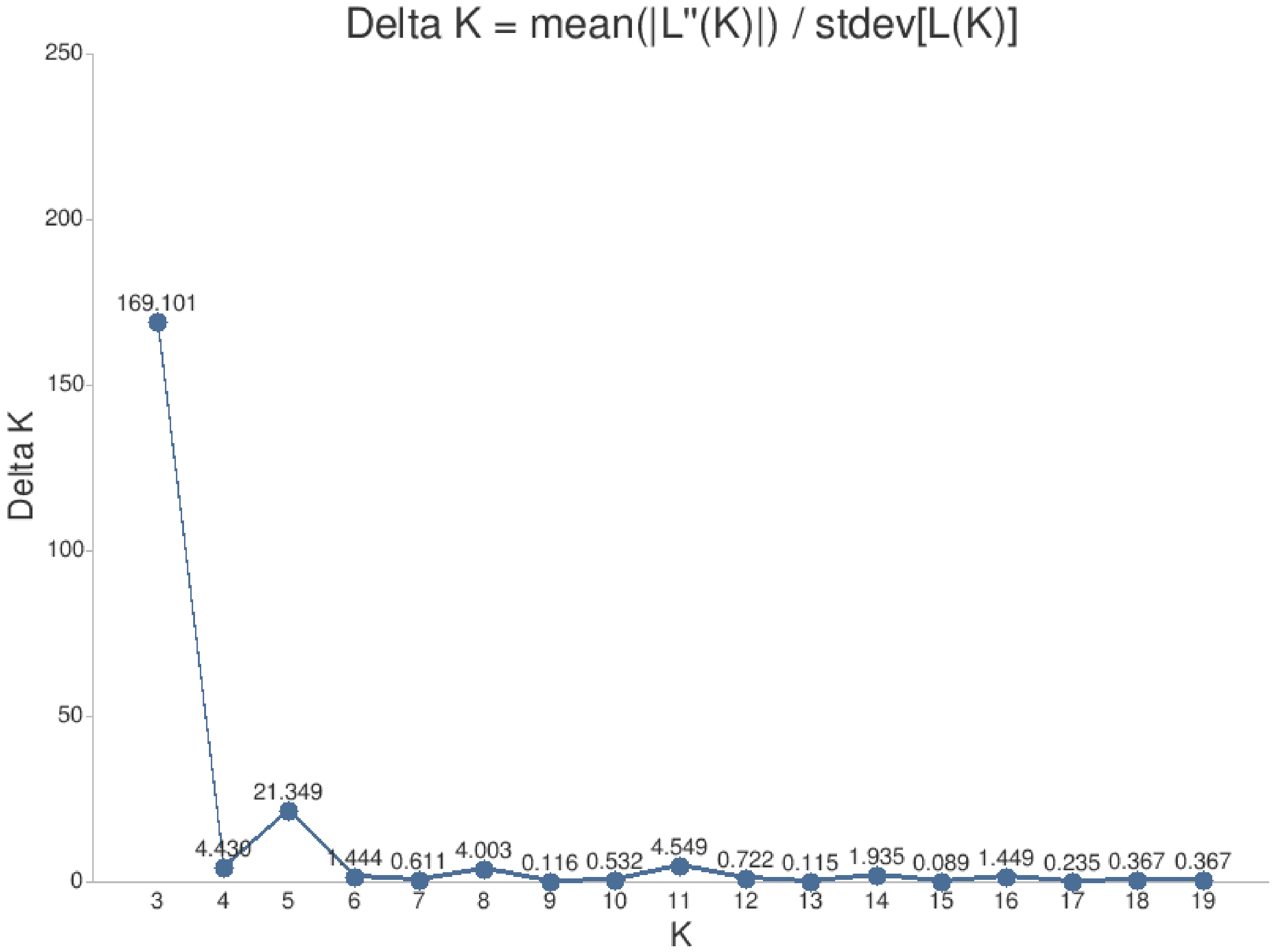

Probability By K graph Using median values of Ln(Pr Data) the k for which Pr(K=k) is highest: 20

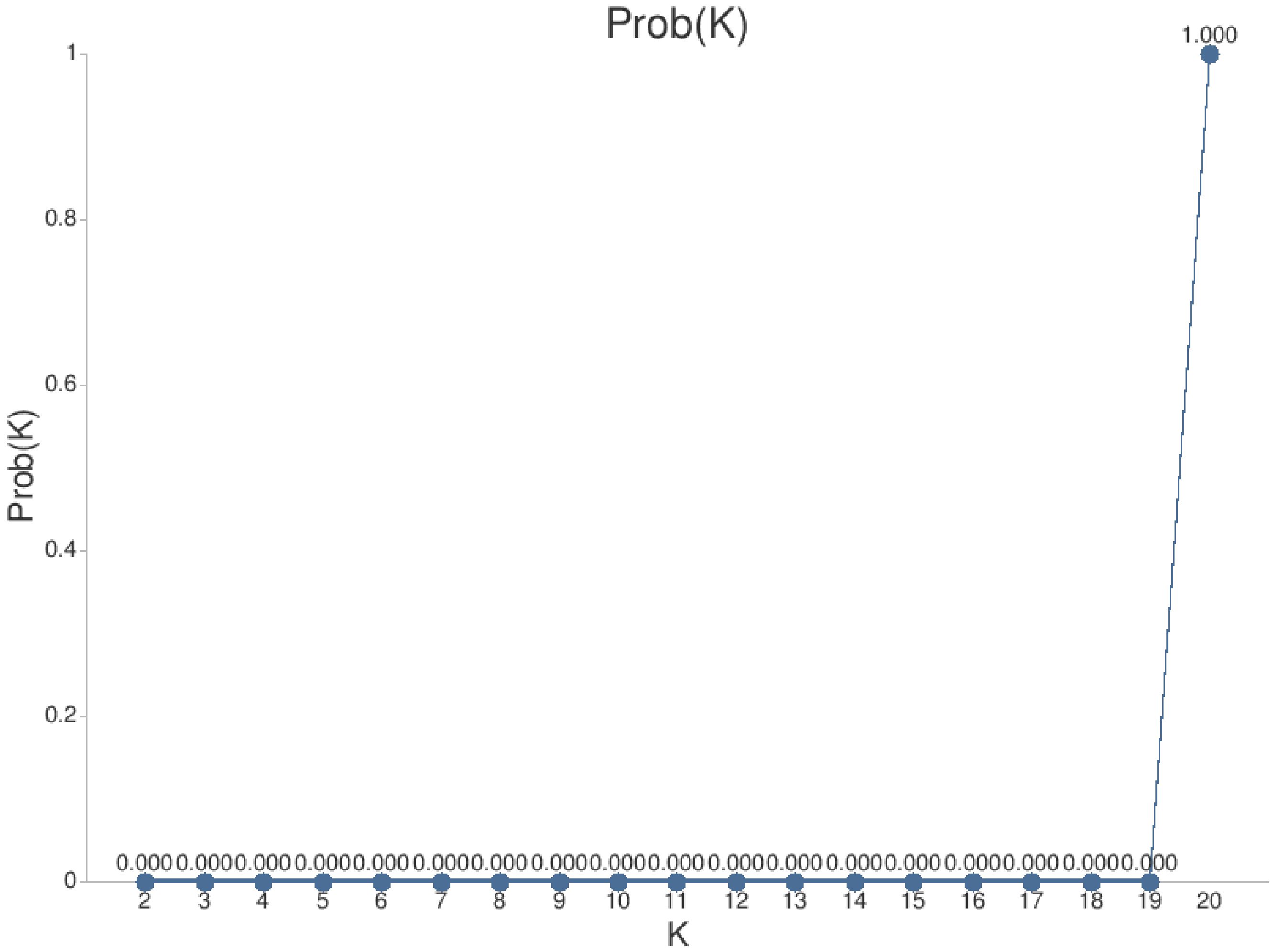

Log Data:

```
Mon May  6 23:03:47 2024: Running validation tests on input files
Mon May  6 23:03:48 2024: Finished validating - Submitting job to queue
Mon May  6 23:03:48 2024: Job 1715025824 started running.
Mon May  6 23:03:49 2024: Retrieving data from files for each K
Mon May  6 23:03:49 2024: K=2 mean: -23738.62
Mon May  6 23:03:49 2024: K=2 standard deviation: 16.2724579854695
Mon May  6 23:03:49 2024: K=2 median: -23731.55
Mon May  6 23:03:49 2024: K=3 mean: -22933.61
```

```
Mon May  6 23:03:49 2024: K=3 standard deviation: 2.12417304170647
Mon May  6 23:03:49 2024: K=3 median: -22933.2
Mon May  6 23:03:49 2024: K=4 mean: -22487.8
Mon May  6 23:03:49 2024: K=4 standard deviation: 21.0781614209798
Mon May  6 23:03:49 2024: K=4 median: -22475.6
Mon May  6 23:03:49 2024: K=5 mean: -22135.36
Mon May  6 23:03:49 2024: K=5 standard deviation: 5.58533794859364
Mon May  6 23:03:49 2024: K=5 median: -22132.95
Mon May  6 23:03:49 2024: K=6 mean: -21902.16
Mon May  6 23:03:49 2024: K=6 standard deviation: 61.7408778687179
Mon May  6 23:03:49 2024: K=6 median: -21876.65
Mon May  6 23:03:49 2024: K=7 mean: -21758.11
Mon May  6 23:03:49 2024: K=7 standard deviation: 229.714656089294
Mon May  6 23:03:49 2024: K=7 median: -21680.6
Mon May  6 23:03:49 2024: K=8 mean: -21473.76
Mon May  6 23:03:49 2024: K=8 standard deviation: 28.7926379479198
Mon May  6 23:03:49 2024: K=8 median: -21481.75
Mon May  6 23:03:49 2024: K=9 mean: -21304.66
Mon May  6 23:03:49 2024: K=9 standard deviation: 69.990939096111
Mon May  6 23:03:49 2024: K=9 median: -21281.2
Mon May  6 23:03:49 2024: K=10 mean: -21127.44
Mon May  6 23:03:49 2024: K=10 standard deviation: 37.3264636536723
Mon May  6 23:03:49 2024: K=10 median: -21124.6
Mon May  6 23:03:49 2024: K=11 mean: -20970.07
Mon May  6 23:03:49 2024: K=11 standard deviation: 21.6065345054063
Mon May  6 23:03:49 2024: K=11 median: -20976.55
Mon May  6 23:03:49 2024: K=12 mean: -20910.98
Mon May  6 23:03:49 2024: K=12 standard deviation: 162.185386381004
Mon May  6 23:03:49 2024: K=12 median: -20831
Mon May  6 23:03:49 2024: K=13 mean: -20734.72
Mon May  6 23:03:49 2024: K=13 standard deviation: 110.572689012955
Mon May  6 23:03:49 2024: K=13 median: -20694.15
Mon May  6 23:03:49 2024: K=14 mean: -20571.17
Mon May  6 23:03:49 2024: K=14 standard deviation: 36.9718526569725
Mon May  6 23:03:49 2024: K=14 median: -20565.55
```

```
Mon May  6 23:03:49 2024: K=15 mean: -20479.17
Mon May  6 23:03:49 2024: K=15 standard deviation: 21.7448870107689
Mon May  6 23:03:49 2024: K=15 median: -20474.4
Mon May  6 23:03:49 2024: K=16 mean: -20385.23
Mon May  6 23:03:49 2024: K=16 standard deviation: 33.0602497133808
Mon May  6 23:03:49 2024: K=16 median: -20378.05
Mon May  6 23:03:49 2024: K=17 mean: -20339.21
Mon May  6 23:03:49 2024: K=17 standard deviation: 68.5203692342653
Mon May  6 23:03:49 2024: K=17 median: -20319.9
Mon May  6 23:03:49 2024: K=18 mean: -20277.07
Mon May  6 23:03:49 2024: K=18 standard deviation: 153.169601350196
Mon May  6 23:03:49 2024: K=18 median: -20230
Mon May  6 23:03:49 2024: K=19 mean: -20158.69
Mon May  6 23:03:49 2024: K=19 standard deviation: 69.9175458824337
Mon May  6 23:03:49 2024: K=19 median: -20128.75
Mon May  6 23:03:49 2024: K=20 mean: -20065.98
Mon May  6 23:03:49 2024: K=20 standard deviation: 86.1120820016946
Mon May  6 23:03:49 2024: K=20 median: -20021.7
Mon May  6 23:03:49 2024: Calculating Best K by Evanno
Mon May  6 23:03:49 2024: Ln'(3) = 805.010000000002
Mon May  6 23:03:49 2024: Ln'(4) = 445.809999999998
Mon May  6 23:03:49 2024: Ln'(5) = 352.439999999995
Mon May  6 23:03:49 2024: Ln'(6) = 233.200000000001
Mon May  6 23:03:49 2024: Ln'(7) = 144.050000000003
Mon May  6 23:03:49 2024: Ln'(8) = 284.350000000002
Mon May  6 23:03:49 2024: Ln'(9) = 169.099999999999
Mon May  6 23:03:49 2024: Ln'(10) = 177.220000000005
Mon May  6 23:03:49 2024: Ln'(11) = 157.369999999995
Mon May  6 23:03:49 2024: Ln'(12) = 59.0900000000001
Mon May  6 23:03:49 2024: Ln'(13) = 176.259999999998
Mon May  6 23:03:49 2024: Ln'(14) = 163.549999999999
Mon May  6 23:03:49 2024: Ln'(15) = 92.0000000000036
Mon May  6 23:03:49 2024: Ln'(16) = 93.9399999999987
Mon May  6 23:03:49 2024: Ln'(17) = 46.0200000000004
Mon May  6 23:03:49 2024: Ln'(18) = 62.1399999999994
```

```
Mon May  6 23:03:49 2024: Ln'(19) = 118.380000000001
Mon May  6 23:03:49 2024: Ln'(20) = 92.7099999999991
Mon May  6 23:03:49 2024: |Ln''(K=3)| = 359.200000000004
Mon May  6 23:03:49 2024: |Ln''(K=4)| = 93.3700000000026
Mon May  6 23:03:49 2024: |Ln''(K=5)| = 119.239999999994
Mon May  6 23:03:49 2024: |Ln''(K=6)| = 89.1499999999978
Mon May  6 23:03:49 2024: |Ln''(K=7)| = 140.299999999999
Mon May  6 23:03:49 2024: |Ln''(K=8)| = 115.250000000004
Mon May  6 23:03:49 2024: |Ln''(K=9)| = 8.12000000000626
Mon May  6 23:03:49 2024: |Ln''(K=10)| = 19.8500000000095
Mon May  6 23:03:49 2024: |Ln''(K=11)| = 98.2799999999952
Mon May  6 23:03:49 2024: |Ln''(K=12)| = 117.169999999998
Mon May  6 23:03:49 2024: |Ln''(K=13)| = 12.7099999999991
Mon May  6 23:03:49 2024: |Ln''(K=14)| = 71.5499999999956
Mon May  6 23:03:49 2024: |Ln''(K=15)| = 1.93999999999505
Mon May  6 23:03:49 2024: |Ln''(K=16)| = 47.9199999999983
Mon May  6 23:03:49 2024: |Ln''(K=17)| = 16.119999999999
Mon May  6 23:03:49 2024: |Ln''(K=18)| = 56.2400000000016
Mon May  6 23:03:49 2024: |Ln''(K=19)| = 25.6700000000019
Mon May  6 23:03:49 2024: Delta(K=3) = 169.101100968421
Mon May  6 23:03:49 2024: Delta(K=4) = 4.4297032428582
Mon May  6 23:03:49 2024: Delta(K=5) = 21.3487529487841
Mon May  6 23:03:49 2024: Delta(K=6) = 1.44393800472939
Mon May  6 23:03:49 2024: Delta(K=7) = 0.610757721725263
Mon May  6 23:03:49 2024: Delta(K=8) = 4.00275932370171
Mon May  6 23:03:49 2024: Delta(K=9) = 0.116015017156091
Mon May  6 23:03:49 2024: Delta(K=10) = 0.531794283653135
Mon May  6 23:03:49 2024: Delta(K=11) = 4.54862393482878
Mon May  6 23:03:49 2024: Delta(K=12) = 0.722444867657461
Mon May  6 23:03:49 2024: Delta(K=13) = 0.114947010093152
Mon May  6 23:03:49 2024: Delta(K=14) = 1.93525600850576
Mon May  6 23:03:49 2024: Delta(K=15) = 0.0892163752809704
Mon May  6 23:03:49 2024: Delta(K=16) = 1.44947483504951
Mon May  6 23:03:49 2024: Delta(K=17) = 0.23525851042755
Mon May  6 23:03:49 2024: Delta(K=18) = 0.367174684168685
```

```
Mon May  6 23:03:49 2024: Delta(K=19) = 0.367146753737123
Mon May  6 23:03:49 2024: Max Delta K: 169.101100968421
Mon May  6 23:03:49 2024: Optimal K by Evanno is: 3
Mon May  6 23:03:49 2024: Using median values of Ln Prob of Data to calculate
Prob(K=k):
Mon May  6 23:03:49 2024: Prob(K=10) = 0
Mon May  6 23:03:49 2024: Prob(K=11) = 0
Mon May  6 23:03:49 2024: Prob(K=12) = 0
Mon May  6 23:03:49 2024: Prob(K=13) = 9.09233951308138e-293
Mon May  6 23:03:49 2024: Prob(K=14) = 6.44089199835849e-237
Mon May  6 23:03:49 2024: Prob(K=15) = 2.48249305617283e-197
Mon May  6 23:03:49 2024: Prob(K=16) = 1.73444835888208e-155
Mon May  6 23:03:49 2024: Prob(K=17) = 3.1144796421669e-130
Mon May  6 23:03:49 2024: Prob(K=18) = 3.43921573869328e-91
Mon May  6 23:03:49 2024: Prob(K=19) = 3.22682722345367e-47
Mon May  6 23:03:49 2024: Prob(K=2) = 0
Mon May  6 23:03:49 2024: Prob(K=20) = 1
Mon May  6 23:03:49 2024: Prob(K=3) = 0
Mon May  6 23:03:49 2024: Prob(K=4) = 0
Mon May  6 23:03:49 2024: Prob(K=5) = 0
Mon May  6 23:03:49 2024: Prob(K=6) = 0
Mon May  6 23:03:49 2024: Prob(K=7) = 0
Mon May  6 23:03:49 2024: Prob(K=8) = 0
Mon May  6 23:03:49 2024: Prob(K=9) = 0
Mon May  6 23:03:49 2024: Max Probability: 1
Mon May  6 23:03:49 2024: The k for which Prob(K=k) obtains the highest value
is: 20
Mon May  6 23:03:49 2024: Creating job zip file
Mon May  6 23:03:49 2024: Job 1715025824 has finished running.
Mon May  6 23:03:49 2024: Job 1715025824 was submitted to queue.
```

Supplementary File 15

Results from STRUCTURE v 2.3.4 run using an admixture model utilizing genetic data AND population information (LOCPRIOR model) to clusters data. Ten repetitions for each K (2-13) were run for the subset of 637 mosquitoes from 24 locations that overlapped with the SNP dataset in Europe using 11 microsatellites.

The admixture plots shown below were created with the CLUMPAK main pipeline. The major mode for each K is shown first, followed by any minor modes for each, and then a summary of the division of runs by mode is provided. Finally, the results of 'Estimating of the Best K' using the Evanno method from CLUMPAK are shown. Additional plots of the major mode for the best K were created in Supplementary File 21.

References:

Evanno, G., Regnaut, S., & Goudet, J. (2005). Detecting the number of clusters of individuals using the software STRUCTURE: a simulation study. *Molecular Ecology*, *14*(8): 2611-2620, doi: https://doi.org/10.1111/j.1365-294X.2005.02553.x

Kopelman, Naama M; Mayzel, Jonathan; Jakobsson, Mattias; Rosenberg, Noah A; Mayrose, Itay. (2015). **CLUMPAK:** a program for identifying clustering modes and packaging population structure inferences across K. *Molecular Ecology Resources* 15(5): 1179-1191, doi: 10.1111/1755-0998.12387

CLUMPAK main pipeline - Job 1715867747 summary

Major modes for the uploaded data:

K=2

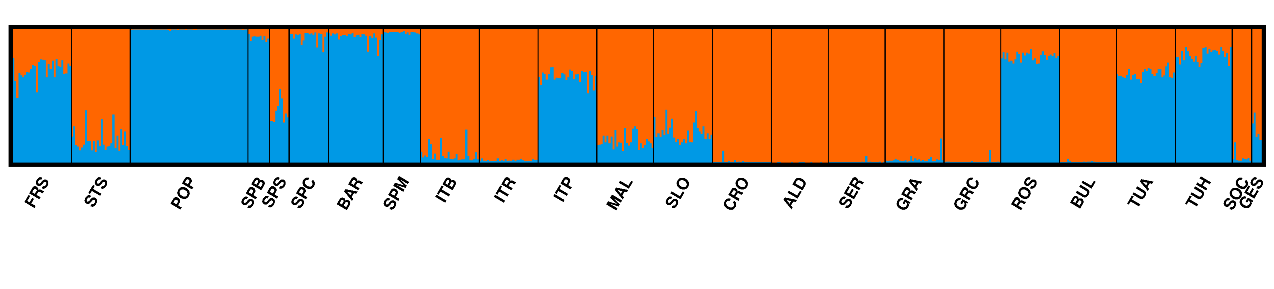

K=3

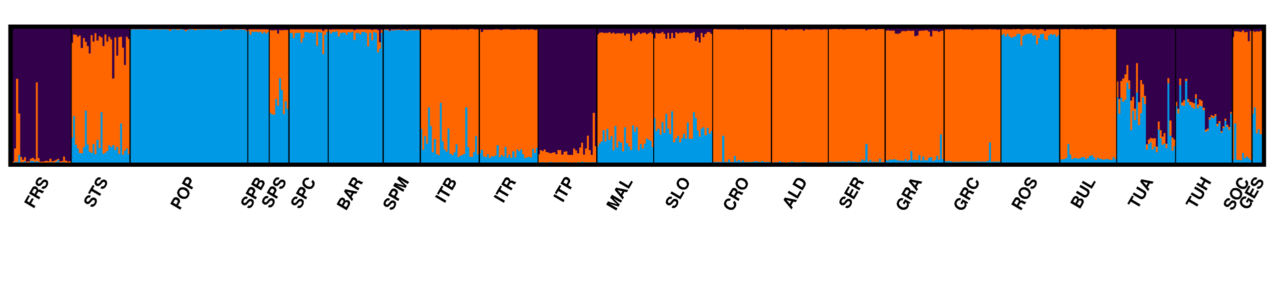

K=4

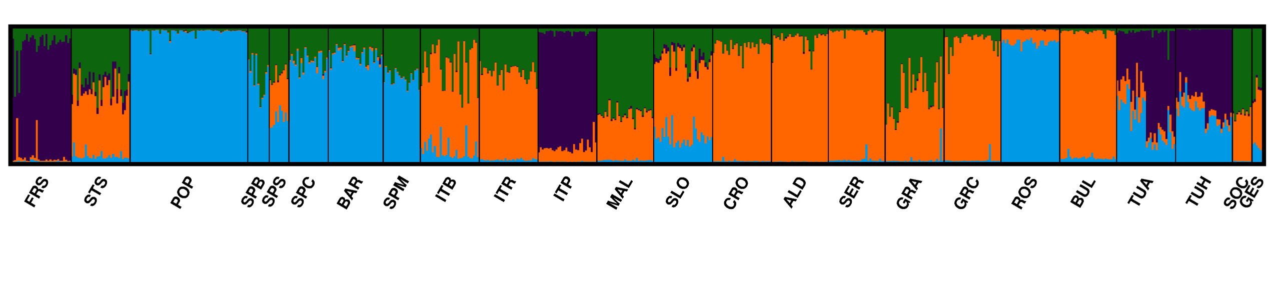

K=5

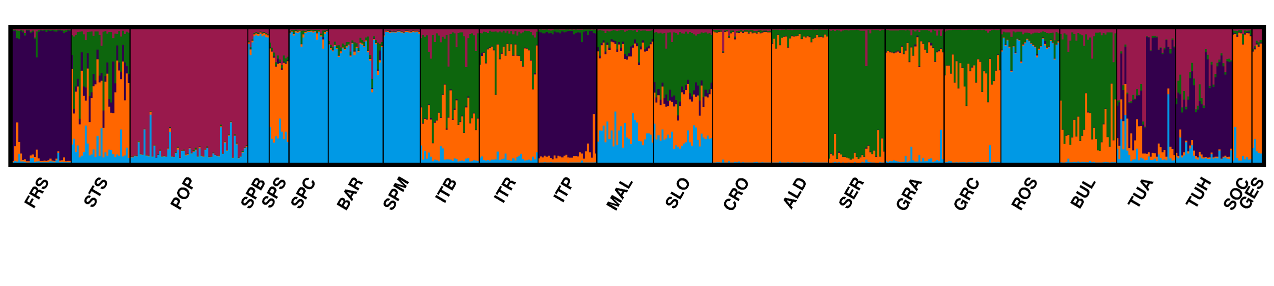

K=6

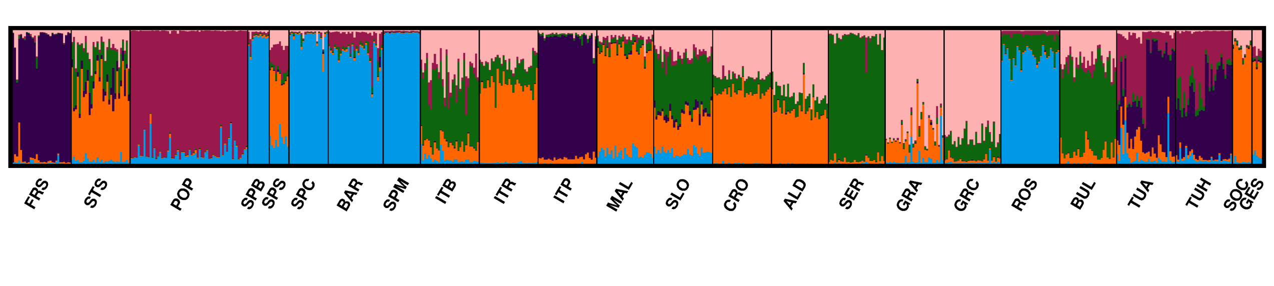

K=7

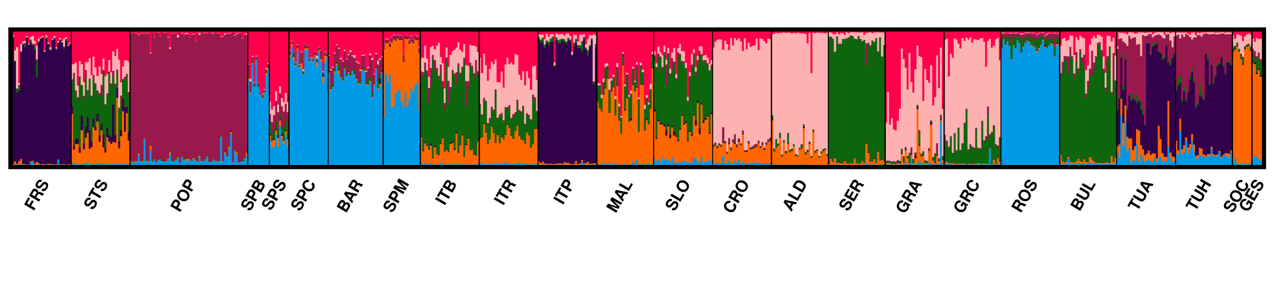

K=8

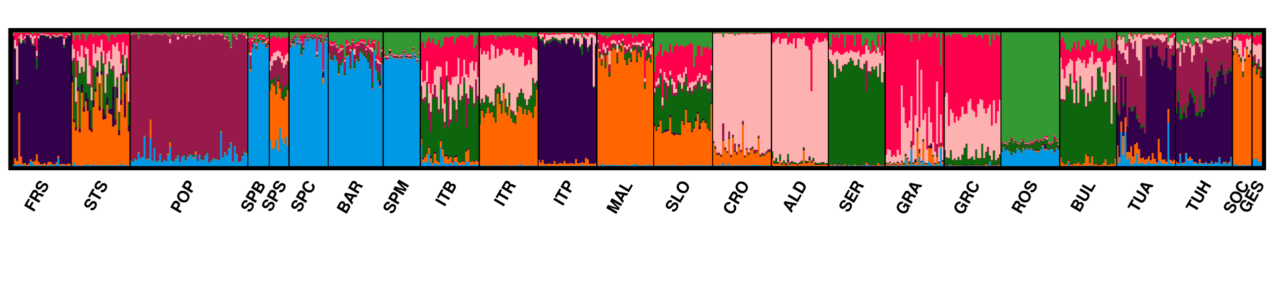

K=9

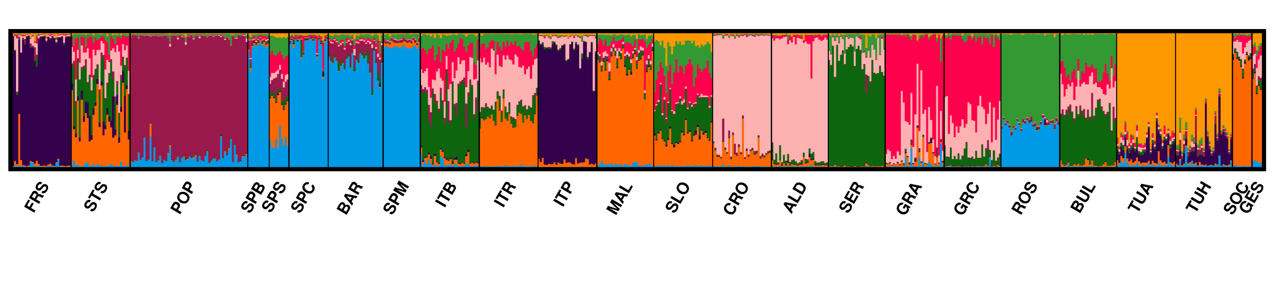

K=10

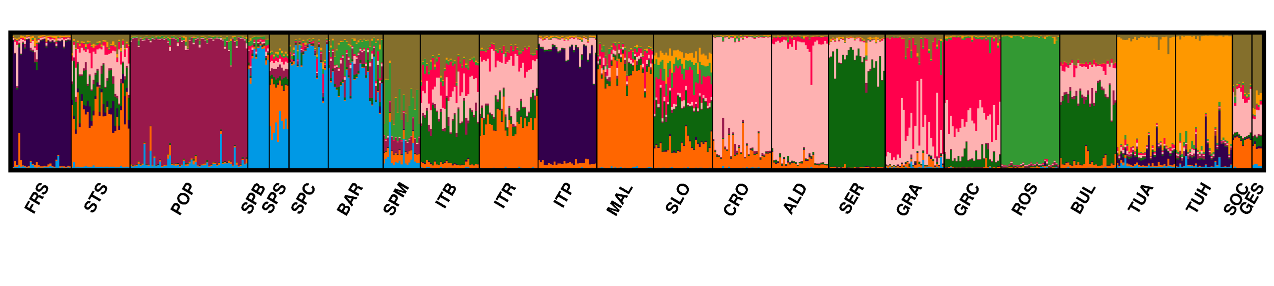

K=11

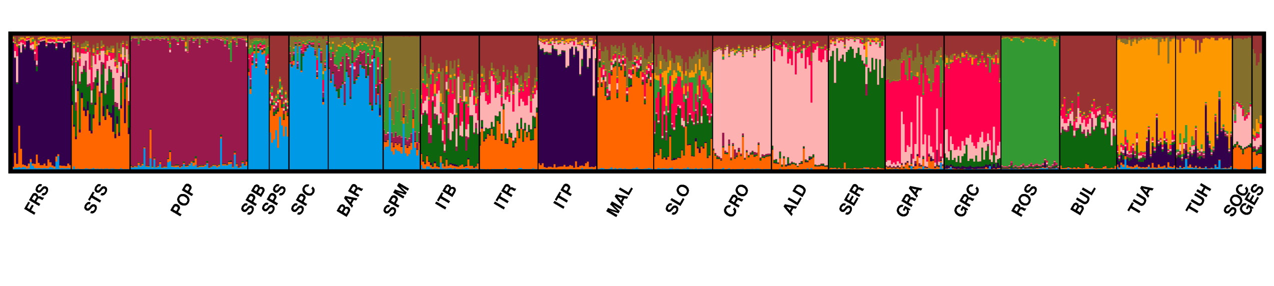

K=12

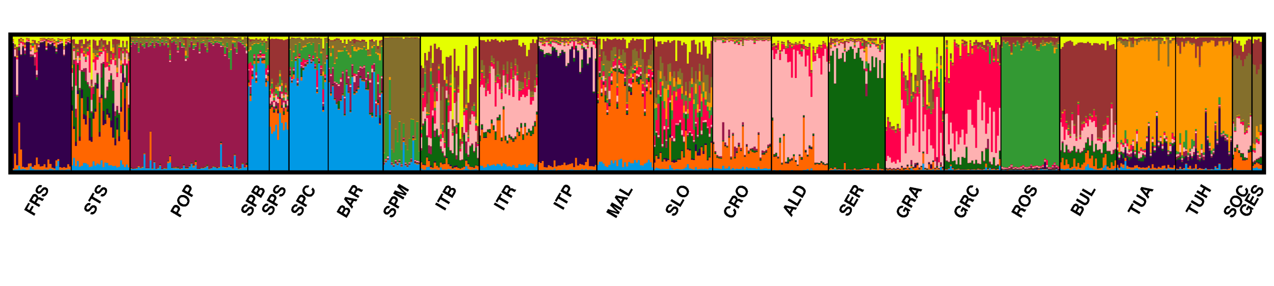

K=13

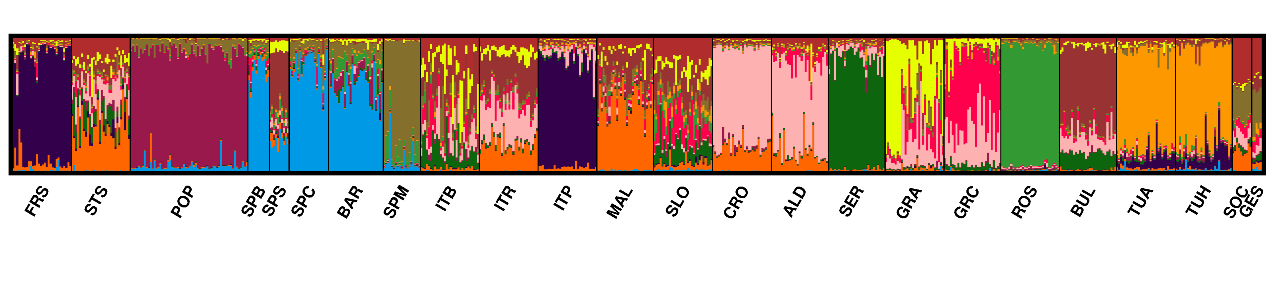

Minor modes for the uploaded data:

K=2 MinorCluster1

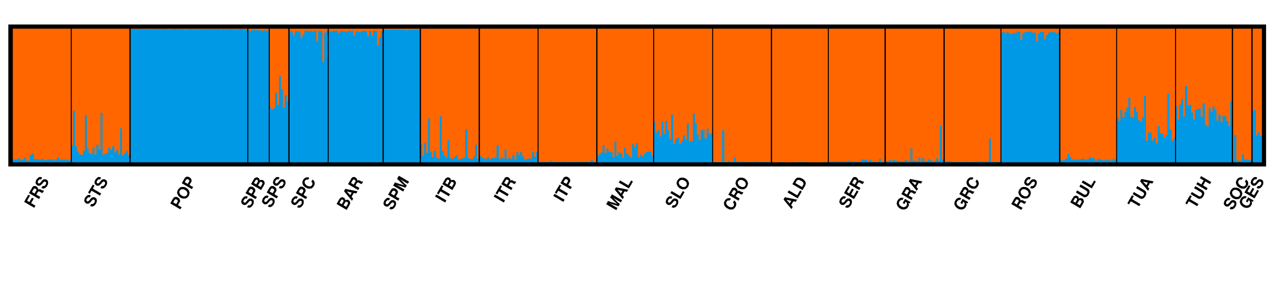

K=4 MinorCluster1

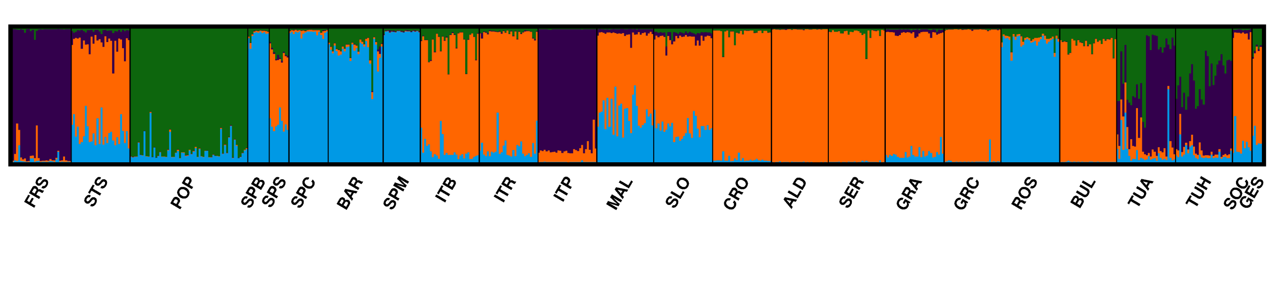

K=4 MinorCluster2

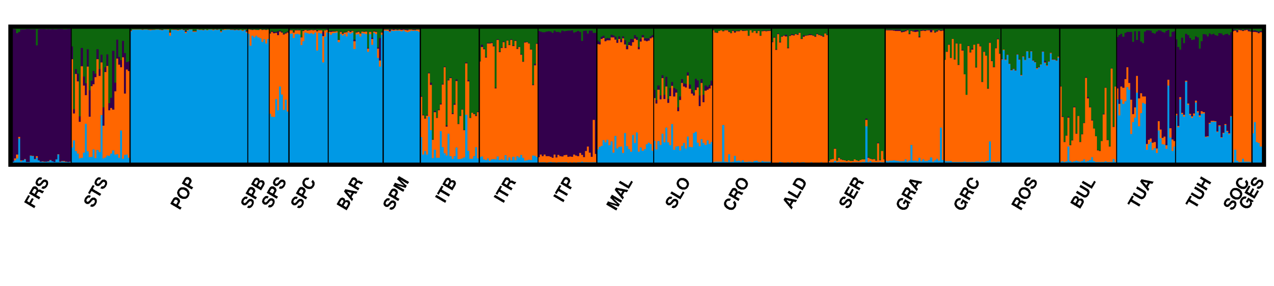

K=5 MinorCluster1

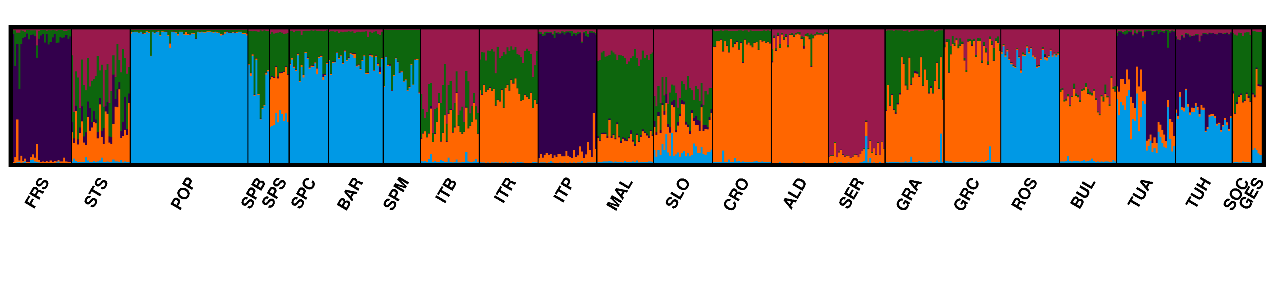

K=5 MinorCluster2

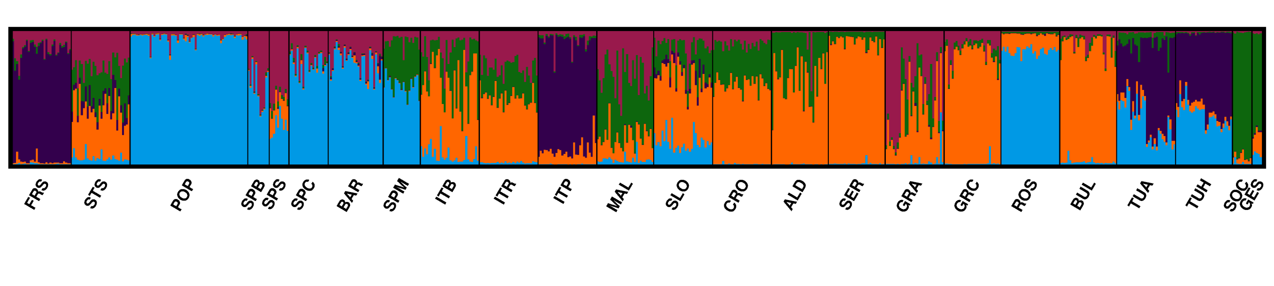

K=6 MinorCluster1

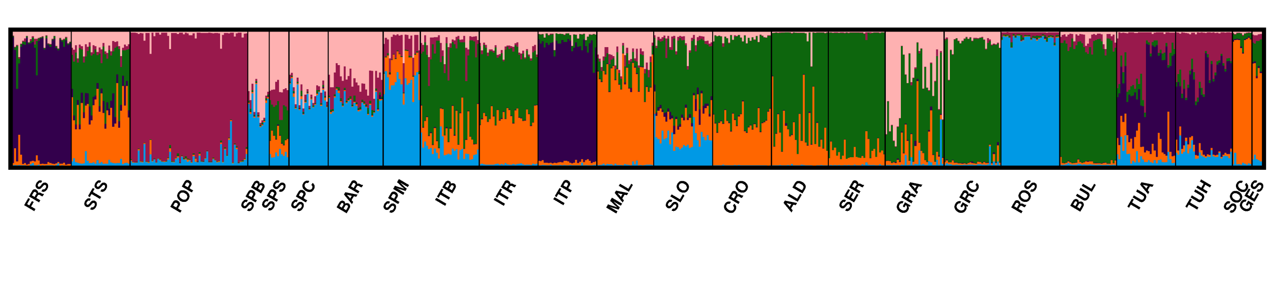

K=6 MinorCluster2

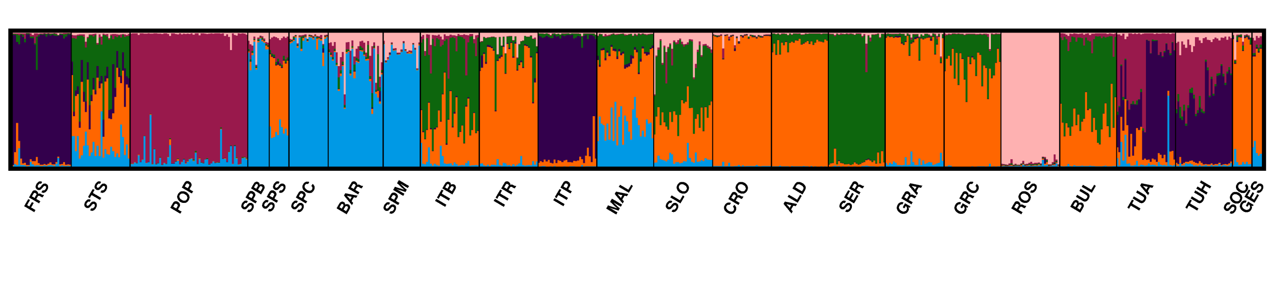

K=7 MinorCluster1

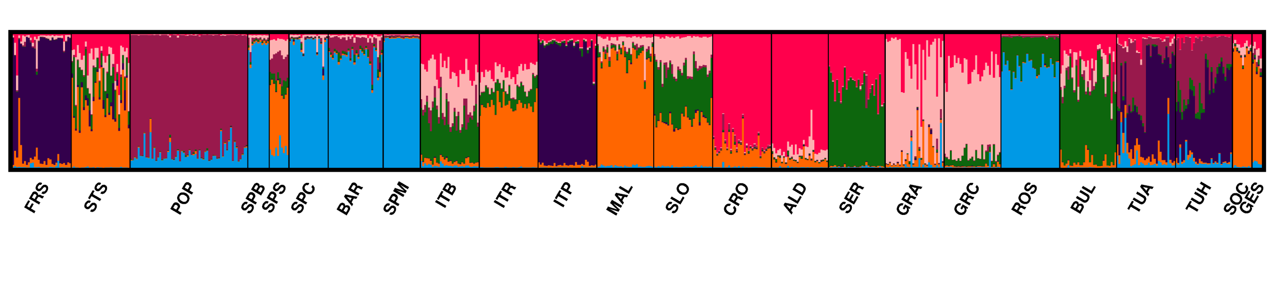

K=7 MinorCluster2

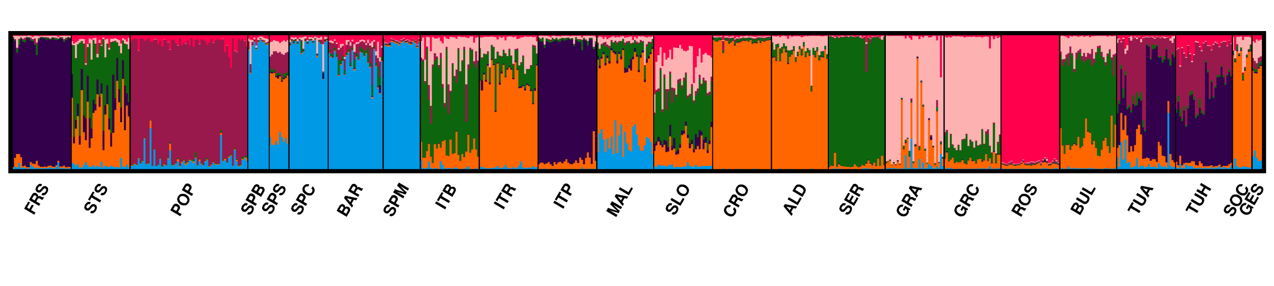

K=8 MinorCluster1

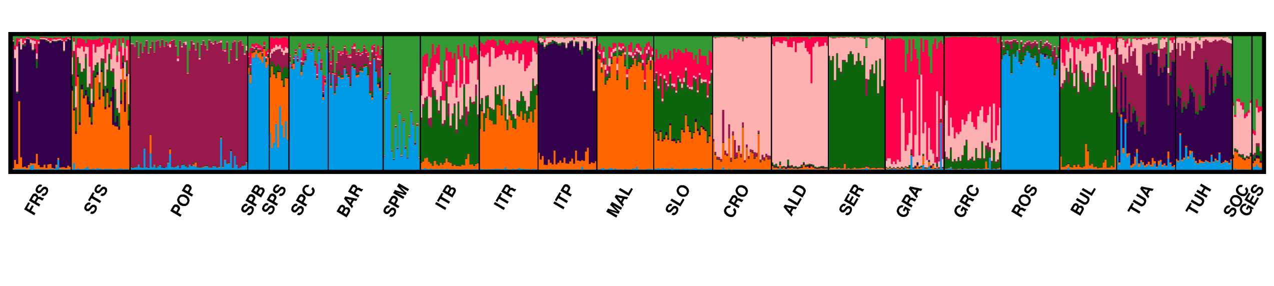

K=8 MinorCluster2

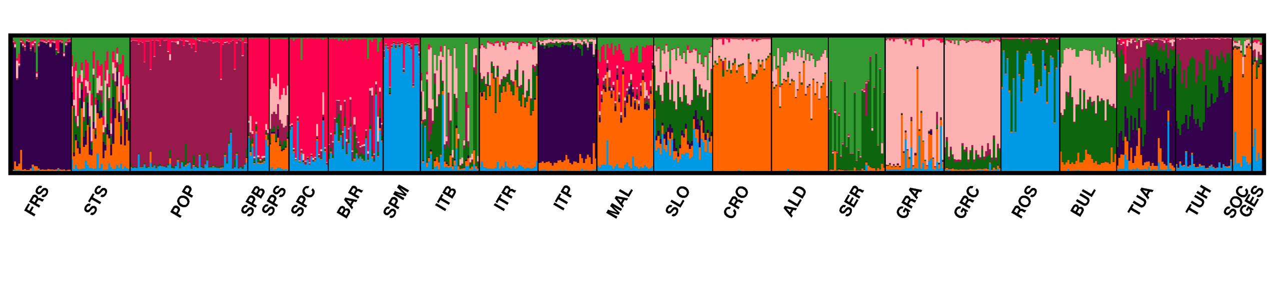

K=9 MinorCluster1

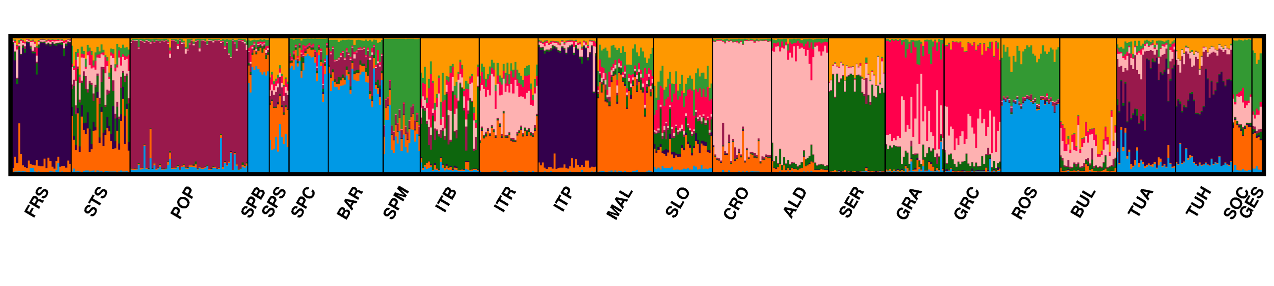

K=10 MinorCluster1

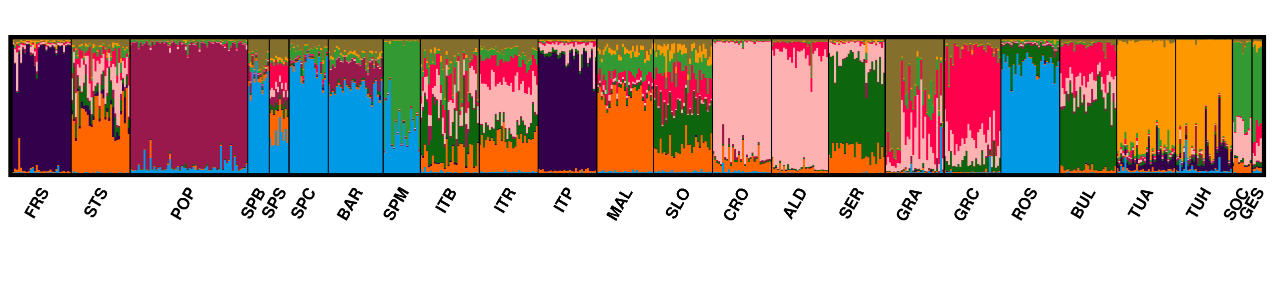

K=10 MinorCluster2

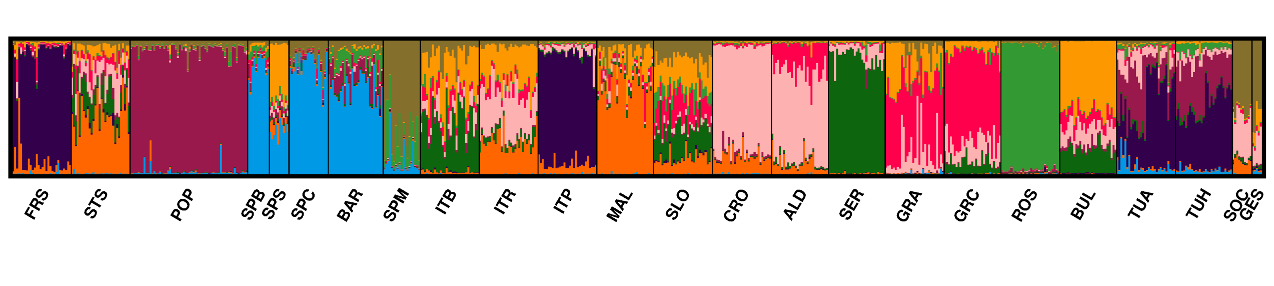

K=11 MinorCluster1

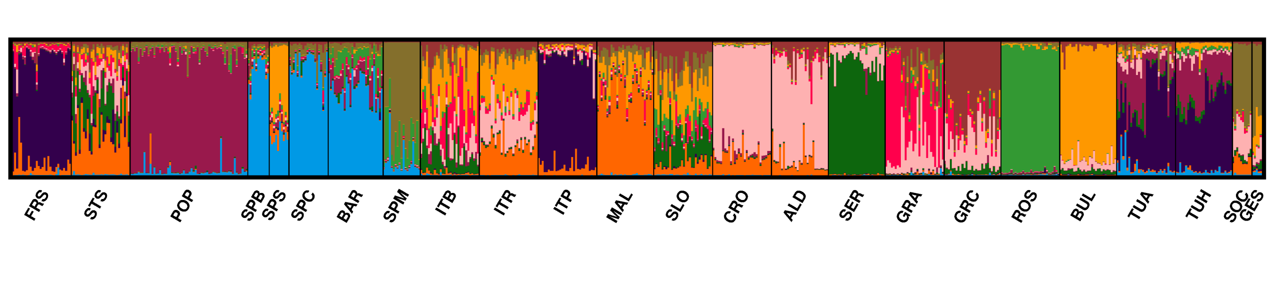

K=11 MinorCluster2

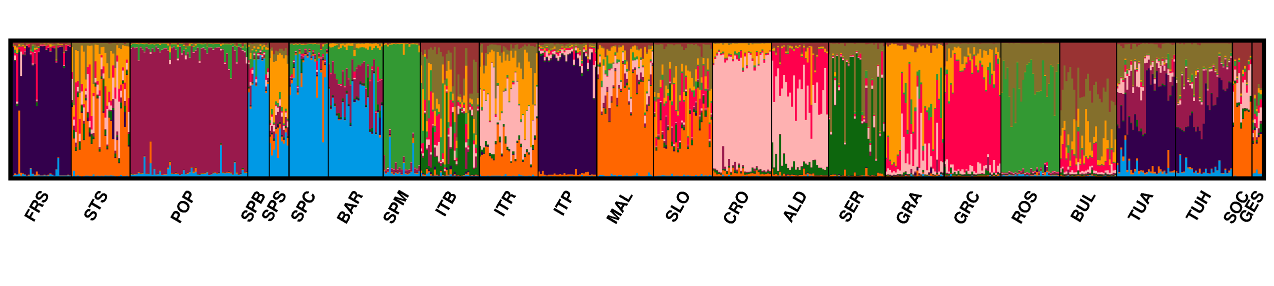

K=12 MinorCluster1

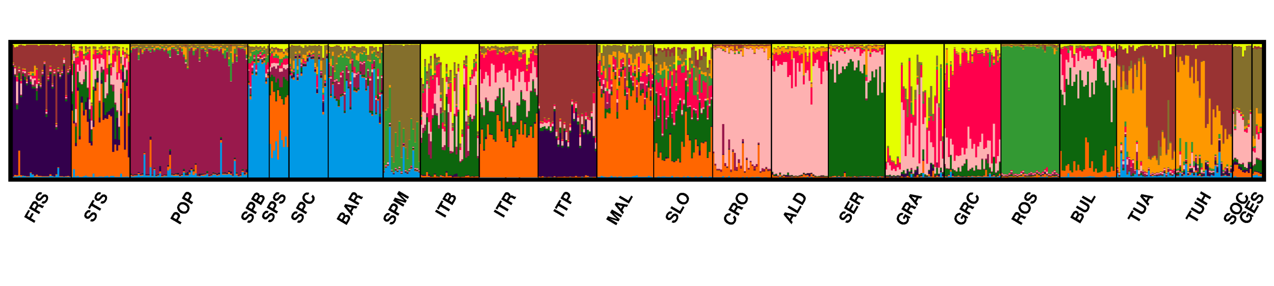

K=13 MinorCluster1

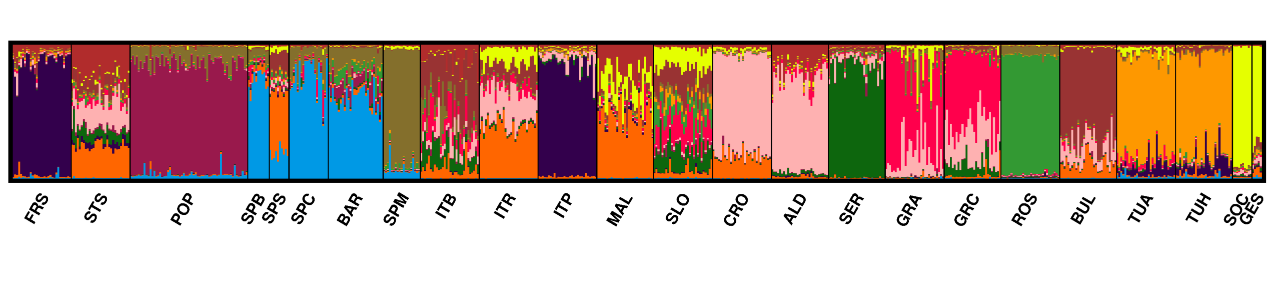

K=13 MinorCluster2

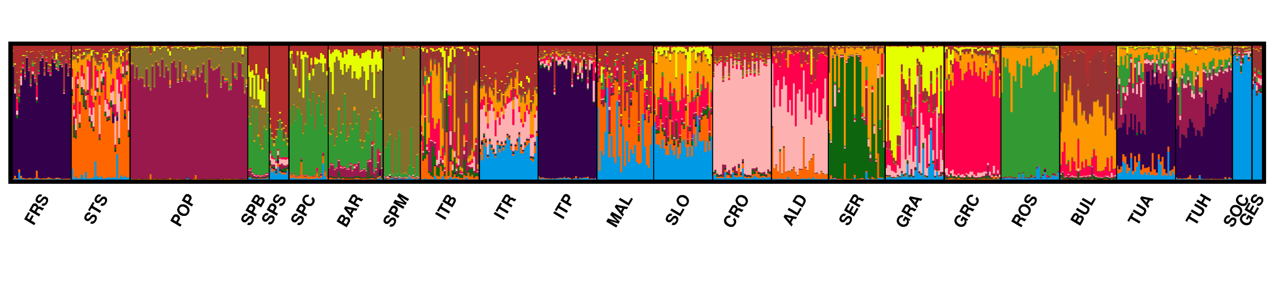

Division of runs by mode:

K=2 6/10, 4/10
K=3 10/10
K=4 7/10, 2/10, 1/10
K=5 6/10, 2/10, 2/10
K=6 7/10, 2/10, 1/10
K=7 5/10, 4/10, 1/10
K=8 6/10, 3/10, 1/10
K=9 5/10, 5/10
K=10 4/10, 4/10, 2/10
K=11 6/10, 3/10, 1/10
K=12 9/10, 1/10
K=13 6/10, 3/10, 1/10

Results from STRUCTURE v 2.3.4 run using an admixture model utilizing genetic data AND population information (LOCPRIOR model) to clusters data. Ten repetitions for each K (2-13) were run for the subset of 637 mosquitoes from 24 locations that overlapped with the SNP dataset in Europe using 11 microsatellites.

Job type: Best *K* by Evanno

Output files:

Output images:

DeltaK graph Optimal K by Evanno is: 3

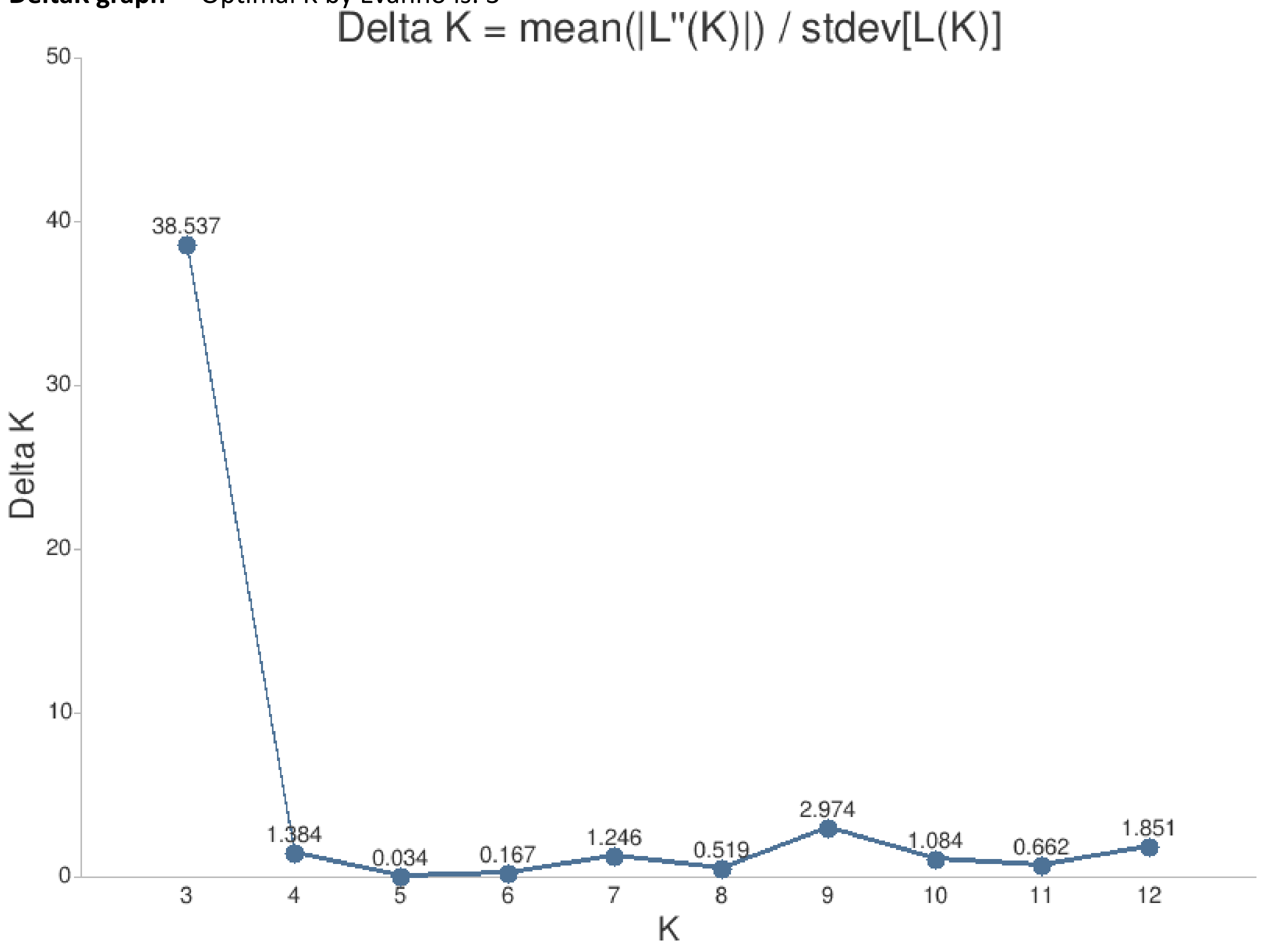

Probability By K graph Using median values of Ln(Pr Data) the k for which Pr(K=k) is highest: 13

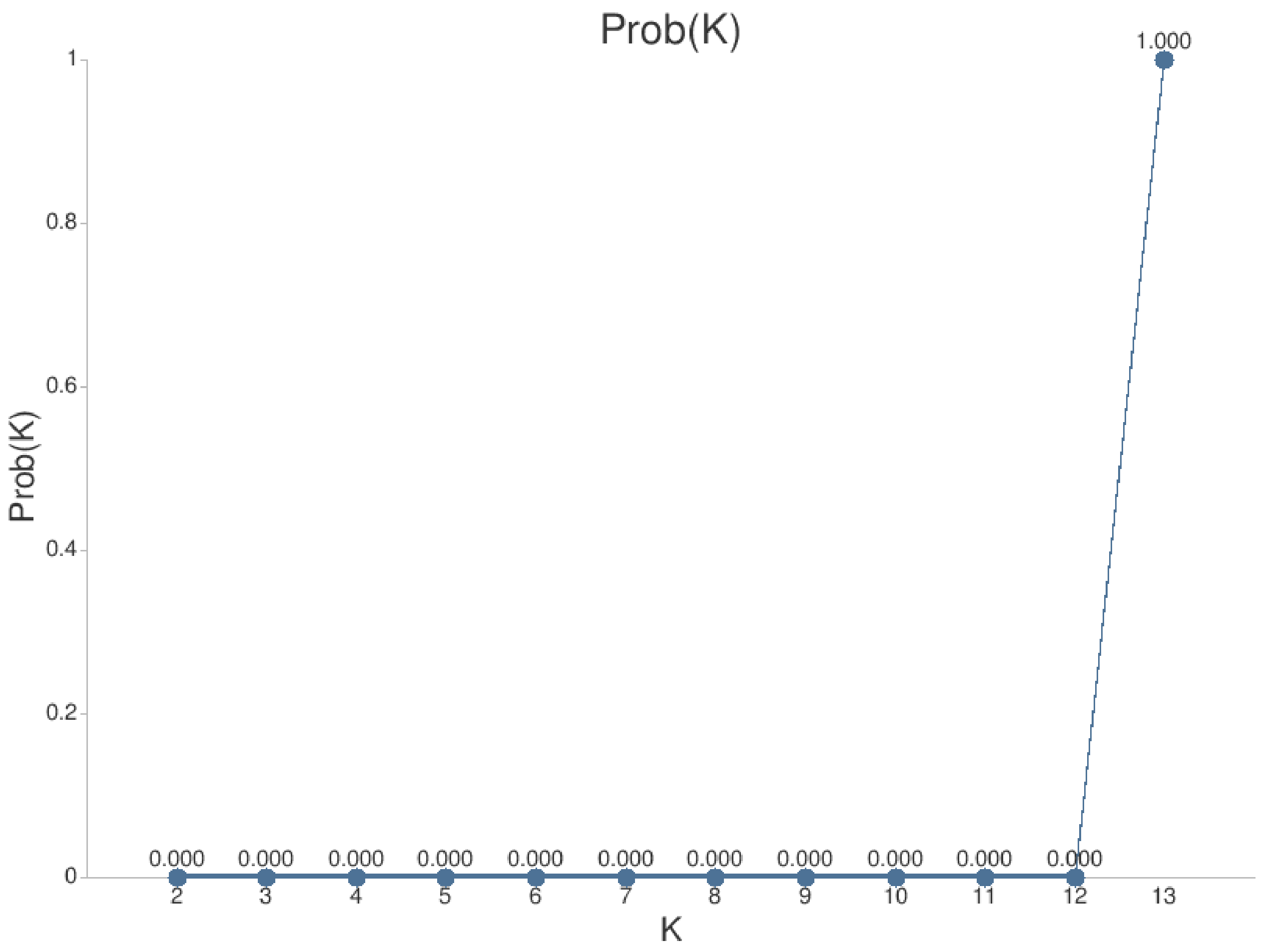

Log Data:

```
Thu May 16 16:50:52 2024: Running validation tests on input files
Thu May 16 16:50:53 2024: Finished validating - Submitting job to queue
Thu May 16 16:50:53 2024: Job 1715867449 started running.
Thu May 16 16:50:53 2024: Retrieving data from files for each K
Thu May 16 16:50:53 2024: K=2 mean: -23688.77
Thu May 16 16:50:53 2024: K=2 standard deviation: 14.4094914707092
Thu May 16 16:50:53 2024: K=2 median: -23692.95
Thu May 16 16:50:53 2024: K=3 mean: -22855.42
Thu May 16 16:50:53 2024: K=3 standard deviation: 13.4331927205208
Thu May 16 16:50:53 2024: K=3 median: -22857.65
Thu May 16 16:50:53 2024: K=4 mean: -22539.74
Thu May 16 16:50:53 2024: K=4 standard deviation: 50.8203415091944
Thu May 16 16:50:53 2024: K=4 median: -22525.3
Thu May 16 16:50:53 2024: K=5 mean: -22294.37
Thu May 16 16:50:53 2024: K=5 standard deviation: 91.7492482560785
Thu May 16 16:50:53 2024: K=5 median: -22292.95
```

```
Thu May 16 16:50:53 2024: K=6 mean: -22045.86
Thu May 16 16:50:53 2024: K=6 standard deviation: 91.7593495084951
Thu May 16 16:50:53 2024: K=6 median: -22050
Thu May 16 16:50:53 2024: K=7 mean: -21782.06
Thu May 16 16:50:53 2024: K=7 standard deviation: 78.3671728326202
Thu May 16 16:50:53 2024: K=7 median: -21801.25
Thu May 16 16:50:53 2024: K=8 mean: -21615.93
Thu May 16 16:50:53 2024: K=8 standard deviation: 82.8765353469311
Thu May 16 16:50:53 2024: K=8 median: -21620.7
Thu May 16 16:50:53 2024: K=9 mean: -21406.76
Thu May 16 16:50:53 2024: K=9 standard deviation: 64.3849568351697
Thu May 16 16:50:53 2024: K=9 median: -21396.55
Thu May 16 16:50:53 2024: K=10 mean: -21389.04
Thu May 16 16:50:53 2024: K=10 standard deviation: 185.395931874341
Thu May 16 16:50:53 2024: K=10 median: -21409.1
Thu May 16 16:50:53 2024: K=11 mean: -21170.32
Thu May 16 16:50:53 2024: K=11 standard deviation: 88.3517062653569
Thu May 16 16:50:53 2024: K=11 median: -21144.2
Thu May 16 16:50:53 2024: K=12 mean: -21010.08
Thu May 16 16:50:53 2024: K=12 standard deviation: 80.3152510769632
Thu May 16 16:50:53 2024: K=12 median: -21016.05
Thu May 16 16:50:53 2024: K=13 mean: -20998.49
Thu May 16 16:50:53 2024: K=13 standard deviation: 210.183451351961
Thu May 16 16:50:53 2024: K=13 median: -20930.6
Thu May 16 16:50:53 2024: Calculating Best K by Evanno
Thu May 16 16:50:53 2024: Ln'(3) = 833.350000000002
Thu May 16 16:50:53 2024: Ln'(4) = 315.679999999997
Thu May 16 16:50:53 2024: Ln'(5) = 245.370000000003
Thu May 16 16:50:53 2024: Ln'(6) = 248.509999999998
Thu May 16 16:50:53 2024: Ln'(7) = 263.800000000003
Thu May 16 16:50:53 2024: Ln'(8) = 166.129999999997
Thu May 16 16:50:53 2024: Ln'(9) = 209.169999999998
Thu May 16 16:50:53 2024: Ln'(10) = 17.7200000000012
Thu May 16 16:50:53 2024: Ln'(11) = 218.720000000001
Thu May 16 16:50:53 2024: Ln'(12) = 160.239999999998
Thu May 16 16:50:53 2024: Ln'(13) = 11.5900000000038
Thu May 16 16:50:53 2024: |Ln''(K=3)| = 517.670000000006
Thu May 16 16:50:53 2024: |Ln''(K=4)| = 70.309999999994
Thu May 16 16:50:53 2024: |Ln''(K=5)| = 3.13999999999578
Thu May 16 16:50:53 2024: |Ln''(K=6)| = 15.2900000000045
Thu May 16 16:50:53 2024: |Ln''(K=7)| = 97.6700000000055
Thu May 16 16:50:53 2024: |Ln''(K=8)| = 43.0400000000009
Thu May 16 16:50:53 2024: |Ln''(K=9)| = 191.449999999997
Thu May 16 16:50:53 2024: |Ln''(K=10)| = 201
Thu May 16 16:50:53 2024: |Ln''(K=11)| = 58.4800000000032
Thu May 16 16:50:53 2024: |Ln''(K=12)| = 148.649999999994
Thu May 16 16:50:53 2024: Delta(K=3) = 38.5366316683006
Thu May 16 16:50:53 2024: Delta(K=4) = 1.38350113187007
Thu May 16 16:50:53 2024: Delta(K=5) = 0.0342237136508391
Thu May 16 16:50:53 2024: Delta(K=6) = 0.166631521277175
Thu May 16 16:50:53 2024: Delta(K=7) = 1.24631266472523
Thu May 16 16:50:53 2024: Delta(K=8) = 0.519326728848283
```

```
Thu May 16 16:50:53 2024: Delta(K=9) = 2.97352067021064
Thu May 16 16:50:53 2024: Delta(K=10) = 1.08416618405756
Thu May 16 16:50:53 2024: Delta(K=11) = 0.661900063642953
Thu May 16 16:50:53 2024: Delta(K=12) = 1.85083154203861
Thu May 16 16:50:53 2024: Max Delta K: 38.5366316683006
Thu May 16 16:50:53 2024: Optimal K by Evanno is: 3
Thu May 16 16:50:53 2024: Using median values of Ln Prob of Data to calculate
Prob(K=k):
Thu May 16 16:50:53 2024: Prob(K=10) = 1.54913907751836e-208
Thu May 16 16:50:53 2024: Prob(K=11) = 1.7167168325886e-93
Thu May 16 16:50:53 2024: Prob(K=12) = 7.75419148370549e-38
Thu May 16 16:50:53 2024: Prob(K=13) = 1
Thu May 16 16:50:53 2024: Prob(K=2) = 0
Thu May 16 16:50:53 2024: Prob(K=3) = 0
Thu May 16 16:50:53 2024: Prob(K=4) = 0
Thu May 16 16:50:53 2024: Prob(K=5) = 0
Thu May 16 16:50:53 2024: Prob(K=6) = 0
Thu May 16 16:50:53 2024: Prob(K=7) = 0
Thu May 16 16:50:53 2024: Prob(K=8) = 1.96507005917833e-300
Thu May 16 16:50:53 2024: Prob(K=9) = 4.37004749640303e-203
Thu May 16 16:50:53 2024: Max Probability: 1
Thu May 16 16:50:53 2024: The k for which Prob(K=k) obtains the highest value is:
13
Thu May 16 16:50:53 2024: Creating job zip file
Thu May 16 16:50:53 2024: Job 1715867449 has finished running.
Thu May 16 16:50:53 2024: Job 1715867449 was submitted to queue.
```
