# Supplementary material for: Population Structure of the Invasive Asian Tiger Mosquito, Aedes albopictus, in Europe
Source: Ecol Evol. 2025 Mar 7;15(3):e71009. doi: 10.1002/ece3.71009 (PMC11886418; doi:10.1002/ece3.71009)
Supplement: Supplementary file 5 — File S3.–S5. [file ECE3-15-e71009-s004.pdf]

S3. Supplementary Methods

A. Mitochondrial COI primers sequences.

Primer sequences used to amplify a 1537 bp fragment of the mitochondrial COI gene for species identification of larval and other unidentified mosquitoes in the SNP datasets.

Name	Sequence	Annealing Temp
COI-Forward	5'-TTT ACA ATT TAT CGC CTA AAC TTC-3'	49.7 °C
COI-Reverse	5'-CAT TGC ACT AAT CTG CCA TA-3'	50.3 °C

B. SNP Set creation and comparison of SNP filtering parameters

The main text reports results for one set of SNPs, which we called SNP Set 3 (filtered for LD half-life r^2<0.01 and MAF >0.01). To explore the impact of using different r^2 values and minor allele frequencies (MAF) on patterns of genomic differentiation and structure, we created two additional SNP Sets for both the Global and European dataset (Table S4). SNP Sets 1 and 2 were filtered to exclude variants with MAF <10%. We performed Linkage Disequilibrium (LD) pruning at two different thresholds, to exclude SNPs with r^2>0.01 for SNP Set 1 and r^2>0.1 for Set 2. The resultant SNP sets contained the following number of variants: r^2<0.01=19,318 (Set 1) and r^2<0.1= 56,384 (Set 2) for the Global dataset; r^2<0.01=17,028 (Set 1) and r^2<0.1=47,484 (Set 2) for the Europe dataset. A summary of the different SNP sets and the number of SNPs retained in each are presented in Tables S4 and S5.

C. Parameters used to run clustering analyses in the software STRUCTURE (v 2.3.4) for data from 11 microsatellite loci.

Parameter Set 1. We used only genetic information to cluster data. These parameters were used to run STRUCTURE for 637 mosquitos in 24 European locations. We ran 10 iterations of K 2-20.

Running Length
Length of Burnin Period: 100000
Number of MCMC Reps after Burnin: 1000000

Ancestry Model Info
Use Admixture Model
* Infer Alpha
* Initial Value of ALPHA (Dirichlet Parameter for Degree of Admixture): 1.0
* Use Same Alpha for all Populations
* Use a Uniform Prior for Alpha
  ** Maximum Value for Alpha: 10.0
  ** SD of Proposal for Updating Alpha: 0.025

Frequency Model Info
Allele Frequencies are Correlated among Pops
* Assume Different Values of Fst for Different Subpopulations

* Prior Mean of Fst for Pops: 0.01
* Prior SD of Fst for Pops: 0.05
* Use Constant Lambda (Allele Frequencies Parameter)
* Value of Lambda: 1.0

Advanced Options
Estimate the Probability of the Data Under the Model
Frequency of Metropolis update for Q: 10

Parameter Set 2. We used sampling locations as prior information in addition to genetic information to cluster data. These parameters were used to run 10 iterations of K 2-13 for the 24 European locations in the microsatellite dataset.

Running Length
Length of Burnin Period: 100000
Number of MCMC Reps after Burnin: 1000000

Ancestry Model Info
Use Admixture Model
* Use Sampling Location Information
* Use Population IDs as Sampling Location Information
* Infer Alpha
* Initial Value of ALPHA (Dirichlet Parameter for Degree of Admixture): 1.0
* Use Same Alpha for all Populations
* Use a Uniform Prior for Alpha
** Maximum Value for Alpha: 10.0
** SD of Proposal for Updating Alpha: 0.025

Frequency Model Info
Allele Frequencies are Correlated among Pops
* Assume Different Values of Fst for Different Subpopulations
* Prior Mean of Fst for Pops: 0.01
* Prior SD of Fst for Pops: 0.05
* Use Constant Lambda (Allele Frequencies Parameter)
* Value of Lambda: 1.0

Advanced Options
Estimate the Probability of the Data Under the Model
Frequency of Metropolis update for Q: 10

D. Methods for microsatellite loci

Genomic DNA used for microsatellites genotyping was extracted from individual adults or larvae mosquitoes using different methods. Most individuals were extracted using the CTAB method (Weeks et al., 2000). The protocol of Collins et al. (1988) was used in samples from Penafiel (Portugal), Beceite (Spain) and Benicassim (Spain). Finally, subsamples of 10-15 specimens per collection site were extracted by commercial DNA extraction kits (NZY Tissue gDNA Isolation; Qiagen DNeasy Blood and Tissue).

Eleven microsatellites were genotyped following primers and protocols from Manni et al. (2017) with minor modifications (**Supplementary Table C**). Briefly, each locus was amplified individually in a PCR reaction containing PCR GoTaq®PCR buffer (Promega), 1.5 mM $MgCl_2$, 0.3 mM of an equimolar dNTP mix, 0.2 µM of each forward and reverse primer, 1 U of GoTaq© DNA polymerase (Promega), and 1 µl (*ca.* 5ng) of DNA template, in a total volume of 20 µl. Each forward primer was labelled with one of three different fluorescent dyes (FAM, NED, HEX). Thermocycling conditions included an initial denaturing step at 94°C for 3 min, followed by 30 cycles each with denaturation at 94°C for 30 sec, annealing at 57-60°C (primer dependent) doe 30 sec and elongation at 72°C for 30 sec. A final elongation step at 72°C for 10 min concluded the cycling program. PCR products were multiplexed in groups of 2 or three according to fluorescent dye and size interval and subjected to fragment analysis on an ABI 3130xl DNA analyzer (Applied Biosystems) at the Yale DNA Analysis Facility at Science Hill. At least three positive controls were used to account for inter-run variations in fragment size. Alleles were scored from electropherograms using the software GENEMARKER (SoftGenetics).

References

Collins F.H. *et al.* (1988). Comparison of DNA-probe and isoenzyme methods for differentiating *Anopheles gambiae* and *Anopheles arabiensis* (Diptera: Culicidae*). J Med Entomol.* **25**: 116–120.

Manni M. *et al.* (2017). Genetic evidence for a worldwide chaotic dispersion pattern of the arbovirus vector*, Aedes albopictus*. *PLoS Negl Trop Dis.* **11**: e0005332.

Weeks A.R. *et al.* (2000). AFLP fingerprinting for assessing intraspecific variation and genome mapping in mites. *Exp Appl Acarol* **24**: 775-793.

Supplementary Table C. Locus names, repeat motifs, primer sequences, and annealing temperatures (Ta) for the 11 microsatellite loci used in the study.

Locus	Repeat motif	Primer sequences	Ta
**Aealbmic2**	(GTT)5TGGAGTGAG(GTT)6	F: [6FAM]ACGATGCGTAACCATTCGAT	58
		R: AACACCGCCGAATATGAAAC	
**Aealbmic3**	(AAC)9	F: [HEX]ACCATACAGCCTGGAGTTCG	58
		R: GGGGTTGTGTGAATTGTCGT	
**Aealbmic4**	(CAA)8	F: [6FAM]ATCGCGGGTTTTCTATTCCT	58
		R: ATCAACGAAACCGAAAGCAT	
**Aealbmic5**	(TGT)13	F: [HEX]AACCCATCGAACACAGAAGG	58
		R: GTACGGTTGACTCGCTGTGA	
**Aealbmic6**	(GTT)3GCT(GTT)3 GGT(GTT)4	F: [NED]GATGGTCCGTATTTGGGTTG	57
		R: ATCTTCACTCATCCGCCATC	
**Aealbmic7**	(TTG)8ATG(TTG)4	F: [HEX]ATAGACGGGAGTCGGTTCCT	58
		R: TCCAACCGCTAGTGTCATCA	
**Aealbmic8**	(GT)8	F: [HEX]TTGTTGTTCGGTTGTTGTTTG	57
		R: CGGGTTCCAACTATGTACGA	
**Aealbmic9**	(GAT)7	F: [HEX]GCGATGACAGTGGAACAAGA	58
		R: GCTTGGCAGGGAACAAATTA	
**Aealbmic10**	(ATC)9	F: [6FAM]ATCGCCTTCACTCTTCTTCG	60
		R: CCAATCCTGAGCCGTACATT	
**Aealbmic11**	(TGT)5	F: [6FAM]CTCTGCGTTCCGGTTCTATC	58
		R: AGGCAACCTCTCGAATGAAA	
**Aealbmic12**	(GAT)7	F: [6FAM]AGAGCCCTCGAAAAGAGAGC	58

S4. European dataset SNP genotype calls

Analysis Summary

- **Batch Name:** albo_europe_7Sep2023
- **Array Package Name:** Axiom_Aealbo.r1
- **Array Display Name:** Axiom_Aealbo.r1
- **Workflow Type:** Best Practices Workflow
- **Date Created:** 9/7/2023 1:52:02 PM

Sample Summary

- Number of input samples: 454
- Samples passing DQC: 429 out of 454
- Samples passing DQC and QC CR: 425 out of 454
- Samples passing DQC, QC CR and Plate QC: 425 out of 454 (93.612%)
- Number of failing samples: 29
- Number of Samples Genotyped: 425
- Average QC CR for the passing samples: 98.782
- Inbred Penalty Applied: no
- Prior Model File: Axiom_Aealbo.r1.generic_prior.txt
- SNP List File: none

Plate QC Summary

Plate Barcode	Result	Number of files in a batch	Number of files failing dish QC	Number of files failing QC Call rate	Number of samples that passed	Percent of passing samples	Average call rate for passing samples	Filtered Call Rate
5513584436722042923427	PASSED	21	0	0	21	100	99.12	98.163
5513584436722042923430	PASSED	8	0	0	8	100	98.959	97.994
5513584436722042923435	PASSED	3	0	0	3	100	99.252	98.141
5513584452328010824440	PASSED	42	2	0	40	95.238	98.706	97.924
5513584452328010824443	PASSED	95	3	0	92	96.842	98.744	98.039
5513584452328010824444	PASSED	24	0	0	24	100	99.195	98.61
5513584452328010824445	PASSED	63	0	0	63	100	99.247	98.607
5513584452328010824446	PASSED	95	4	0	91	95.789	98.851	98.13
5513584452328010824447	PASSED	51	12	0	39	76.471	99.162	98.493
5513584452328010824449	PASSED	36	3	2	31	86.111	97.389	96.513
5513584478174020425867	PASSED	10	0	2	8	80	96.436	95.454
5513584478174020425873	PASSED	6	1	0	5	83.333	98.451	97.498

ProbeSet Metrics Summary

- Number of ProbeSets: 175396

ConversionType	Count	Percentage
PolyHighResolution	103048	58.752
Other	42824	24.416
OTV	22994	13.11
NoMinorHom	3679	2.098
MonoHighResolution	1961	1.118
CallRateBelowThreshold	890	0.507

Marker Metrics Summary

- Number of Markers: 175396

- Number of BestandRecommended: 108688
- Percent BestandRecommended: 61.967

ConversionType	Count	Percentage
PolyHighResolution	103048	58.752
Other	42824	24.416
OTV	22994	13.11
NoMinorHom	3679	2.098
MonoHighResolution	1961	1.118
CallRateBelowThreshold	890	0.507

Sample QC Thresholds

- DQC: ≥ 0.82
- QC call_rate: ≥ 90
- Average call rate for passing samples: ≥ 90
- Percent of passing samples: ≥ 90

SNP QC Thresholds

- species-type: Diploid
- cr-cutoff: ≥ 90
- fld-cutoff: ≥ 3.6
- het-so-cutoff: ≥ -0.1
- het-so-XChr-cutoff: ≥ -0.1
- het-so-ZChr-cutoff: ≥ -0.1
- het-so-otv-cutoff: ≥ -0.3
- hom-ro-1-cutoff: ≥ 0.6
- hom-ro-2-cutoff: ≥ 0.3
- hom-ro-3-cutoff: ≥ -0.9
- hom-ro: true
- num-minor-allele-cutoff: ≥ 2
- hom-ro-hap-1-XChr-cutoff: ≥ 0.1
- hom-ro-hap-1-MTChr-cutoff: ≥ 0.4
- hom-ro-hap-1-ZChr-cutoff: ≥ 0.1
- hom-ro-hap-2-XChr-cutoff: ≥ 0.05
- hom-ro-hap-2-MTChr-cutoff: ≥ 0.2
- hom-ro-hap-2-ZChr-cutoff: ≥ 0.05
- aaf-XChr-cut: < 0.36
- aaf-ZChr-cut: < 0.36
- fld-XChr-cut: ≥ 4
- fld-ZChr-cut: ≥ 4
- homfld-XChr-cut: ≥ 6.5
- homfld-ZChr-cut: ≥ 6.5
- homfld-YChr-cut: ≥ 6.5
- homfld-WChr-cut: ≥ 6.5
- min-YChr-samples-cut: ≥ 5
- min-WChr-samples-cut: ≥ 5

- priority-order: PolyHighResolution, NoMinorHom, MonoHighResolution, OTV, UnexpectedGenotypeFreq, CallRateBelowThreshold, Other, OtherMA
- recommended: PolyHighResolution, NoMinorHom, MonoHighResolution, Hemizygous
- y-restrict: ≤ 0.2
- min-genotype-freq-samples: ≥ 20
- genotype-p-value-cutoff: ≥ 1E-06

Multi-Allelic SNP QC Thresholds

- HomMMA-cutoff: > 10
- FLD-MA-cutoff: > 5.2
- FLD-MA-2-cutoff: > 5.2
- Min-FLD-MA-cutoff: > 0
- Min-FLD-MA-2-cutoff: > 0
- HetSO-MA-2-cutoff: > -0.1
- HomRO-MA-cutoff: > 0.5
- HomRO-MA-2-cutoff: > 0.5
- HomRO-MA-1-cutoff: > 1
- priority-order-MA: PolyHighResolution, NoMinorHom, MonoHighResolution, Hemizygous, UnexpectedGenotypeFreq, CallRateBelowThreshold, Other, OtherMA
- Best-CR-MA-cutoff: > 90

Analysis Summary

- **Batch Name:** albo_europe_newprior_7Sep23
- **Array Package Name:** Axiom_Aealbo.r1
- **Array Display Name:** Axiom_Aealbo.r1
- **Workflow Type:** Best Practices Workflow
- **Date Created:** 9/7/2023 4:57:21 PM

Sample Summary

- Number of input samples: 454
- Samples passing DQC: 429 out of 454
- Samples passing DQC and QC CR: 423 out of 454
- Samples passing DQC, QC CR and Plate QC: 423 out of 454 (93.172%)
- Number of failing samples: 31
- Number of Samples Genotyped: 423
- Average QC CR for the passing samples: 98.788
- Inbred Penalty Applied: no
- Prior Model File: Axiom_Aealbo.r1.20230907.Europe.models
- SNP List File: none

Plate QC Summary

Plate Barcode	Result	Number of files in a batch	Number of files failing dish QC	Number of files failing QC Call rate	Number of samples that passed	Percent of passing samples	Average call rate for passing samples	Filtered Call Rate
5513584436722042923427	PASSED	21	0	0	21	100	99.1	96.631
5513584436722042923430	PASSED	8	0	0	8	100	98.856	95.884
5513584436722042923435	PASSED	3	0	0	3	100	99.243	96.508
5513584452328010824440	PASSED	42	2	0	40	95.238	98.656	96.1
5513584452328010824443	PASSED	95	3	0	92	96.842	98.722	96.869
5513584452328010824444	PASSED	24	0	0	24	100	99.174	97.459
5513584452328010824445	PASSED	63	0	0	63	100	99.24	97.522
5513584452328010824446	PASSED	95	4	0	91	95.789	98.831	97.055
5513584452328010824447	PASSED	51	12	0	39	76.471	99.155	97.35
5513584452328010824449	PASSED	36	3	4	29	80.556	97.768	94.684
5513584478174020425867	PASSED	10	0	2	8	80	96.073	92.047
5513584478174020425873	PASSED	6	1	0	5	83.333	98.401	94.776

ProbeSet Metrics Summary

- Number of ProbeSets: 175396

ConversionType	Count	Percentage
PolyHighResolution	106231	60.566
Other	37093	21.148
OTV	19430	11.078
NoMinorHom	7146	4.074
MonoHighResolution	4604	2.625
CallRateBelowThreshold	892	0.509

Marker Metrics Summary

- Number of Markers: 175396

- Number of BestandRecommended: 117981
- Percent BestandRecommended: 67.266

ConversionType	Count	Percentage
PolyHighResolution	106231	60.566
Other	37093	21.148
OTV	19430	11.078
NoMinorHom	7146	4.074
MonoHighResolution	4604	2.625
CallRateBelowThreshold	892	0.509

Sample QC Thresholds

- DQC: ≥ 0.82
- QC call_rate: ≥ 90
- Average call rate for passing samples: ≥ 90
- Percent of passing samples: ≥ 90

SNP QC Thresholds

- species-type: Diploid
- cr-cutoff: ≥ 90
- fld-cutoff: ≥ 3.6
- het-so-cutoff: ≥ -0.1
- het-so-XChr-cutoff: ≥ -0.1
- het-so-ZChr-cutoff: ≥ -0.1
- het-so-otv-cutoff: ≥ -0.3
- hom-ro-1-cutoff: ≥ 0.6
- hom-ro-2-cutoff: ≥ 0.3
- hom-ro-3-cutoff: ≥ -0.9
- hom-ro: true
- num-minor-allele-cutoff: ≥ 2
- hom-ro-hap-1-XChr-cutoff: ≥ 0.1
- hom-ro-hap-1-MTChr-cutoff: ≥ 0.4
- hom-ro-hap-1-ZChr-cutoff: ≥ 0.1
- hom-ro-hap-2-XChr-cutoff: ≥ 0.05
- hom-ro-hap-2-MTChr-cutoff: ≥ 0.2
- hom-ro-hap-2-ZChr-cutoff: ≥ 0.05
- aaf-XChr-cut: < 0.36
- aaf-ZChr-cut: < 0.36
- fld-XChr-cut: ≥ 4
- fld-ZChr-cut: ≥ 4
- homfld-XChr-cut: ≥ 6.5
- homfld-ZChr-cut: ≥ 6.5
- homfld-YChr-cut: ≥ 6.5
- homfld-WChr-cut: ≥ 6.5
- min-YChr-samples-cut: ≥ 5
- min-WChr-samples-cut: ≥ 5

- priority-order: PolyHighResolution, NoMinorHom, MonoHighResolution, OTV, UnexpectedGenotypeFreq, CallRateBelowThreshold, Other, OtherMA
- recommended: PolyHighResolution, NoMinorHom, MonoHighResolution, Hemizygous
- y-restrict: ≤ 0.2
- min-genotype-freq-samples: ≥ 20
- genotype-p-value-cutoff: ≥ 1E-06

Multi-Allelic SNP QC Thresholds

- HomMMA-cutoff: > 10
- FLD-MA-cutoff: > 5.2
- FLD-MA-2-cutoff: > 5.2
- Min-FLD-MA-cutoff: > 0
- Min-FLD-MA-2-cutoff: > 0
- HetSO-MA-2-cutoff: > -0.1
- HomRO-MA-cutoff: > 0.5
- HomRO-MA-2-cutoff: > 0.5
- HomRO-MA-1-cutoff: > 1
- priority-order-MA: PolyHighResolution, NoMinorHom, MonoHighResolution, Hemizygous, UnexpectedGenotypeFreq, CallRateBelowThreshold, Other, OtherMA
- Best-CR-MA-cutoff: > 90

S5. Global dataset SNP genotype calls

Analysis Summary

- **Batch Name:** albo_euro_global_1Dec2023
- **Array Package Name:** Axiom_Aealbo.r1
- **Array Display Name:** Axiom_Aealbo.r1
- **Workflow Type:** Best Practices Workflow
- **Date Created:** 12/1/2023 12:11:44 PM

Sample Summary

- Number of input samples: 768
- Samples passing DQC: 722 out of 768
- Samples passing DQC and QC CR: 713 out of 768
- Samples passing DQC, QC CR and Plate QC: 713 out of 768 (92.839%)
- Number of failing samples: 55
- Number of Samples Genotyped: 713
- Average QC CR for the passing samples: 98.732
- Inbred Penalty Applied: no
- Prior Model File: Axiom_Aealbo.r1.generic_prior.txt
- SNP List File: none

Plate QC Summary

Plate Barcode	Result	Number of files in a batch	Number of files failing dish QC	Number of files failing QC Call rate	Number of samples that passed	Percent of passing samples	Average call rate for passing samples	Filtered Call Rate
5513584436722042923427	PASSED	66	7	1	58	87.879	98.913	97.744
5513584436722042923430	PASSED	20	0	0	20	100	99.05	97.684
5513584436722042923432	PASSED	12	0	0	12	100	99.186	98.287
5513584436722042923435	PASSED	15	0	1	14	93.333	99.051	97.94
5513584452328010824440	PASSED	95	10	0	85	89.474	98.689	97.821
5513584452328010824441	PASSED	24	0	0	24	100	99.118	98.344
5513584452328010824443	PASSED	95	3	0	92	96.842	98.737	98.002
5513584452328010824444	PASSED	55	0	0	55	100	98.996	98.237
5513584452328010824445	PASSED	95	1	2	92	96.842	98.829	98.075
5513584452328010824446	PASSED	95	4	0	91	95.789	98.849	98.109
5513584452328010824447	PASSED	83	17	0	66	79.518	98.82	97.99
5513584452328010824448	PASSED	49	0	0	49	100	98.412	97.345
5513584452328010824449	PASSED	36	3	3	30	83.333	97.617	96.685
5513584478174020425867	PASSED	22	0	2	20	90.909	97.612	96.591
5513584478174020425873	PASSED	6	1	0	5	83.333	98.465	97.452

ProbeSet Metrics Summary

- Number of ProbeSets: 175396

ConversionType	Count	Percentage
PolyHighResolution	104503	59.581
Other	42261	24.095
OTV	24700	14.082
NoMinorHom	2427	1.384
CallRateBelowThreshold	793	0.452
MonoHighResolution	712	0.406

Marker Metrics Summary

- Number of Markers: 175396
- Number of BestandRecommended: 107642
- Percent BestandRecommended: 61.371

ConversionType	Count	Percentage
PolyHighResolution	104503	59.581
Other	42261	24.095
OTV	24700	14.082
NoMinorHom	2427	1.384
CallRateBelowThreshold	793	0.452
MonoHighResolution	712	0.406

Sample QC Thresholds

- DQC: ≥ 0.82
- QC call_rate: ≥ 90
- Average call rate for passing samples: ≥ 90
- Percent of passing samples: ≥ 90

SNP QC Thresholds

- species-type: Diploid
- cr-cutoff: ≥ 90
- fld-cutoff: ≥ 3.6
- het-so-cutoff: ≥ -0.1
- het-so-XChr-cutoff: ≥ -0.1
- het-so-ZChr-cutoff: ≥ -0.1
- het-so-otv-cutoff: ≥ -0.3
- hom-ro-1-cutoff: ≥ 0.6
- hom-ro-2-cutoff: ≥ 0.3
- hom-ro-3-cutoff: ≥ -0.9
- hom-ro: true
- num-minor-allele-cutoff: ≥ 2
- hom-ro-hap-1-XChr-cutoff: ≥ 0.1
- hom-ro-hap-1-MTChr-cutoff: ≥ 0.4
- hom-ro-hap-1-ZChr-cutoff: ≥ 0.1
- hom-ro-hap-2-XChr-cutoff: ≥ 0.05
- hom-ro-hap-2-MTChr-cutoff: ≥ 0.2
- hom-ro-hap-2-ZChr-cutoff: ≥ 0.05
- aaf-XChr-cut: < 0.36
- aaf-ZChr-cut: < 0.36
- fld-XChr-cut: ≥ 4
- fld-ZChr-cut: ≥ 4
- homfld-XChr-cut: ≥ 6.5
- homfld-ZChr-cut: ≥ 6.5
- homfld-YChr-cut: ≥ 6.5
- homfld-WChr-cut: ≥ 6.5
- min-YChr-samples-cut: ≥ 5

- min-WChr-samples-cut: ≥ 5
- priority-order: PolyHighResolution, NoMinorHom, MonoHighResolution, OTV, UnexpectedGenotypeFreq, CallRateBelowThreshold, Other, OtherMA
- recommended: PolyHighResolution, NoMinorHom, MonoHighResolution, Hemizygous
- y-restrict: ≤ 0.2
- min-genotype-freq-samples: ≥ 20
- genotype-p-value-cutoff: ≥ 1E-06

Multi-Allelic SNP QC Thresholds

- HomMMA-cutoff: > 10
- FLD-MA-cutoff: > 5.2
- FLD-MA-2-cutoff: > 5.2
- Min-FLD-MA-cutoff: > 0
- Min-FLD-MA-2-cutoff: > 0
- HetSO-MA-2-cutoff: > -0.1
- HomRO-MA-cutoff: > 0.5
- HomRO-MA-2-cutoff: > 0.5
- HomRO-MA-1-cutoff: > 1
- priority-order-MA: PolyHighResolution, NoMinorHom, MonoHighResolution, Hemizygous, UnexpectedGenotypeFreq, CallRateBelowThreshold, Other, OtherMA
- Best-CR-MA-cutoff: > 90

Analysis Summary

- **Batch Name:** albo_euro_global_newprior_1Dec2023
- **Array Package Name:** Axiom_Aealbo.r1
- **Array Display Name:** Axiom_Aealbo.r1
- **Workflow Type:** Best Practices Workflow
- **Date Created:** 12/1/2023 2:51:02 PM

Sample Summary

- Number of input samples: 749
- Samples passing DQC: 722 out of 749
- Samples passing DQC and QC CR: 712 out of 749
- Samples passing DQC, QC CR and Plate QC: 712 out of 749 (95.06%)
- Number of failing samples: 37
- Number of Samples Genotyped: 712
- Average QC CR for the passing samples: 98.702
- Inbred Penalty Applied: no
- Prior Model File: albo_euro_global_1Dec2023_newprior.models
- SNP List File: none

Plate QC Summary

Plate Barcode	Result	Number of files in a batch	Number of files failing dish QC	Number of files failing QC Call rate	Number of samples that passed	Percent of passing samples	Average call rate for passing samples	Filtered Call Rate
5513584436722042923427	PASSED	59	0	1	58	98.305	98.876	96.329
5513584436722042923430	PASSED	20	0	0	20	100	99.003	95.588
5513584436722042923432	PASSED	12	0	0	12	100	99.164	96.889
5513584436722042923435	PASSED	15	0	1	14	93.333	99.014	96.274
5513584452328010824440	PASSED	95	10	0	85	89.474	98.646	96.407
5513584452328010824441	PASSED	24	0	0	24	100	99.087	97.193
5513584452328010824443	PASSED	95	3	0	92	96.842	98.707	96.869
5513584452328010824444	PASSED	55	0	0	55	100	98.969	97.138
5513584452328010824445	PASSED	95	1	2	92	96.842	98.779	96.968
5513584452328010824446	PASSED	95	4	0	91	95.789	98.826	97.096
5513584452328010824447	PASSED	71	5	0	66	92.958	98.79	96.862
5513584452328010824448	PASSED	49	0	0	49	100	98.355	96.108
5513584452328010824449	PASSED	36	3	4	29	80.556	97.766	94.668
5513584478174020425867	PASSED	22	0	2	20	90.909	97.486	94.254
5513584478174020425873	PASSED	6	1	0	5	83.333	98.387	94.755

ProbeSet Metrics Summary

- Number of ProbeSets: 175396

ConversionType	Count	Percentage
PolyHighResolution	106422	60.675
Other	38289	21.83
OTV	22464	12.808
NoMinorHom	5489	3.129
MonoHighResolution	1912	1.09
CallRateBelowThreshold	820	0.468

Marker Metrics Summary

- Number of Markers: 175396
- Number of BestandRecommended: 113823
- Percent BestandRecommended: 64.895

ConversionType	Count	Percentage
PolyHighResolution	106422	60.675
Other	38289	21.83
OTV	22464	12.808
NoMinorHom	5489	3.129
MonoHighResolution	1912	1.09
CallRateBelowThreshold	820	0.468

Sample QC Thresholds

- DQC: ≥ 0.82
- QC call_rate: ≥ 90
- Average call rate for passing samples: ≥ 90
- Percent of passing samples: ≥ 90

SNP QC Thresholds

- species-type: Diploid
- cr-cutoff: ≥ 90
- fld-cutoff: ≥ 3.6
- het-so-cutoff: ≥ -0.1
- het-so-XChr-cutoff: ≥ -0.1
- het-so-ZChr-cutoff: ≥ -0.1
- het-so-otv-cutoff: ≥ -0.3
- hom-ro-1-cutoff: ≥ 0.6
- hom-ro-2-cutoff: ≥ 0.3
- hom-ro-3-cutoff: ≥ -0.9
- hom-ro: true
- num-minor-allele-cutoff: ≥ 2
- hom-ro-hap-1-XChr-cutoff: ≥ 0.1
- hom-ro-hap-1-MTChr-cutoff: ≥ 0.4
- hom-ro-hap-1-ZChr-cutoff: ≥ 0.1
- hom-ro-hap-2-XChr-cutoff: ≥ 0.05
- hom-ro-hap-2-MTChr-cutoff: ≥ 0.2
- hom-ro-hap-2-ZChr-cutoff: ≥ 0.05
- aaf-XChr-cut: < 0.36
- aaf-ZChr-cut: < 0.36
- fld-XChr-cut: ≥ 4
- fld-ZChr-cut: ≥ 4
- homfld-XChr-cut: ≥ 6.5
- homfld-ZChr-cut: ≥ 6.5
- homfld-YChr-cut: ≥ 6.5
- homfld-WChr-cut: ≥ 6.5
- min-YChr-samples-cut: ≥ 5

- min-WChr-samples-cut: ≥ 5
- priority-order: PolyHighResolution, NoMinorHom, MonoHighResolution, OTV, UnexpectedGenotypeFreq, CallRateBelowThreshold, Other, OtherMA
- recommended: PolyHighResolution, NoMinorHom, MonoHighResolution, Hemizygous
- y-restrict: ≤ 0.2
- min-genotype-freq-samples: ≥ 20
- genotype-p-value-cutoff: ≥ 1E-06

Multi-Allelic SNP QC Thresholds

- HomMMA-cutoff: > 10
- FLD-MA-cutoff: > 5.2
- FLD-MA-2-cutoff: > 5.2
- Min-FLD-MA-cutoff: > 0
- Min-FLD-MA-2-cutoff: > 0
- HetSO-MA-2-cutoff: > -0.1
- HomRO-MA-cutoff: > 0.5
- HomRO-MA-2-cutoff: > 0.5
- HomRO-MA-1-cutoff: > 1
- priority-order-MA: PolyHighResolution, NoMinorHom, MonoHighResolution, Hemizygous, UnexpectedGenotypeFreq, CallRateBelowThreshold, Other, OtherMA
- Best-CR-MA-cutoff: > 90
